# Aluminium‐Catalyzed C(sp)−H Borylation of Alkynes

**DOI:** 10.1002/anie.202106216

**Published:** 2021-08-17

**Authors:** Dominic R. Willcox, Daniel M. De Rosa, Jack Howley, Abigail Levy, Alan Steven, Gary S. Nichol, Carole A. Morrison, Michael J. Cowley, Stephen P. Thomas

**Affiliations:** ^1^ EaStCHEM School of Chemistry University of Edinburgh Joseph Black Building, David Brewster Road Edinburgh EH9 3FJ UK; ^2^ AstraZeneca Pharmaceutical Technology and Development Macclesfield Campus Cheshire SK10 2NA UK

**Keywords:** aluminium, borylation, catalysis, main-group chemistry, σ-bond metathesis

## Abstract

Historically used in stoichiometric hydroalumination chemistry, recent advances have transformed aluminium hydrides into versatile catalysts for the hydroboration of unsaturated multiple bonds. This catalytic ability is founded on the defining reactivity of aluminium hydrides with alkynes and alkenes: 1,2‐hydroalumination of the unsaturated π‐system. This manuscript reports the aluminium hydride catalyzed dehydroborylation of terminal alkynes. A tethered intramolecular amine ligand controls reactivity at the aluminium hydride centre, switching off hydroalumination and instead enabling selective reactions at the alkyne C−H σ‐bond. Chemoselective C−H borylation was observed across a series of aryl‐ and alkyl‐substituted alkynes (21 examples). On the basis of kinetic and density functional theory studies, a mechanism in which C−H borylation proceeds by σ‐bond metathesis between pinacolborane (HBpin) and alkynyl aluminium intermediates is proposed.

## Graphical abstract

The aluminium hydride catalyzed dehydroborylation of terminal alkynes is reported. By using a tethered‐intramolecular‐amine Lewis base as a ligand at the aluminium hydride centre the reactivity is controlled. Hydroalumination is switched off and selective reactions at the alkyne C−H σ‐bond are enabled.

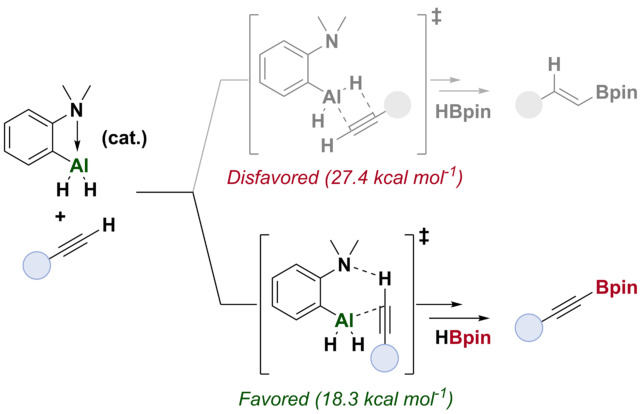

Alkyne hydroalumination is a textbook application of main group species for organic synthesis.^[1]^ The intermediate alkenyl aluminium compounds are rarely isolated, but rather treated in situ with electrophiles to give functionalized alkenes.^[2]^ Recently, we^[3]^ and others^[4]^ have rendered these reactions catalytic and increased the breadth of aluminium catalysis beyond Ziegler–Natta processes, reduction (Meerwein‐Ponndorf‐Verley) and Lewis acid catalysis.^[5]^

Aluminium‐catalyzed hydroboration combines the prototypical alkyne hydroalumination with a turnover step that uses pinacolborane (HBpin) to provide boronic esters and regenerate the aluminium hydride catalyst. However, these reactions were limited to the preparation of alkenyl‐ and alkyl boronic esters.[^3^, ^4^] We thus sought to develop Al−C bond forming reactivity of aluminium hydrides that is not based on hydroalumination, and open aluminium catalysis to the synthesis of other classes of boronic ester.^[6]^ Particularly promising in this regard was the work of Roesky and Zhu, who reported an Al/N frustrated Lewis pair for the stoichiometric dehydrogenative alumination of heterocycles and alkynes.^[7]^

Here, we report an aluminium‐catalyzed dehydrogenative C−H borylation. Using the in situ generated tethered Lewis pair catalyst **1 a**, we “switch off” alkyne hydroalumination to favour C−H alumination. The resulting alkynyl aluminium species react with HBpin to provide alkynyl boronic esters and regenerate the aluminium hydride. This C−H borylation is complementary to the typical reactivity of transition metal catalysts with HBpin and alkynes, which overwhelmingly results in hydroboration.^[8]^

To favour dehydrogenative C−H alumination over hydroalumination, we designed the aluminium dihydride **1 a** by adapting principles from previously reported aluminium‐ and boron‐based intramolecular FLPs.[^7^, ^9^] Our hypothesis was that the rigid aromatic backbone bearing a “hard” amine donor *ortho* to the aluminium centre would increase the basicity of the aluminium hydrides, and quench the Lewis acidity of the aluminium centre.

A boron analogue of the aluminium dihydride **1 a** reacts with alkynes by hydroboration,^[9b]^ reflecting the low barrier for that reaction (compared to deprotonation). We carried out preliminary DFT calculations [ωB97XD/6‐311++G(d,p)] to examine the relative barriers to alkyne hydroalumination and dehydrogenative C−H alumination. Computationally, the anilino‐ligand of the alane **1 a** provides substantial energetic discrimination between the two pathways, with the activation barrier for hydroalumination being 9.1 kcal mol^−1^ higher than that predicted for C−H alumination (Scheme 1, c).

**Scheme 1:**
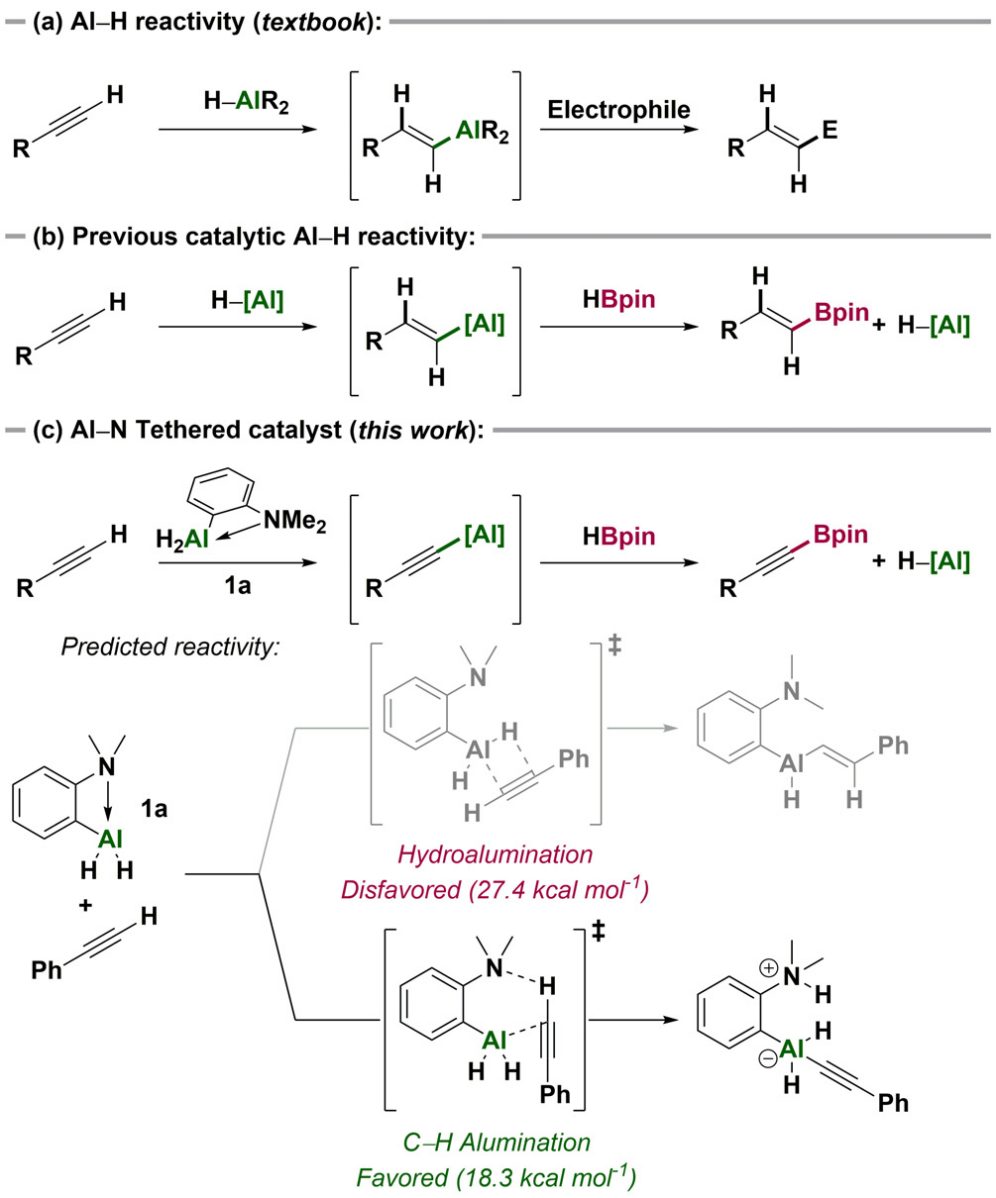
(a) Stoichiometric hydroalumination of alkynes. (b) Catalytic aluminium hydride reactivity with alkynes. (c) Hydroalumination versus C−H alumination of alkynes.

The prediction of good discrimination between the two possible pathways encouraged us to seek experimental verification. We have previously used alkylalanes and HBpin to generate catalytically‐active aluminium hydrides under reaction conditions.^[3]^ Adopting the same concept, we prepared the dimethylalane **1 b** in 54 % yield by reaction of 2‐lithio‐*N*,*N*‐dimethylaniline with Me_2_AlCl, as a precursor to the dihydride **1 a**.

The dimethylalane **1 b** is a pre‐catalyst for C−H borylation of alkynes, generating dihydride **1 a** in situ. Reaction of 1‐ethynyl‐4‐fluoro‐benzene with HBpin in the presence of dimethylalane **1 b** (10 mol %) gave only trace amounts of alkynyl boronic ester **3** at room temperature (Table 1). At higher temperatures, we observed formation of alkynyl boronic ester **3** (30 %), dihydrogen and significant hydroboration (entries 2 and 3). That alkyne hydroboration was competitive with C−H borylation seemed at odds with the predicted large barrier to hydroalumination by **1 a** (Scheme 1, c). Furthermore, Roesky and Zhu reported high selectivity for dehydrogenative alumination of alkynes with a related aluminium dihydride.^[7]^ We postulated that the hydroboration product could instead be the result of hidden BH_3_‐catalyzed hydroboration.^[10]^ Monitoring the reaction of **1 b** with HBpin by ^11^B NMR spectroscopy revealed signals attributable to BH_3_ (SI section 2.3). The same was observed when monitoring catalytic reactions.

**Table 1:** Catalyst optimization. 
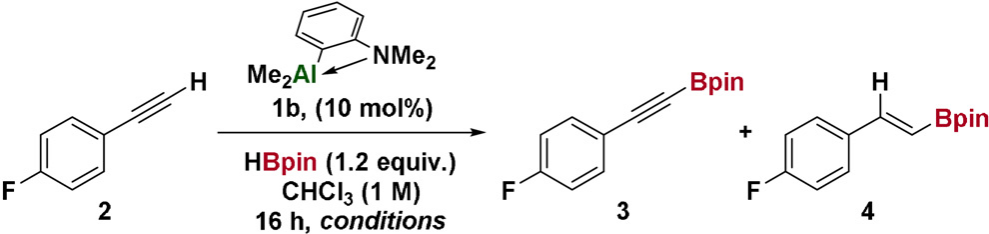

Entry	Conditions	**3** [%]	**4** [%]
1	r.t.	trace	trace
2	60 °C	30	8
3	80 °C	50	14
4	80 °C with 0.1 equiv 1,5‐COD	72	20
5	80 °C with 1.0 equiv 1,5‐COD	89	8
6	80 °C with 0.8 equiv 1,5‐hexadiene	90	3

Yields determined by ^19^F NMR spectroscopy using fluorobenzene as an internal standard 1,5‐COD=1,5‐cyclooctadiene.

To suppress the unwanted reaction of BH_3_ with the substrate, we investigated the addition of dienes as BH_3_ traps (see SI section 2.2). Reaction in the presence of 0.1 equivalents of 1,5‐cyclooctadiene (COD) showed the formation of cylcooctenyl‐*B*‐9‐BBN, and increased selectivity for the C−H borylation product (72:20, Table 1, entry 4). Using 1 equivalent of COD further improved the yield and selectivity (entry 5). 1,5‐Hexadiene^[11]^ gave the greatest selectivity (entry 6, see also SI section 2.2).

Control reactions confirm pre‐catalyst **1 b** is needed for C−H borylation. Reactions in the absence of **1 b** led only to recovery of starting materials. The bifunctional alane‐amine structure of **1 a/1 b** is required for C−H borylation: neither PhAlMe_2_ alone nor in combination with an external amine (*N*,*N*‐dimethylaniline) are active for catalytic C−H borylation.

We next examined the substrate scope of the Al‐catalyzed C−H borylation (Table 2). *Ortho*‐, *meta*‐, and *para*‐substituted arylalkynes **3 b**–**d** showed equal reactivity and selectivity for C−H borylation over hydroboration. Anisole and aniline derivatives **3 f**,**g**, competing Lewis bases, did not perturb reactivity or selectivity. Reaction of dialkyne **3 h** proceeded to give the diborylated species. A CF_3_ group led to substantially reduced reactivity (**3 i**), which was not observed for aryl chloride or fluoride substituents (**3 j**,**k**). Chemoselectivity was maintained with a thiophene‐substituted alkyne **3 l** and an internal alkene **3 o**. Ethynylcyclopropane **3 q** reacted without observable ring‐opening, and the trimethylsilyl group in **3 r** was retained. An alkyl chloride **3 s**, thiol ether **3 t** and ether **3 u** were all tolerated with no substitution or elimination observed. The synthetic utility of the alkynyl boronic esters was demonstrated by Carboni‐Lindsey reaction with a 1,2,4,6‐tetrazine^[12]^ to give a pyridazine **4** and BH_3_‐catalyzed hydroboration to give a diborylated alkene **5**.^[10a]^

**Table 2:** Scope of C−H borylation of terminal alkynes and their applications.

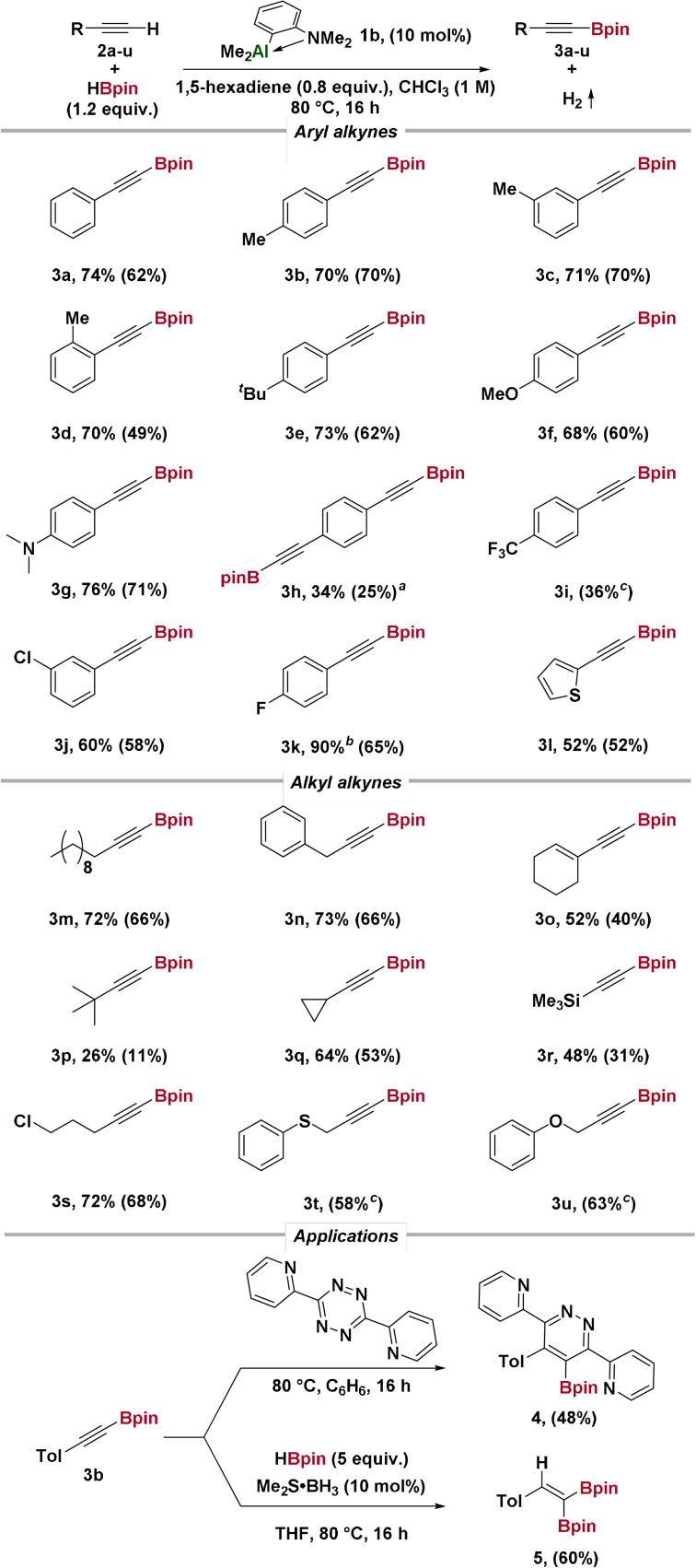

Yields determined by ^1^H NMR spectroscopy using 1,3,5‐trimethyoxybenzene as an internal standard (isolated yields in parentheses). Reaction conditions: substrate (1.0 mmol), HBpin (1.2 mmol), 1,5‐hexadiene (0.8 mmol), **1 b** (0.1 mmol), CHCl_3_ (1.0 mL), 80 °C, and 16 h. [a] 0.5 mmol of substrate. [b] Yield determined by ^19^F NMR spectroscopy with fluorobenzene as an internal standard. [c] 20 mol % **1 b**.

Under reaction conditions there are two possibilities for generating the active aluminium dihydride catalyst **1 a** from the dimethyl pre‐catalyst **1 b**. Al/B exchange with two equivalents of HBpin would generate **1 a** and MeBpin.^[3]^ Alternatively, deprotonation of the alkyne by **1 b** would generate methane^[13]^ and a bis(alkynyl)aluminium compound which could enter the catalytic cycle after Al/B exchange.

Using ^19^F NMR spectroscopy, we monitored the reaction of 4‐fluorophenylacetylene **2 k** with HBpin in the presence of 10 mol % pre‐catalyst **1 b** at 80 °C (Scheme 2). In the initial stages of the reaction, alkyne consumption and product formation were rapid. After this initial period, the rate of reaction decreased. In the first 10 minutes, approximately 10 % of the substrate was consumed, corresponding to the formation of 10 % of borylated product (Scheme 2). The total consumption of substrate during the initial period was always equivalent to the catalyst loading (see SI 8.2). Accompanying this, we observed the generation of CH_4_ by ^1^H and ^13^C NMR spectroscopy, and MeBpin in the ^11^B NMR spectrum. Using D‐4‐fluorophenylacetylene *d_1_* **‐2 k**, DCH_3_ was observed. The simultaneous observation of methane and MeBpin suggests two concurrent pathways for pre‐catalyst activation. We thus examined in turn the stoichiometric reactivity of the dimethylalane pre‐catalyst **1 b** with alkyne and HBpin.

**Scheme 2:**
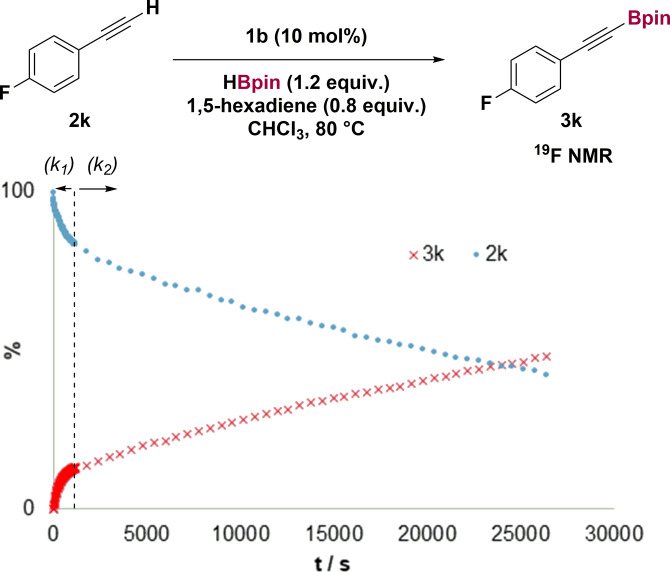
Reaction profile using 4‐fluorophenylacetylene **2 k**, monitored by ^19^F NMR spectroscopy.

Reaction of pre‐catalyst **1 b** with excess phenylacetylene rapidly generated the tris‐acetylide species **6**, which was structurally characterized (Scheme 3, a). The formation of the tris‐acetylide **6** results from protonolysis of all Al−C bonds in **1 b**, generating methane and *N*,*N*‐dimethylaniline, which coordinates to the aluminium centre. Under catalytic conditions this would generate methane (as observed) and an alkynyl‐aluminium species which would then undergo Al/B exchange with HBpin to generate the product **3 a**. Treatment of the tris‐acetylide **6** with HBpin gave alkynyl boronic ester **3 a** in a reduced yield (39 %). The tris‐acetylide **6** is not catalytically active, presumably because the dimethyl‐aniline unit is no longer covalently attached to the aluminium centre.

**Scheme 3:**
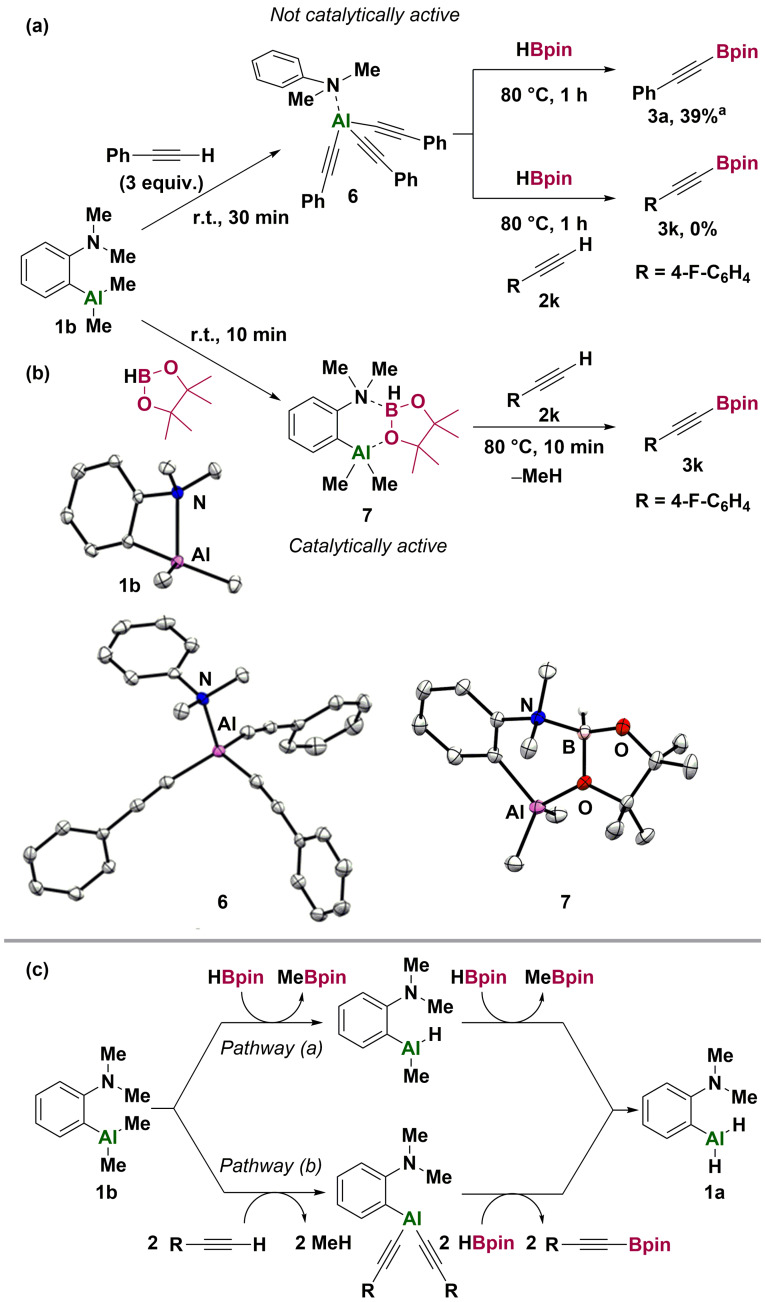
(a) Formation and reactivity of tris(phenylacetylide)aluminium‐*N*,*N*‐dimethylaniline **6**. (b) Formation and reactivity of **1 b**‐HBpin adduct **7**. (c) Proposed catalyst activation pathways. Thermal ellipsoids shown at 50 % probability level. Hydrogen atoms except B−H omitted. [a] Yield determined by comparison to *N*,*N*‐dimethylaniline.^[23]^

With HBpin, pre‐catalyst **1 b** gave the adduct **7** at room temperature, indicated by the observation of a signal in the ^11^B NMR spectrum at *δ*=6.0 (d, ^1^ *J*_B−H_=135 Hz). Crystallography revealed coordination of the amine of **1 b** to the boron centre of HBpin. The aluminium centre is maintained as four‐coordinate by coordination of an HBpin oxygen. Heating **7** to 80 °C generated MeBpin; we infer corresponding formation of the Al‐H functionality. When **7** is heated in the presence of alkyne, rapid formation of borylated alkyne **3**, MeBpin and CH_4_ occurs, as observed in catalysis (Scheme 3, b).

Based on these observations, we propose that catalyst activation occurs through two distinct and concurrent pathways to give the common Al‐H catalyst **1 a**. In pathway (a), Al/B exchange with HBpin generates MeBpin and Al‐H functionality. In pathway (b), deprotonation of the alkyne by **1 b** generates methane and alkynyl‐aluminium species which can undergo Al/B exchange with HBpin, generating product and the Al‐H catalyst **1 a** (Scheme 3, c). Both of these pathways were found to be viable by DFT calculations, with pathway (a) having a much higher barrier to activation (28.7 kcal mol^−1^) compared to the alkyne‐deprotonation pathway (b) (18.7 kcal mol^−1^) (Figure 1).

**Figure 1:**
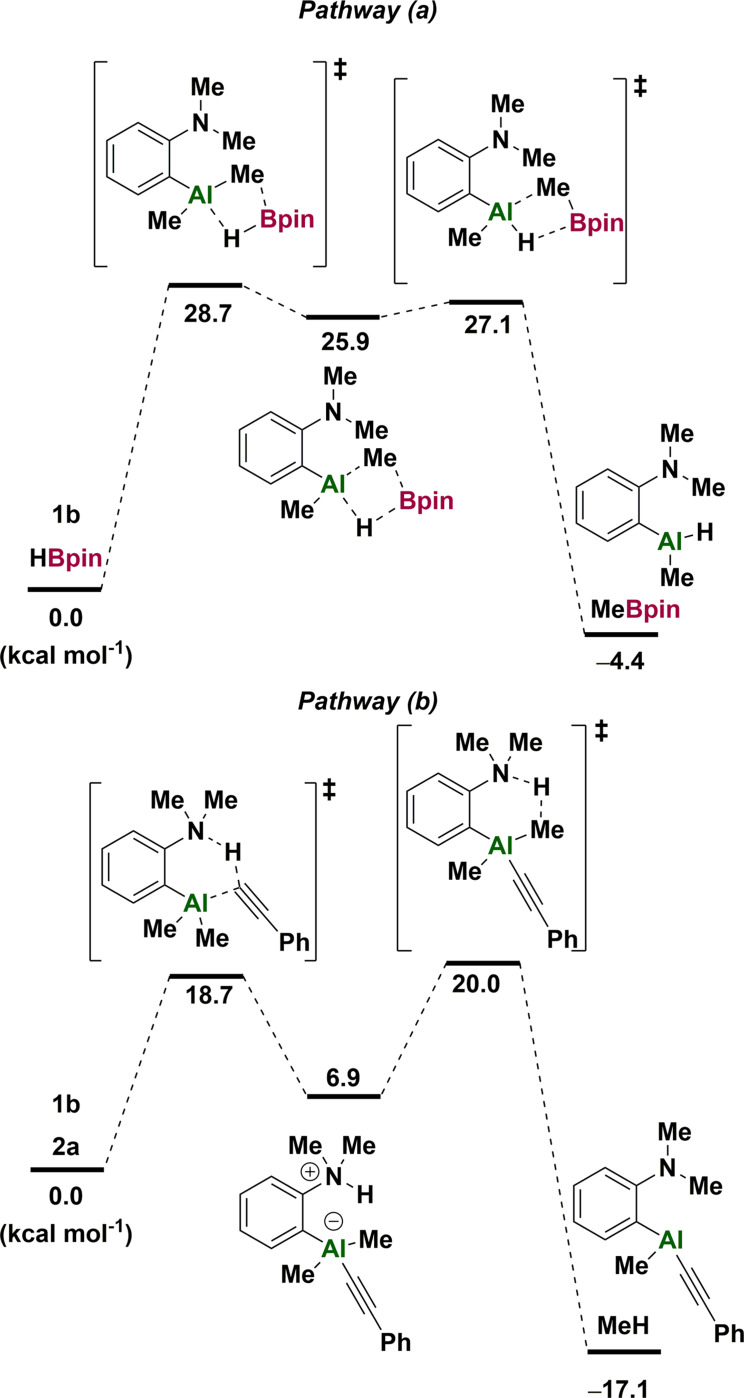
DFT‐computed free energies for the two possible catalyst activation pathways of **1 a**. (Energies calculated at ωB97XD/6‐311++G(d,p) on ωB97XD/6‐31+G(d,p)‐optimised structures).

From our initial kinetic study, it is clear that catalyst activation generates product more rapidly than under steady‐state conditions. Thus, alkyne deprotonation is more rapid from the dimethyl aluminium pre‐catalyst **1 b** than the dihydride catalyst **1 a**, in line with the expected reactivity differences of alkyl‐ and hydrido‐aluminium compounds.^[14]^

Dividing the reaction into two phases, we determined rates for the initiation phase with rate *k*_1_ and “turnover” phase with rate *k*_2_. Using time‐normalization kinetics,^[15]^ the order of the reaction in each component was determined for the “turnover” phase (*k*_2_ *)* (Scheme 4, a). The reaction was found to be 1^st^ order with respect to alkyne, and 2^nd^ order with respect to catalyst, suggesting the active catalyst **1 a** exists in solution largely as a dimer, [**1 a**]_2_, which is reacting directly with alkyne in the rate‐limiting step.

**Scheme 4:**
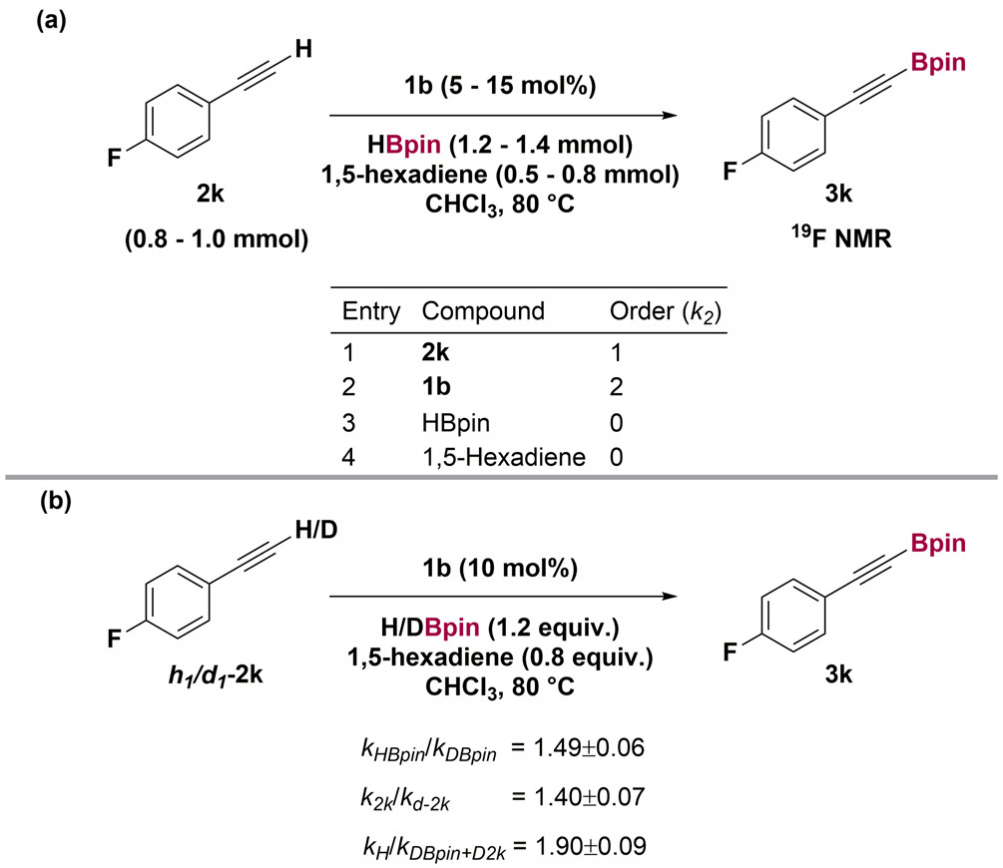
(a) Reactant orders from variable time normalization analysis (VTNA). (b) Kinetic isotope effect with D‐4‐fluorophenylacetylene *d_1_*‐**2 k** and DBpin.

Consistent with the presence of an oligomeric catalyst species under reaction conditions,^[16]^ attempts to isolate the dihydride **1 a** (by reaction of 2‐lithio‐*N*,*N*‐dimethylaniline with H_2_AlCl), gave a tetrameric aluminium species **8**. The alane **8** is formally a tetramer of **1 a** (i.e. the molecular formulae of **8** and 4[**1 a**] are the same). The tetramer **8** can be considered a hydride‐bridged dimer of an AlH_3_ adduct of a bis(anilino)aluminium hydride (Figure 2). Each aluminium centre in the tetramer **8** is 5‐coordinate. The tetramer **8** is catalytically competent for dehydrogenative alkyne hydroboration, though a significant level of hydroboration was observed, presumably catalyzed by AlH_3_ released from the tetramer **8**.^[3]^ Using DFT [ωB97XD/6‐311++G(d,p)]), we calculated the relative energies of the catalyst **1 a**, dimeric catalyst [**1 a**]_2_, [anilino]_2_AlH⋅AlH_3_, and its hydride‐bridged dimer **8**. All four compounds were within 4.4 kcal mol^−1^ of each other (see SI 9.5).

**Figure 2:**
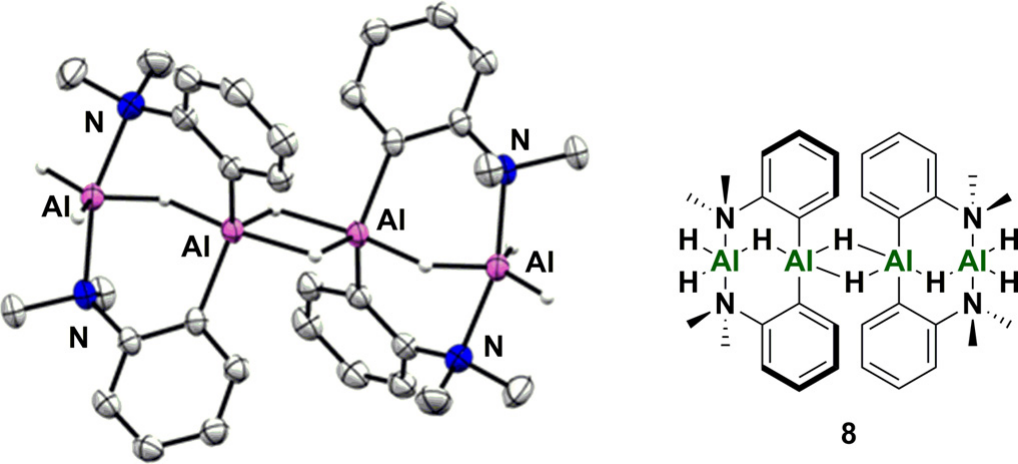
Crystal structure of dihydride oligomer **8**. Thermal ellipsoids shown at 50 % probability level. Hydrogen atoms except Al‐H omitted.^[23]^

Returning to the kinetic studies, we sought preliminary evidence for the operative catalytic mechanism beyond the catalyst activation stage. We determined kinetic isotope effects in the “turnover” phase of the reaction (i.e. *k*_2_) using both DBpin and *D*‐4‐fluorophenylacetylene (*d*_1_ **‐2 k**), substituting each reagent in turn and then both together (Scheme 4, b). Both the B−H and C−H bond KIE values are small (*k*_H_/*k*_D_ B−H 1.5; C−H 1.4). ^2^H NMR spectra during these experiments excluded isotopic scrambling between starting materials as an explanation for the small isotope effects.^[17]^

We can readily exclude σ‐bond metathesis of Al−C and B−H bonds as the rate‐limiting step in the C−H borylation of alkynes by the catalyst **1 a** since: i) the reaction is zero order in HBpin and, ii) *k*_H_/*k*_D_(B−H), at 1.5, is substantially smaller than expected for a process in which σ‐bond metathesis is rate‐limiting.^[18]^ We instead explain the observed value of *k*_H_/*k*_D_(B−H) as a Al‐H/Al‐D isotope effect, since HBpin generates Al‐H functionality during activation and in the catalytic cycle (see below).

The reaction is second order in the pre‐catalyst **1 b** and first order in the alkyne substrate.^[19]^ These measurements indicate that the rate‐limiting step is deprotonation of the alkyne C−H and that this is effected a dimeric aluminium species, for example, [**1 a**]_2_ (see SI 9.5). The small *k*_H_/*k*_D_(C−H) value, at 1.4, is typical for asynchronous deprotonation during the rate‐limiting step.^[20]^ The inferred (see above) *k*_H_/*k*_D_(Al‐H) of 1.5 is of the correct magnitude for a primary isotope effect (predicted value 2.1, see SI S8.4) and indicates that at least one Al−H bond is broken during this step.

With the combined evidence, we propose a catalytic cycle (Scheme 5) in which dimeric catalyst [**1 a**]_2_ deprotonates the alkyne to form the zwitterionic acetylide **9**, necessitating cleavage of a bridging Al−H bond. Subsequent H_2_ elimination precedes Al/B exchange which occurs by σ‐bond metathesis (as during catalyst activation), regenerating the bridged aluminium dihydride [**1 a**]_2_.

**Scheme 5:**
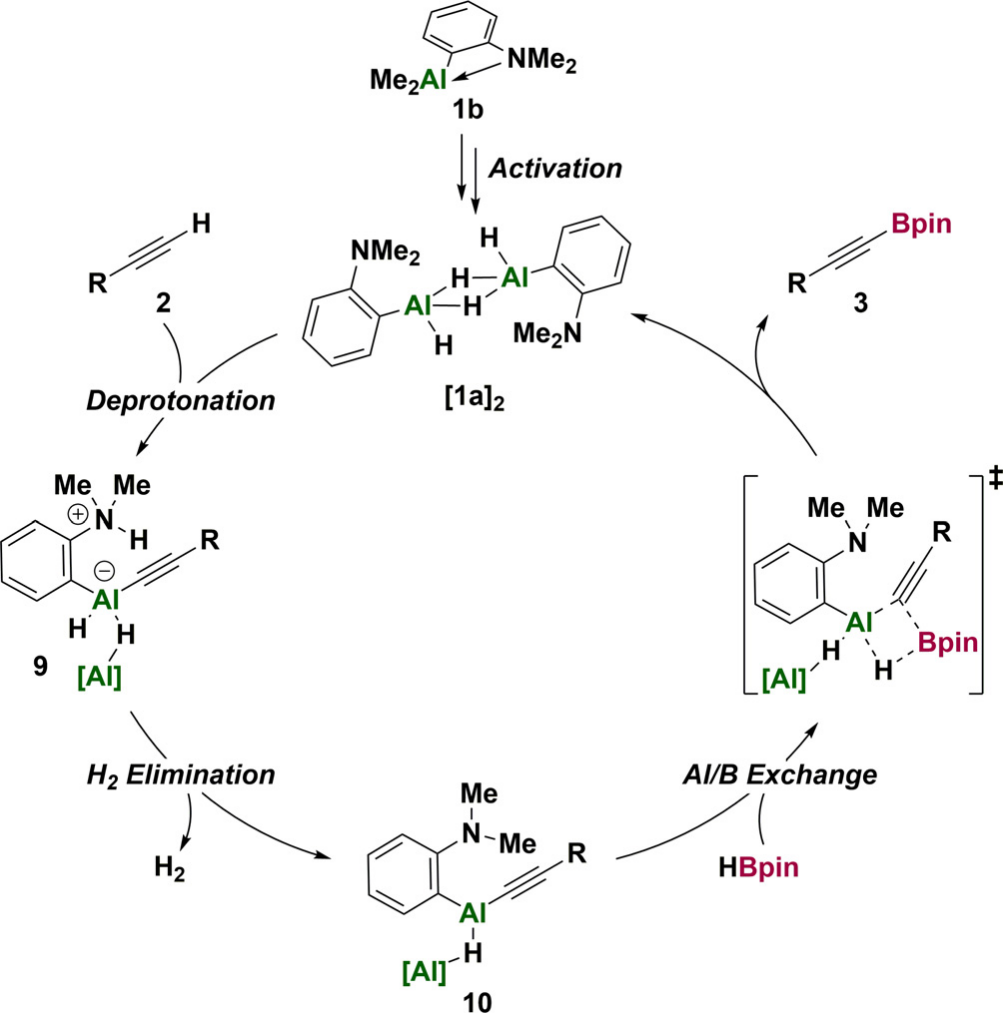
Proposed catalytic cycle.

As main group compounds continue to find increasing use as catalysts^[21]^ chemists will need new strategies to control reactivity at the main‐group centre, mirroring the journey to establish ligand design principles in transition‐metal chemistry.^[22]^ Here, we have shown how a pendant amine functionality can alter the preference of aluminium for hydroalumination of alkynes and instead enable selective catalytic functionalization of the C−H σ bonds. Our findings suggest new principles for ligand design in the growing field of aluminium‐catalyzed functionalization.[^3^, ^4^] We are currently undertaking a detailed mechanistic study to establish the precise nature of the catalytic cycle in this reaction.

## Conflict of interest

The authors declare no conflict of interest.

## Supporting information

As a service to our authors and readers, this journal provides supporting information supplied by the authors. Such materials are peer reviewed and may be re‐organized for online delivery, but are not copy‐edited or typeset. Technical support issues arising from supporting information (other than missing files) should be addressed to the authors.

Supporting Information
