# Supplementary material for: Aluminium‐Catalyzed C(sp)−H Borylation of Alkynes
Source: Angew Chem Int Ed Engl. 2021 Aug 17;60(38):20672–7. doi: 10.1002/anie.202106216 (PMC8518654; doi:10.1002/anie.202106216)
Supplement: Supplementary file 1 — Supporting Information [file ANIE-60-20672-s001.pdf]

Angewandte Chemie
Eine Zeitschrift der Gesellschaft Deutscher Chemiker

Supporting Information

Aluminium-Catalyzed C(sp)–H Borylation of Alkynes

Dominic R. Willcox[+], Daniel M. De Rosa[+], Jack Howley, Abigail Levy, Alan Steven, Gary S. Nichol, Carole A. Morrison, Michael J. Cowley, and Stephen P. Thomas**

anie_202106216_sm_miscellaneous_information.pdf

Supporting Information

Contents

1. General Considerations S2
2. Optimization S4
   2.1. Solvent Screening S4
   2.2. BH_3 Trap Screening S6
   2.3. BH_3 Trap Mechanism S7
3. Control Reactions S9
4. C–H Borylation of Alkynes Scope S11
5. Reactions of Alkynyl Boronic Esters S34
6. Synthesis of Aluminium Compounds S36
7. Preparation of Deuterated Compounds S42
8. Mechanistic Analysis S44
   8.1. Deuterium Labelling S44
   8.2. VTNA Kinetics S46
   8.3. Kinetic Isotope Effect S56
   8.4. Predicted Primary KIEs S66
   8.5. Variable Temperature Experiments on HBpin-**1b** Adduct S67
   8.6. Catalytic Competence of Alane **8** S68
   8.7. Al/B Exchange in Tris-Acetylide **6** S69
   8.8. Catalytic Competence of Tris-Acetylide **6** S71
9. Computational Details S75
   9.1. Computational Details S75
   9.2. Energy Profile for Terminal Alumination S76
   9.3. Energy Profile for Hydroboration S77
   9.4. Catalyst Activation Pathways S78
   9.5. Optimized Structures of Aluminium Dihydride Monomers, Dimers and Oligomers S80
   9.6. Cartesian Coordinates for Quantum Chemical Calculations S81
10. NMR Spectra of Isolated Compounds S82
11. References S169

1. General Considerations

Reaction Setup: All reactions were performed in oven (185 °C) and/or flamed-dried glassware under an atmosphere of anhydrous nitrogen or argon, unless otherwise indicated. All air- and moisture sensitive reactions were carried out using standard vacuum line and Schlenk techniques, or in a glovebox with a purified argon atmosphere. All glassware was cleaned using base (KOH, ^{i}PrOH) and acid (HCl_{aq}) baths. All reported reaction temperatures correspond to external bath temperatures. Room temperature (r.t.) was approximately 22 °C.

NMR Spectroscopy: ^{1}H, ^{11}B, ^{13}C, ^{19}F and ^{29}Si NMR spectra were recorded on Bruker Avance III 400 and 500 MHz; Bruker AVI 400 MHz; Bruker Avance I 600 MHz spectrometers. Chemical shifts are reported in parts per million (ppm). ^{1}H and ^{13}C NMR spectra were referenced to the residual solvent peak ($CHCl_3$: 7.27 ppm, 77.00 ppm; C_6H_6: 7.16 ppm, 128.06 ppm).[1] Multiplicities are indicated by app. (apparent), br. (broad), s (singlet), d (doublet), t (triplet), q (quartet), quin. (quintet), sext. (sextet), sept. (septet), non. (nonet). Coupling constants, *J*, are reported in Hertz and rounded to the nearest 0.1 Hz. Integration is provided.

Infrared Spectroscopy: Infra-red (IR) spectra were recorded on a Shimadzu IRAffinity-1 spectrometer (serial no. A213749) spectrometer. Relevant peaks are reported in cm^{-1}.

Mass Spectrometry: Mass spectrometry (MS) was performed by the University of Edinburgh, School of Chemistry, Mass Spectrometry Laboratory. High resolution mass spectra were recorded on a VG autospec, or Thermo/Finnigan MAT 900, mass spectrometer. Electron Impact (EI^+) spectra were performed at 70 eV using methane as the carrier gas, with either a double focusing sector field (DFSF) or time-of-flight (TOF) mass analyzer. Chemical Ionization (CI^+) spectra were performed with methane reagent gas, with either a double focusing sector field (DFSF) or time-of-flight (TOF) mass analyzer. Electrospray Ionization (ESI^+) spectra were performed using a time-of-flight (TOF) mass analyzer. Data are reported in the form of m/z (intensity relative to the base peak = 100).

Melting Points: Melting points (mp) were determined on a Stuart Scientific SMP10, or Griffin Gallankamp, melting point apparatus in capillary tubes and are uncorrected.

Chromatography: Analytical thin-layer chromatography was performed on aluminium-backed silica plates (Merck 60 F_{254}). Pet. ether refers to petroleum ether 40-60. Product spots were visualised by UV light at 254 nm, and subsequently developed using potassium permanganate solution if appropriate. Flash column chromatography was performed on silica gel (Merck Kielselgel 60, 40-63 μm) unless otherwise stated.

Solvents: All solvents for air- and moisture sensitive techniques were obtained from an anhydrous solvent system (Innovative Technology). Anhydrous C_6D_6 was distilled from sodium/benzophenone. Reaction solvents tetrahydrofuran (THF) (Fisher, HPLC grade), ether (Et_2O) (Fisher, BHT stabilized ACS grade), and dichloromethane (CH_2Cl_2) (Fisher, unstabilized HPLC grade) were dried by percolation through two columns packed with neutral alumina under a positive pressure of argon. Reaction solvent toluene (ACS grade) was dried by percolation through a column packed with neutral alumina and a column packed with Q5 reactant (supported copper catalyst for scavenging oxygen) under a positive pressure of argon. Solvents for filtration, transfers, cromatography, and recrystallization were dichloromethane (CH_2Cl_2) (ACS grade, amylene stabilized), ether (Et_2O) (Fisher, BHT stabilized ACS grade), ethyl acetate (EtOAc) (Fisher, ACS grade), hexane (Optima), methanol (MeOH) (ACS grade), pentane (ACS grade), and petroleum ether (40–60°C, ACS grade).

Chemicals: All reagents were purchased from Merck, Alfa Aesar, Acros organics, Tokyo Chemical Industries UK, Fluorochem and Apollo Scientific or synthesised within the laboratory. $AlMe_2Cl$ (1.0 M in hexanes) was transferred into an airtight J. Young's tap ampoule and then used as supplied. All other reagents were used as supplied unless indicated otherwise.

2. Optimization

2.1. Solvent Screening

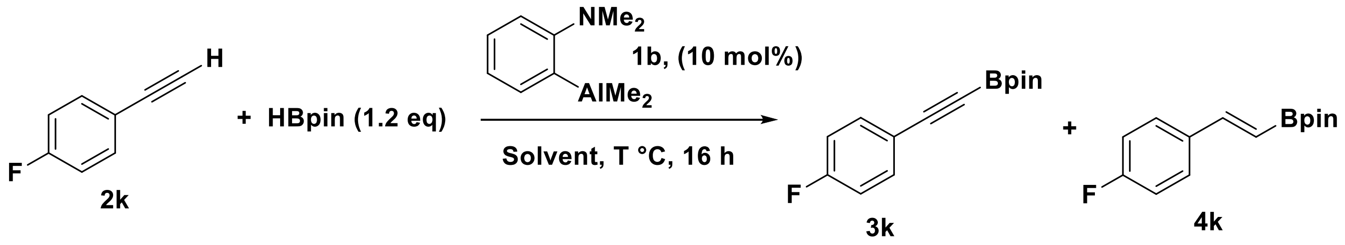

Table S1. Solvent Screening

Entry	Solvent	T / °C	**3k** / %	**4k** / %	Conversion / %
1	$CHCl_3$	60	30	8	50
2	$CHCl_3$	80	50	14	80
3	CH_2Cl_2	60	14	6	38
4	C_6D_6	25	trace	trace	8
5	C_6D_6	60	14	4	68
6	C_6D_6	80	48	14	62
7	Et_2O	60	7	4	42
8	THF	60	9	1	46
9	*n*-Hexane	60	21	0*	28
10	*n*-Pentane	60	28	8	64
11	MeCN	60	0	0	0
12	Toluene	60	12	6	46
13	None	60	16	10	54
14	Cyclohexane	60	24	4	54
15	Cyclohexane	80	32	4	84
16	*n*-Heptane	60	20	6	48
17	*n*-Heptane	80	28	18	76

*__4k__ was insoluble so was not observed

Under an inert atmosphere, 2-dimethylaluminium-*N,N*-dimethylaniline **1b** (9.0 mg, 0.050 mmol) and HBpin (0.600 mmol, 87.0 µL) were dissolved in solvent (0.500 mL). 4'-fluorophenylacetylene (0.500 mmol, 57.0 µL) was added and the mixture heated at T °C for 16 hours. NMR yields were calculated by ^{19}F NMR (fluorobenzene as internal standard) of the crude reaction mixture.

Chloroform and C_6D_6 at 80 °C gave comparable yields of the desired product, but chloroform afforded better selectivity so was taken forward for further optimization. In some cases where conversion was poor, a number of by-products were formed, which we were unable to identify.

2.2. BH_3 Trap Screening

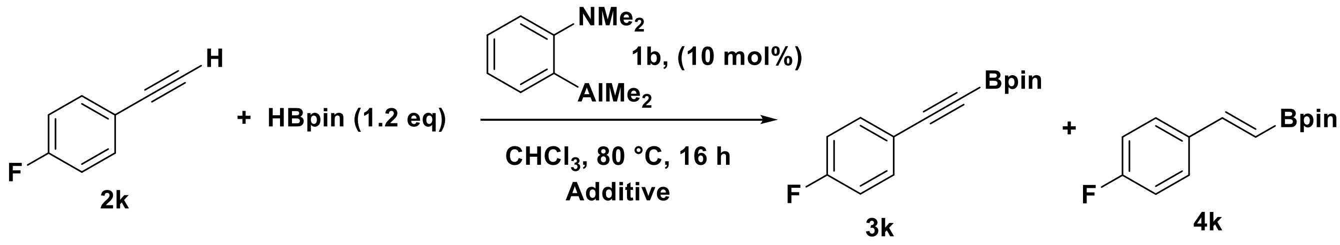

Table S2. Alkene Screening

Entry	Additive	**3k** / %	**4k** / %	Conversion / %
1	No additive	50	14	80
2	1,5-Cyclooctadiene	93	5	98
3	Norbornene	38	32	70
4	Norbornadiene	48	28	76
5	3,3-Dimethylbutene	42	14	56
6	2,3-Dimethylbutadiene	48	10	58
7	Cyclohexene	38	32	70
8	1,5-Hexadiene	90	3	93
9	Styrene	52	10	70

Under an inert atmosphere, 2-dimethylaluminium-*N,N*-dimethylaniline **1b** (9.0 mg, 0.050 mmol) and HBpin (0.600 mmol, 87.0 µL) were dissolved in chloroform (0.500 mL). Any additive, followed by 4'-fluorophenylacetylene (0.500 mmol, 57.0 µL) was added and the mixture heated at 80 °C for 16 hours. NMR yields were calculated by ^{19}F NMR (fluorobenzene as the internal standard) of the crude reaction mixture.

1,5-Cyclooctadiene and 1,5-hexadiene gave a large increase in the selectivity and yield of the reaction, diminishing the proportion of hydroboration product. Due to its slightly better selectivity over multiple runs and substrates, 1,5-hexadiene was chosen as the ideal additive.

In many cases (e.g. cyclohexene), mono-alkene additives increased the proportion of hydroboration product **4k** substantially. In the case of cyclohexene, we attribute this to the formation of Cy_2BH, which is a very effective catalyst for the hydroboration of alkynes with HBPin.[2]

2.3. BH_3 Trap Mechanism

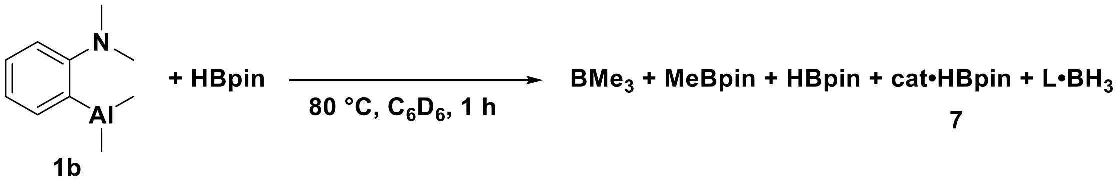

Upon heating 2-dimethylaluminium-*N,N*-dimethylaniline **1b** (0.100 mmol, 18.0 mg) with HBpin (0.100 mmol, 15.0 µL) at 80 °C for an hour, a number of group-13 exchange products were observed. Trace BH_3 is seen, which was likely responsible for the background hydroboration.

^{11}B NMR (128.34 MHz, C_6D_6):

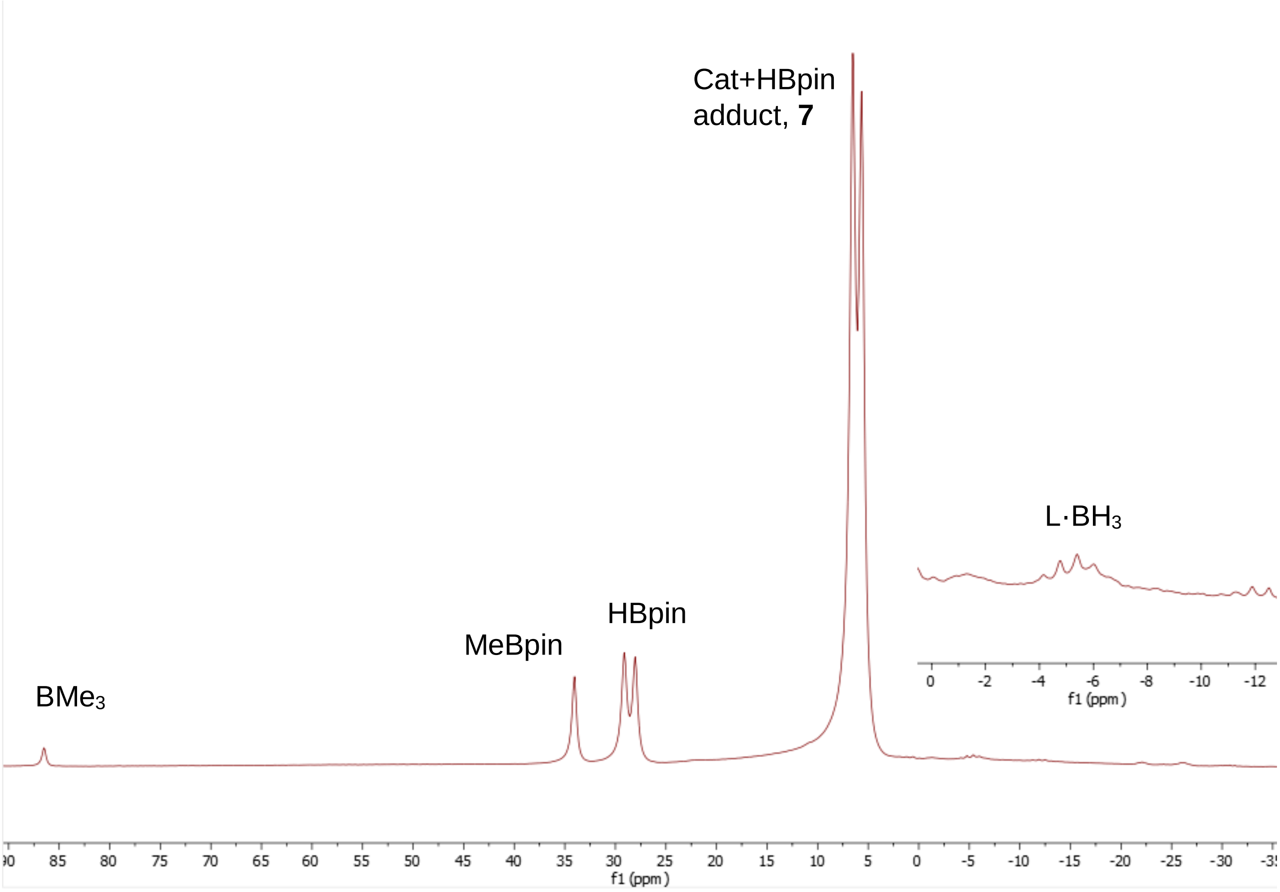

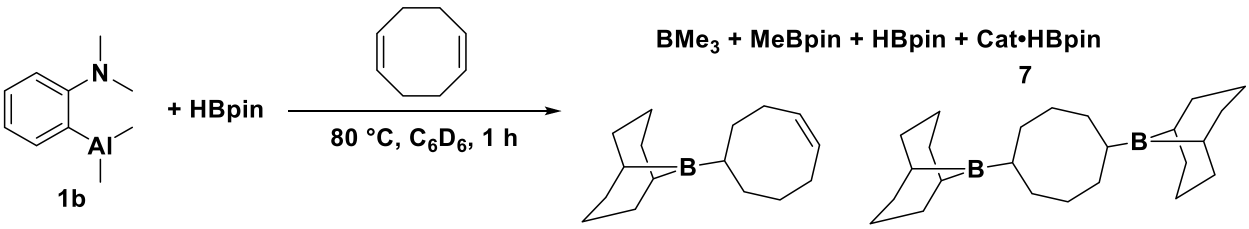

Upon heating 2-dimethylaluminium-*N,N*-dimethylaniline **1b** (0.100 mmol, 18 mg) with HBpin (0.100 mmol, 15.0 µL) and 1,5-cyclooctadiene (0.100 mmol, 12.0 µL) at 80 °C for 1 hour, two

more trialkylboranes were observed which we characterise as multiple hydroboration products of 1,5-cyclooctadiene. We confirmed this by taking H-*B*-9-BBN and 1,5-cyclooctadiene and heating it to 80 °C, giving a broad signal at 81 – 91 ppm in the ^{11}B NMR. This demonstrates that the trace BH_3 was trapped by dienes such as 1,5-cyclooctadiene under reaction conditions.

^{11}B NMR (128.34 MHz, C_6D_6):

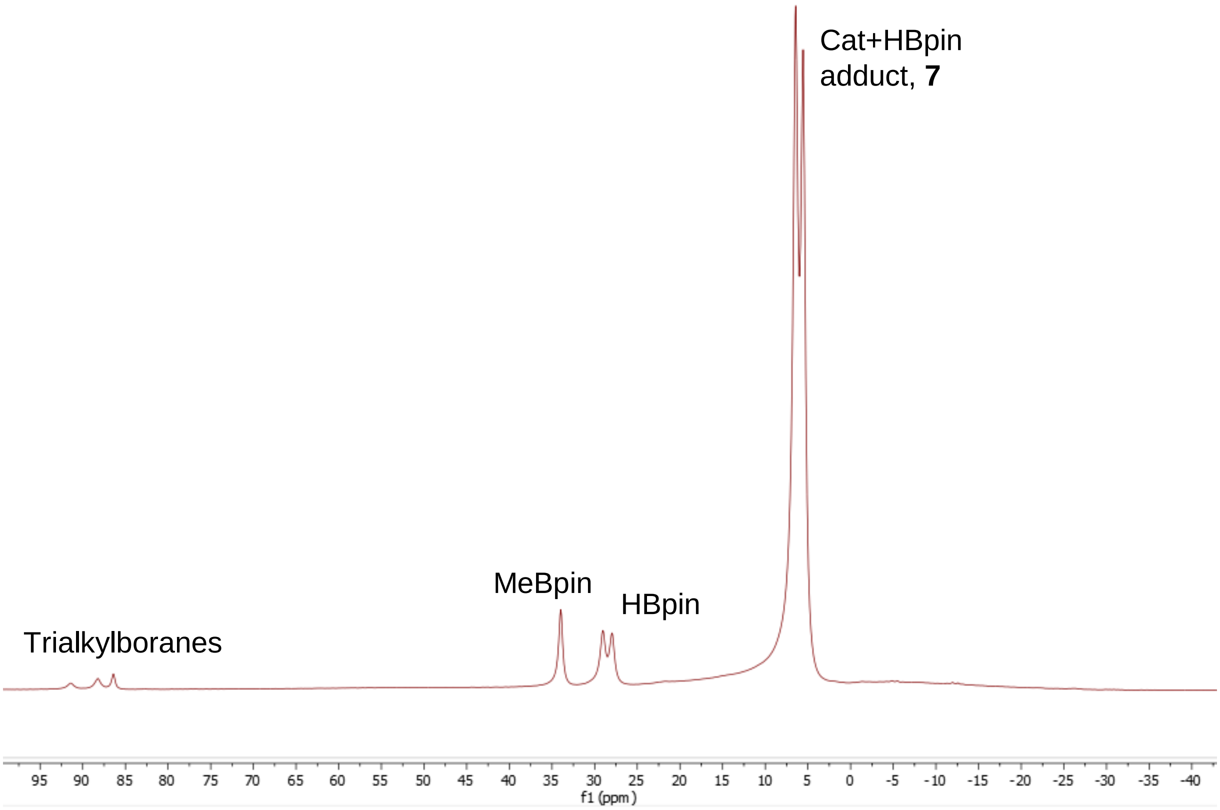

3. Control Reactions

a)

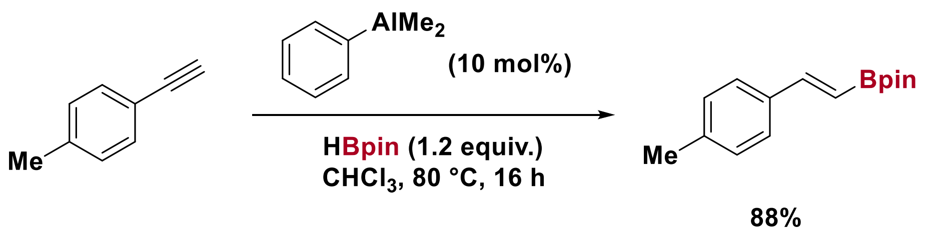

b)

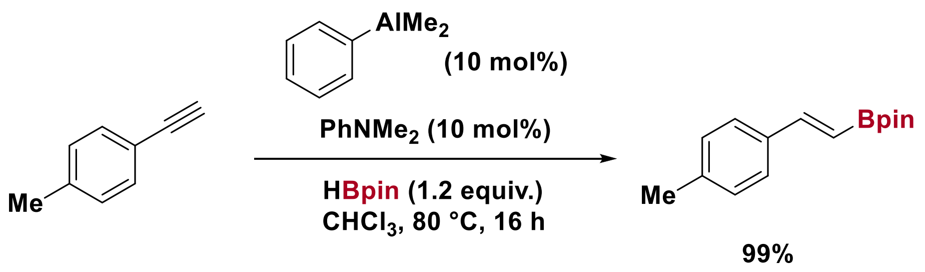

c)

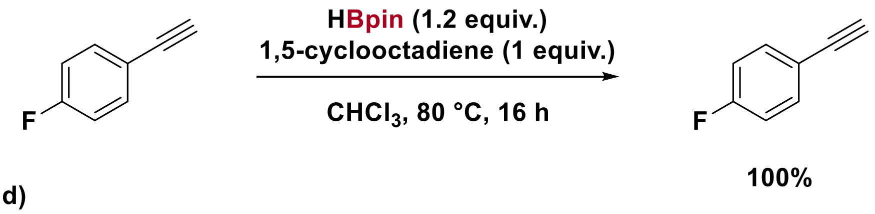

d)

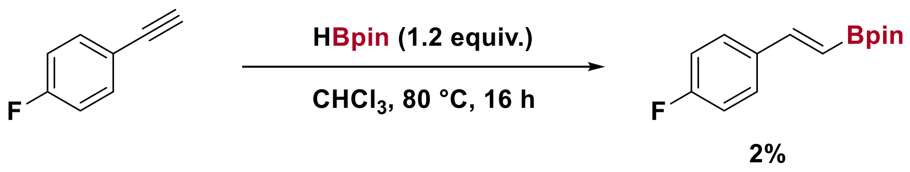

e)

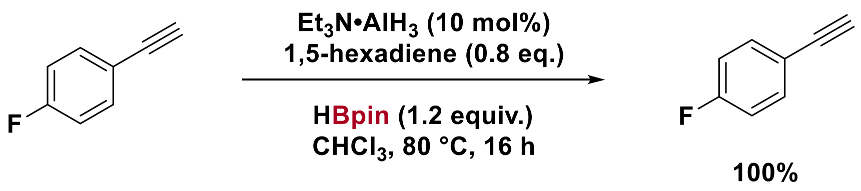

(a) Under an inert atmosphere, $PhAlMe_2$ (0.0500 mmol, 125 µL, 0.4 M in Toluene) and HBpin (0.600 mmol, 87.0 µL) were dissolved in chloroform (0.500 mL). 4-ethynyltoluene (0.500 mmol, 63.0 µL) was added, and the mixture heated at 80 °C for 16 hours. The yield was determined by ^{1}H NMR of the crude reaction mixture, with

1,3,5-trimethoxybenzene as an internal standard. No C–H borylation was observed, giving 88 % hydroboration product.

(b) Under an inert atmosphere, $PhAlMe_2$ (0.0500 mmol, 125 µL, 0.4 M in Toluene), *N,N*-dimethylaniline (0.050, 6.0 µL), and HBpin (0.600 mmol, 87.0 µL) were dissolved in chloroform (0.500 mL). 4-ethynyltoluene (0.500 mmol, 63.0 µL) was added, and the mixture heated at 80 °C for 16 hours. The yield was determined by ^{1}H NMR spectroscopy of the crude reaction mixture, with 1,3,5-trimethoxybenzene as an internal standard. No C–H borylation was observed, giving 99% hydroboration product.

(c) Under an inert atmosphere, HBpin (0.600 mmol, 87.0 µL) and 1,5-cyclooctadiene (0.500 mmol, 61.0 µL) were dissolved in chloroform (0.500 mL). 4'-fluorophenylacetylene (0.500 mmol, 57.0 µL) was added, and the mixture heated at 80 °C for 16 hours. The yield was determined by ^{19}F NMR spectroscopy of the crude reaction mixture, with fluorobenzene as an internal standard. No conversion was observed, 100% starting material was recovered.

(d) Under an inert atmosphere, HBpin (0.600 mmol, 87.0 µL) was dissolved in chloroform (0.500 mL). 4'-fluorophenylacetylene (0.500 mmol, 57.0 µL) was added, and the mixture heated at 80 °C for 16 hours. The yield was determined by ^{19}F NMR spectroscopy of the crude reaction mixture, with fluorobenzene as an internal standard. 2% of the hydroboration product was observed, with no C–H borylation.

(e) Under an inert atmosphere, $Et_3N{\cdot}AlH_3$ (0.0500 mmol, 100 µL, 0.5 M in Toluene), 1,5-hexadiene (0.400 mmol, 48.0 µL) and HBpin (0.600 mmol, 87.0 µL) were dissolved in chloroform (0.500 mL). 4'-fluorophenylacetylene (0.500 mmol, 57.0 µL) was added, and the mixture heated at 80 °C for 16 hours. The yield was determined by ^{19}F NMR spectroscopy of the crude reaction mixture, with fluorobenzene as an internal standard. No conversion of the alkyne was observed, 100% starting material was recovered. $Et_3N{\cdot}BH_3$ (^{11}B: –9.0 ppm; q, J = 98 Hz) was observed by ^{11}B NMR spectroscopy.

4. C–H Borylation of Alkynes Scope

General procedure for the C–H borylation of terminal alkynes

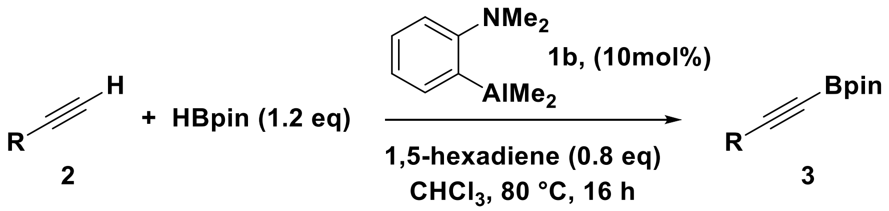

Under an inert atmosphere, 1,5-hexadiene (0.800 mmol, 95.0 µL) followed by the alkyne (1.00 mmol) were added to a solution of 2-dimethylaluminium-*N,N*-dimethylaniline **1b** (18.0 mg, 0.100 mmol) and HBpin (1.20 mmol, 174 µL) in chloroform (1.00 mL) and the mixture heated at 80 °C for 16 hours. Volatiles were removed *in vacuo*. The crude reaction mixture was purified by flash chromatography (SiO_2, 2.30 g, 1:9 Et_2O:pentane, 30.0 mL). NMR yields were calculated by ^{1}H NMR spectroscopy (1,3,5-trimethoxybenzene as internal standard) or ^{19}F NMR spectroscopy (fluorobenzene as internal standard) of the crude reaction mixture. Note that the ^{13}C NMR spectroscopy signal associated with the C–B bond was not always observed.

It should be noted that isolation of alkynyl boronic esters was challenging due to limited stability on silica or alumina for extended periods of time. Although a number of other methods have been reported,[3],[4],[5] in our hands it was found that flash chromatography on silica gel (not dried) over a short plug (see below) was effective for most substrates, ensuring contact time with the silica did not exceed 10 minutes. In certain cases alkynyl boronate ester was isolated as an insepreable mixture with the hydroboration product and MeBpin. These were observed in the corresponding ^{11}B NMR spectra (<5% by ^{1}H NMR spectroscopy).

Purification of alkynyl boronic esters

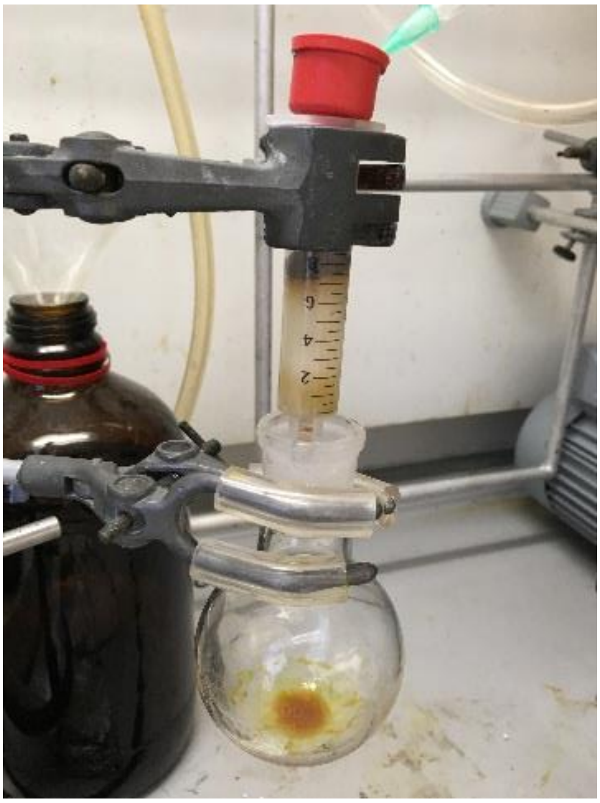

A 10 mL syringe was plugged with cotton wool, then a small layer of sand was used to cover the bottom. Silica (2.30 g) was added, then the plug was flushed through three times with the solvent mixture of choice. The sample was diluted with the solvent mixture (1.00 mL) then loaded onto the silica. Using a septum and needle, the sample was flushed through with solvent under pressure from nitrogen until approximately 30.0 mL of diluted sample was collected. This system was very effective for the removal of HBpin/B_2pin_3/Al_yO_x/*N,N*-dimethylaniline. In cases with >1:9 Et_2O:pentane, co-elution of B_2pin_3 was observed.

4,4,5,5-Tetramethyl-2-(phenylethynyl)-1,3,2-dioxaborolane (3a)

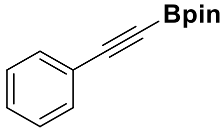

Synthesised and isolated using the general procedure: Under an inert atmosphere, 2-dimethylaluminium-*N,N*-dimethylaniline **1b** (18.0 mg, 0.100 mmol) and HBpin (1.20 mmol, 174 µL) were dissolved in chloroform (1.00 mL). 1,5-Hexadiene (0.800 mmol, 95.0 µL) was added, then phenylacetylene (1.00 mmol, 110 µL), and the mixture heated at 80 °C for 16 hours. Volatiles were removed *in vacuo*. The crude reaction mixture was purified by flash chromatography (SiO_2, 2.30 g, 1:9 Et_2O:pentane, 30.0 mL). The solvent was removed *in vacuo* to give 4,4,5,5-tetramethyl-2-(phenylethynyl)-1,3,2-dioxaborolane as a pale yellow amorphous solid (141 mg, 0.620 mmol, 62%).

^{1}H NMR: (500.12 MHz, C_6D_6)
1.03 (12H, s), 6.82-6.92 (3H, m), 7.37-7.41 (2H, m).

^{11}B NMR: (128.34 MHz, C_6D_6)
24.9 (s br).

^{13}C NMR: (125.77 MHz, C_6D_6)
24.7, 84.1, 101.9, 122.7, 128.5, 129.3, 132.7.

Analytical data were in accordance with those previously reported.[3,5]

4,4,5,5-Tetramethyl-2-(*p*-tolylethynyl)-1,3,2-dioxaborolane (3b)

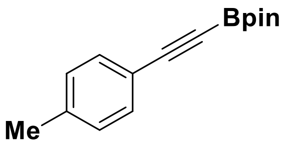

Synthesised and isolated using the general procedure: Under an inert atmosphere, 2-dimethylaluminium-*N,N*-dimethylaniline **1b** (18.0 mg, 0.100 mmol) and HBpin (1.20 mmol, 174 µL) were dissolved in chloroform (1.00 mL). 1,5-Hexadiene (0.800 mmol, 95.0 µL) was added, then 4-ethynyltoluene (1.00 mmol, 127 µL), and the mixture heated at 80 °C for 16 hours. Volatiles were removed *in vacuo*. The crude reaction mixture was purified by flash chromatography (SiO_2, 2.30 g, 1:9 Et_2O:pentane, 30.0 mL). The solvent was removed *in vacuo* to give 4,4,5,5-tetramethyl-2-(p-tolylethynyl)-1,3,2-dioxaborolane as a colourless amorphous solid (169 mg, 0.700 mmol, 70%).

^{1}H NMR: (500 MHz, C_6D_6)
1.04 (12H, s), 1.88 (3H, s), 6.67 (2H, d, *J* = 7.9 Hz), 7.35 (2H, d, *J* = 7.9 Hz).

^{11}B NMR: (128 MHz, C_6D_6)
24.8 (s br).

^{13}C NMR: (126 MHz, C_6D_6)
21.3, 24.7, 84.0, 119.8, 129.4, 132.8, 139.5.

Analytical data were in accordance with those previously reported.[3]

4,4,5,5-Tetramethyl-2-(*m*-tolylethynyl)-1,3,2-dioxaborolane (3c)

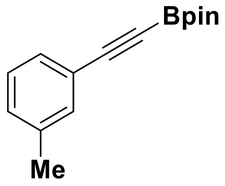

Synthesised and isolated using the general procedure: Under an inert atmosphere, 2-dimethylaluminium-*N,N*-dimethylaniline **1b** (18.0 mg, 0.100 mmol) and HBpin (1.20 mmol, 174 µL) were dissolved in chloroform (1.00 mL). 1,5-Hexadiene (0.800 mmol, 95.0 µL) was added, then 3-ethynyltoluene (1.00 mmol, 129 µL), and the mixture heated at 80 °C for 16 hours. Volatiles were removed *in vacuo*. The crude reaction mixture was purified by flash chromatography (SiO_2, 2.30 g, 1:9 Et_2O:pentane, 30.0 mL). The solvent was removed *in vacuo*, to give 4,4,5,5-tetramethyl-2-(m-tolylethynyl)-1,3,2-dioxaborolane as a pale yellow amorphous solid (170 mg, 0.700 mmol, 70%).

^{1}H NMR: (500 MHz, C_6D_6)
1.04 (12H, s), 1.86 (3H, s), 6.75 (1H, m) 6.82 (1H, t, *J* = 7.9), 7.21 (1H, s), 7.29 (1H, d, *J* = 7.9 Hz).

^{11}B NMR: (128 MHz, C_6D_6)
24.8 (s br).

^{13}C NMR: (126 MHz, C_6D_6)
20.9, 24.7, 84.1, 122.7, 128.5, 129.9, 130.3, 133.5, 138.2.

Analytical data were in accordance with those previously reported.[6]

4,4,5,5-Tetramethyl-2-(*o*-tolylethynyl)-1,3,2-dioxaborolane (3d)

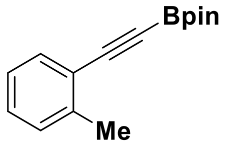

Synthesised and isolated using the general procedure: Under an inert atmosphere, 2-dimethylaluminium-*N,N*-dimethylaniline **1b** (18.0 mg, 0.100 mmol) and HBpin (1.20 mmol, 174 µL) were dissolved in chloroform (1.00 mL). 1,5-Hexadiene (0.800 mmol, 95.0 µL) was added, then 2-ethynyltoluene (1.00 mmol, 126 µL), and the mixture heated at 80 °C for 16 hours. Volatiles were removed *in vacuo*. The crude reaction mixture was purified by flash chromatography (SiO_2, 2.30 g, 1:9 Et_2O:pentane, 30.0 mL). The solvent was removed *in vacuo* to give 4,4,5,5-tetramethyl-2-(*o*-tolylethynyl)-1,3,2-dioxaborolane as a pale yellow oil (119 mg, 0.490 mmol, 49%).

^{1}H NMR: (500 MHz, C_6D_6)
1.04 (12H, s), 2.35 (3H, s), 6.78 (1H, t, *J* = 7.8 Hz) 6.83 (1H, m), 6.88-6.92 (1H, m), 7.44 (1H, dd, *J* = 7.8 Hz, *J* = 1.3 Hz).

^{11}B NMR: (128 MHz, C_6D_6)
25.0 (s br).

^{13}C NMR: (126 MHz, C_6D_6)
20.7, 24.7, 84.1, 122.5, 125.9, 129.4, 129.8, 133.3, 141.6.

Analytical data were in accordance with those previously reported.[6]

2-[(4-(*tert*-Butyl)phenyl)ethynyl]-4,4,5,5-tetramethyl-1,3,2-dioxaborolane (3e)

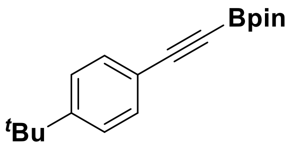

Synthesised and isolated using the general procedure: Under an inert atmosphere, 2-dimethylaluminium-*N,N*-dimethylaniline **1b** (18.0 mg, 0.100 mmol) and HBpin (1.20 mmol, 174 µL) were dissolved in chloroform (1.00 mL). 1,5-Hexadiene (0.800 mmol, 95.0 µL) was added, then 4-ethynyl(*tert*-butyl)benzene (1.00 mmol, 180 µL), and the mixture heated at 80 °C for 16 hours. Volatiles were removed *in vacuo*. The crude reaction mixture was purified by flash chromatography (SiO_2, 2.30 g, 1:9 Et_2O:pentane, 30.0 mL). The solvent was removed *in vacuo*, to give 2-[(4-(*tert*-butyl)phenyl)ethynyl]-4,4,5,5-tetramethyl-1,3,2-dioxaborolane as a pale yellow amorphous solid (176 mg, 0.620 mmol, 62%).

^{1}H NMR: (500.12 MHz, C_6D_6)
1.03 (12H, s), 1.04 (9H, s), 6.98 (2H, dt, *J* = 8.7 Hz, *J* = 2.0 Hz), 7.45 (2H, dt, *J* = 8.7 Hz, *J* = 2.0 Hz).

^{11}B NMR: (128.34 MHz, C_6D_6)
25.0 (s br).

^{13}C NMR: (125.77 MHz, C_6D_6)
24.7, 31.0, 34.7, 84.0, 119.9, 125.7, 132.7, 152.5.

Analytical data were in accordance with those previously reported.[3]

2-[(4-Methoxyphenyl)ethynyl]-4,4,5,5-tetramethyl-1,3,2-dioxaborolane (3f)

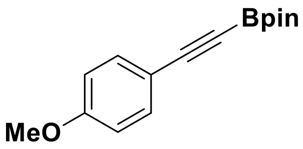

Synthesised and isolated using the general procedure: Under an inert atmosphere, 2-dimethylaluminium-*N,N*-dimethylaniline **1b** (18.0 mg, 0.100 mmol) and HBpin (1.20 mmol, 174 µL) were dissolved in chloroform (1.00 mL). 1,5-Hexadiene (0.800 mmol, 95.0 µL) was added, then 4-ethynylanisole (1.00 mmol, 130 µL), and the mixture heated at 80 °C for 16 hours. Volatiles were removed *in vacuo*. The crude reaction mixture was purified by flash chromatography (SiO_2, 2.30 g, 1:1 Et_2O:pentane, 30.0 mL). The solvent was removed *in vacuo*, to give 2-[(4-methoxyphenyl)ethynyl]-4,4,5,5-tetramethyl-1,3,2-dioxaborolane as a colourless oil (155 mg, 0.600 mmol, 60%).

^{1}H NMR: (500.12 MHz, C_6D_6)
1.05 (12H, s), 3.10 (3H, s), 6.44 (2H, d, J = 8.2 Hz), 7.35 (2H, d, J = 9.4 Hz).

^{11}B NMR: (128.34 MHz, C_6D_6)
25.0 (s br).

^{13}C NMR: (125.77 MHz, C_6D_6)
24.7, 54.7, 84.0, 114.3, 114.8, 134.5, 160.8.

Analytical data were in accordance with those previously reported.[3]

4,4,5,5-Tetramethyl-2-(*p*-*N,N*-dimethylaniline)-1,3,2-dioxaborolane (3g)

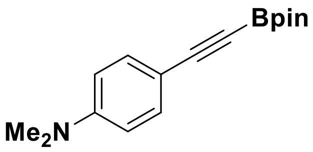

Synthesised and isolated using the general procedure: Under an inert atmosphere, 2-dimethylaluminium-*N,N*-dimethylaniline **1b** (18.0 mg, 0.100 mmol) and HBpin (1.20 mmol, 174 µL) were dissolved in chloroform (1.00 mL). 1,5-Hexadiene (0.800 mmol, 95.0 µL) was added, then 4-ethynyl-*N,N*-dimethylaniline (1.00 mmol, 145 mg), and the mixture heated at 80 °C for 16 hours. Volatiles were removed *in vacuo*. The crude reaction mixture was purified by flash chromatography (SiO_2, 2.30 g, Et_2O, 30.0 mL). The solvent was removed *in vacuo*, to give 4,4,5,5-tetramethyl-2-(*p*-*N,N*-dimethylaniline)-1,3,2-dioxaborolane as an orange oil (193 mg, 0.710 mmol, 71%).

^{1}H NMR: (500 MHz, C_6D_6)
1.06 (12H, s), 2.28 (6H, s), 6.19 (2H, d, *J* = 8.9 Hz), 7.48 (2H, d, *J* = 8.9 Hz).

^{11}B NMR: (128 MHz, C_6D_6)
25.3 (s br).

^{13}C NMR: (126 MHz, C_6D_6)
24.8, 39.5, 83.7, 109.6, 112.0, 134.3, 150.9.

Analytical data were in accordance with those previously reported.[7]

1,4-Bis[(4,4,5,5-tetramethyl-1,3,2-dioxaborolan-2-yl)ethynyl]benzene (3h)

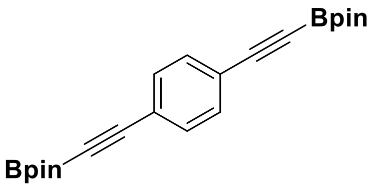

Synthesised and isolated using the general procedure: Under an inert atmosphere, 2-dimethylaluminium-*N,N*-dimethylaniline **1b** (18.0 mg, 0.100 mmol) and HBpin (1.20 mmol, 174 µL) were dissolved in chloroform (1.00 mL). 1,5-Hexadiene (0.800 mmol, 95.0 µL) was added, then 1,4-bis(ethynyl)benzene (0.500 mmol, 63.0 mg), and the mixture heated at 80 °C for 16 hours. Volatiles were removed *in vacuo*. The crude reaction mixture was purified by flash chromatography (SiO_2, 2.30 g, 1:1 Et_2O:pentane, 30.0 mL), to give a yellow solid. This was then washed with pentane (2.00 mL) and the solvent was removed *in vacuo*, to give 1,4-bis[(4,4,5,5-tetramethyl-1,3,2-dioxaborolan-2-yl)ethynyl]benzene as a colourless amorphous solid (48 mg, 0.125 mmol, 25%).

^{1}H NMR: (500 MHz, C_6D_6)
1.01 (24H, s), 7.06 (4H, s).

^{11}B NMR: (128 MHz, C_6D_6)
24.9 (s br).

^{13}C NMR: (126 MHz, C_6D_6)
24.7, 84.2, 101.1, 123.3, 132.6.

Analytical data were in accordance with those previously reported.[3]

4,4,5,5-Tetramethyl-2-((4-(trifluoromethyl)phenyl)ethynyl)-1,3,2-dioxaborolane (3i)

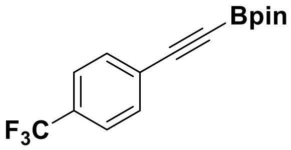

Synthesised using 20 mol% of **1b**, and isolated using the general procedure: Under an inert atmosphere, 2-dimethylaluminium-*N,N*-dimethylaniline **1b** (36.0 mg, 0.200 mmol) and HBpin (1.20 mmol, 174 µL) were dissolved in chloroform (1.00 mL). 1,5-Hexadiene (0.800 mmol, 95.0 µL) was added, then 4-ethynyl-(trifluoromethyl)benzene (1.00 mmol, 153 µL), and the mixture heated at 80 °C for 16 hours. The crude reaction mixture was purified by flash chromatography (SiO_2, 2.30 g, 1:9 Et_2O:pentane, 30.0 mL). The solvent was removed *in vacuo*, to give 4,4,5,5-tetramethyl-2-((4-(trifluoromethyl)phenyl)ethynyl)-1,3,2-dioxaborolane as a pale yellow amorphous solid (112 mg, 0.360 mmol, 36%)

^{1}H NMR: (500.12 MHz, C_6D_6)
1.03 (12H, s), 6.98 (2H, d, *J* = 8.0 Hz), 7.12 (2H, d, *J* = 8.0 Hz).

^{11}B NMR: (128.34 MHz, C_6D_6)
24.9 (s br).

^{13}C NMR: (125.77 MHz, C_6D_6)
24.7, 84.4, 125.4 (q, *J* = 3.8 Hz), 132.8.

^{19}F NMR: (470.39 MHz, C_6D_6)
–62.8 (s).

Analytical data were in accordance with those previously reported.[3]

4,4,5,5-Tetramethyl-2-((3-chlorophenyl)ethynyl)-1,3,2-dioxaborolane (3j)

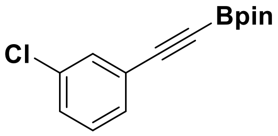

Synthesised and isolated using the general procedure: Under an inert atmosphere, 2-dimethylaluminium-*N,N*-dimethylaniline **1b** (18.0 mg, 0.100 mmol) and HBpin (1.20 mmol, 174 µL) were dissolved in chloroform (1.00 mL). 1,5-Hexadiene (0.800 mmol, 95.0 µL) was added, then 3-ethynylchlorobenzene (1.00 mmol, 123 µL), and the mixture heated at 80 °C for 16 hours. Volatiles were removed *in vacuo*. The crude reaction mixture was purified by flash chromatography (SiO_2, 2.30 g, 1:9 Et_2O:pentane, 30.0 mL). The solvent was removed *in vacuo*, to give *4,4,5,5-tetramethyl-2-((3-chlorophenyl)ethynyl)-1,3,2-dioxaborolane* as a pale yellow oil (152 mg, 0.580 mmol, 58%).

^{1}H NMR: (500.12 MHz, C_6D_6)
1.03 (12H, s), 6.52 (1H, t, *J* = 7.9 Hz), 6.85 (1H, d(1:1:1:1)q, *J* = 8.2 Hz, *J* = 1.1 Hz), 7.05 (1H, d, *J* = 7.8 Hz), 7.29 (1H, m).

^{11}B NMR: (128.34 MHz, C_6D_6)
24.7 (s br).

^{13}C NMR: (125.77 MHz, C_6D_6)
24.7, 84.3, 100.1 (br), 124.3, 129.6, 129.7, 130.6, 132.5, 134.5.

MS: (HRMS – ESI$^+$)
Found: 252.09291 ($C_{14}H_{16}O_2{}^{11}B_1{}^{35}Cl_1$), requires: 262.09264.

2-((4-Fluorophenyl)ethynyl)-4,4,5,5-tetramethyl-1,3,2-dioxaborolane (3k)

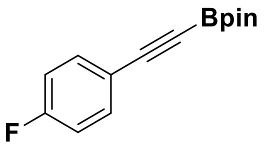

Synthesised and isolated using the general procedure: Under an inert atmosphere, 2-dimethylaluminium-*N,N*-dimethylaniline **1b** (18.0 mg, 0.100 mmol) and HBpin (1.20 mmol, 174 µL) were dissolved in chloroform (1.00 mL). 1,5-Hexadiene (0.800 mmol, 95.0 µL) was added, then 4'-fluorophenylacetylene (1.00 mmol, 115 µL), and the mixture heated at 80 °C for 16 hours. Volatiles were removed *in vacuo*. The crude reaction mixture was purified by flash chromatography (SiO_2, 2.30 g, 1:9 Et_2O:pentane, 30.0 mL). The solvent was removed *in vacuo*, to give 2-((4-fluorophenyl)ethynyl)-4,4,5,5-tetramethyl-1,3,2-dioxaborolane as colourless block crystals (159 mg, 0.650 mmol, 65%).

^{1}H NMR: (500.12 MHz, C_6D_6)
1.03 (12H, s), 6.44 (2H, t, *J* = 8.8 Hz), 7.12 (2H, m).

^{11}B NMR: (128.34 MHz, C_6D_6)
24.8 (s br).

^{13}C NMR: (125.77 MHz, C_6D_6)
24.3, 83.8, 115.4 (d, *J* = 22.4 Hz), 134.4 (d, *J* = 8.7 Hz) 163.0 (d, *J* = 250.5 Hz).

^{19}F NMR: (470.39 MHz, C_6D_6)
–109.18 (m).

Analytical data were in accordance with those previously reported.[6]

4,4,5,5-Tetramethyl-2-(2-ethynylthiophene)-1,3,2-dioxaborolane (3l)

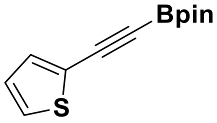

Synthesised and isolated using the general procedure: Under an inert atmosphere, 2-dimethylaluminium-*N,N*-dimethylaniline **1b** (18.0 mg, 0.100 mmol) and HBpin (1.20 mmol, 174 µL) were dissolved in chloroform (1.00 mL). 1,5-Hexadiene (0.800 mmol, 95.0 µL) was added, then 2-ethynylthiophene (1.00 mmol, 95.0 µL), and the mixture heated at 80 °C for 16 hours. Volatiles were removed *in vacuo*. The crude reaction mixture was purified by flash chromatography (SiO_2, 2.30 g, 1:1 Et_2O:pentane, 30.0 mL). The solvent was removed *in vacuo*, to give 4,4,5,5-tetramethyl-2-(3-phenyletherprop-1-yn-1-yl)-1,3,2-dioxaborolane as a pale yellow oil (163 mg, 0.520 mmol, 52%).

^{1}H NMR: (500.12 MHz, C_6D_6)
1.01 (12H, s), 6.38 (1H, dd, *J* = 5.1 Hz, *J* = 3.7 Hz), 6.57 (1H, dd, *J* = 5.2 Hz, *J* = 1.2 Hz), 7.02 (1H, dd, *J* = 3.6 Hz, *J* = 1.1 Hz).

^{11}B NMR: (128.34 MHz, C_6D_6)
24.2 (s br).

^{13}C NMR: (125.77 MHz, C_6D_6)
24.2, 55.7, 83.8, 122.7, 127.2, 128.8, 134.5.

Analytical data were in accordance with those previously reported.[6]

4,4,5,5-Tetramethyl-2-(1-dodec-1-yn-1-yl)-1,3,2-dioxaborolane (3m)

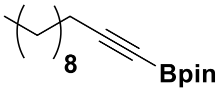

Synthesised and isolated using the general procedure: Under an inert atmosphere, 2-dimethylaluminium-*N,N*-dimethylaniline **1b** (18.0 mg, 0.100 mmol) and HBpin (1.20 mmol, 174 µL) were dissolved in chloroform (1.00 mL). 1,5-Hexadiene (0.800 mmol, 95.0 µL) was added, then 1-dodecyne (1.00 mmol, 214 µL), and the mixture heated at 80 °C for 16 hours. Volatiles were removed *in vacuo*. The crude reaction mixture was purified by flash chromatography (SiO_2, 2.30 g, 1:9 Et_2O:pentane, 30.0 mL). The solvent was removed *in vacuo*, to give 4,4,5,5-tetramethyl-2-(1-dodec-1-yn-1-yl)-1,3,2-dioxaborolane as a colourless oil (193 mg, 0.660 mmol, 66%).

^{1}H NMR: (500.12 MHz, C_6D_6)
0.90 (3H, t, J = 7.2 Hz), 1.01 (12H, s), 1.06-1.39 (16H, m), 2.01 (2H, t, J = 7.1).

^{11}B NMR: (128.34 MHz, C_6D_6)
24.1(s br).

^{13}C NMR: (125.77 MHz, C_6D_6)
14.4, 19.8, 23.1, 24.7, 28.5, 29.1, 29.5, 29.7, 29.8, 30.0, 32.3, 83.6.

Analytical data were in accordance with those previously reported.[3]

4,4,5,5-Tetramethyl-2-(3-phenylprop-1-yn-1-yl)-1,3,2-dioxaborolane (3n)

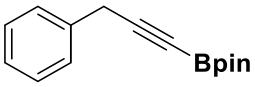

Synthesised and isolated using the general procedure: Under an inert atmosphere, 2-dimethylaluminium-*N,N*-dimethylaniline **1b** (18.0 mg, 0.100 mmol) and HBpin (1.20 mmol, 174 µL) were dissolved in chloroform (1.00 mL). 1,5-Hexadiene (0.800 mmol, 95.0 µL) was added, then 3-phenyl-1-propyne (1.00 mmol, 124 µL), and the mixture heated at 80 °C for 16 hours. Volatiles were removed *in vacuo*. The crude reaction mixture was purified by flash chromatography (SiO_2, 2.30 g, 1:9 Et_2O:pentane, 30.0 mL). The solvent was removed *in vacuo*, to give 4,4,5,5-tetramethyl-2-(3-phenylprop-1-yn-1-yl)-1,3,2-dioxaborolane as a colourless oil (159 mg, 0.660 mmol, 66%).

^{1}H NMR: (500.12 MHz, C_6D_6)
1.01 (12H, s), 3.29 (2H, s), 6.95-7.05 (3H, m), 7.14-7.17 (2H, m).

^{11}B NMR: (128.34 MHz, C_6D_6)
25.0 (s br).

^{13}C NMR: (125.77 MHz, C_6D_6)
24.7, 25.9, 83.8, 101.6 (br), 126.9, 128.3, 128.7, 135.9.

Analytical data were in accordance with those previously reported.[3]

4,4,5,5-Tetramethyl-2-(1-cyclohex-1-enyl)-1,3,2-dioxaborolane (3o)

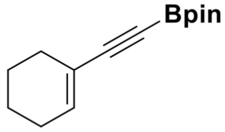

Synthesised and isolated using the general procedure: Under an inert atmosphere, 2-dimethylaluminium-*N,N*-dimethylaniline **1b** (18.0 mg, 0.100 mmol) and HBpin (1.20 mmol, 174 µL) were dissolved in chloroform (1.00 mL). 1,5-Hexadiene (0.800 mmol, 95.0 µL) was added, then 1-ethynylcyclohex-2-ene (1.00 mmol, 118 µL), and the mixture heated at 80 °C for 16 hours. Volatiles were removed *in vacuo*. The crude reaction mixture was purified by flash chromatography (SiO_2, 2.30 g, 1:9 Et_2O:pentane, 30.0 mL). The solvent was removed *in vacuo*, to give 4,4,5,5-tetramethyl-2-(1-cyclohex-1-enyl)-1,3,2-dioxaborolane as a colourless amorphous solid (94 mg, 0.400 mmol, 40%).

^{1}H NMR: (500.12 MHz, C_6D_6)
1.01 (12H, s), 1.18 (2H, m), 1.25 (2H, m), 1.67 (2H, m), 2.05 (2H, m), 6.18 (1H, sept).

^{11}B NMR: (128.34 MHz, C_6D_6)
24.9 (s br).

^{13}C NMR: (125.77 MHz, C_6D_6)
21.5, 22.2, 24.7, 25.8, 28.9, 83.8, 120.9, 138.1.

Analytical data were in accordance with those previously reported.[6]

4,4,5,5-Tetramethyl-2-(3,3-dimethylbut-1-yn-1-yl)-1,3,2-dioxaborolane (3p)

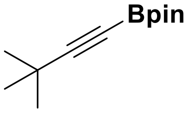

Synthesised and isolated using the general procedure: Under an inert atmosphere, 2-dimethylaluminium-*N,N*-dimethylaniline **1b** (18.0 mg, 0.100 mmol) and HBpin (1.20 mmol, 174 µL) were dissolved in chloroform (1.00 mL). 1,5-Hexadiene (0.800 mmol, 95.0 µL) was added, then 2,2-dimethylbut-1-yne (1.00 mmol, 123 µL), and the mixture heated at 80 °C for 16 hours. Volatiles were removed *in vacuo*. The crude reaction mixture was purified by flash chromatography (SiO_2, 2.30 g, 1:9 Et_2O:pentane, 30.0 mL). The solvent was removed *in vacuo*, to give 4,4,5,5-tetramethyl-2-(3,3-dimethylbut-1-yn-1-yl)-1,3,2-dioxaborolane as a colourless amorphous solid (23 mg, 0.110 mmol, 11%).

^{1}H NMR: (500.12 MHz, C_6D_6)
0.99 (12H, s), 1.09 (9H, s).

^{11}B NMR: (128.34 MHz, C_6D_6)
24.5 (s br).

^{13}C NMR: (125.77 MHz, C_6D_6)
24.7, 28.1, 30.6, 83.6.

Analytical data were in accordance with those previously reported.[3]

4,4,5,5-Tetramethyl-2-(2-cyclopropyleth-1-yn-1-yl)-1,3,2-dioxaborolane (3q)

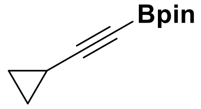

Synthesised and isolated using the general procedure: Under an inert atmosphere, 2-dimethylaluminium-*N,N*-dimethylaniline **1b** (18.0 mg, 0.100 mmol) and HBpin (1.20 mmol, 174 µL) were dissolved in chloroform (1.00 mL). 1,5-Hexadiene (0.800 mmol, 95.0 µL) was added, then 1-ethynylcyclopropane (1.00 mmol, 85.0 µL), and the mixture heated at 80 °C for 16 hours. Volatiles were removed *in vacuo*. The crude reaction mixture was purified by flash chromatography (SiO_2, 2.30 g, 1:9 Et_2O:pentane, 30.0 mL). The solvent was removed *in vacuo*, to give 4,4,5,5-tetramethyl-2-(2-cyclopropyleth-1-yn-1-yl)-1,3,2-dioxaborolane as a colourless microcrystalline solid (102 mg, 0.530 mmol, 53%).

^{1}H NMR: (500.12 MHz, C_6D_6)
0.24 (2H, m), 0.54 (2H, m) 0.92 (1H, m), 1.00 (12H, s).

^{11}B NMR: (128.34 MHz, C_6D_6)
24.2 (s br).

^{13}C NMR: (125.77 MHz, C_6D_6)
0.5, 8.7, 24.7, 83.6.

Analytical data were in accordance with those previously reported.[3]

4,4,5,5-Tetramethyl-2-(5-chloropent-1-yn-1-yl)-1,3,2-dioxaborolane (3r)

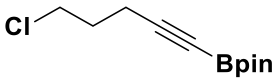

Synthesised and isolated using the general procedure: Under an inert atmosphere, 2-dimethylaluminium-*N,N*-dimethylaniline **1b** (18.0 mg, 0.100 mmol) and HBpin (1.20 mmol, 174 µL) were dissolved in chloroform (1.00 mL). 1,5-Hexadiene (0.800 mmol, 95.0 µL) was added, then 5-chloropent-1-yne (1.00 mmol, 108 µL), and the mixture heated at 80 °C for 16 hours. Volatiles were removed *in vacuo*. The crude reaction mixture was purified by flash chromatography (SiO_2, 2.30 g, 1:9 Et_2O:pentane, 30.0 mL). The solvent was removed *in vacuo*, to give 4,4,5,5-tetramethyl-2-(5-chloropent-1-yn-1-yl)-1,3,2-dioxaborolane as a colourless oil (154 mg, 0.680 mmol, 68%).

^{1}H NMR: (500.12 MHz, C_6D_6)
1.00 (12H, s), 1.42 (2H, quin, *J* = 6.8 Hz), 1.99 (2H, t, *J* = 7.0 Hz), 3.09 (2H, t, *J* = 6.4 Hz).

^{11}B NMR: (128.34 MHz, C_6D_6)
24.2 (s br).

^{13}C NMR: (125.77 MHz, C_6D_6)
16.9, 24.7, 31.0, 43.3, 83.8, 102.3 (br s).

Analytical data were in accordance with those previously reported.[5]

4,4,5,5-Tetramethyl-2-(trimethylsilylethynyl)-1,3,2-dioxaborolane (3s)

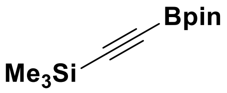

Synthesised and isolated using the general procedure: Under an inert atmosphere, 2-dimethylaluminium-*N,N*-dimethylaniline **1b** (18.0 mg, 0.100 mmol) and HBpin (1.20 mmol, 174 µL) were dissolved in chloroform (1.00 mL). 1,5-Hexadiene (0.800 mmol, 95.0 µL) was added, then 2-trimethylsilylethyne (1.00 mmol, 139 µL), and the mixture heated at 80 °C for 16 hours. Volatiles were removed *in vacuo*. The crude reaction mixture was purified by flash chromatography (SiO_2, 2.30 g, 1:9 Et_2O:pentane, 30.0 mL). The solvent was removed *in vacuo*, to give 4,4,5,5-tetramethyl-2-(5-chloropent-1-yn-1-yl)-1,3,2-dioxaborolane as a colourless amorphous solid (69 mg, 0.310 mmol, 31%).

^{1}H NMR: (500.12 MHz, C_6D_6)
0.07 (9H, s), 0.96 (12H, s).
^{11}B NMR: (128.34 MHz, C_6D_6)
23.6 (s br).
^{13}C NMR: (125.77 MHz, C_6D_6)
–0.5, 24.6, 84.0, 110.7 (br s).
^{29}Si NMR: (99.33 MHz, C_6D_6)
–17.9.

Analytical data were in accordance with those previously reported.[3]

4,4,5,5-Tetramethyl-2-(3-phenylsulfideprop-1-yn-1-yl)-1,3,2-dioxaborolane (3t)

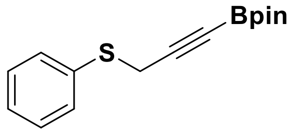

Synthesised and isolated using the general procedure: Under an inert atmosphere, 2-dimethylaluminium-*N,N*-dimethylaniline **1b** (0.200 mmol, 36.0 mg) and HBpin (1.20 mmol, 174 µL) were dissolved in chloroform (1.00 mL). 1,5-Hexadiene (0.800 mmol, 95.0 µL) was added, then 3-phenylsulfideprop-1-yne (1.00 mmol, 139 µL), and the mixture heated at 80 °C for 16 hours. Volatiles were removed *in vacuo*. The crude reaction mixture was purified by flash chromatography (SiO_2, 2.30 g, 1:1 Et_2O:pentane, 30.0 mL). The solvent was removed *in vacuo*, to give 4,4,5,5-tetramethyl-2-(3-phenylsulfideprop-1-yn-1-yl)-1,3,2-dioxaborolane as a pale yellow oil (160 mg, 0.580 mmol, 58%).

^{1}H NMR: (500.12 MHz, C_6D_6)
0.95 (12H, s), 3.16 (2H, s), 6.85-7.05 (3H, m), 7.28-7.32 (2H, m).

^{11}B NMR: (128.34 MHz, C_6D_6)
24.2 (s br).

^{13}C NMR: (125.77 MHz, C_6D_6)
23.2, 24.6, 84.0, 126.9, 129.2, 130.3.

Analytical data were in accordance with those previously reported.[8]

4,4,5,5-Tetramethyl-2-(3-phenyletherprop-1-yn-1-yl)-1,3,2-dioxaborolane (3u)

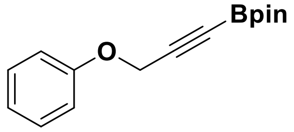

Synthesised and isolated using the general procedure: Under an inert atmosphere, 2-dimethylaluminium-*N,N*-dimethylaniline **1b** (0.200 mmol, 36.0 mg) and HBpin (1.20 mmol, 174 µL) were dissolved in chloroform (1.00 mL). 1,5-Hexadiene (0.800 mmol, 95.0 µL) was added, then 3-phenyletherprop-1-yne (1.00 mmol, 128 µL), and the mixture heated at 80 °C for 16 hours. Volatiles were removed *in vacuo*. The crude reaction mixture was purified by flash chromatography (SiO_2, 2.30 g, 1:9 Et_2O:pentane, 30.0 mL). The solvent was removed *in vacuo*, to give *4,4,5,5-tetramethyl-2-(3-phenyletherprop-1-yn-1-yl)-1,3,2-dioxaborolane* as a colourless oil (163 mg, 0.630 mmol, 63%).

^{1}H NMR: (500.12 MHz, $CDCl_3$)
1.27 (12H, s), 4.72 (2H, s), 6.94-7.01 (3H, m), 7.26-7.32 (2H, m).

^{11}B NMR: (128.34 MHz, $CDCl_3$)
23.8 (s br).

^{13}C NMR: (125.77 MHz, $CDCl_3$)
24.8, 56.2, 84.7, 115.0, 121.7, 129.6, 157.8.

MS: (HRMS – ESI^+)
Found: 258.14134 ($C_{15}H_{19}O_3{}^{11}B_1$), requires: 258.14218.

5. Reactions of Alkynyl Boronic Esters

Preparation of 3,6-bis(2-pyridyl)-4-tolyl-5-(4,4,5,5-tetramethyl[1,3,2]dioxaborolan-2-yl)pyridazine (4)

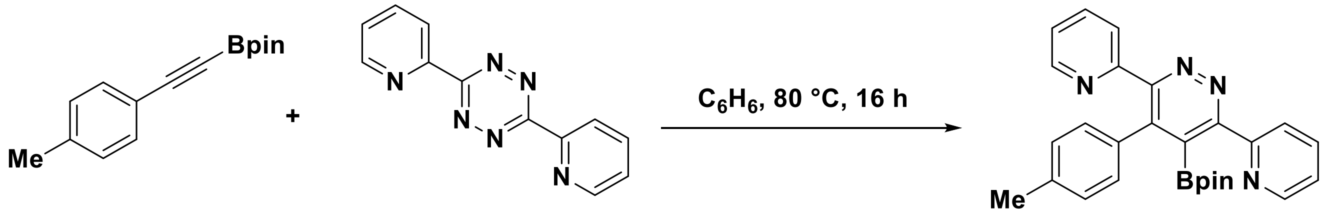

4,4,5,5-Tetramethyl-2-(*p*-tolylethynyl)-1,3,2-dioxaborolane (0.500 mmol, 121 mg) and 1,4-bis(2-pyridyl)-2,3,5,6-tetrazine (0.500 mmol, 236 mg) were dissolved in benzene (1.00 mL) and stirred at 80 °C for 16 hours. Upon completion, the reaction turned from purple to pale yellow. The crude reaction mixture was purified by flash chromatography (SiO_2, R_f = 0.4; 7% MeOH in CH_2Cl_2 as the eluent). The solvent was removed *in vacuo* to give the *pyridazine* as a pale yellow amourphous solid (108 mg, 0.240 mmol, 48%).

^{1}H NMR: (500.12 MHz, C_6D_6)
1.10 (12H, s), 2.07 (3H, s), 6.46 (1H, ddd, *J* = 7.7 Hz, *J* = 4.8 Hz, *J* = 1.1 Hz), 6.60 (1H, ddd, *J* = 7.5 Hz, *J* = 5.1 Hz, *J* = 1.1 Hz), 6.93 (2H, d, *J* = 7.9 Hz), 7.05 (2H, m), 7.34 (2H, d, *J* = 8.0 Hz), 7.73 (1H, d, *J* = 7.8 Hz), 8.22 (1H, d, *J* = 4.9 Hz), 8.39 (1H, *J* = 4.9 Hz), 8.72 (1H, d, *J* = 8.0 Hz).

^{11}B NMR: (128.34 MHz, C_6D_6)
26.1 (s br).

^{13}C NMR: (125.77 MHz, C_6D_6)
21.2, 22.0, 26.5, 74.6, 83.1, 121.5, 122.4, 124.7, 125.2, 128.2, 130.9, 135.6, 137.0, 138.3, 145.0, 145.8, 148.7, 154.3, 157.7, 159.4.

M.P: 182 – 183 °C (MeOH)

I.R: (*vmax (neat)* / cm^{-1})
1377 (C–N), 1142 (C–O).

MS: (HRMS – ESI$^+$)
Found: 450.22188 ($C_{27}H_{27}O_2N_4{}^{11}B_1$), requires: 450.22216.

Preparation of 2,2'-(2-(*p*-tolyl)ethene-1,1-diyl)bis(4,4,5,5-tetramethyl-1,3,2-dioxaborolane) (5)

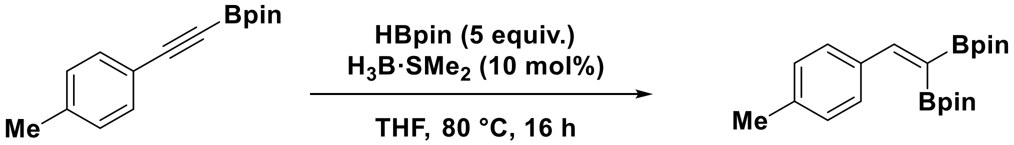

4,4,5,5-Tetramethyl-2-(*p*-tolylethynyl)-1,3,2-dioxaborolane (0.50 mmol, 121 mg) and HBpin (2.50 mmol, 348 µL) were dissolved in THF (1.00 mL). $H_3B{\cdot}SMe_2$ (0.05 mmol, 5 µL) was added, then the reaction was left stirring at 80 °C for 16 hours. Volatiles were removed *in vacuo*. The crude reaction mixture was purified by flash chromatography (SiO_2, 2.30 g, 1:9 Et_2O:pentane, 30.0 mL). All solvent was removed *in vacuo* giving 2,2'-(2-(*p*-tolyl)ethene-1,1-diyl)bis(4,4,5,5-tetramethyl-1,3,2-dioxaborolane) as a colourless oil (110 mg, 60 %).

^{1}H NMR: (500.12 MHz, $CDCl_3$)
1.27 (12H, s), 1.32 (12H, s), 2.33 (3H, s), 7.10 (2H, d, *J* = 8.0 Hz), 7.39 (2H, d, *J* = 8.0 Hz), 7.67 (1H, s).

^{11}B NMR: (128.34 MHz, $CDCl_3$)
31.2, 32.5 (overlapping with each other).

^{13}C NMR: (125.77 MHz, $CDCl_3$)
21.5, 24.8, 25.0, 83.3, 83.7, 128.4, 129.0, 137.0, 138.6, 155.3.

Analytical data were in accordance with those previously reported.[6]

6. Synthesis of Aluminium Compounds

Preparation of 2-lithio-*N,N*-dimethylaniline

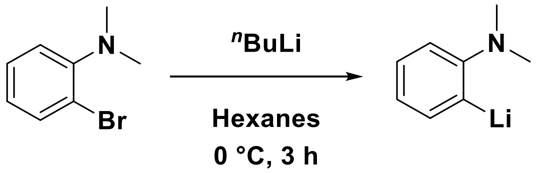

By the method of Chernichenko *et al*,[9] 2-bromo-*N,N*-dimethylaniline (25.0 mmol, 3.62 mL) was added to hexane (30.0 mL) and the resultant solution stirred and cooled to 0 °C. *n*-Butyllithium solution in hexanes (25.0 mmol, 10.0 mL, 2.5 M) was added dropwise and the solution stirred for another 3 hours. The solution was filtered leaving an off white solid which was washed with hexanes (3 × 5.0 mL). All solvent was removed *in vacuo* to give the product as an off-white amorphous solid (2.48 g, 19.5 mmol, 78%).

^{1}H NMR: (500.12 MHz, C_6D_6)
2.08 (6H, s br), 7.01-7.05 (1H, m), 7.24-7.28 (2H, m), 8.27 (1H, s br).

^{13}C NMR: (125.77 MHz, C_6D_6)
46.9, 119.0, 126.2, 128.0, 140.3, 166.2, 168.9 (br).

Analytical data were in accordance with those previously reported.[9]

Preparation of 2-dimethylaluminium-*N,N*-dimethylaniline (1b)

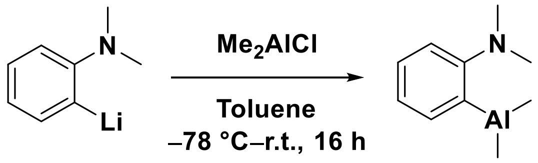

2-Lithio-*N,N*-dimethylaniline (7.85 mmol, 1.00 g) was added to toluene (50.0 mL) and the resultant suspension stirred and cooled to –78 °C. Dimethyl aluminium chloride solution in hexanes (7.85 mmol, 7.85 mL, 1.00 M) was added dropwise. The solution was then allowed to warm to room temperature and stirred for 16 hours. The solution was filtered then all solvent removed *in vacuo* to leave the crude product as a yellow oil. Vacuum sublimation directly from this oil (0.1 mbar, 60 – 70 °C) gave the *alane* as a colourless cubiods (620 mg, 3.53 mmol, 45%; CCDC deposition number: 2074250).

^{1}H NMR: (500.12 MHz, C_6D_6)
–0.30 (6H, s), 2.10 (6H, s), 6.61-6.64 (1H, m), 7.10-7.14 (1H, m), 7.17-7.20 (1H, m), 7.69-7.71 (1H, m).

^{13}C NMR: (125.77 MHz, C_6D_6)
–9.1 (br), 46.8, 116.3, 129.0, 136.9, 152.6 (br), 159.0. One C–H carbon peak not observed due to overlap with the solvent peak.

^{27}Al NMR: (130.34 MHz, C_6D_6)
182 (s br).

MS: (HRMS – EI$^+$)
Found: 177.10995 ($C_{10}H_{16}AlN$), requires: 177.10927.

CHN: Found: C, 62.6; H, 8.3; N 7.6. Calc. for $C_{10}H_{16}AlN$: C, 67.8; H 9.1; N 7.9%

MP: 63 – 65 °C (Toluene)

Preparation of tris(phenylacetyl)aluminium-*N,N*-dimethylaniline adduct (6)

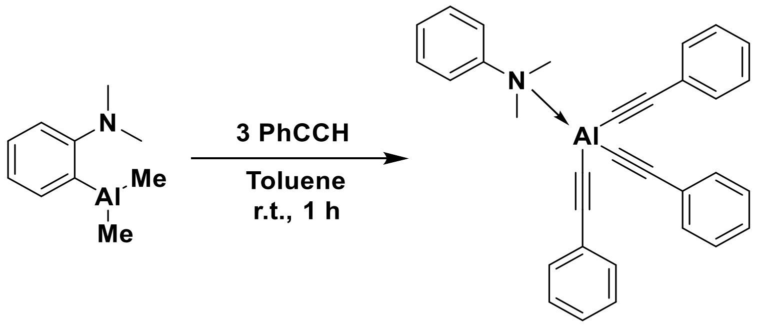

2-Dimethylaluminium-*N,N*-dimethylaniline **1b** (1.15 mmol, 0.204 g) was dissolved in toluene (20 ml), to which phenylacetylene (3.46 mmol, 0.380 mL) was added dropwise and stirred for 1 hour. The solution was then concentrated in vacuo to remove residual phenyacetylene, precipitating upon cooling the acetylide product **8** as a colourless amorphous solid (0.268 g, 0.760 mmol, 66%). The alkynylaluminium product was recrystallized from minimum toluene at 2 °C to give colourless block crystals for XRD structure determination (CCDC deposition number: 2074252).

^{1}H NMR: (500.12 MHz, C_6D_6)
2.84 (s, 6H), 6.87-7.07 (m, 9H), 7.10 (t, 2H, *J* = 7.4 Hz), 7.27 (2H, m), 7.38-7.48 (5H, m). One C–H signal not observed due to overlap with the solvent.

^{13}C NMR: (125.77 MHz, C_6D_6)
43.2, 77.8, 83.9, 108.4, 117.3, 121.7, 122.9, 125.4, 129.2, 132.4, 149.2.

MP: 100 °C (toluene) – melts with decomposition.

Preparation of 2-dimethylaluminium-*N,N*-dimethylaniline-HBpin adduct (7)

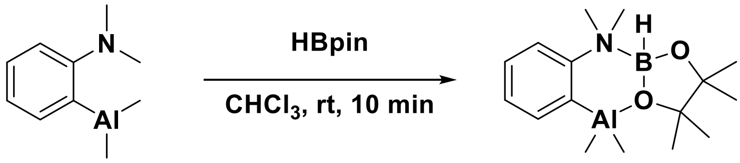

2-Dimethylaluminium-*N,N*-dimethylaniline **1b** (0.500 mmol, 90.0 mg) and HBpin (1.40 mmol, 200 µL) were dissolved in $CHCl_3$ (3.00 mL) at room temperature. Volatiles were removed *in vacuo* to yield the crude adduct **7** as colourless oil (160 mg, 0.524 mmol, >100% {a small portion of irremovable HBpin remained in the crude oil}). The oil was dissolved in pentane (2.00 mL) then left at –40 °C for 16 hours, to give colourless cubic crystals suitable for X-ray crystallography (CCDC deposition number: 2074251). Preparative isolation of crystalline material was complicated by the low-melting point (below room temperature) of the adduct **7**, which prevented full characterization.

^{1}H NMR: (500.12 MHz, C_6D_6)
-0.22 (3H, s), -0.12 (3H, s), 1.03 (3H, s), 1.19-1.20 (6H, overlapping singlets), 1.34 (3H, s), 2.28 (3H, s), 2.42 (3H, s), 3.35 (1H, 1:1:1:1 q, *J* = 154 Hz), 6.67 (1H, d, *J* = 8.4 Hz), 7.01-7.05 (1H, m), 7.14 (1H, td, *J* = 6.87 Hz, *J* = 0.9 Hz), 7.82 (1H, dd, *J* = 7.0 Hz, *J* = 1.8 Hz).

^{11}B NMR: (128.34 MHz, C_6D_6)
6.02 (d, *J* = 134.6 Hz).

^{13}C NMR: (125.77 MHz, C_6D_6)
–8.2 (br), –6.68 (br), 23.9, 24.7, 25.1, 25.4, 44.7, 49.0, 78.3, 87.1, 116.7, 127.1, 127.5, 138.5, 151.1 (br), 155.1.

Preparation of bis-{(di-2-*N,N*-dimethylaniline aluminium hydride)-alane adduct} (8)

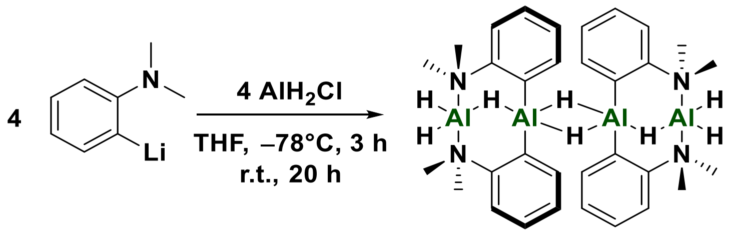

2-Lithio-*N,N*-dimethylaniline (7.87 mmol, 1.00 g) was dissolved in THF (30.0 ml) and cooled to –78 °C, to which a solution of AlH_2Cl in THF (15.0 mmol, 30.0 mL, 0.500 M) was added dropwise. The yellow solution was stirred for 3 hours, warmed to room temperature and stirred for 20 h. The solution turned pale yellow over the course of the reaction. Volatiles were removed *in vacuo* to yield a yellow oil. Toluene (25.0 mL) was added, and the solution filtered. The filtrate was concentrated and hexane added, such that the solution was approximately a 1:1 volume ratio of toluene and hexane. The solution was cooled to –20 °C, affording bis-{*(di-2-N,N-dimethylaniline aluminium hydride)-alane} adduct* as colourless block crystals (0.800 g, 1.34 mmol, 68%). The alane adduct was dissolved in minimum diethyl ether at room temperature, then slowly cooled to afford colourless block crystals of *bis-{(di-2-N,N-dimethylaniline aluminium hydride)-alane adduct}* suitable for XRD structure determination (CCDC deposition number: 2074253). By NMR spectroscopy, the adduct was found to be in a variety of coordination modes.

^{1}H NMR: (500.12 MHz, C_6D_6)
2.29-2.72 (24H, m), 4.08 (8H, br s), 6.44-6.66 (2H, m), 6.70-6.89 (4H, m), 7.00-7.48 (6H, m), 7.81-8.36 (4H, m).

^{13}C NMR: (125.77 MHz, C_6D_6)
40.3, 46.5-47.0 (br), 47.8, 113.1, 116.0 (br), 116.3 (br), 117.1, 117.3, 123.8, 123.9 (br), 124.8, 128.5, 128.7, 129.4, 140.7, 140.9 (br), 141.2, 151.1, 160.5, 160.8.

MS: (HRMS – EI^+)
Found: 596.31288 ($C_{32}H_{48}N_4{}^{27}Al_4$), requires: 596.31351.

Preparation of Diphenylmethylaluminium

$$Me_2AlCl + PhMgCl \xrightarrow[-78\ °C]{THF} Me_2AlPh + MgCl_2$$

By the method of Zezschwitz *et al.*,[10] PhMgCl (2.00 mmol, 2 M in hexanes, 1.00 mL) was added to THF (1.00 mL), then cooled to –78 °C. Me_2AlCl (2.00 mmol, 1 M in hexanes, 2.00 mL) was added dropwise with stirring. The mixture was warmed to room temperature and left stirring for 16 hours. Pentane (1.00 mL) was added, then the precipitate was allowed to settle. The top pentane layer was used in reactions without further purification, assuming 0.4 M concentration.

Note: Attempts were made to isolate this species by recrystalization as it has not been fully characterized before, however, we were unsuccessful.

7. Preparation of Deuterated Compounds

Preparation of 1-deutero-4'-fluorophenylacetylene

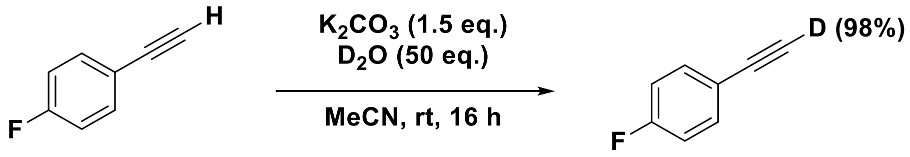

By the method of Poullain *et al.*,[11] 4'-fluorophenylacetylene (5.00 mmol, 573 µL) was dissolved in MeCN (10.0 mL), then K_2CO_3 (7.50 mmol, 1.04 g) was added and left to stir for 30 minutes. D_2O (100 mmol, 2.00 mL) was added, then left to stir for 16 hours. CH_2Cl_2 (25 mL) was added, the organic layer extracted and dried ($MgSO_4$). The solvent was removed *in vacuo*, to give 1-deutero-4'-fluorophenylacetylene as a colourless oily solid (473.2 mg, 78%). The deuterium incorporation was calculated to be 98% by integration of the residual alkyne C–H signal (δ 3.04 ppm, $CDCl_3$).

^{1}H NMR: (500.12 MHz, $CDCl_3$)
7.02 (2H, t, *J* = 8.6 Hz), 7.48 (2H. m).

^{2}D NMR: (76.75 MHz, $CDCl_3$)
3.04 (s).

^{13}C NMR: (125.77 MHz, $CDCl_3$)
76.8 (1:1:1 t, *J* = 41.2 Hz), 82.3 (1:1:1 t, *J* = 7.3 Hz), 115.8 (d, *J* = 23.8), 118.3 (d, *J* = 3.5 Hz), 134.2 (d, *J* = 8.7 Hz), 163.4 (d, *J* = 250.4 Hz).

Preparation of DBpin

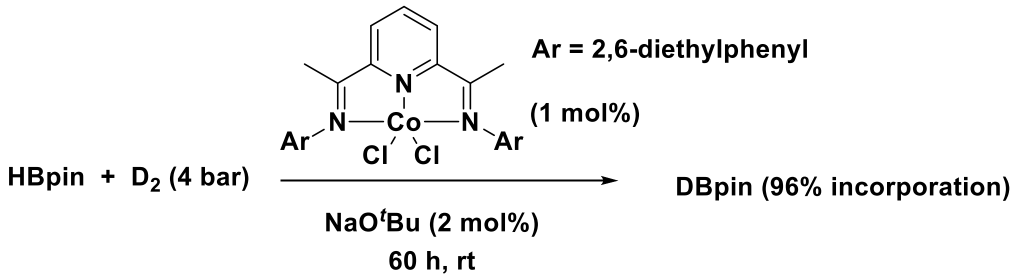

By the method of Thomas *et al.*,[12] under an atmosphere of argon, to a pressure vessel was added $^{Et}BIPCoCl_2$ (0.140 mmol, 76.0 mg), and NaO*t*Bu (0.280 mmol, 30.0 mg). HBpin (2.00 mL, 14.0 mmol) was added dropwise (the reaction of HBpin and NaO*t*Bu it highly exothermic and has a long induction period). The vessel was sealed, flushed with D_2, then pressurised under an atmosphere of D_2 (4 bar). The reaction was left stirring at room temperature for 36 h, then the flush/pressurise cycle was repeated, and the reaction was left stirring for another 24 h. The crude reaction mixture was distilled (60 mbar, 40 °C) to give DBpin as a colourless oil (0.500 g, 3.50 mmol, 25%). The deuterium incorporation was determined by integration of ¼ of the residual HBpin signal.

^{1}H NMR: (500.12 MHz, $CDCl_3$)
1.27 (12H, s).

^{11}B NMR: (128.34 MHz, $CDCl_3$)
28.2 (s br).

^{13}C NMR: (125.77 MHz, $CDCl_3$)
25.0, 83.4.

^{2}D NMR: (76.75 MHz, toluene)
4.15 (br m).

Analytical data were in accordance with those previously reported.[12]

8. Mechanistic Analysis

8.1. Deuterium Labelling

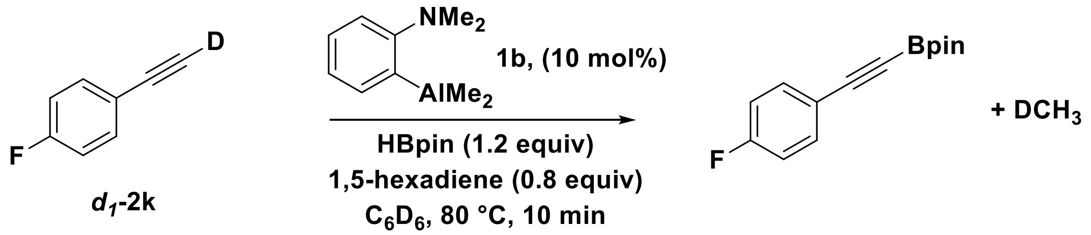

To an NMR tube with a J-Young tap fitted, 2-dimethylaluminium-*N,N*-dimethylaniline **1b** (0.050 mmol, 9.0 mg), HBpin (0.600 mmol, 87.0 µL), and 1,5-hexadiene (0.400 mmol, 48.0 µL) were dissolved in C_6D_6 (0.500 mL) then cooled to –196 °C. d_1-4'-Fluorophenyl acetylene (0.500 mmol, 55.0 µL) was added and the tube quickly sealed. The tube was allowed to warm to room temperature, whereby it was subjected to ^1H NMR spectroscopy where no DCH_3 was observed. The tube was heated to 80 °C for 10 minutes, then subjected to ^1H and ^{13}C NMR spectroscopy, where DCH_3 was observed. (^1H NMR: δ 0.125 ppm, 1:1:1 t, J = 1.9 Hz; consistent with literature coupling constant.[13] ^{13}C NMR: δ 4.55 ppm, 1:1:1 t, J = 19.1 Hz; consistent with literature coupling constant[14]). Trace MeBpin (δ 0.31 ppm, s; <0.005 mmol) was also observed, using the 1,5-hexadiene as an internal standard (assumed to have remained constant).

^{1}H NMR (500.12 MHz, C_6D_6):

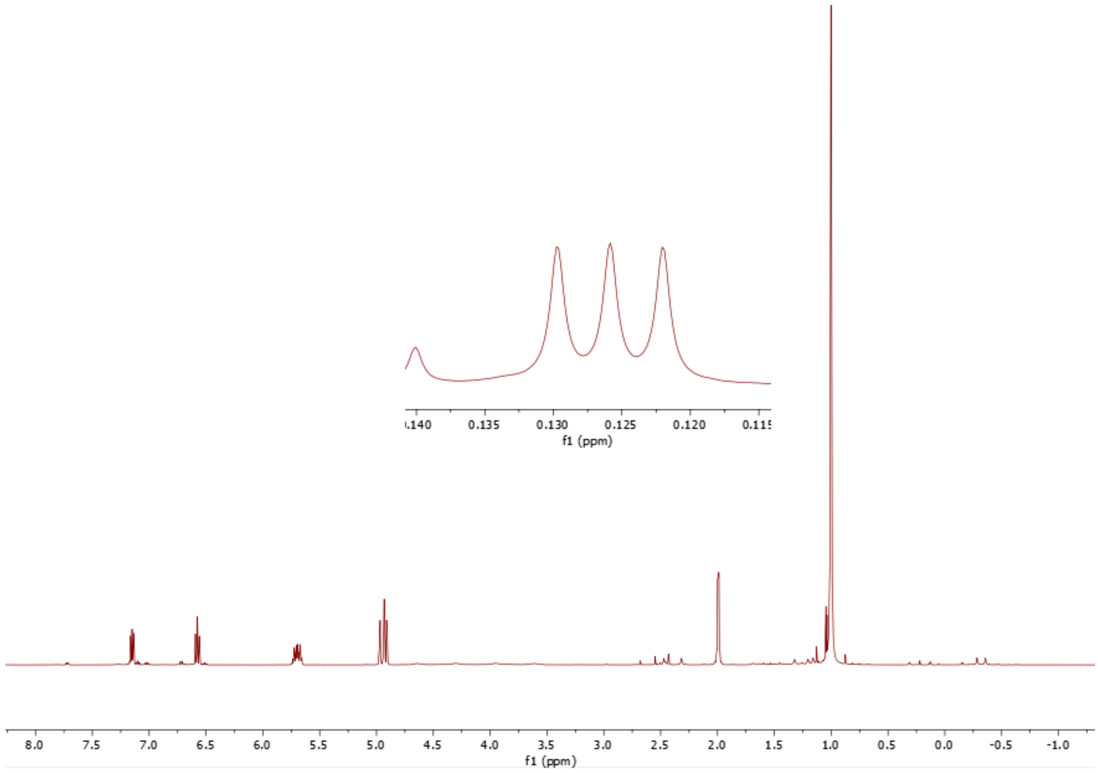

^{13}C (125.77 MHz, C_6D_6):

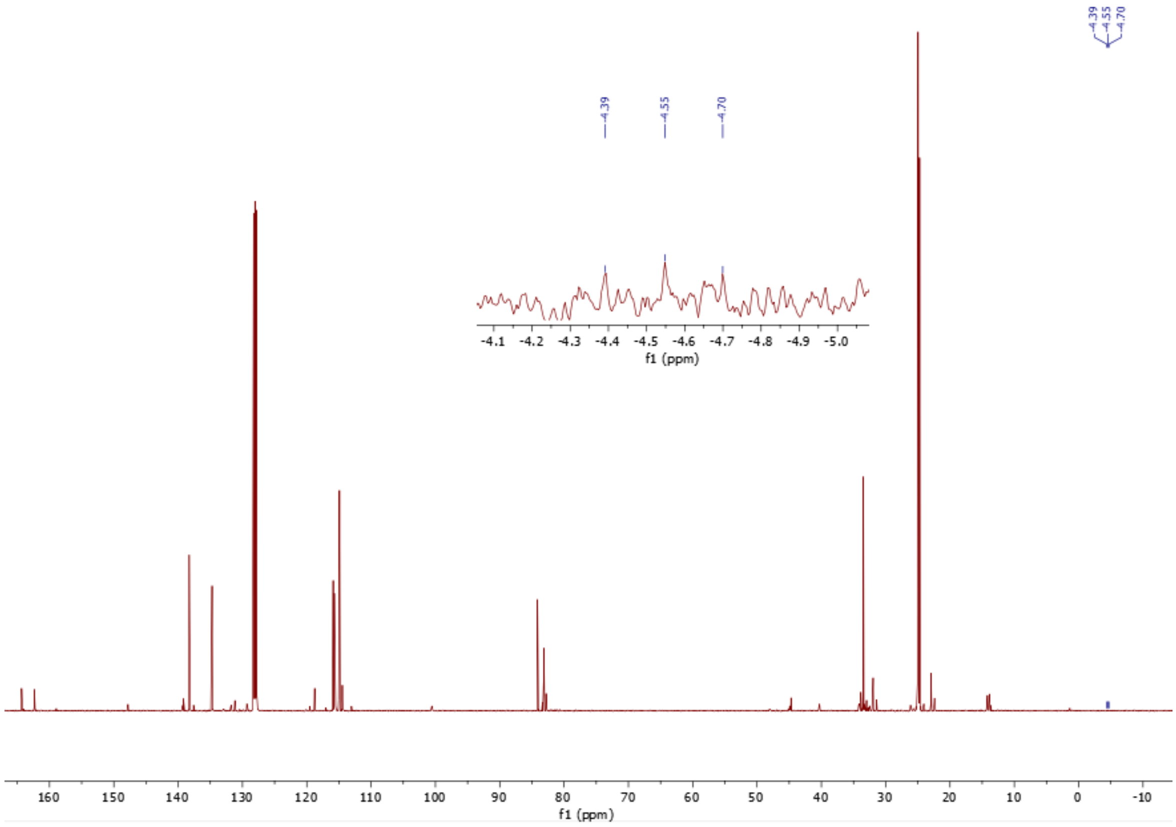

8.2. VTNA Kinetics

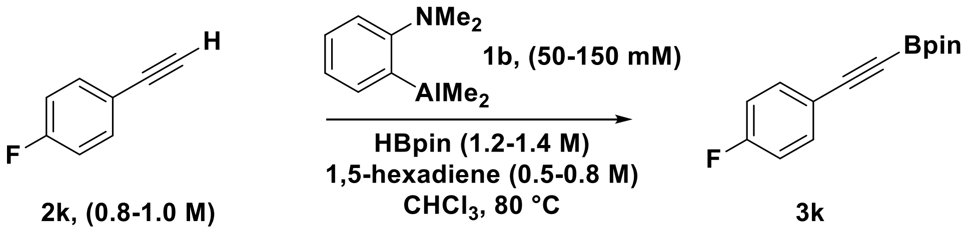

All kinetic reactions followed a general procedure: Under an inert atmosphere, 2-dimethylaluminium-*N,N*-dimethylaniline **1b** (50-150 µmol) and HBpin (1.20-1.40 mmol) were dissolved in chloroform (1.00 mL). 1,5-Hexadiene (0.500-0.800 mmol) was added, then 4'-fluorophenylacetylene (0.800-1.00 mmol). The reaction was immediately heated to 80 °C and an aliquot (<20 µL) was added to C_6D_6 in air during the course of the reaction. The rate was determined by disappearance of starting material and appearance of product in the ^{19}F NMR spectrum.

Following the method described by Burés,[15] the concentration of each component was varied systematically while all the others remained the same. The kinetic profiles were then plotted on a time-normalised axis with appearance of **3k** and the disappearance of **2k**.

*Variation in **2k***

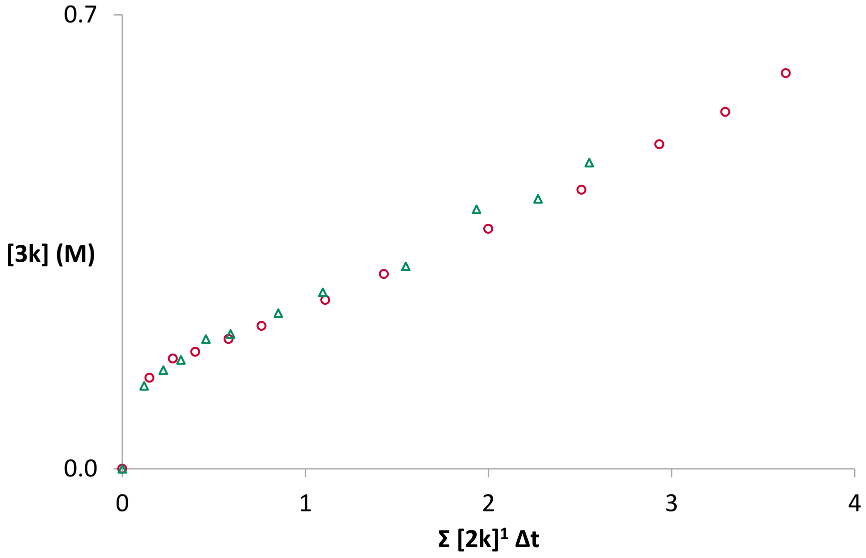

Figure S1. [3k] against normalised time scale with $[2k]_0$ = 0.80 M, 1.00 M.

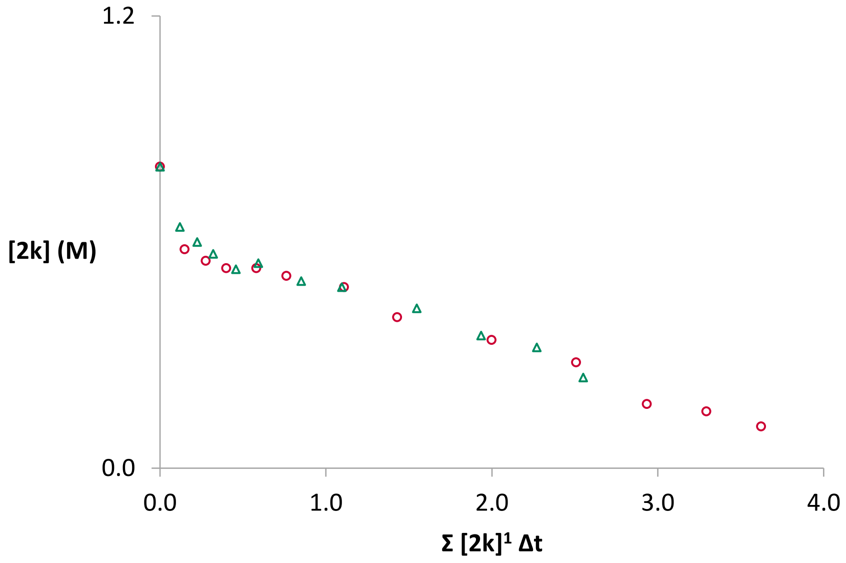

Figure S2. [2k] against normalised time scale with $[2k]_0$ = 0.80 M, 1.00 M.

The best overlap was achieved with an order of 1 with respect to **2k**.

Variation in HBpin

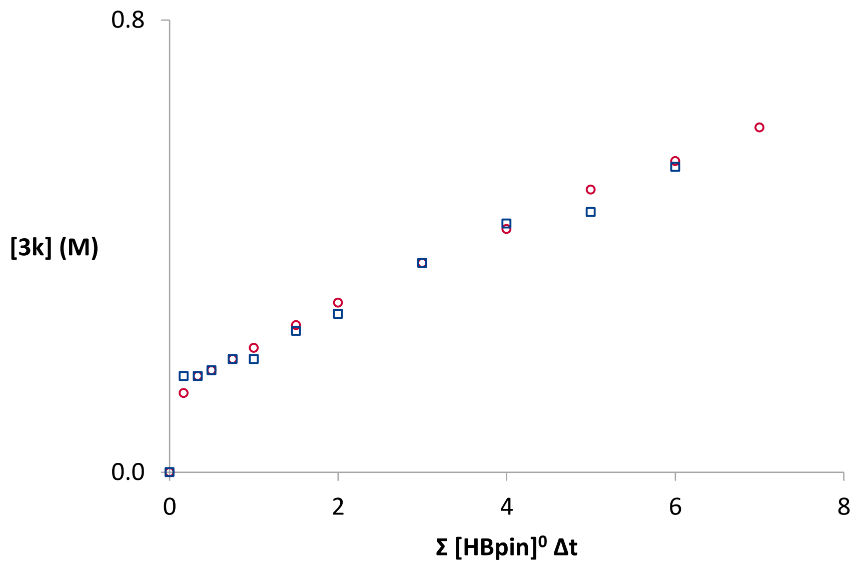

Figure S3. [3k] against normalised time scale with $[HBpin]_0$ = 1.20 M, 1.40 M.

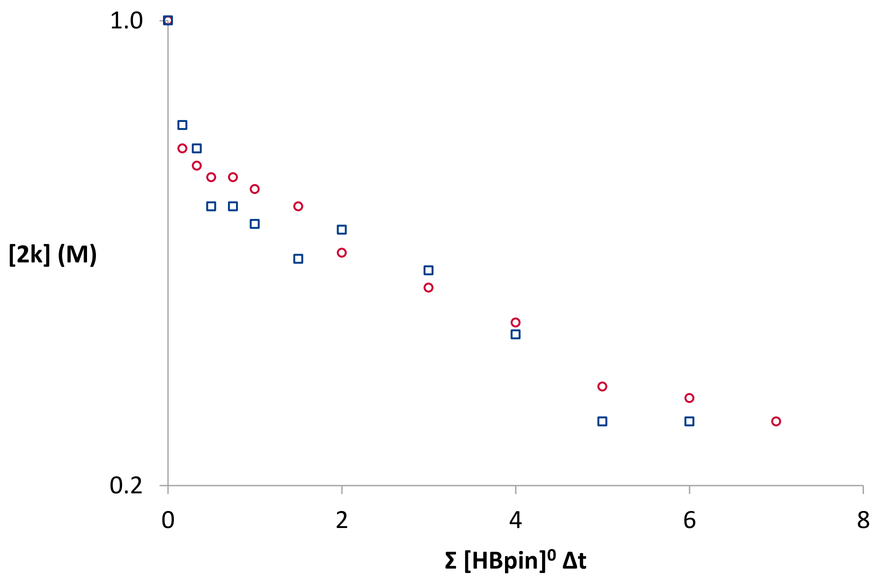

Figure S4. [2k] against normalised time scale with $[HBpin]_0$ = 1.20 M, 1.40 M.

The best overlap was achieved with an order of 0 with respect to HBpin

Variation in 1,5-hexadiene

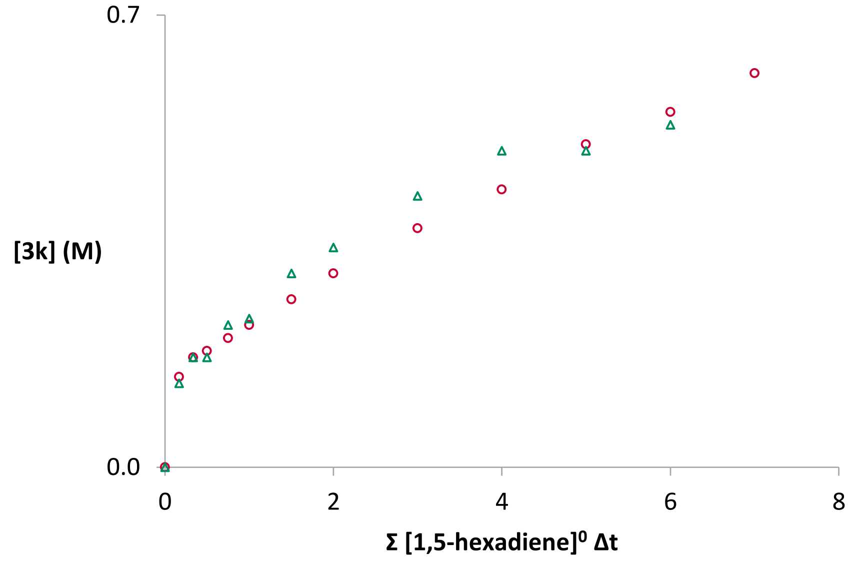

Figure S5. [3k] against normalised time scale with $[1,5\text{-hexadiene}]_0$ = 0.50 M, 0.80 M.

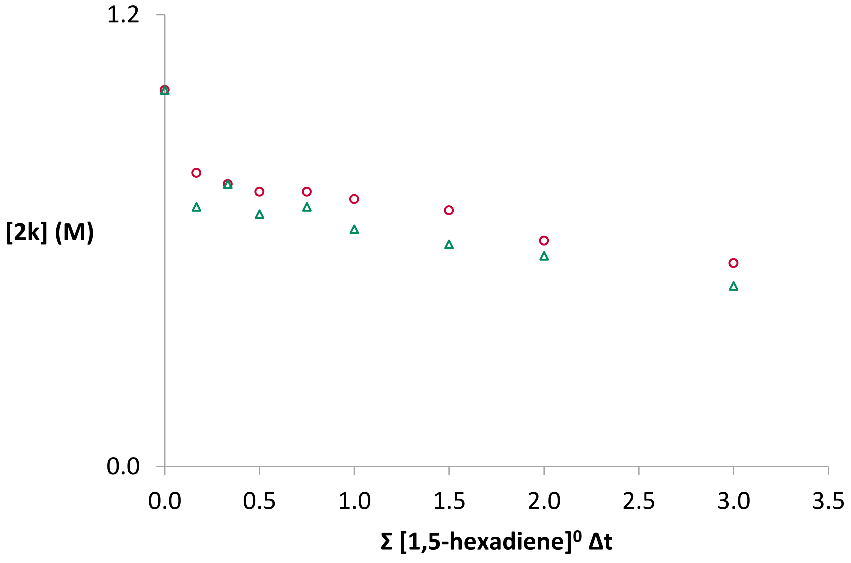

Figure S6. [2k] against normalised time scale with $[1,5\text{-hexadiene}]_0$ = 0.50 M, 0.80 M.

The best overlap was achieved with an order of 0 with respect to 1,5-hexadiene when tracking product formation. The overlap was not very good when it came to tracking starting material, possibly due to increased background hydroboration.

Variation of catalyst

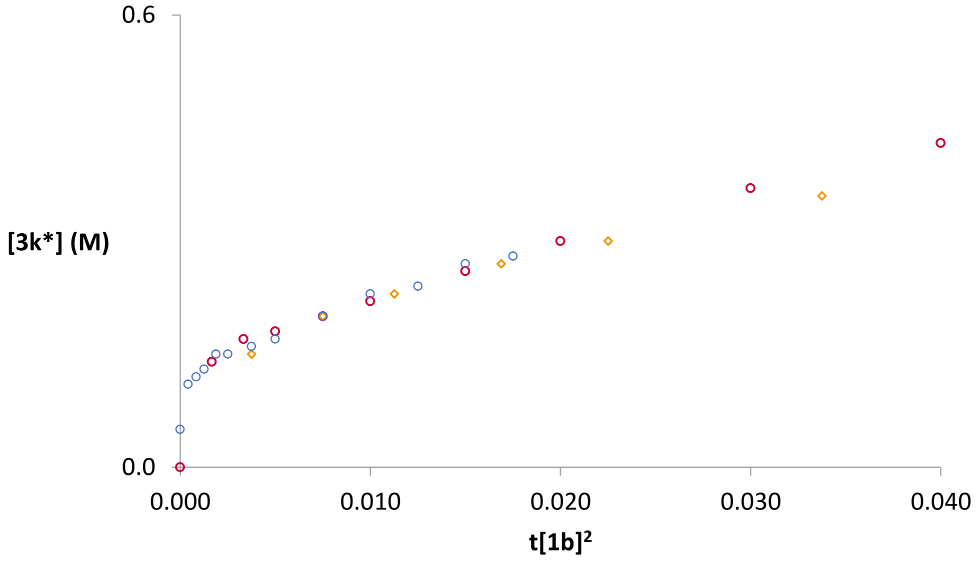

Figure S7. [3k*] against normalised time scale with [1b] = 0.05 M, 0.10 M. 0.15 M.

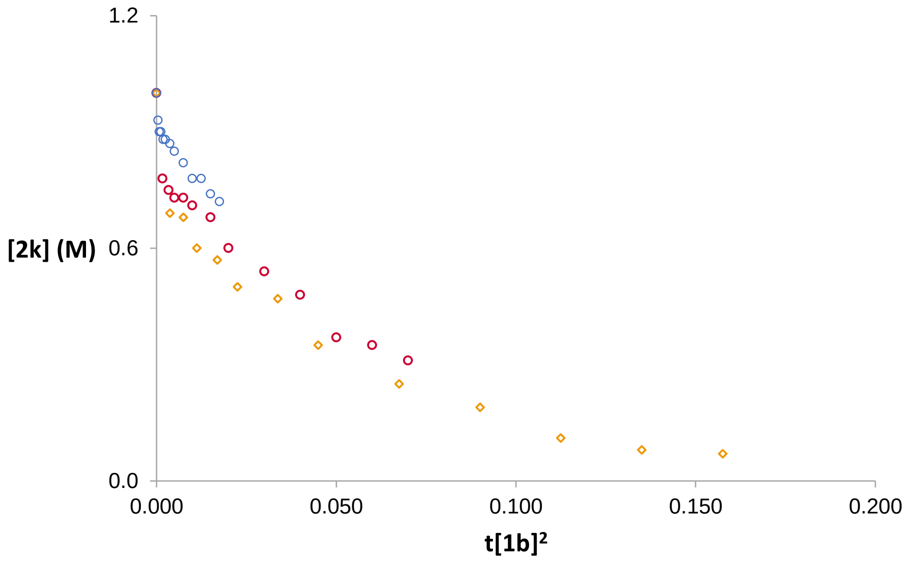

Figure S8. [2k] against normalised time scale with [1b] = 0.05 M, 0.10 M. 0.15 M.

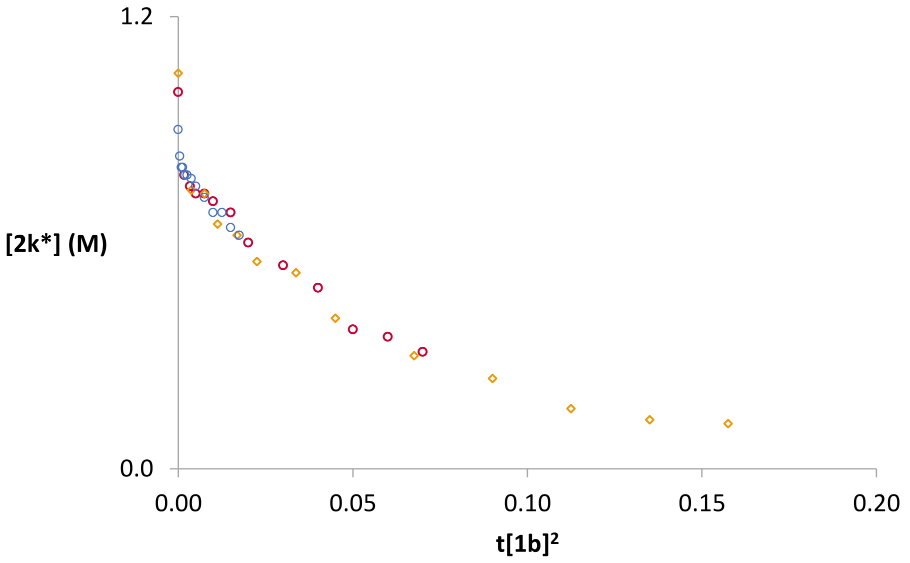

Figure S9. [2k*] against normalised time scale with [1b] = 0.05 M, 0.10 M. 0.15 M.

*When the catalyst loading was increased, there was a sharp initial increase in product formation approximately equal to the loading. This lead to parallel curves, so the values were normalised according to the loading, *e.g.*:

$$[3k^*] = [3k] - \Delta[1b]$$

The best overlap was achieved with an order of 2 with respect to **1b**.

Overall

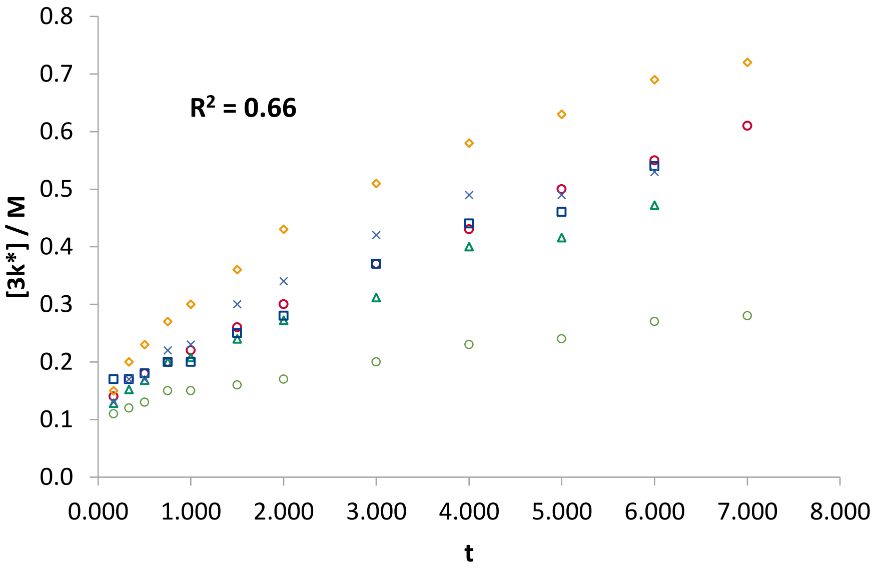

Figure S10. [3k*] against time with $[2k]_0$ = 0.80 M, 1.00 M; $[HBpin]_0$ = 1.20 M, 1.40 M; [1,5-hexadiene]$_0$ = 0.50 M, 0.80 M; [1b] = 0.05 M, 0.10 M. 0.15 M.

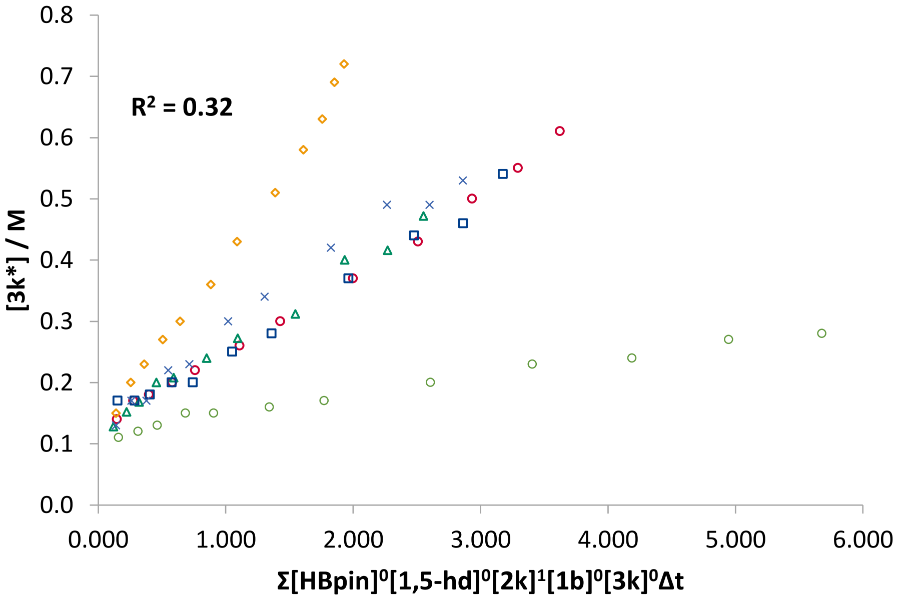

Figure S11. [3k*] against normalised time scale ($[2k]^1$) with $[2k]_0$ = 0.80 M, 1.00 M; $[HBpin]_0$ = 1.20 M, 1.40 M; [1,5-hexadiene]$_0$ = 0.50 M, 0.80 M; [1b] = 0.05 M, 0.10 M. 0.15 M.

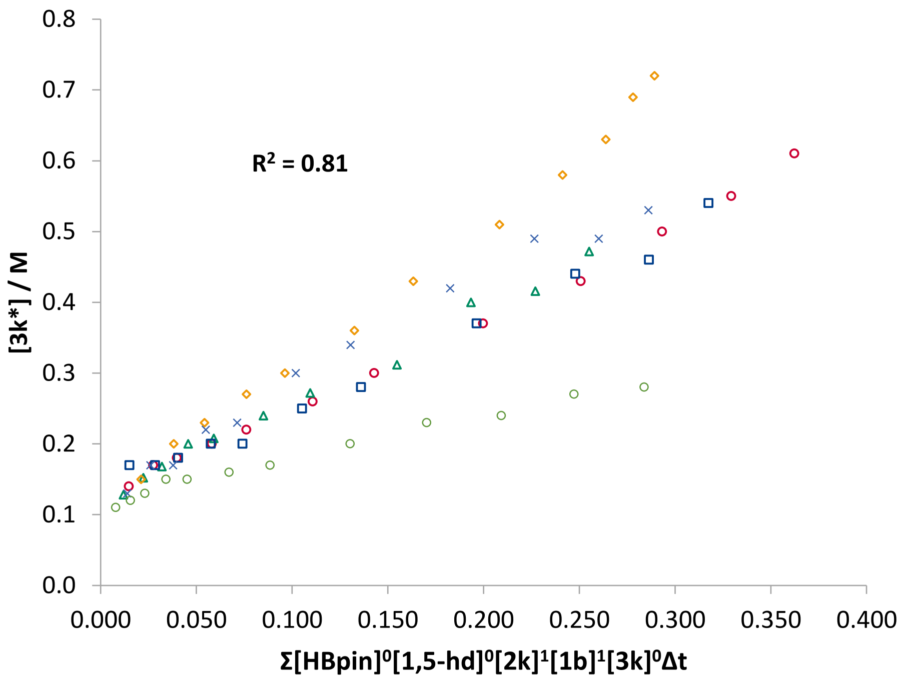

Figure S12. [3k*] against normalised time scale ($[2k]^1[1b]^1$) with $[2k]_0$ = 0.80 M, 1.00 M; $[HBpin]_0$ = 1.20 M, 1.40 M; $[1,5\text{-hexadiene}]_0$ = 0.50 M, 0.80 M; [1b] = 0.05 M, 0.10 M. 0.15 M.

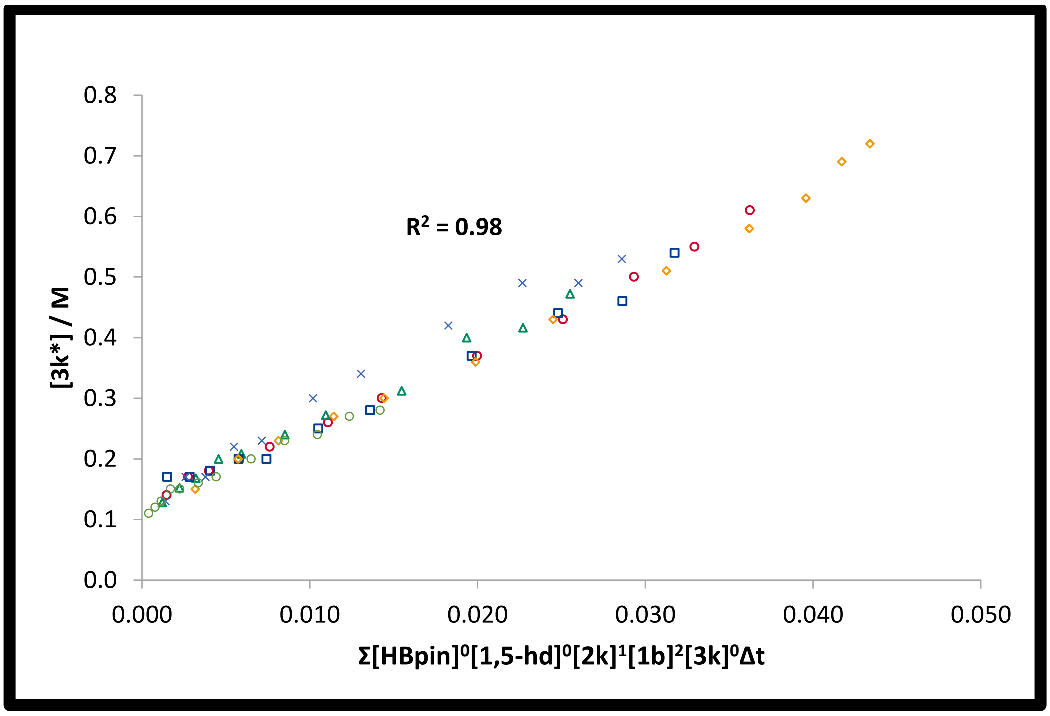

Figure S13. [3k*] against normalised time scale ($[2k]^1[1b]^2$) with $[2k]_0$ = 0.80 M, 1.00 M; $[HBpin]_0$ = 1.20 M, 1.40 M; $[1,5\text{-hexadiene}]_0$ = 0.50 M, 0.80 M; [1b] = 0.05 M, 0.10 M. 0.15 M.

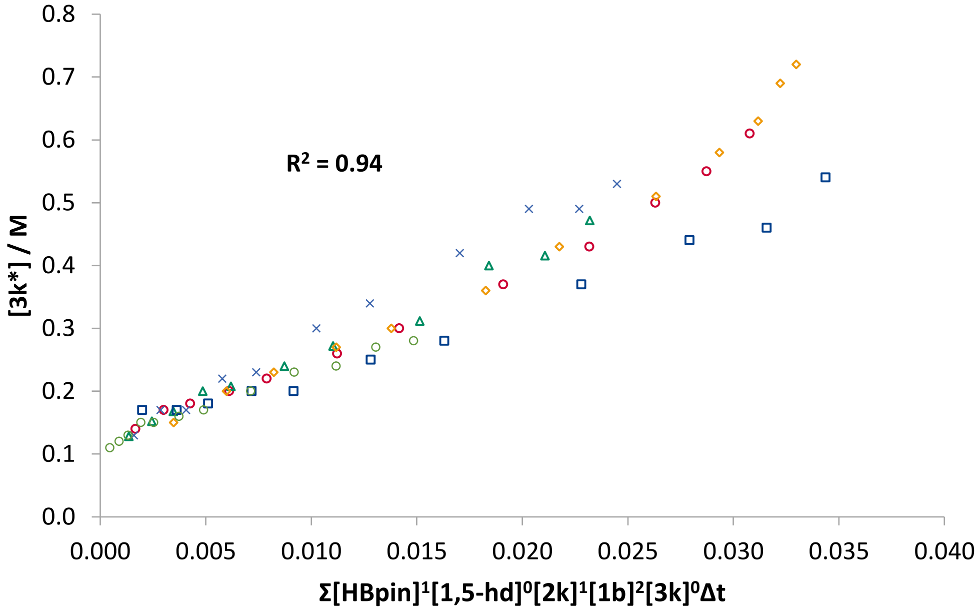

Figure S14. [3k*] against normalised time scale ($[2k]^1[1b]^2[HBpin]^1$) with $[2k]_0$ = 0.80 M, 1.00 M; $[HBpin]_0$ = 1.20 M, 1.40 M; $[1,5\text{-hexadiene}]_0$ = 0.50 M, 0.80 M; [1b] = 0.05 M, 0.10 M. 0.15 M.

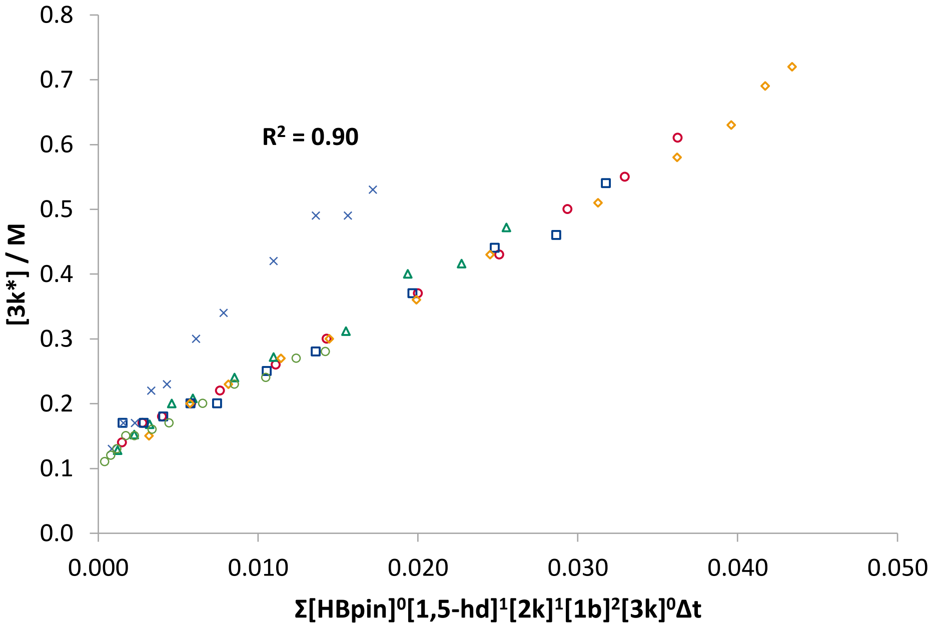

Figure S15. [3k*] against normalised time scale ($[2k]^1[1b]^2[1,5\text{-hexadiene}]^1$) with $[2k]_0$ = 0.80 M, 1.00 M; $[HBpin]_0$ = 1.20 M, 1.40 M; $[1,5\text{-hexadiene}]_0$ = 0.50 M, 0.80 M; [1b] = 0.05 M, 0.10 M. 0.15 M.

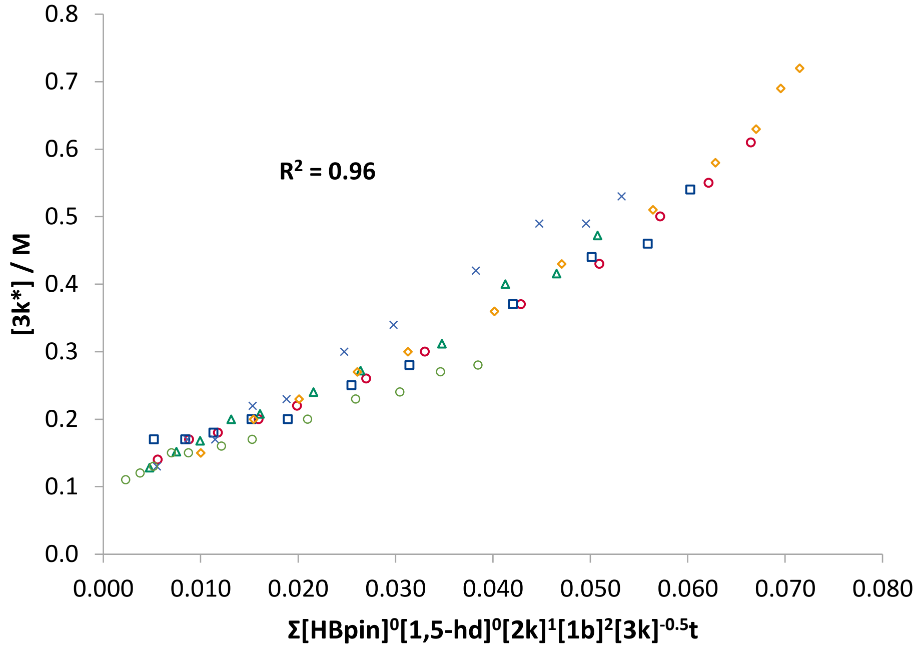

Figure S16. [3k*] against normalised time scale ($[2k]^1[1b]^2[3k]^{-0.5}$) with $[2k]_0$ = 0.80 M, 1.00 M; $[HBpin]_0$ = 1.20 M, 1.40 M; $[1,5\text{-hexadiene}]_0$ = 0.50 M, 0.80 M; [1b] = 0.05 M, 0.10 M. 0.15 M.

Optimal overlap was achieved with: **$\Sigma[HBpin]^0[1,5\text{-hd}]^0[2k]^1[1b]^2[3k]^0\Delta t$** (as denoted by black box).

8.3. Kinetic Isotope Effect

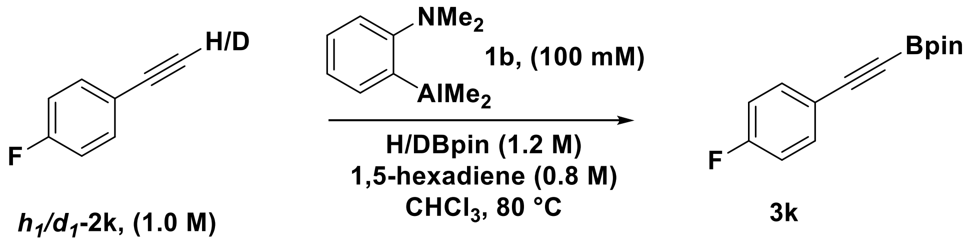

Under an inert atmosphere, in an NMR tube with a J-Young tap fitting (capabable of sustaining high pressures), 2-dimethylaluminium-*N,N*-dimethylaniline **1b** (50 µmol, 9.0 mg) and either DBpin (0.600 mmol, 78.0 mg) or HBpin (0.600 mmol, 87.0 µL) were dissolved in chloroform (0.500 mL). 1,5-Hexadiene (0.400 mmol, 48.0 µL) was added, then 1-deutero-4'-fluorophenylacetylene (0.500 mmol, 61.0 mg) or 4'-fluorophenylacetylene (0.500 mmol, 58.0 µL). In a Bruker Avance III 400 MHz spectrometer, the sample was shimmed at room temperature, then removed as the instrument heated to 80 °C. The sample was loaded into the instrument at temperature and spectra acquisition began immediately. The rate was determined by disappearance of starting material and appearance of product in the ^{19}F NMR spectroscopy by conversion. k_2 was calculated by plotting ln(***[2k]***) against *t* and taking the gradient of the straight line. ^{1}H and ^{2}D NMR spectra were recorded before and after each run.

Kinetic Plots

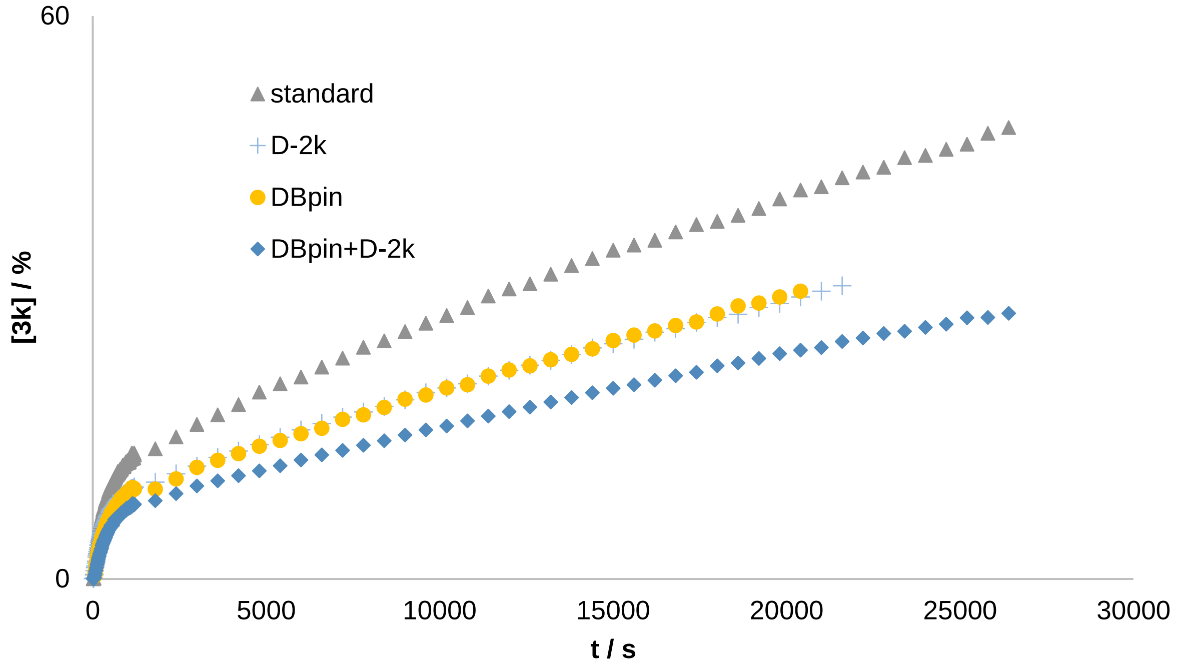

Figure S17. Kinetic isotope effect relative to [3k], where 'standard' is under standard conditions, 'D-2k' is with d_1-4'-fluorophenylacetyelene (***d_1*-2k**), 'DBpin' is with DBpin, and 'DBpin+D-2k' is with both DBpin and ***d_1*-2k**.

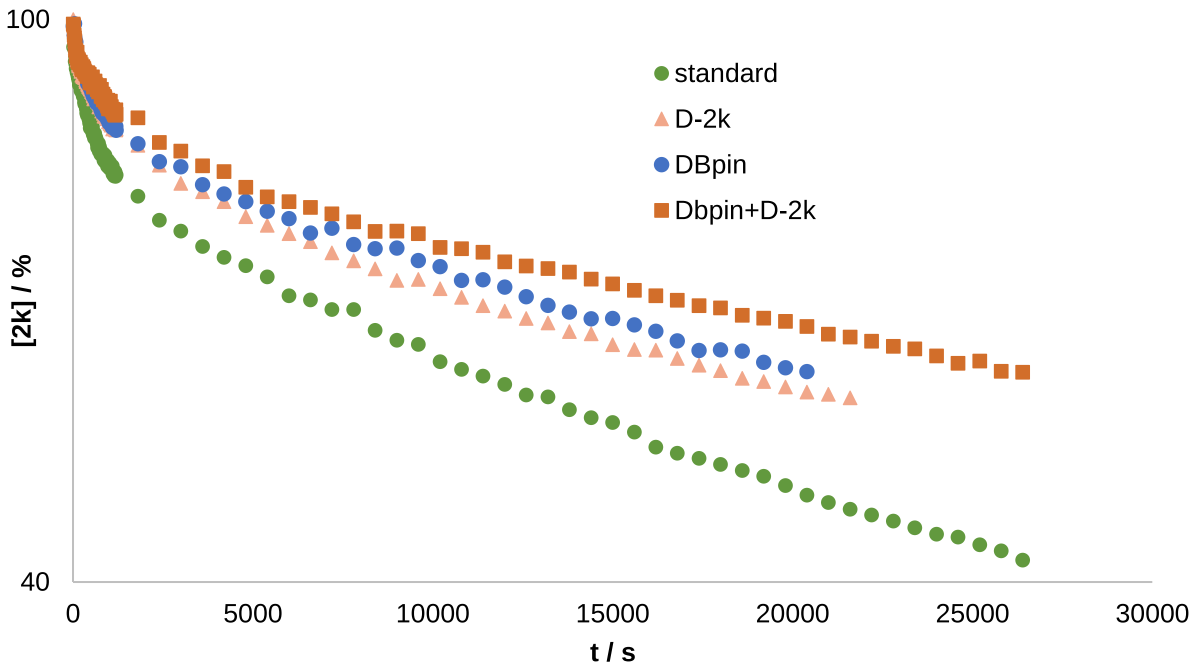

Figure S18. Kinetic isotope effect relative to [2k], where 'standard' is under standard conditions, 'D-2k' is with d_1-4'-fluorophenylacetyelene (***d_1*-2k**), 'DBpin' is with DBpin, and 'DBpin+D-2k' is with both DBpin and ***d_1*-2k**.

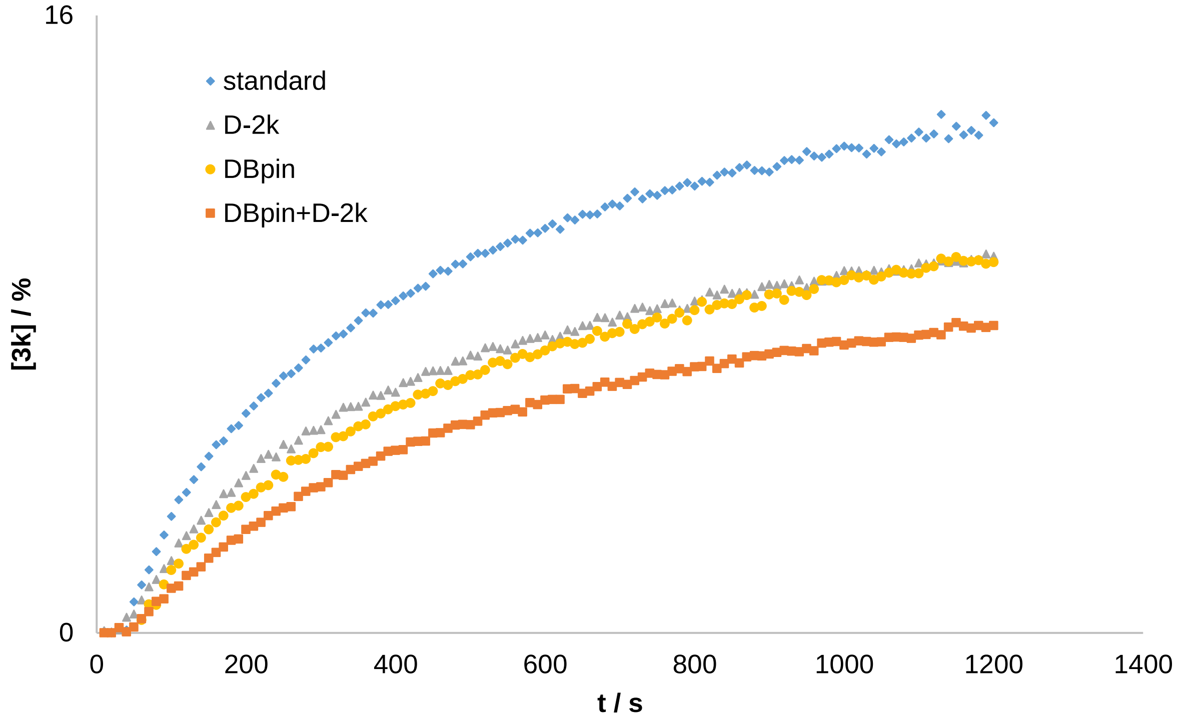

Figure S19. Kinetic isotope effect of k_1 relative to [3k], where 'standard' is under standard conditions, 'D-2k' is with d_1-4'-fluorophenylacetyelene (***d*₁-2k**), 'DBpin' is with DBpin, and 'DBpin+D-2k' is with both DBpin and ***d*₁-2k**.

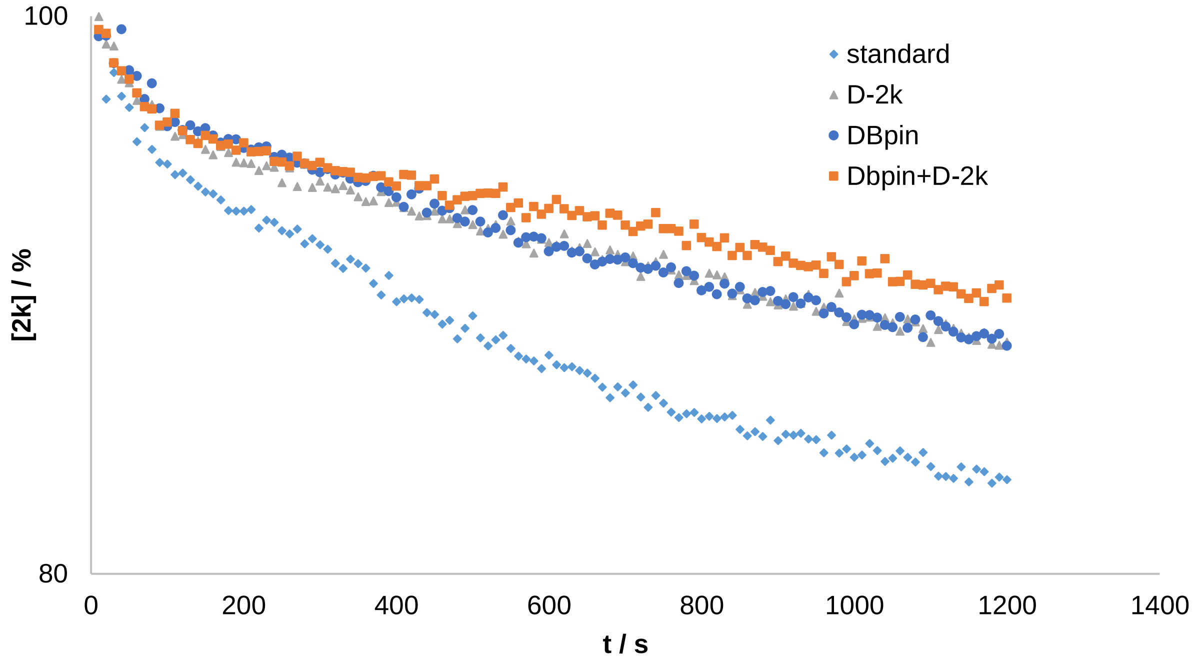

Figure S20. Kinetic isotope effect of k_1 relative to [2k], where 'standard' is under standard conditions, 'D-2k' is with d_1-4'-fluorophenylacetyelene (***d*₁-2k**), 'DBpin' is with DBpin, and 'DBpin+D-2k' is with both DBpin and ***d*₁-2k**.

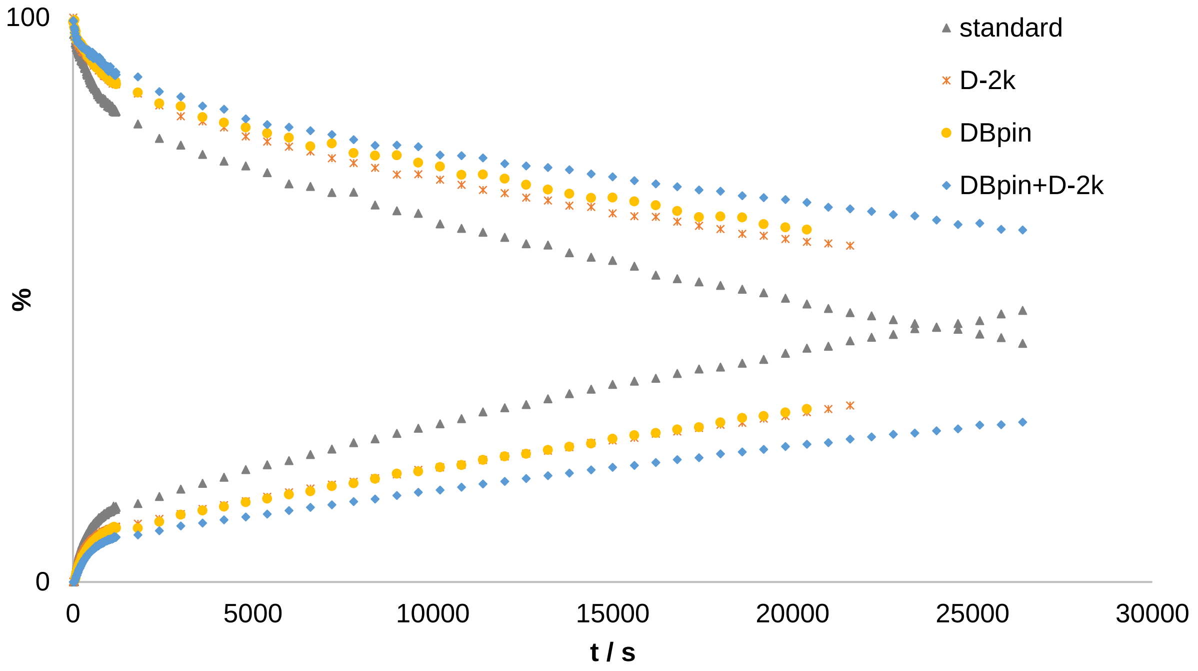

Figure S21. Kinetic isotope effect relative to [2k] and [3k], where 'standard' is under standard conditions, 'D-2k' is with d_1-4'-fluorophenylacetyelene (***d*$_1$-2k**), 'DBpin' is with DBpin, and 'DBpin+D-2k' is with both DBpin and ***d*$_1$-2k**.

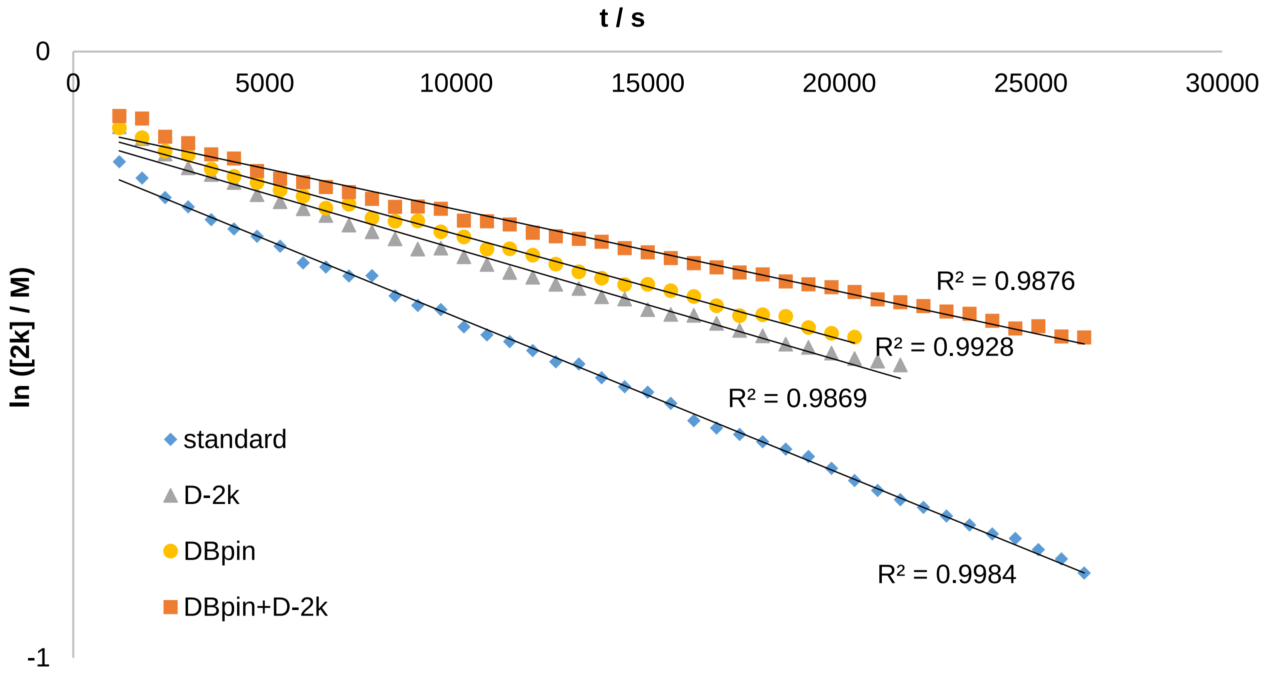

Figure S22. Kinetic isotope effect relative to ln[2k] where 'standard' is under standard conditions (k_2 = –2.57±0.03×10^{-5}), 'D-2k' is with d_1-4'-fluorophenylacetyelene (***d*$_1$-2k**) (k_2 = –1.84±0.07×10^{-5}), 'DBpin' is with DBpin (k_2 = –1.73±0.05×10^{-5}), and 'DBpin+D-2k' is with both DBpin and ***d*$_1$-2k** (k_2 = –1.35±0.05×10^{-5}).

*Typical ^{19}F NMR ($CHCl_3$, 376.50 MHz) spectra (δ –109.1 ppm = **3k**; δ –110.3 ppm = **2k**):*

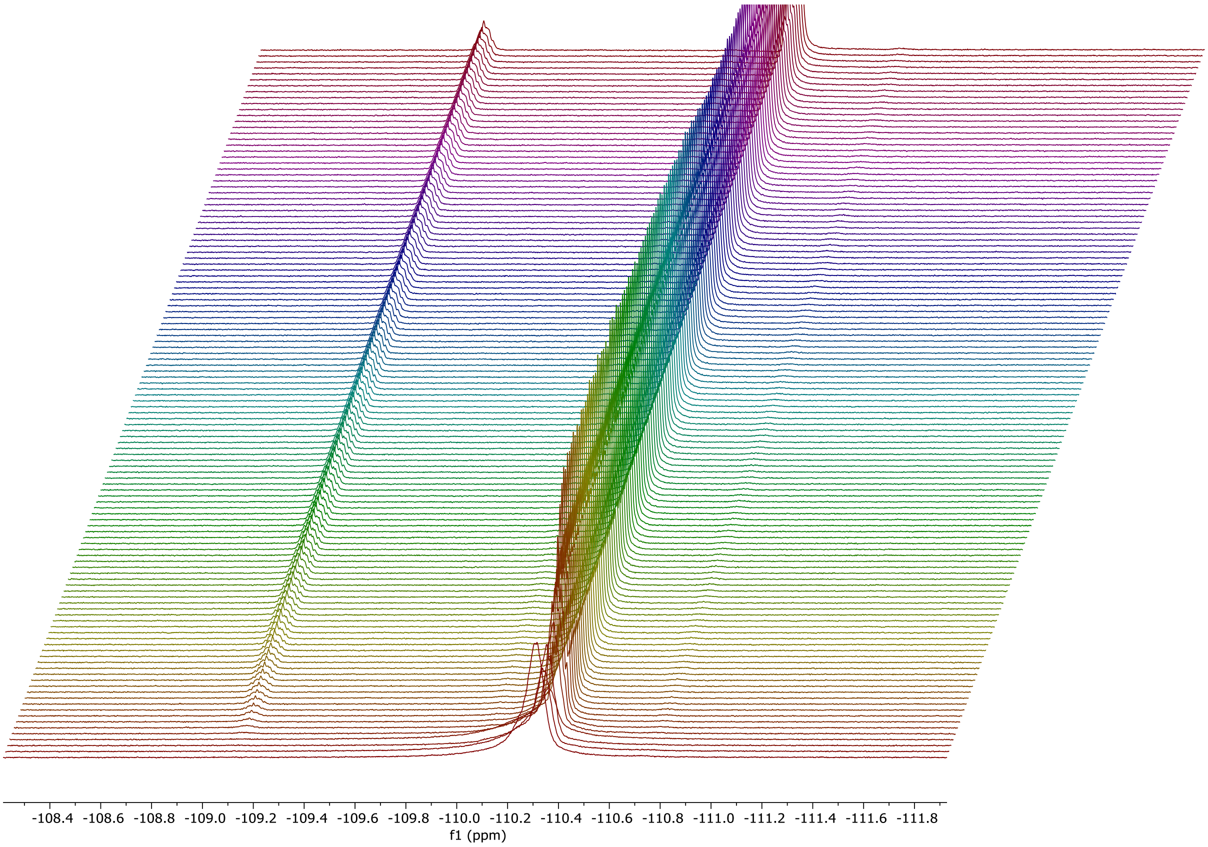

Figure S23. ^{19}F NMR ($CHCl_3$, 376.50 MHz) spectra against t, for the first 20 minutes of 'standard' reaction.

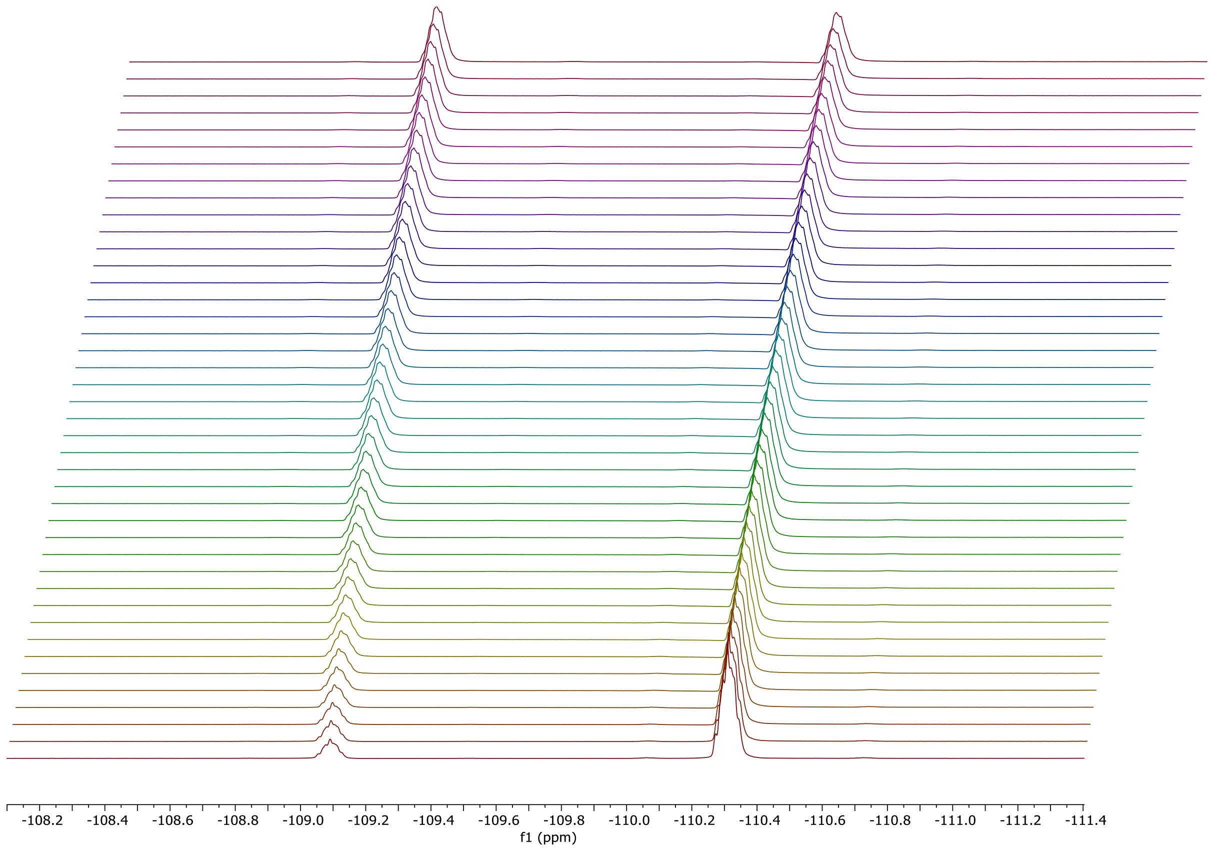

Figure S24. ^{19}F NMR ($CHCl_3$, 376.50 MHz) spectra against t, for 20 – 420 minutes of 'standard' reaction.

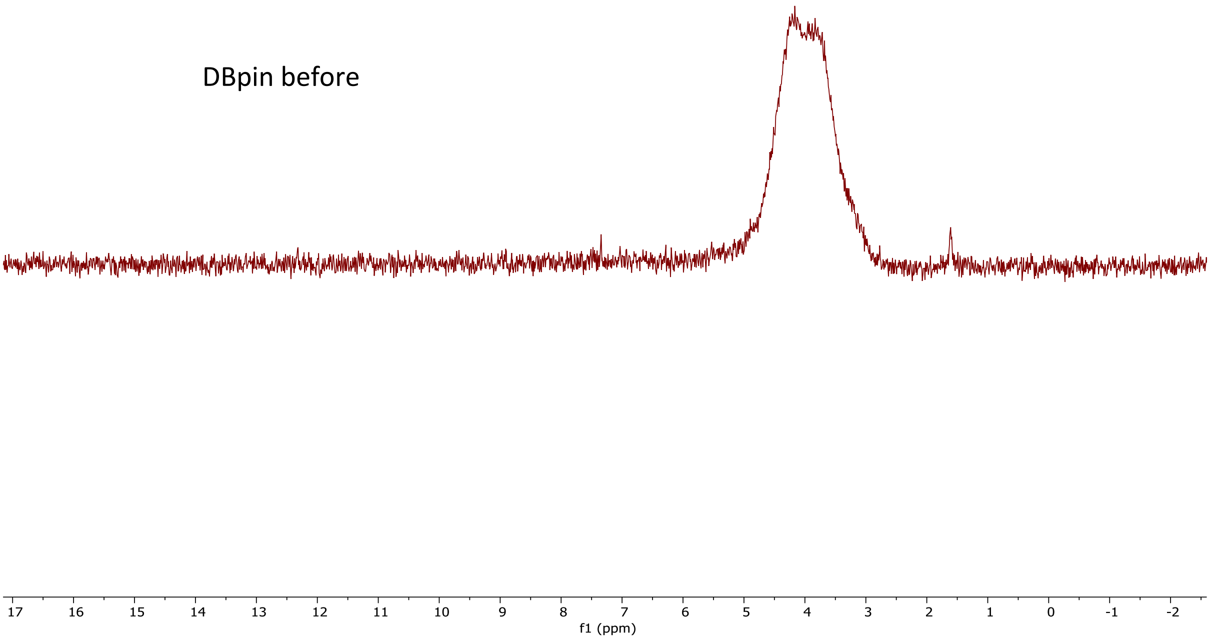

Figure S25. ^{2}D NMR (61.4 MHz, $CHCl_3$) spectrum of 'DBpin' reaction at t = 0.

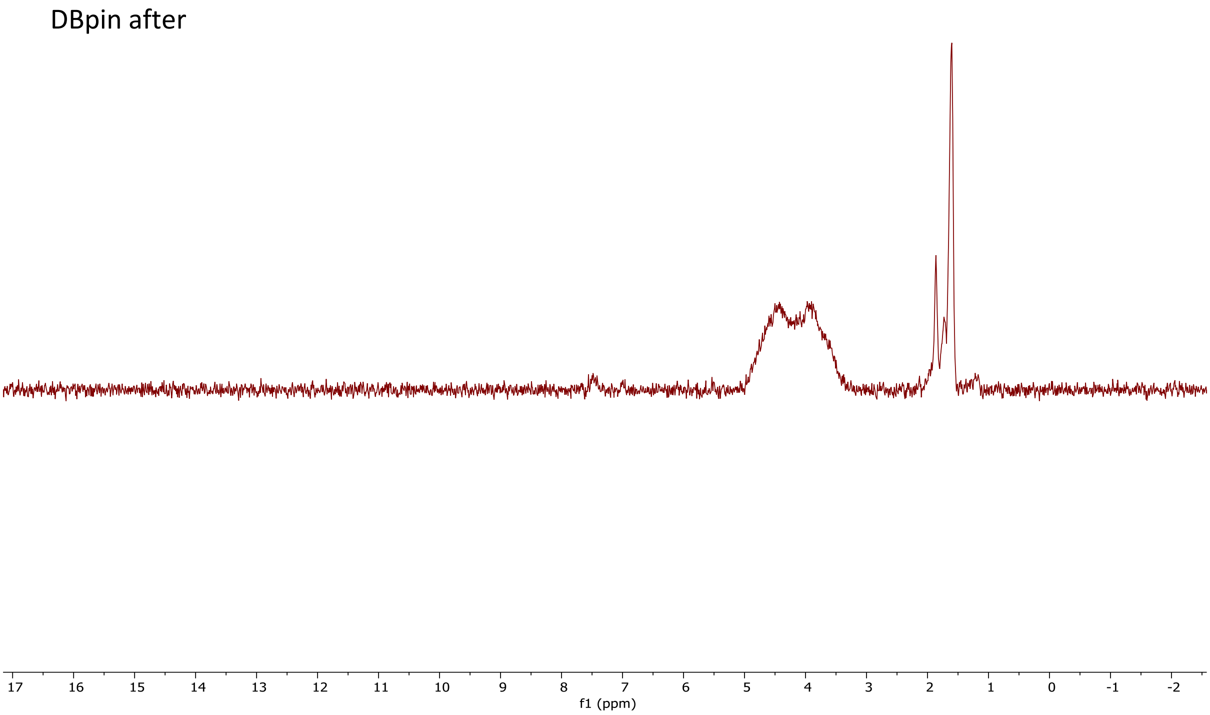

Figure S26. ^{2}D NMR (61.4 MHz, $CHCl_3$) spectrum of 'DBpin' reaction at t = 420 mins.

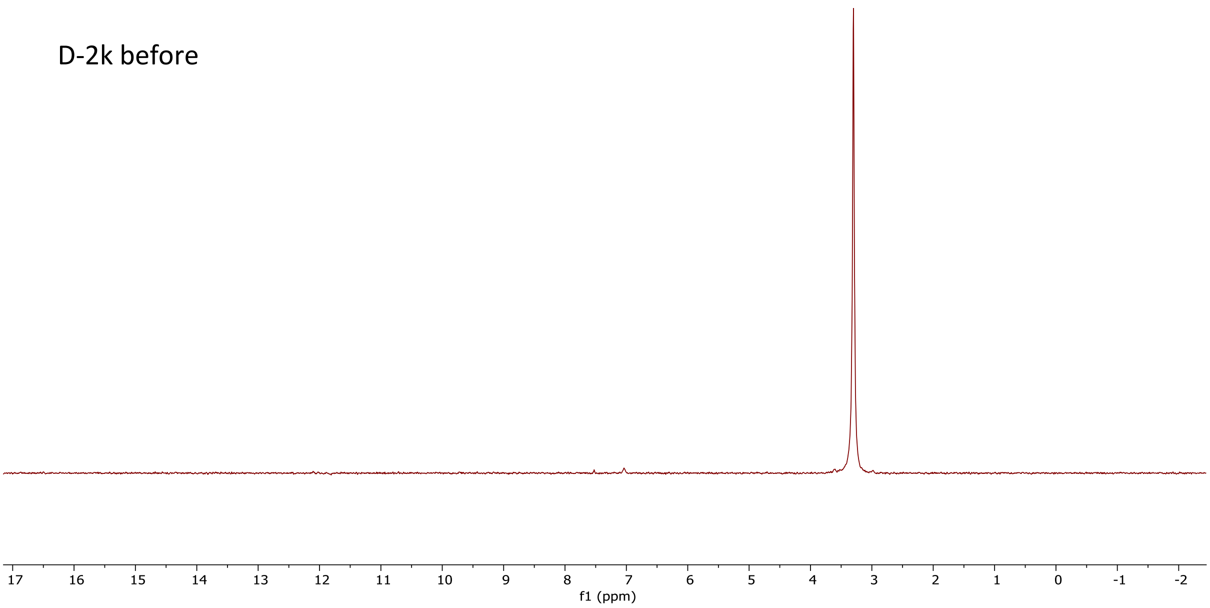

Figure S27. [2]D NMR (61.4 MHz, $CHCl_3$) spectrum of 'D-2k' reaction at *t* = 0.

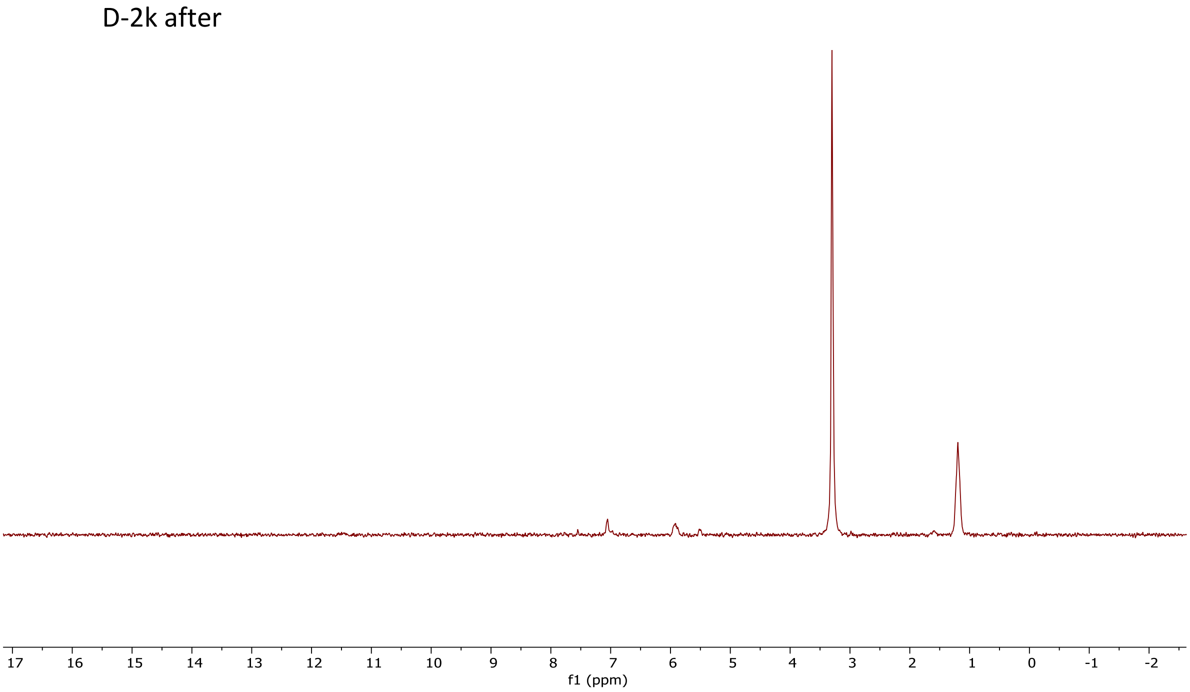

Figure S28. [2]D NMR (61.4 MHz, $CHCl_3$) spectrum of 'D-2k' reaction at *t* = 420 mins.

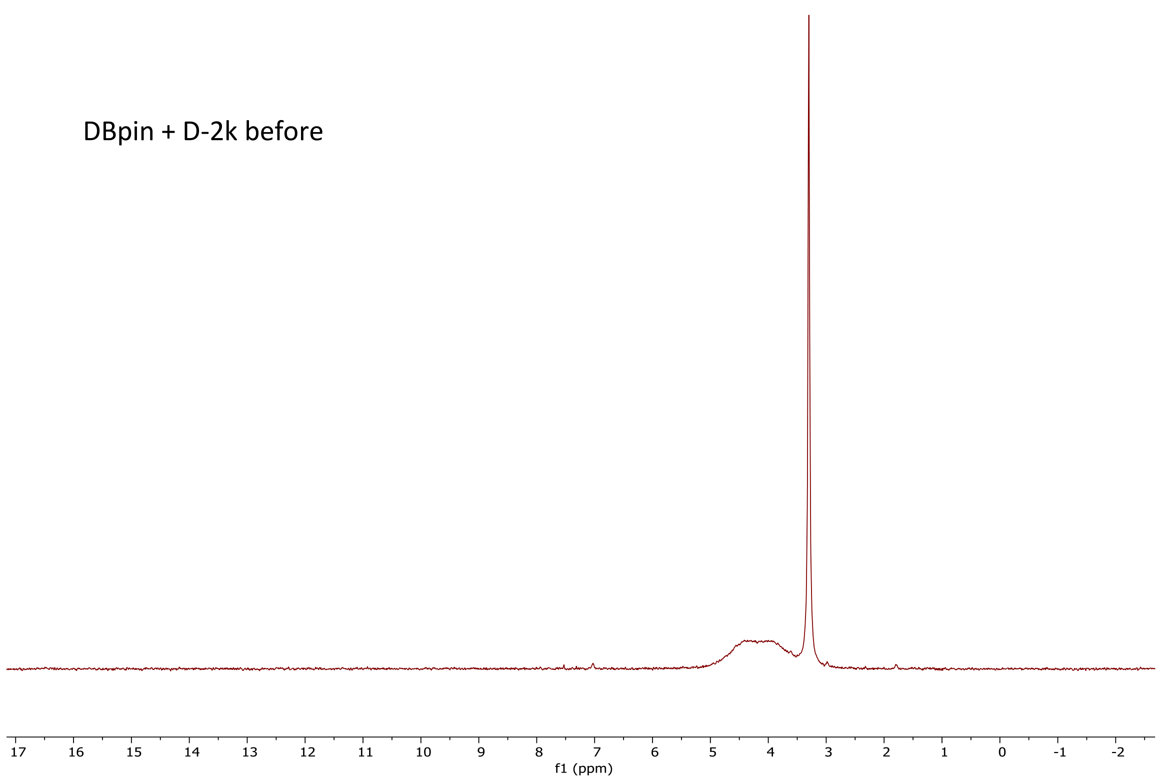

Figure S29. ^{2}D NMR (61.4 MHz, $CHCl_3$) spectrum of 'DBpin+D-2k' reaction at t = 0.

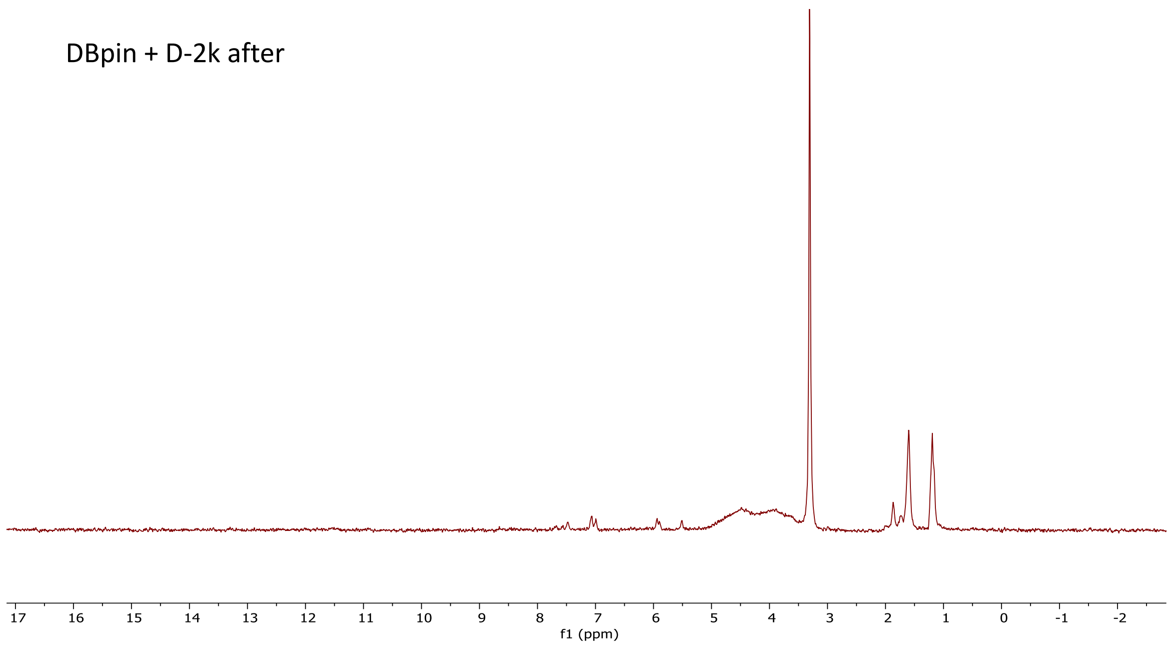

Figure S30. ^{2}D NMR (61.4 MHz, $CHCl_3$) spectrum of 'D-2k' reaction at t = 420 mins.

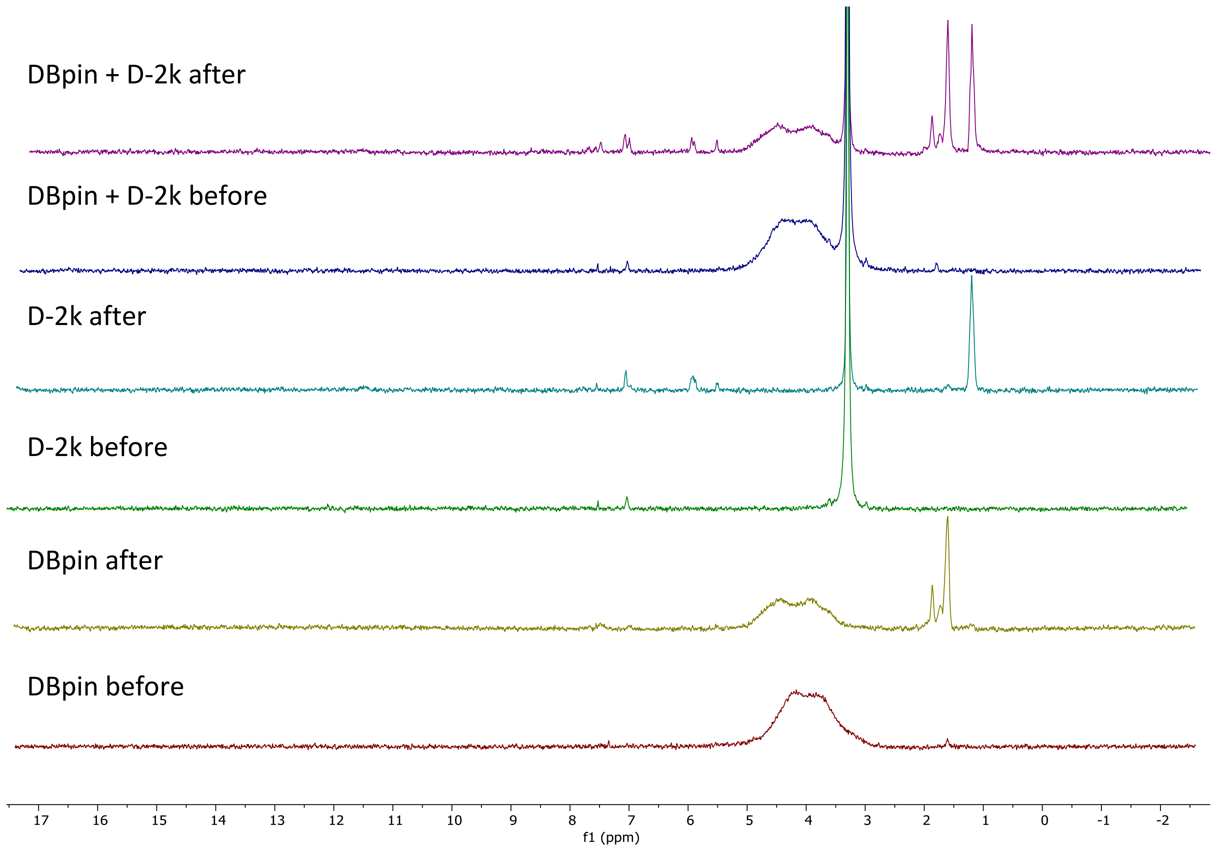

Figure S31. Overlaid ^2D NMR (61.4 MHz, $CHCl_3$) spectra before and after kinetics runs for kinetic isotope effect experiments.

From the overlaid spectra, it is clear that no deuterium scrambling is taking place between 2k and DBpin (and *visa versa*).

8.4. Predicted Primary KIEs

By using the equation (derived from the harmonic ossilator approximation):

$$\frac{k_H}{k_D} = exp\left(\frac{h(\nu_{X-H} - \nu_{X-D})}{2k_B T}\right)$$

Where k_H/k_D is the kinetic isotope effect, h is Planck's constant, $\nu_{X\text{-}H/D}$ is the IR stretching frequency of the bond being broken, k_B is the Boltzmann constant, and T is temperature (293 K).

By using literature values for a dimeric Al–H/D species with bridging hydrides,[16] and our own data for H/DBpin, the KIEs were predicted as follows:

Table S3. Predicted primary KIE values for B–H and Al–H bonds.

Compounds	$\nu_{X\text{-}H}$ / cm^{-1}	$\nu_{X\text{-}D}$ / cm^{-1}	k_H/k_D
H/DBpin	2580	1941	4.70
$Me_2Al(H/D)_2AlMe_2$	1215	905	2.08

8.5. Variable Temperature Experiments on HBpin-1b Adduct

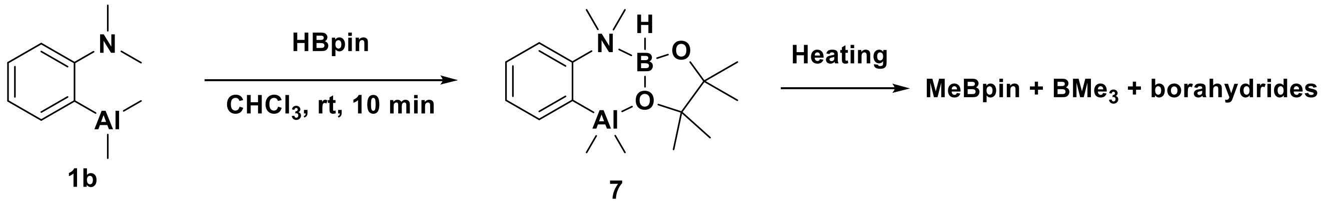

The HBpin-**1b** adduct **7** was prepared *in-situ* as described in the experimental section. This sample was heated for approximately 10 minutes at the stated temperature between each acquisition. By conversion in the ^{11}B NMR spectrum, HBpin content remained at approximately 40%, whereas the adduct disappeared and MeBpin appeared. This suggests that MeBpin production is from the adduct.

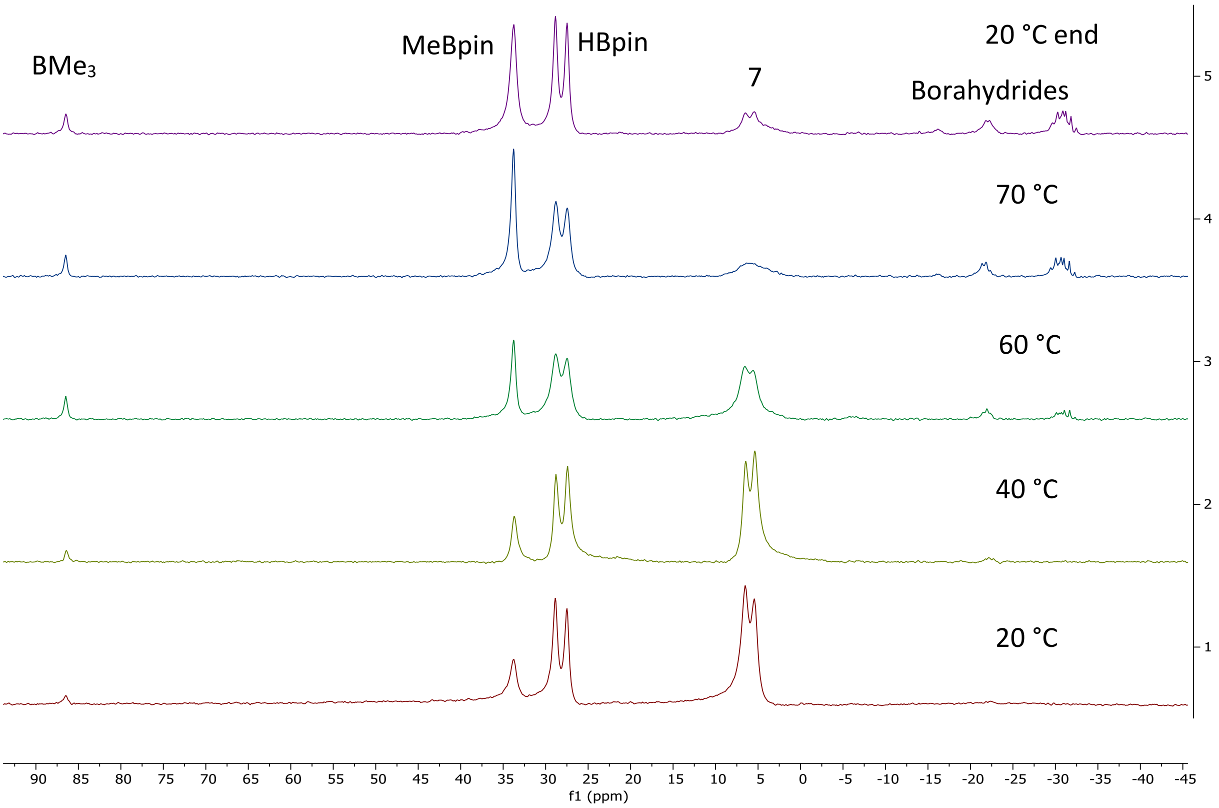

Figure S32. Overlaid ^{11}B NMR (160.46 MHz, $CDCl_3$) spectra of *in-situ*-prepared adduct **7** at 20 – 70°C, followed by another spectrum at 20 °C afterwards ('20 °C end').

8.6. Catalytic Competence of Alane 8

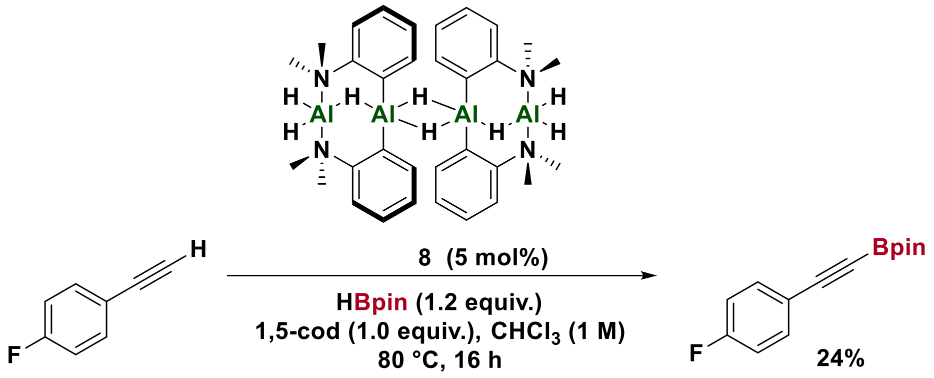

Under an inert atmosphere, bis(*N,N*-dimethylaniline)methylaluminium-alane adduct (0.025 mmol, 14 mg) and HBpin (0.600 mmol, 87.0 µL) were dissolved in chloroform (0.500 mL). 1,5-Cyclooctadiene (0.500 mmol, 61.0 µL) followed by 4'-fluorophenylacetylene (0.50 mmol, 57 µL) was added and the mixture heated at 80 °C for 16 hours. NMR yields were calculated by ^{19}F NMR spectroscopy (fluorobenzene as an internal standard) of the crude reaction mixture. By ^{19}F NMR spectroscopy, there was 24% of the C–H borylation product, indicating that the catalyst achieved turnover.

8.7. Al/B exchange in tris-acetylide 6

Al/B Exchange

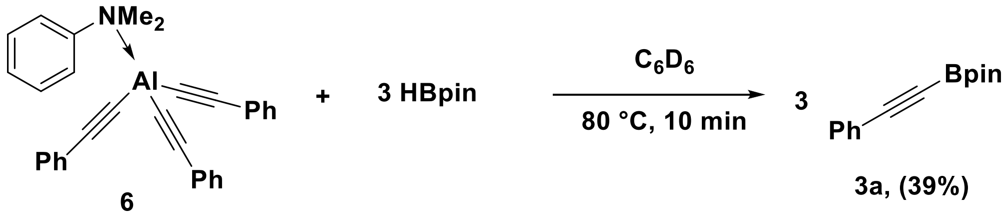

In an NMR tube with a J-Young tap fitting, aluminium trisacetylide **6** (0.100 mmol, 45.0 mg) was dissolved in C_6D_6 (0.500 mL). HBpin (0.300 mmol, 44.0 µL) was added, the tube was sealed and heated at 80 °C for 10 minutes, then ^1H and ^{11}B NMR spectroscopy were performed. The yield of C–H borylation product was determined by comparison to the *N,N*-dimethylaniline signal to be 39%.

^1H NMR (C_6D_6, 500.13 MHz):

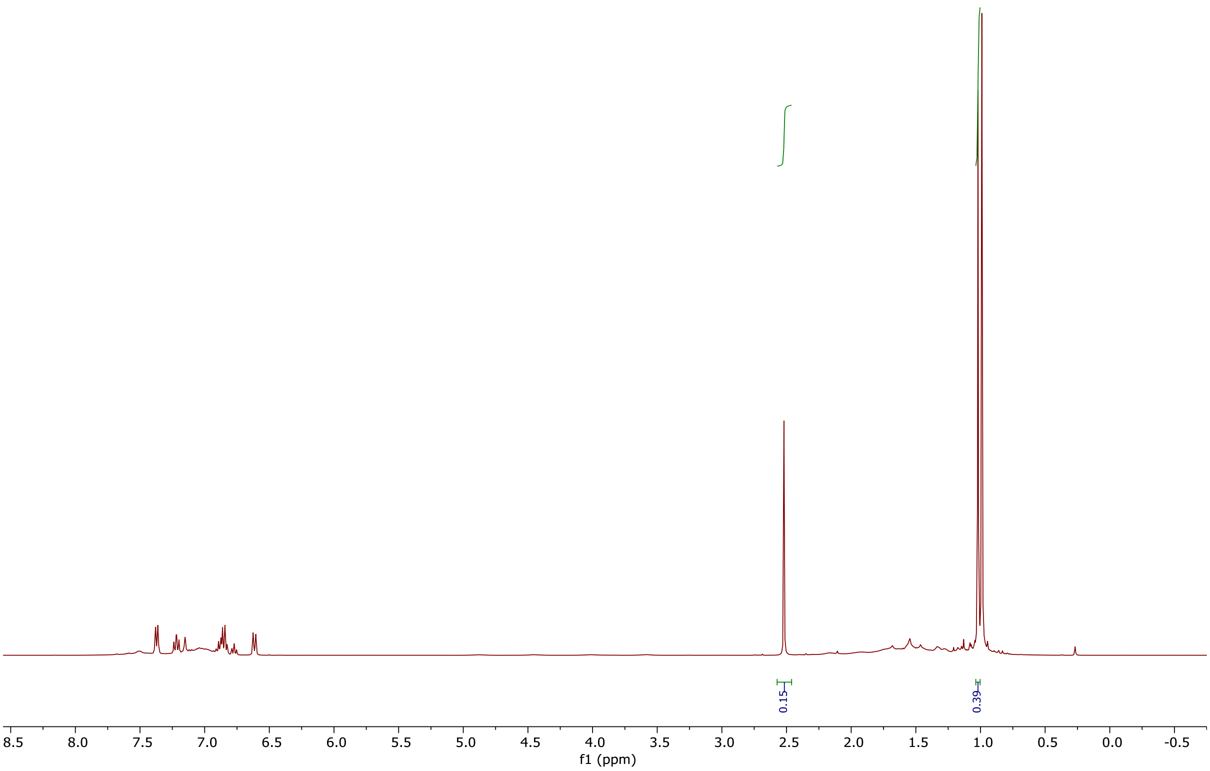

^{11}B NMR (C_6D_6, 160.46 MHz):

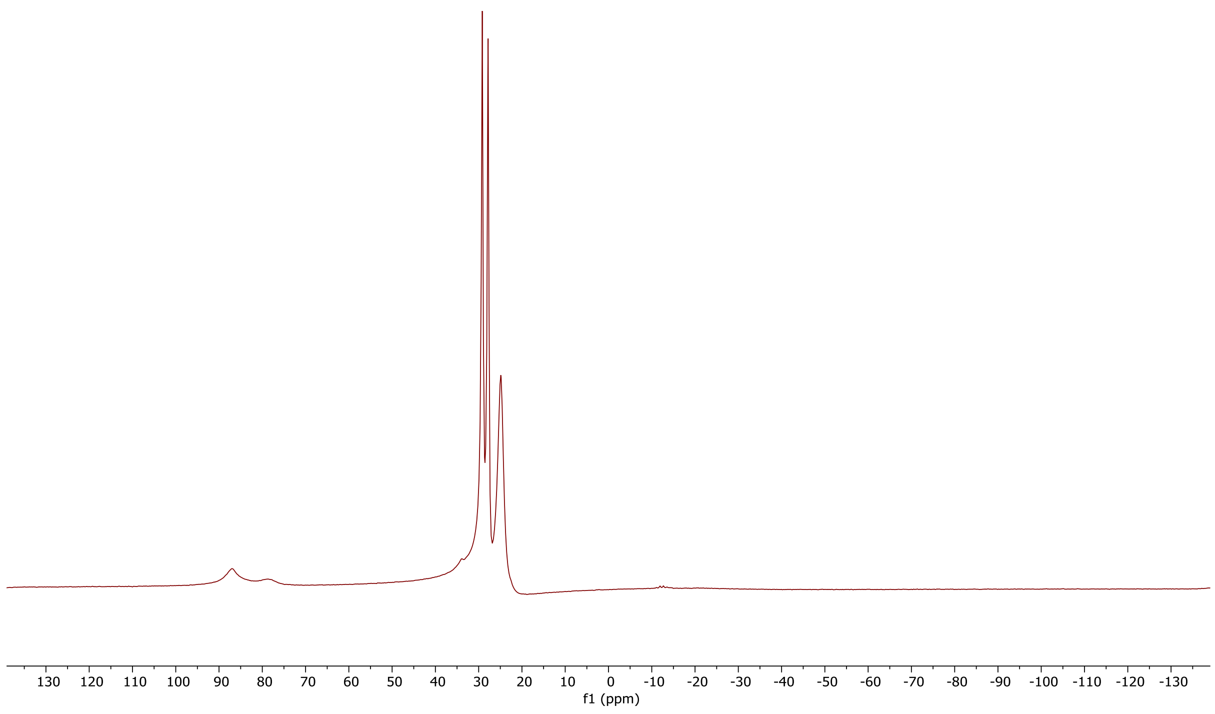

8.8. Catalytic Competence of Tris-Acetylide 6

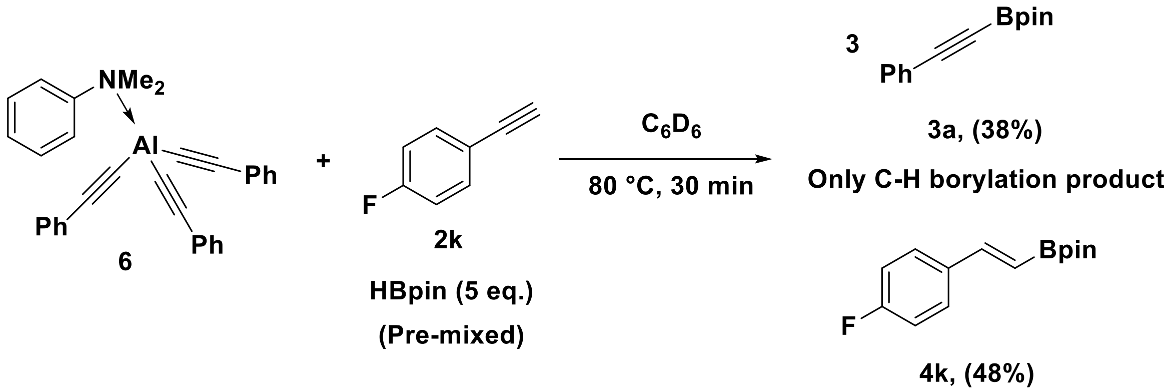

In an NMR tube with a J-Young tap fitting, aluminium trisacetylide (0.100 mmol, 45.0 mg) was dissolved in C_6D_6 (0.250 mL). HBpin (0.500 mmol, 73.0 µL) and 4'-fluorophenylacetylene (0.100 mmol, 12.0 µL) were dissolved in C_6D_6 (0.250 mL), then added, the tube was sealed and heated at 80 °C for 30 minutes, then ^{1}H and ^{11}B NMR spectroscopy were performed. The yield of C–H borylation product was determined by comparison to the *N,N*-dimethylaniline signal to be 38%. There was no C–H borylation of 4'-fluorophenylacetylene, although significant hydroboration (48%) was observed.

^{1}H NMR (C_6D_6, 500.13 MHz):

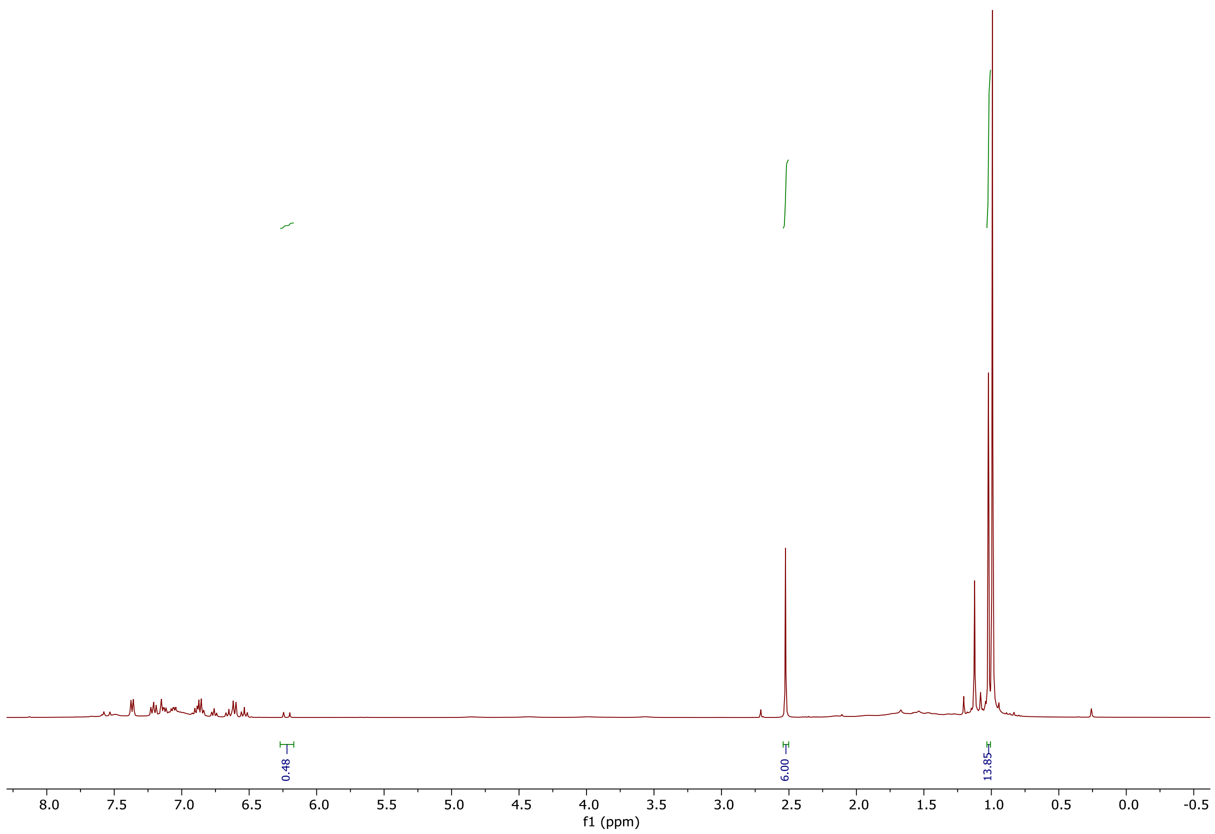

^{11}B NMR (C_6D_6, 160.46 MHz):

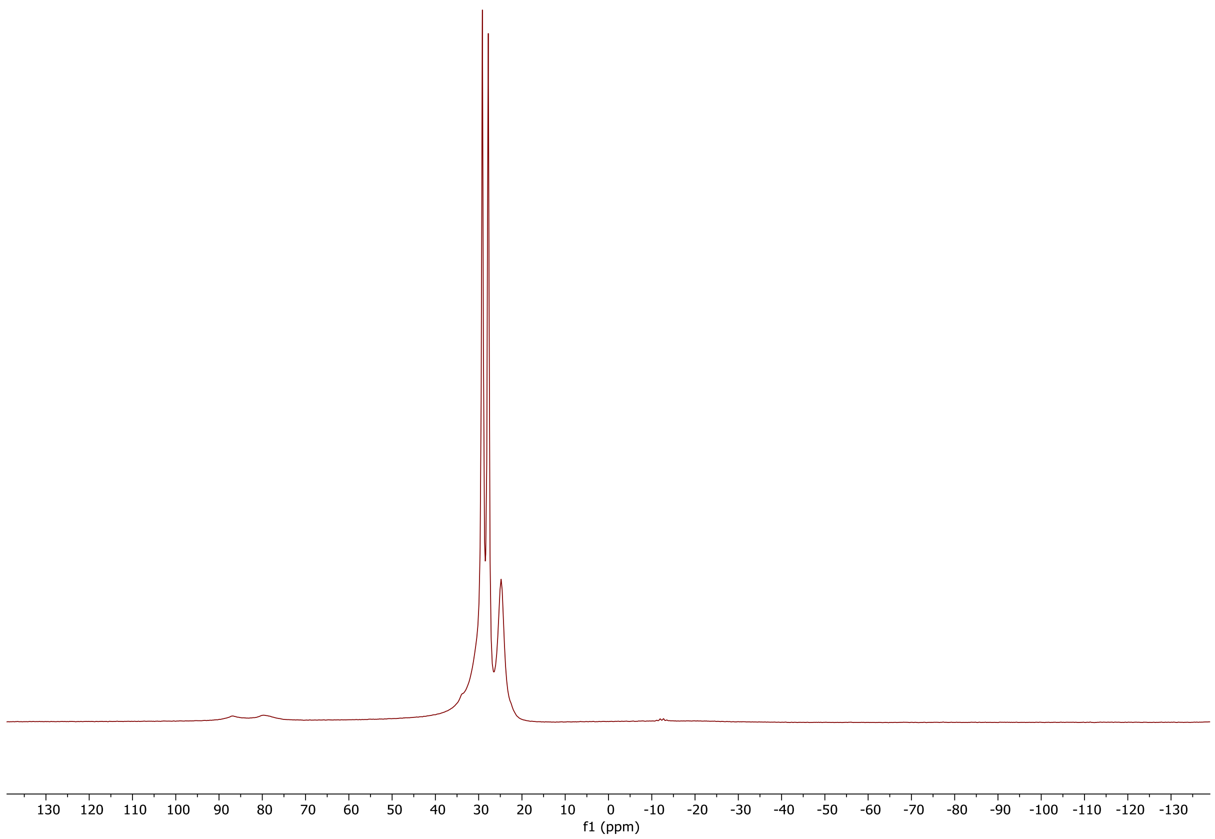

^{19}F NMR (C_6D_6, 470.59 MHz):

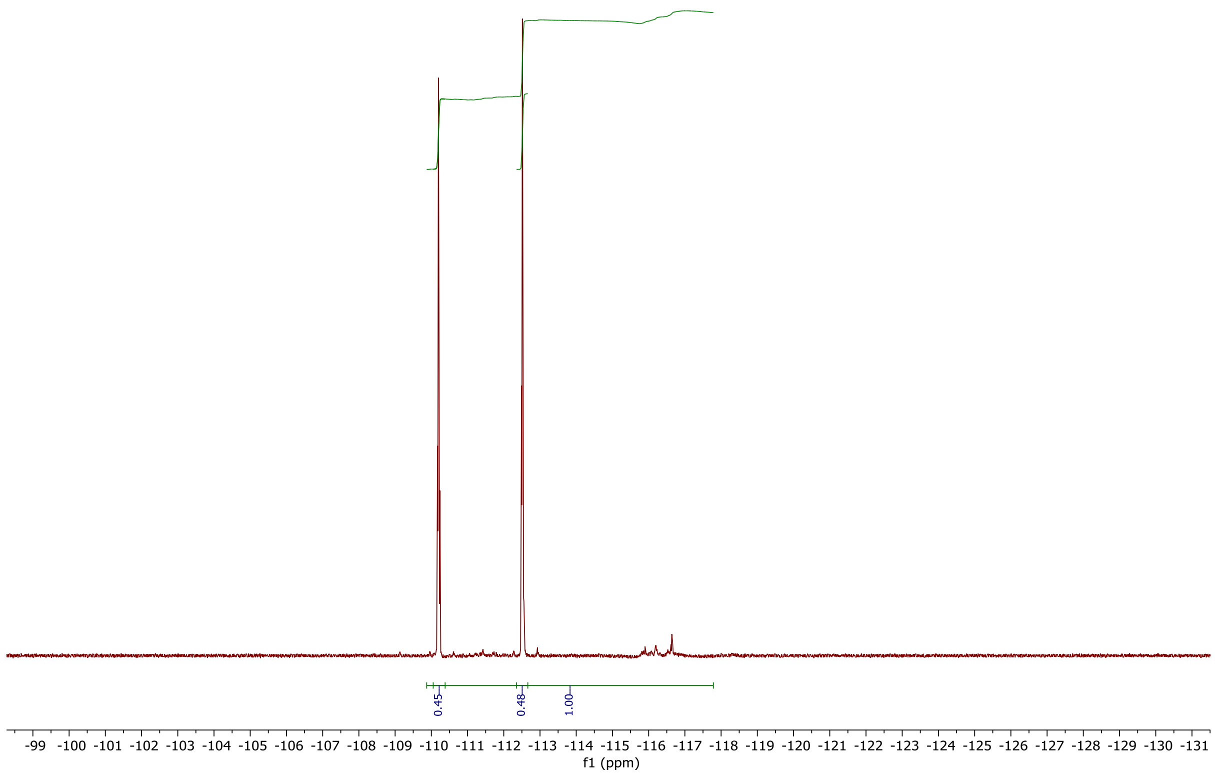

9. Computational Details

9.1. Computational Details

Geometry optimizations and harmonic frequency calculations were performed using the Gaussian16 program package[17] employing the ωb97XD density functional[18] in combination with the 6-31+G(d,p) basis set using standard convergence criteria. The SMD polarizable continuum model[19] was employed to account for solvent effects (Chloroform). Stationary points were characterized as minima or 1st order transition states by analysis of the frequency calculations with zero or one imaginary frequency. Single-point calculations were conducted on the optimized geometries using the ωb97XD functional in combination with the higher level 6-311++G(d,p) basis set. Unscaled zero-point vibrational, thermal and entropic corrections were obtained from the frequency calculations at the ωb97XD/6-31+G(d,p) level using general procedures to obtain free energy corrections at standard conditions (T = 298.15 K, p = 1 atm) and combined with internal energy from the 6-311++G(d,p) higher level calculation to give Gibbs free energy values at greater accuracy with lower computational cost of geometry Optimization. Free energy reaction schemes are presented relative to the free energies of the starting material. Pictures of molecular structures were generated with the ChemCraft[20] program.

9.2. Energy Profile for Terminal Alumination

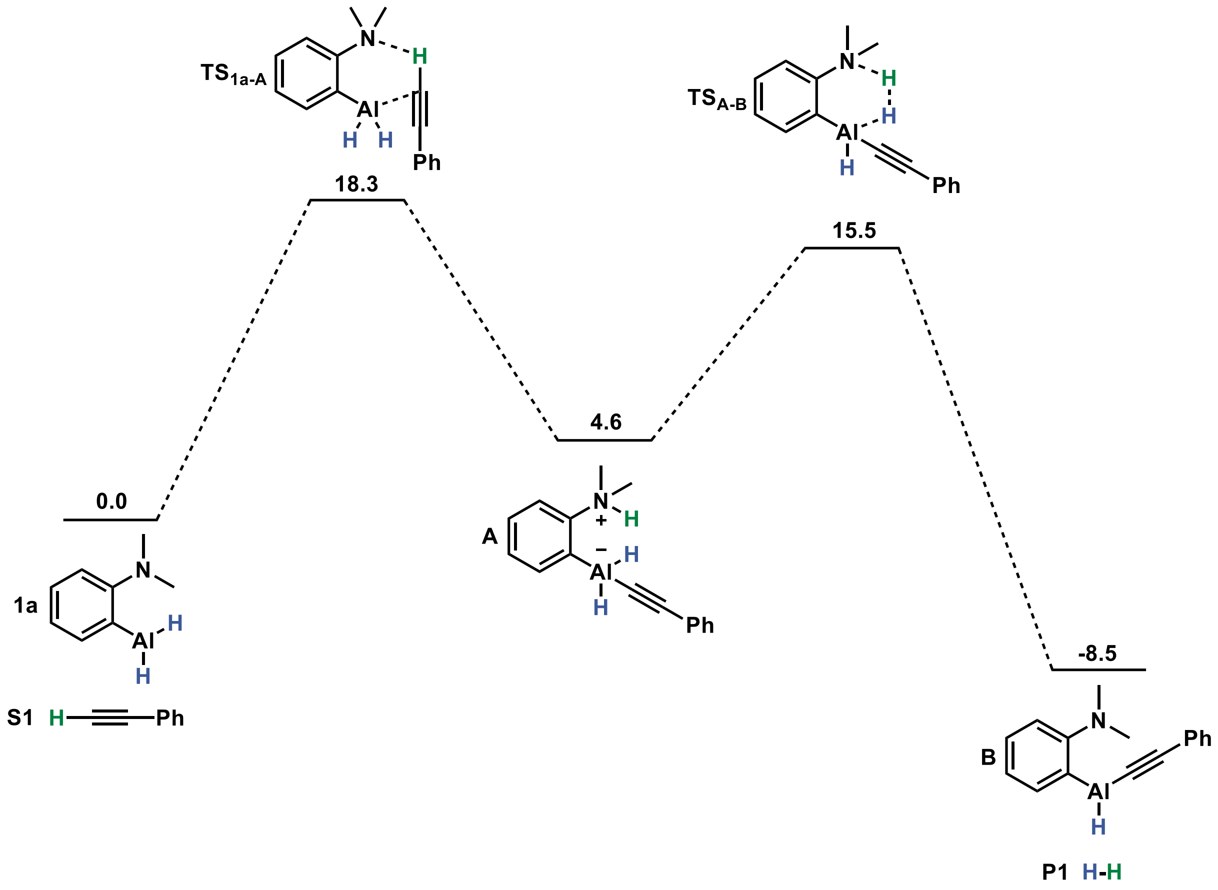

Figure S33. Free energy profile (kcal mol^{-1}) for C–H alumination of phenylacetylene, starting from 2-aluminiumdihydride-*N,N*-dimethylaniline **1a**.

9.3. Energy Profile for Hydroboration

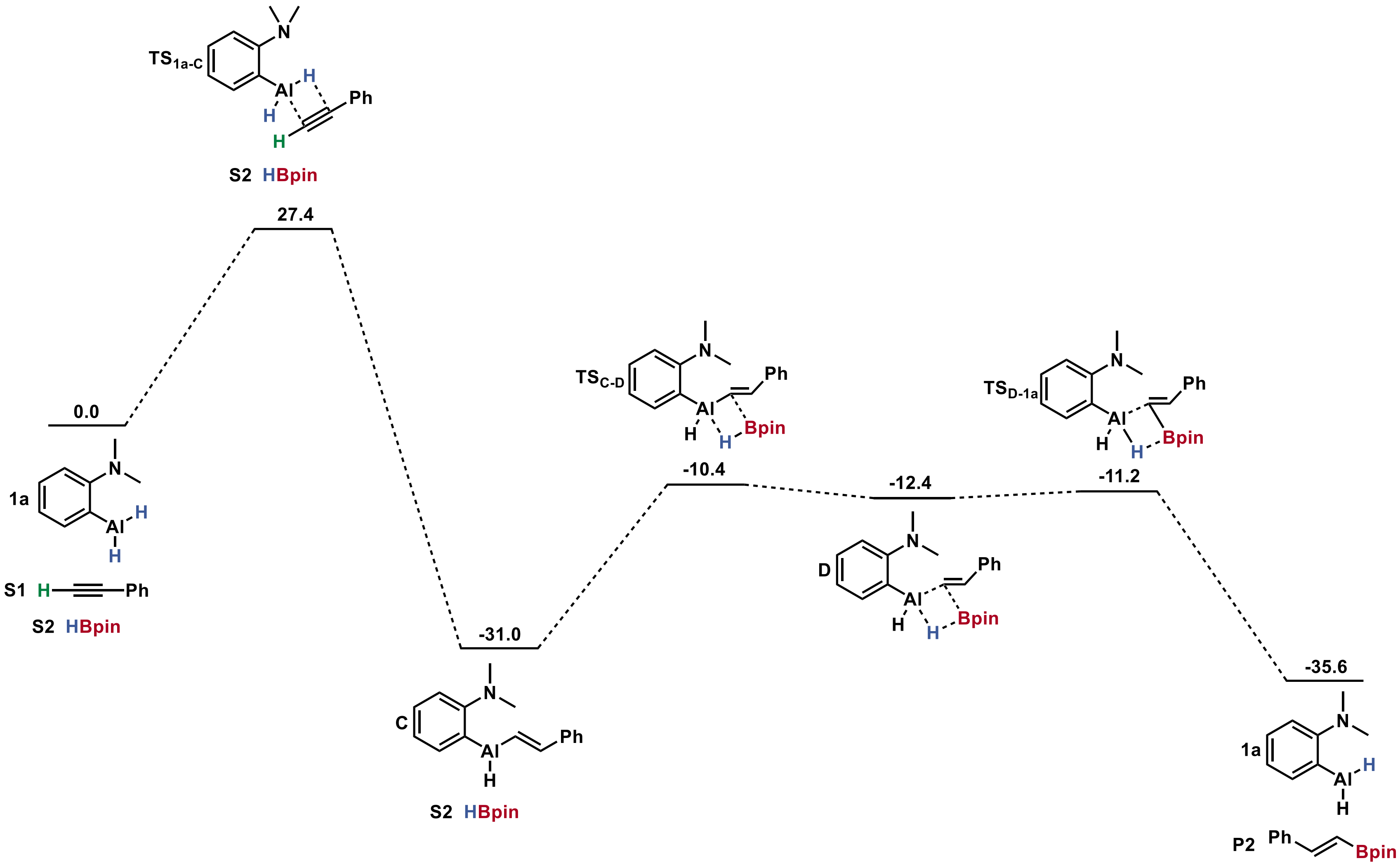

Figure S34. Free energy profile (kcal mol^{-1}) for hydroboration of phenylacetylene, starting from 2-aluminiumdihydride-*N,N*-dimethylaniline **1a**..

9.4. Catalyst Activation Pathways

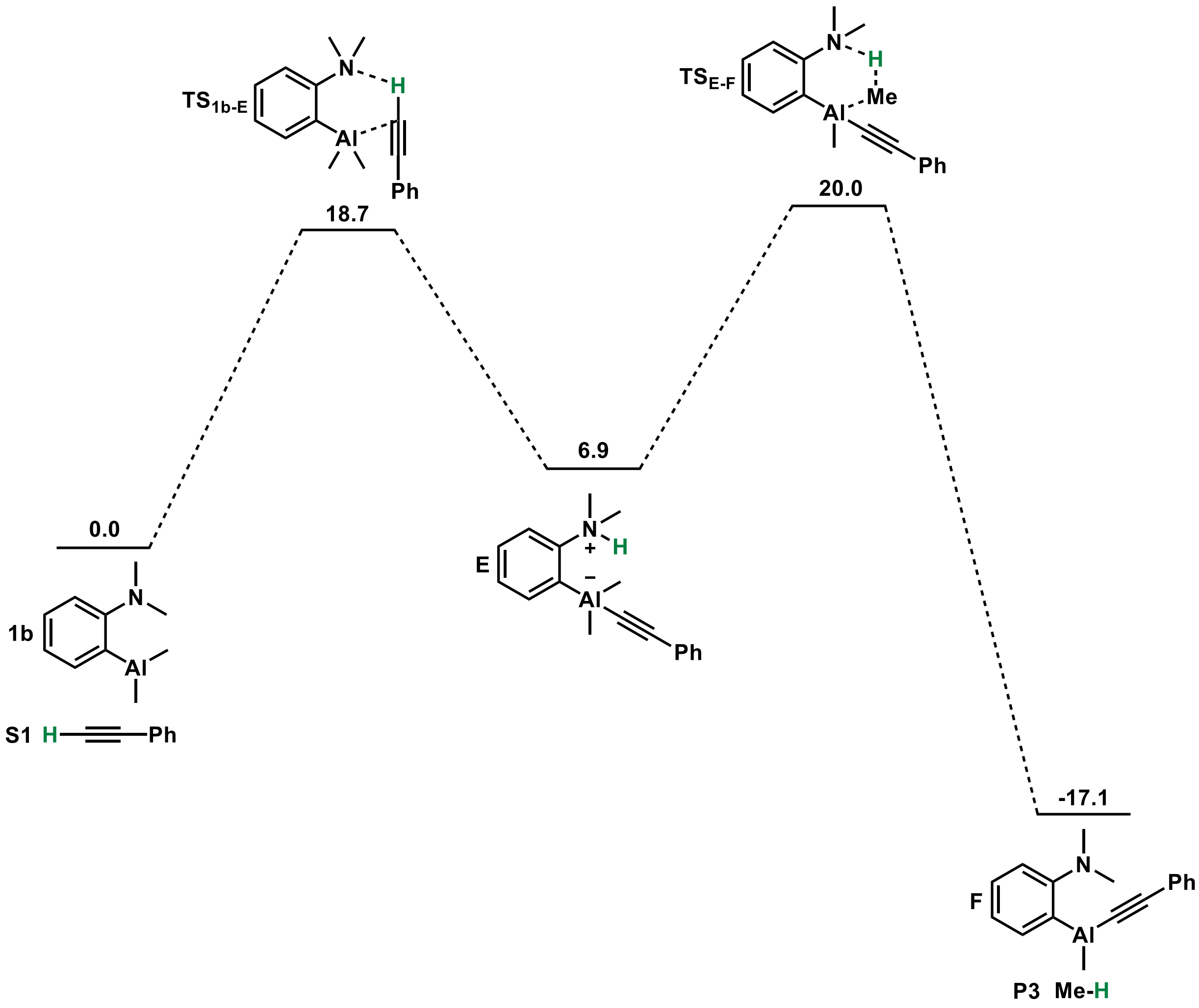

Figure S35. Free energy profile (kcal mol^{-1}) of catalyst activation of 2-dimethylaluminium-*N,N*-dimethylaniline **1b** with phenylacetylene.

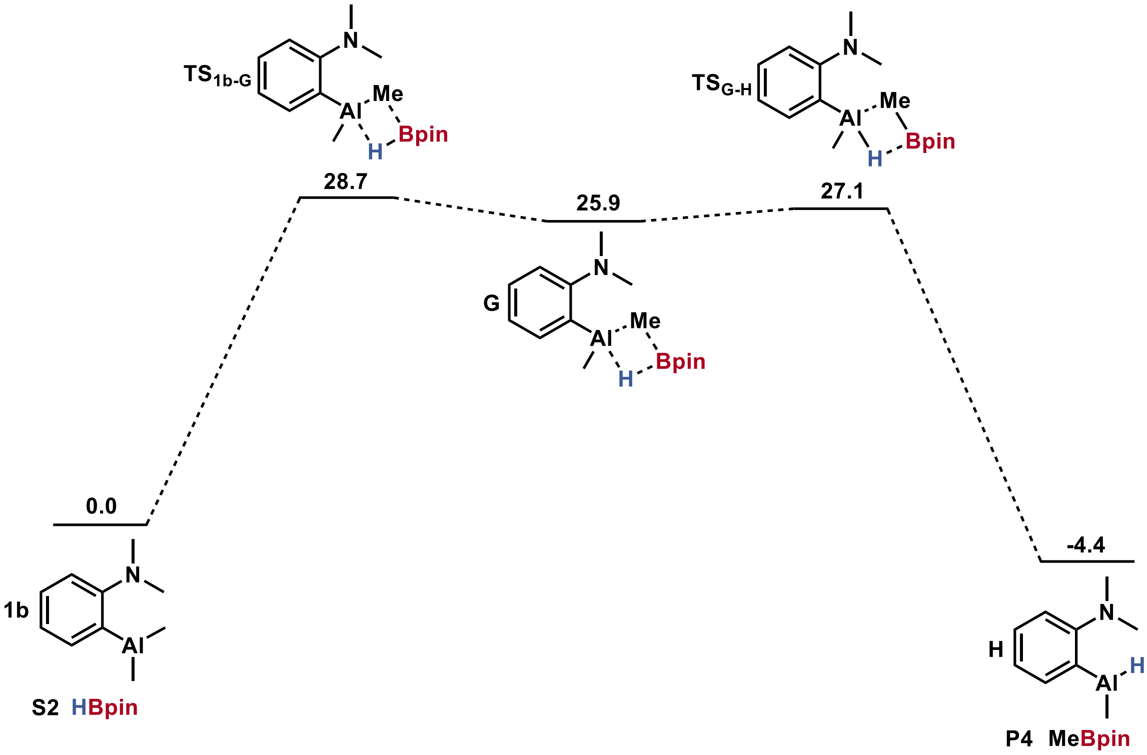

Figure S36. Free energy profile (kcal mol^{-1}) of catalyst activation of 2-dimethylaluminium-*N*,*N*-dimethylaniline **1b** with HBpin.

9.5. Optimized Structures of Aluminium Dihydride Monomers, Dimers and Oligomers

$[1a]_2$ 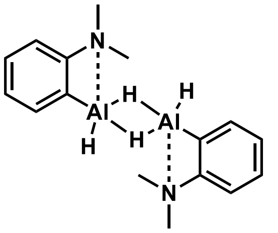

1a 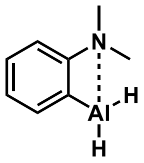

-1.5 0

Transition State to oligomers:

$TS_{1a\text{-}I}$ 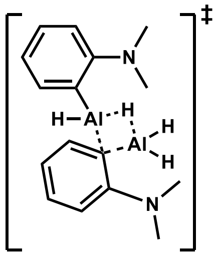

17.8

Oligomers:

8 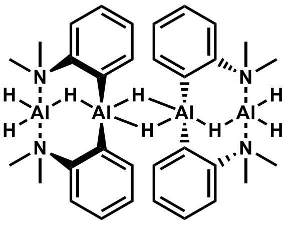

$[anilino]_2AlH{\cdot}AlH_3$ 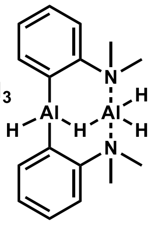

I 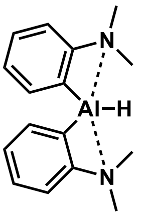

AlH_3

-2.9 -4.4 14.3

Figure S37. Free energies (kcal mol^{-1}) of oligomers of 2-aluminiumdihydride-*N,N*-dimethylaniline **1a**.

9.6. Cartesian Coordinates for Quantum Chemical Calculations

A zip folder containing all .xyz files for the final structures from the calculations can be found attached numbered as in the schemes included above in the SI.

10. NMR Spectra of Isolated Compounds

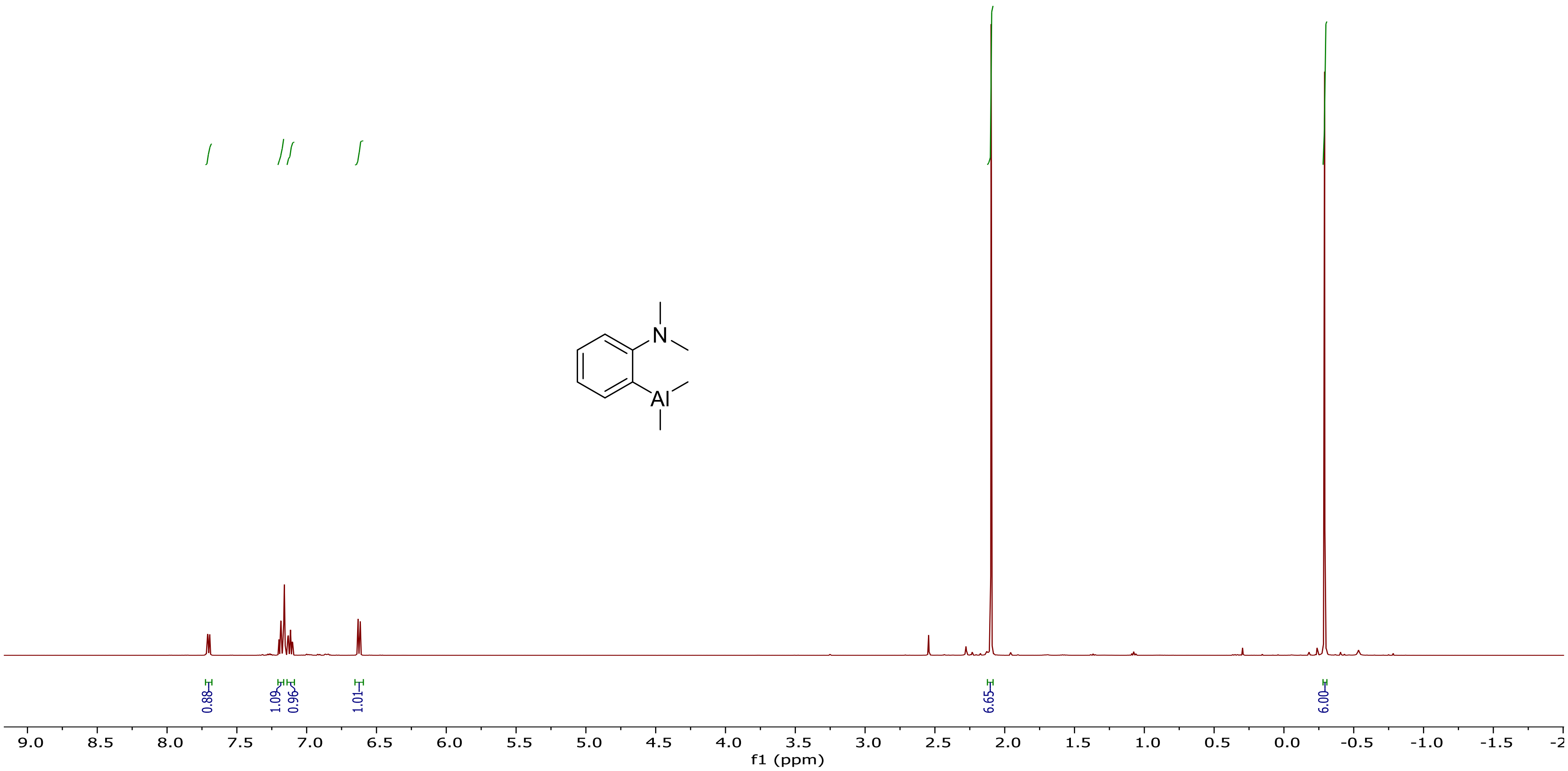

^{1}H NMR (C_6D_6, 500.12 MHz) of dimethylaluminium-*N*,*N*-dimethylaniline.

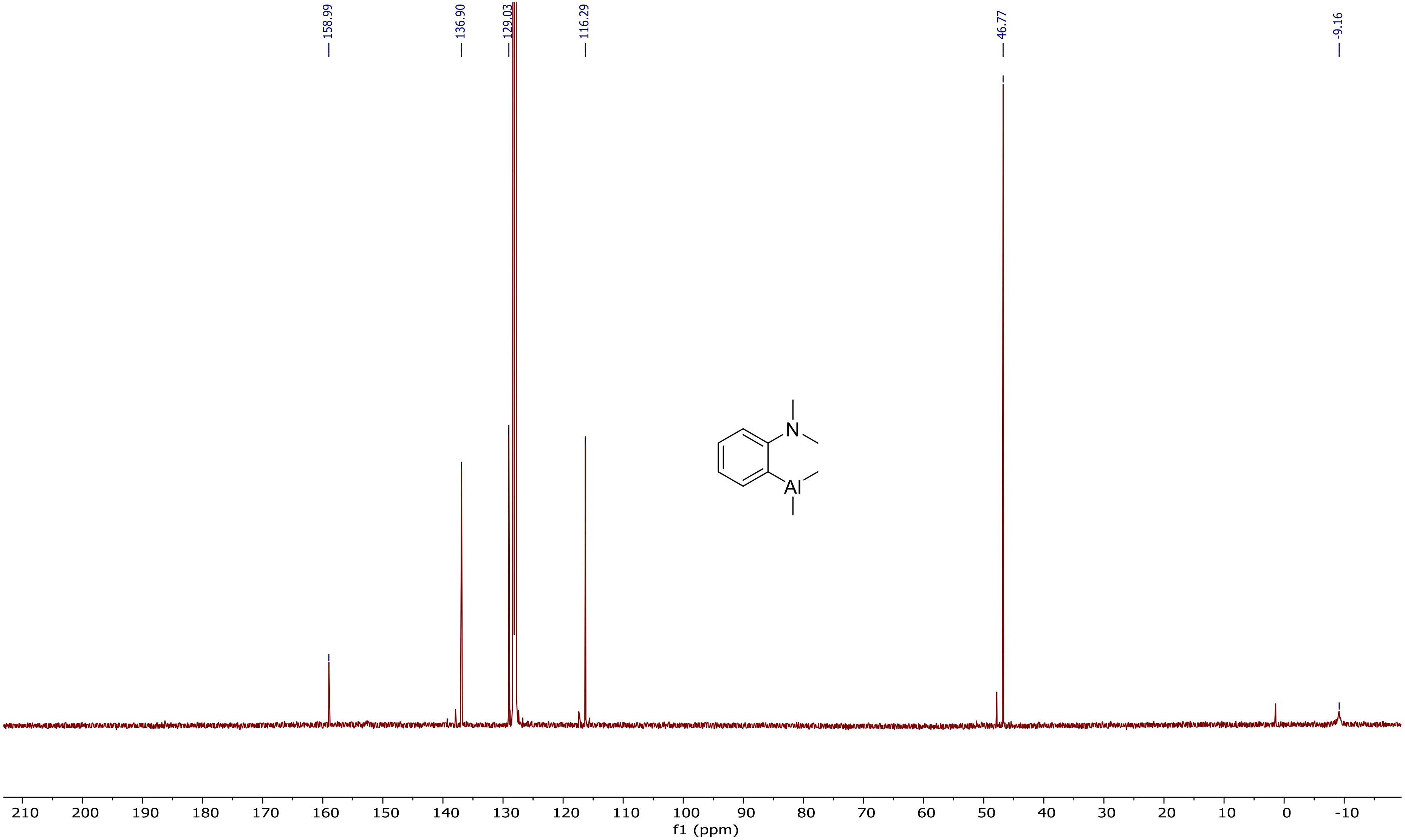

^{13}C NMR (C_6D_6, 125.77 MHz) of dimethylaluminium-*N,N*-dimethylaniline.

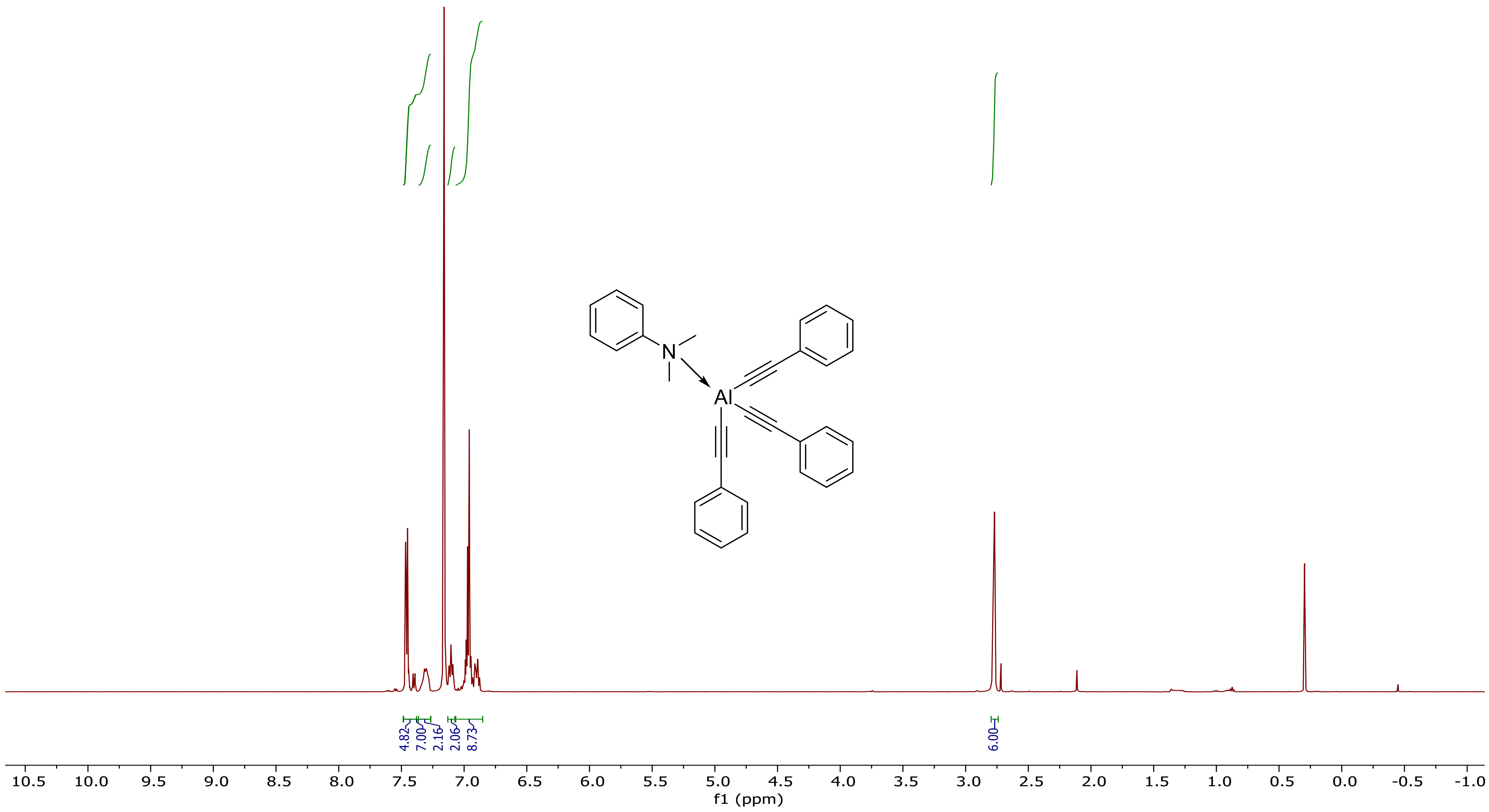

^{1}H NMR (C_6D_6, 500.12 MHz) of tris(phenylacetyl)aluminium-N,N-dimethylaniline adduct.

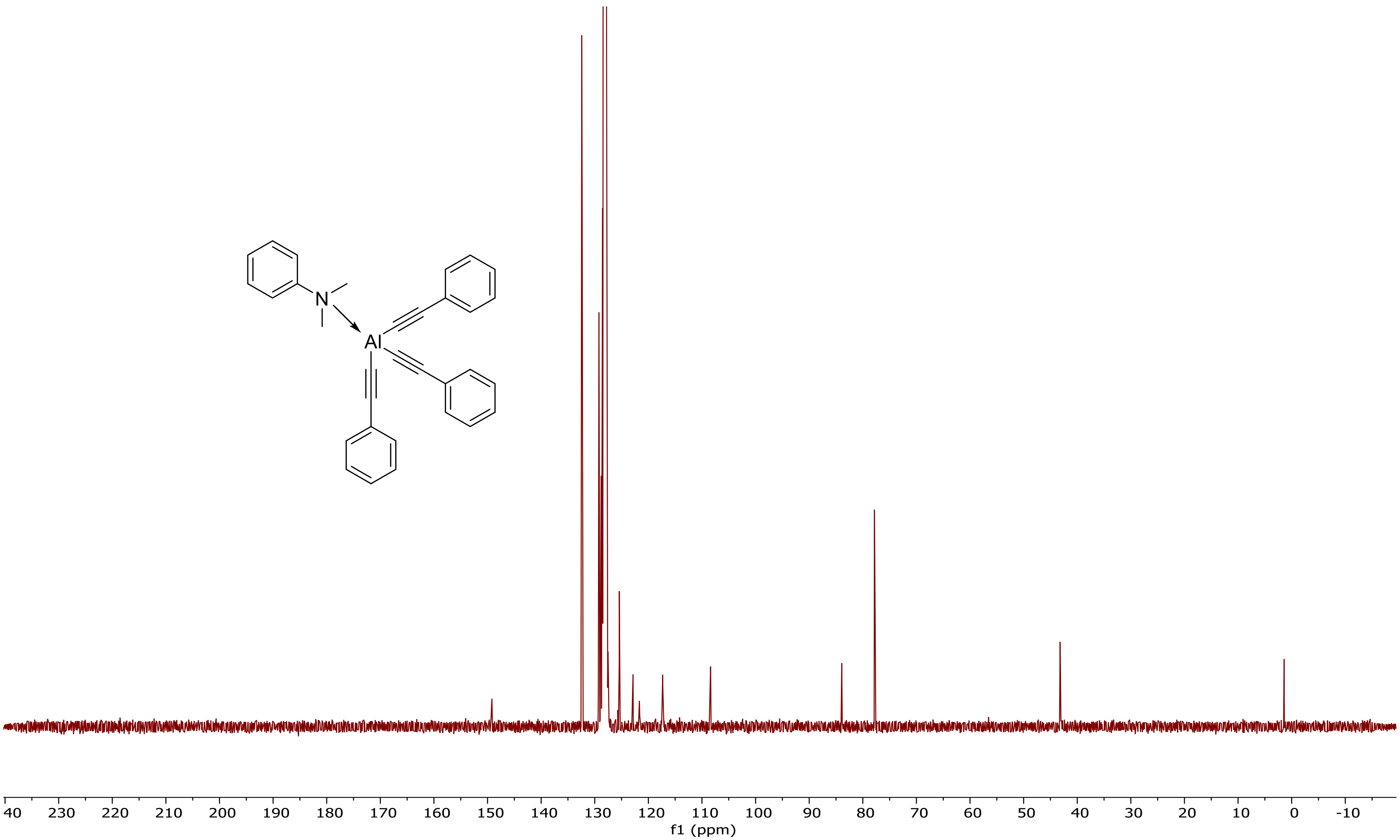

^{13}C NMR (C_6D_6, 125.77 MHz) of tris(phenylacetyl)aluminium-N,N-dimethylaniline adduct.

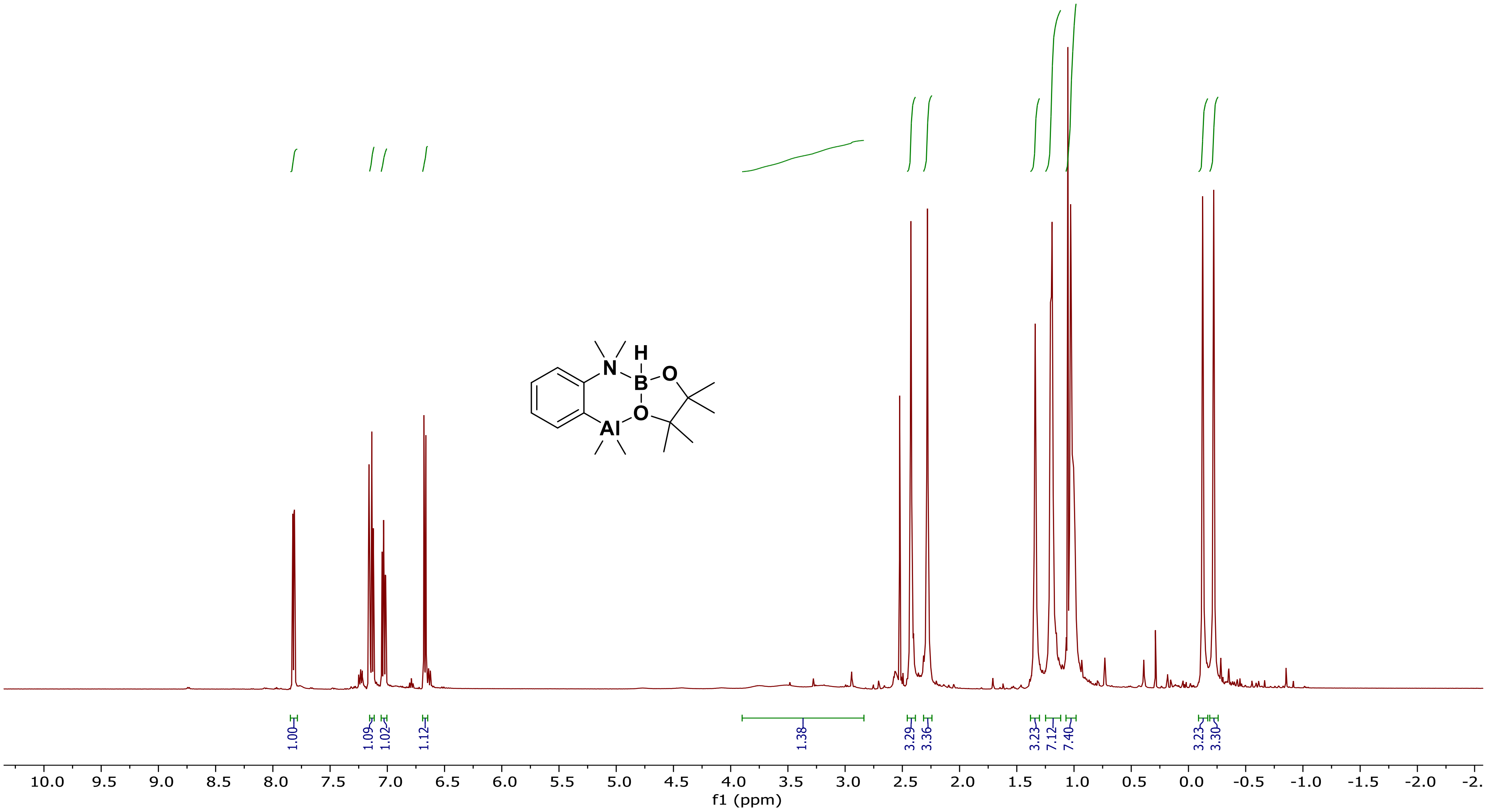

^{1}H NMR (C_6D_6, 500.12 MHz) of 2-dimethylaluminium-*N,N*-dimethylaniline-HBpin adduct (crude).

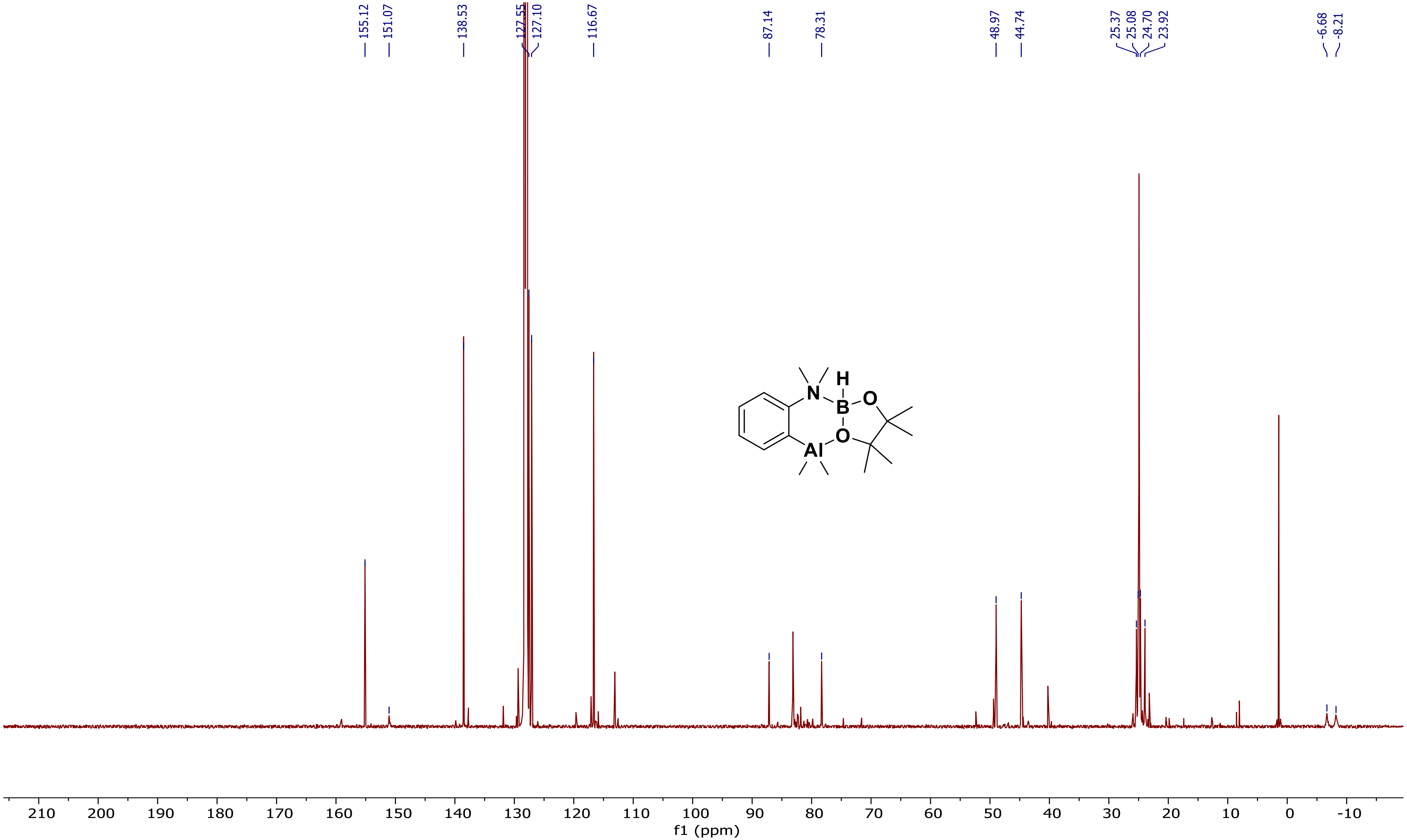

^{13}C NMR (C_6D_6, 125.77 MHz) of 2-dimethylaluminium-*N*,*N*-dimethylaniline-HBpin adduct (crude).

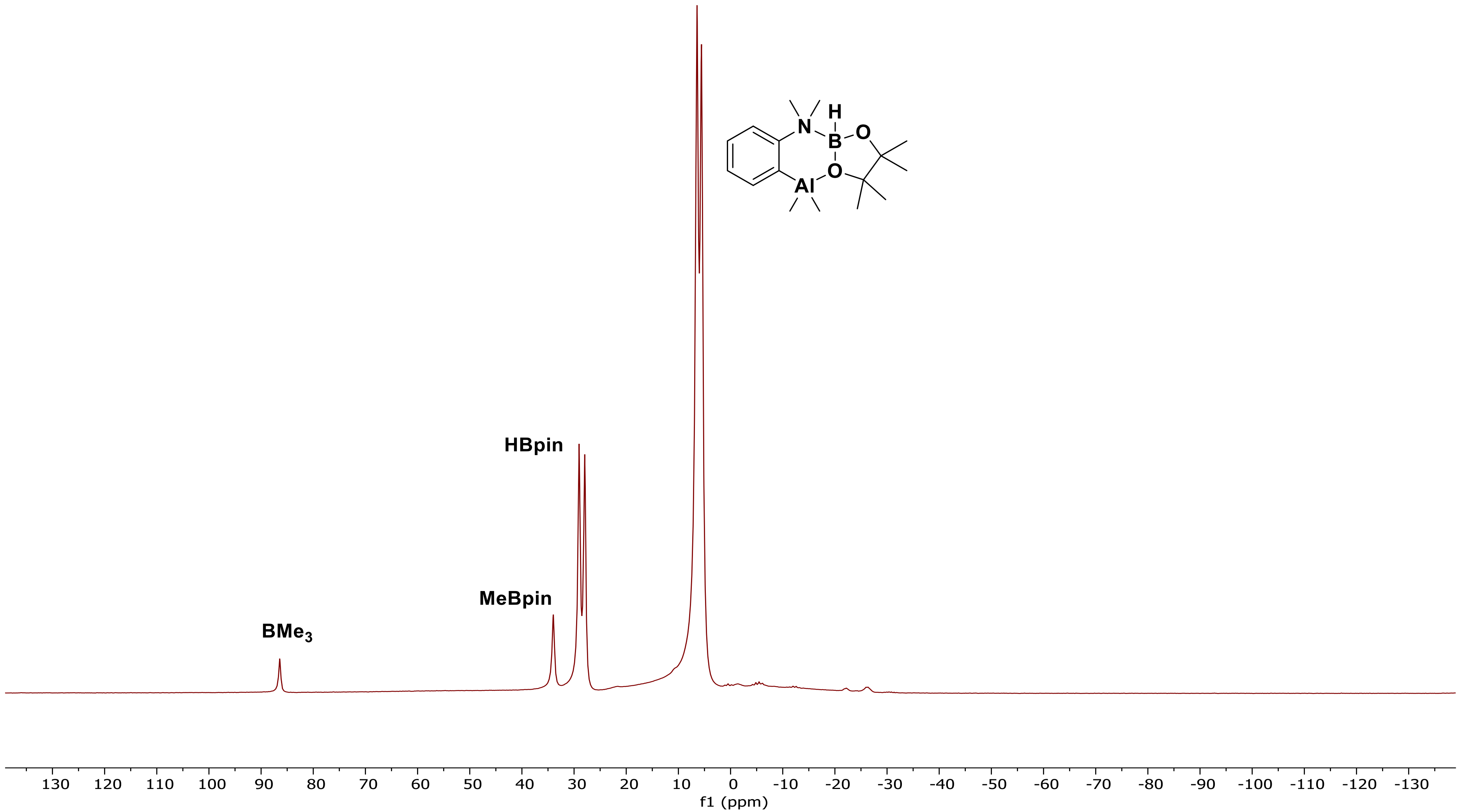

^{11}B NMR (C_6D_6, 128.34 MHz) of 2-dimethylaluminium-*N,N*-dimethylaniline-HBpin adduct, with impurities labelled (crude).

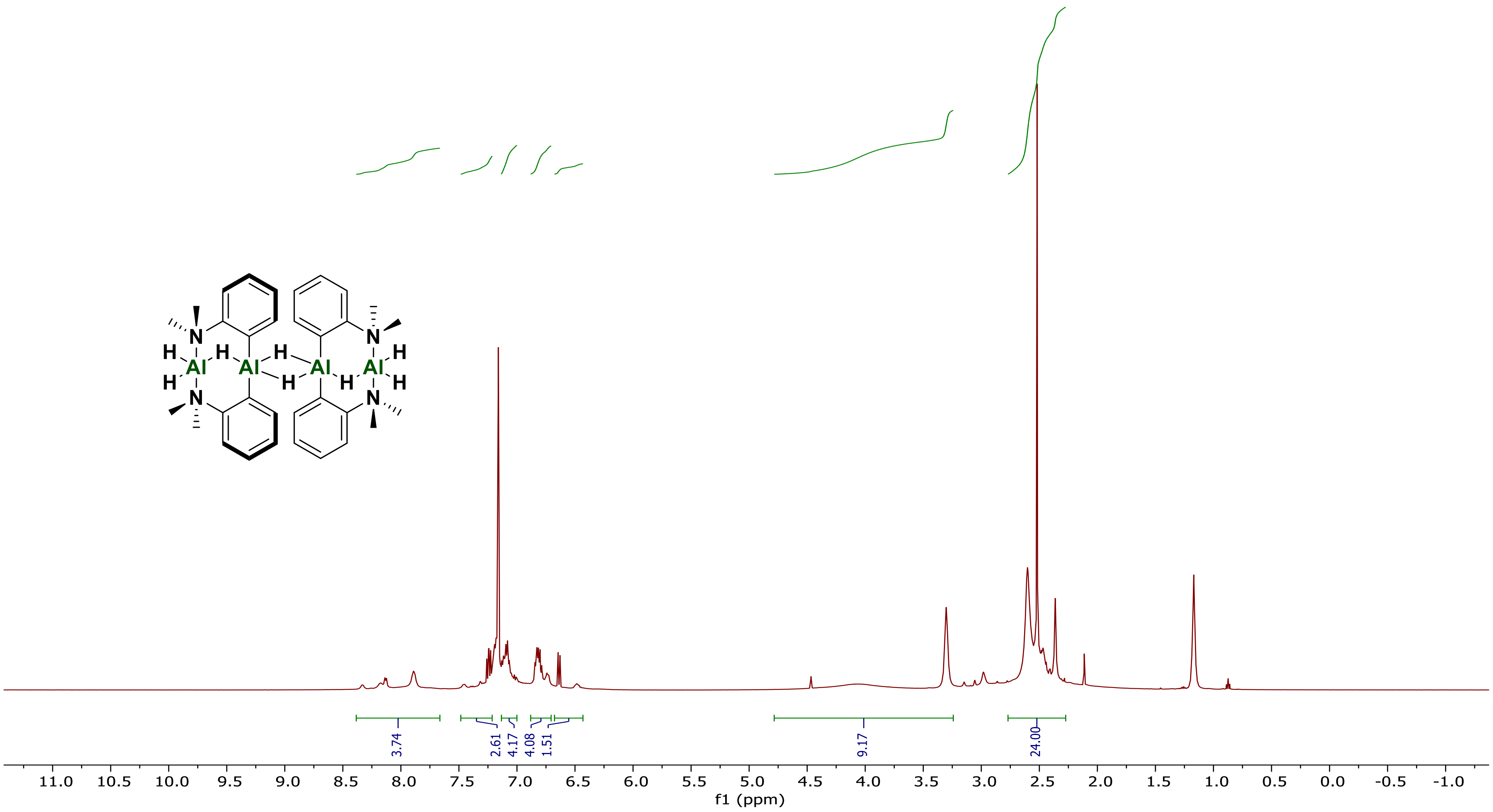

^{1}H NMR (C_6D_6, 500.12 MHz) of bis-{(di-2-*N*,*N*-dimethylaniline aluminium hydride)-alane adduct}.

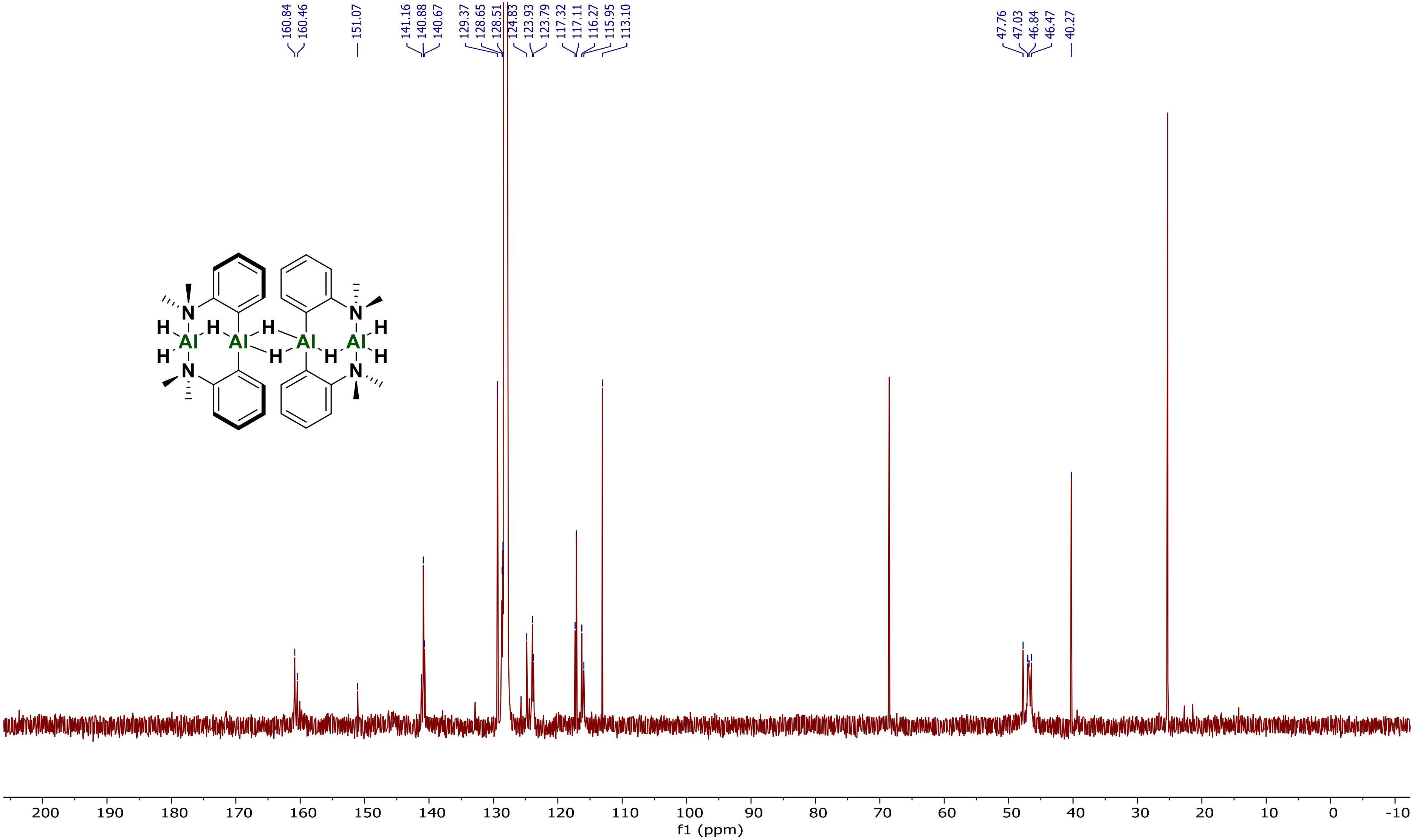

^{13}C NMR (C_6D_6, 125.77 MHz) of bis-{(di-2-*N*,*N*-dimethylaniline aluminium hydride)-alane adduct}.

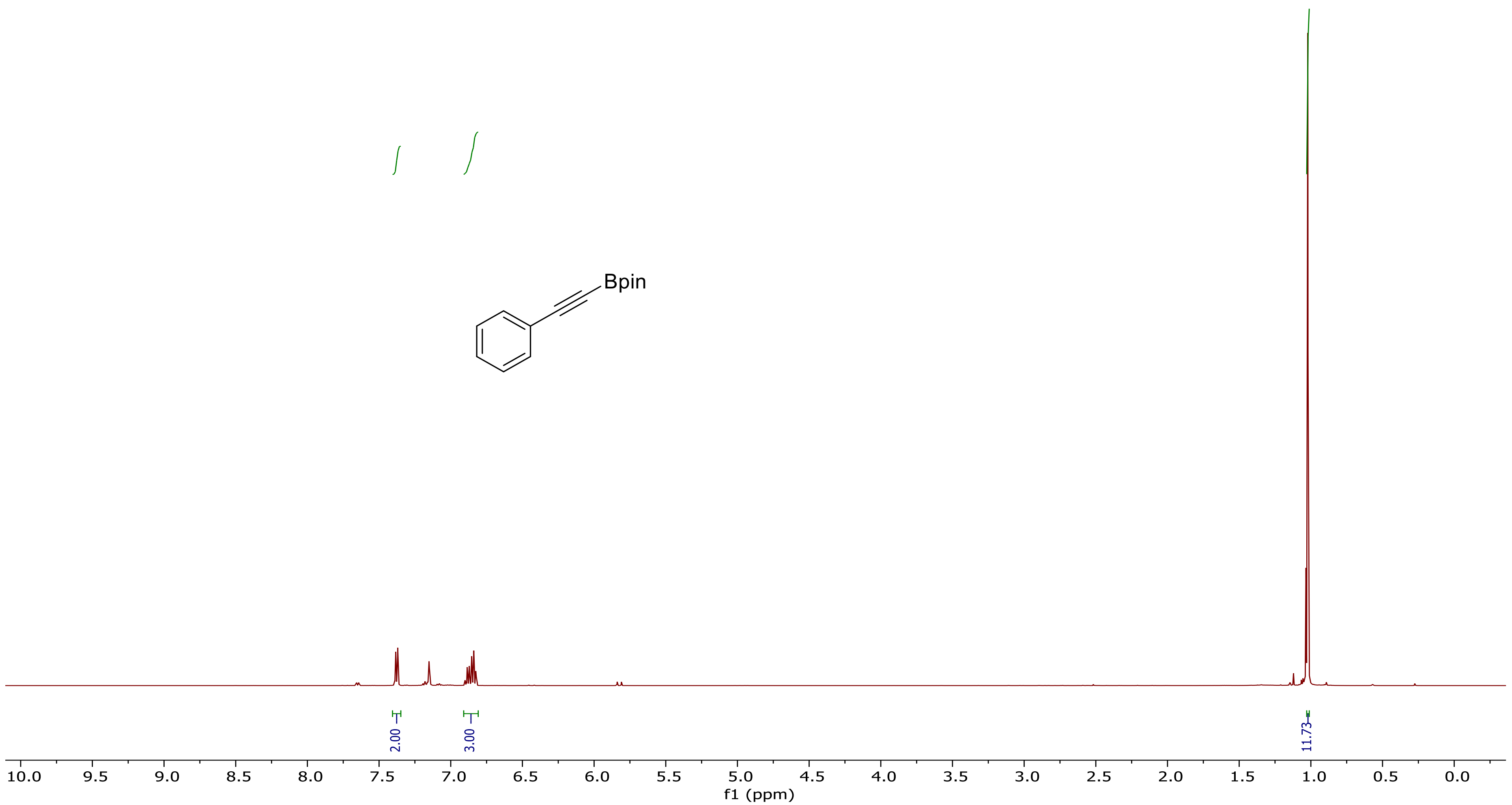

^{1}H NMR (C_6D_6, 500.12 MHz) of 4,4,5,5-Tetramethyl-2-(phenylethynyl)-1,3,2-dioxaborolane.

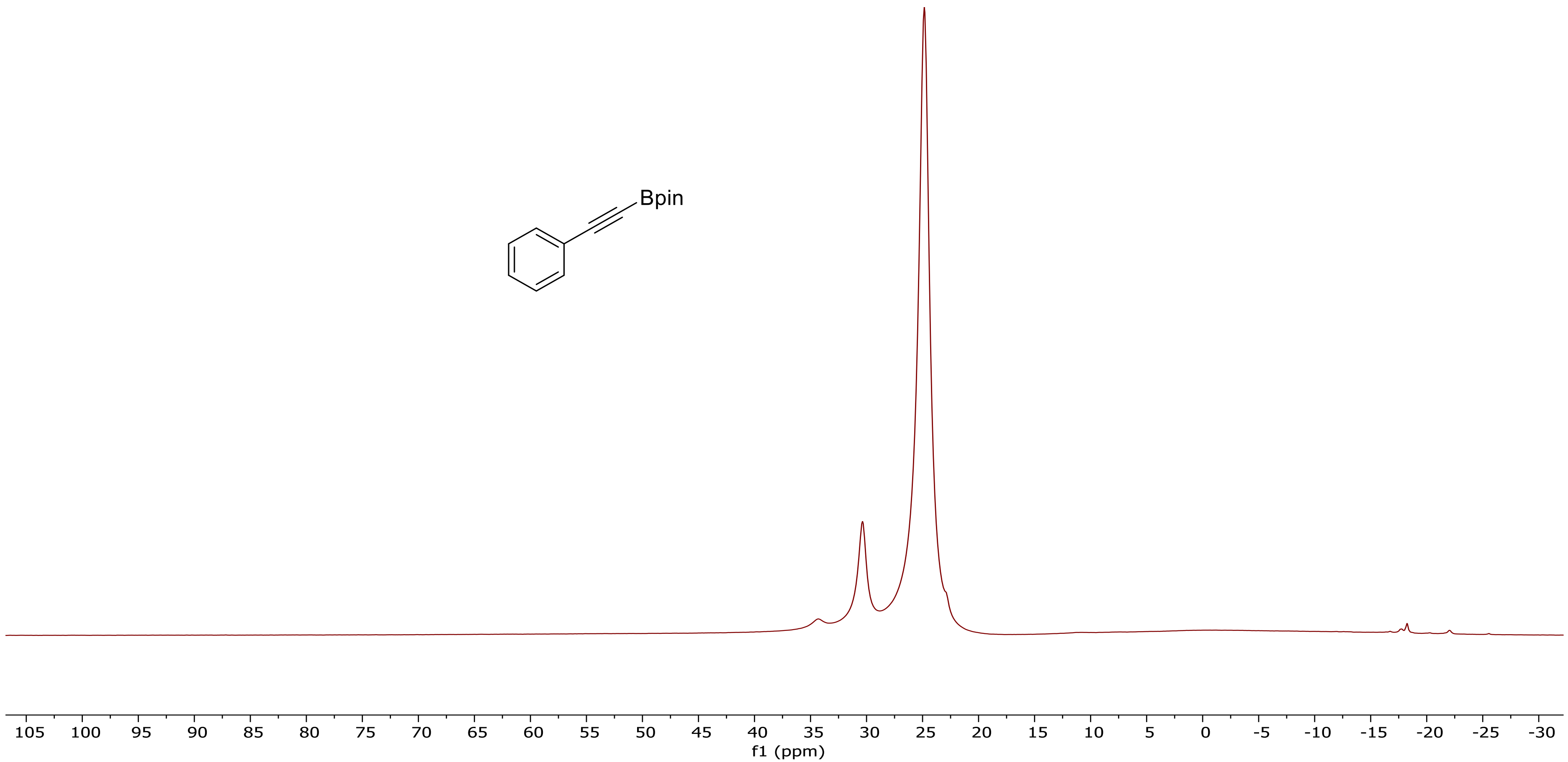

^{11}B NMR (C_6D_6, 128.34 MHz) of 4,4,5,5-Tetramethyl-2-(phenylethynyl)-1,3,2-dioxaborolane.

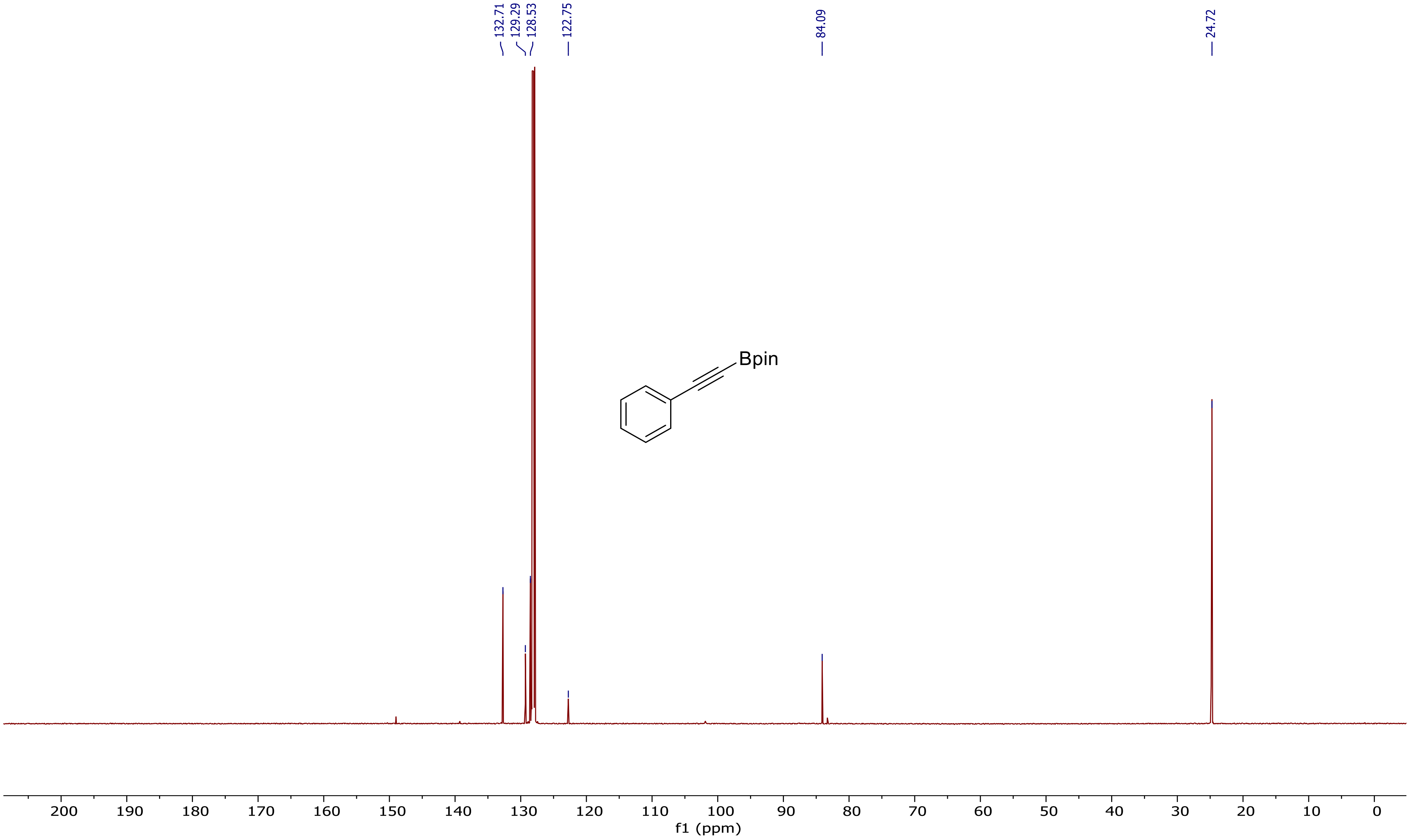

^{13}C NMR (C_6D_6, 125.77 MHz) of 4,4,5,5-Tetramethyl-2-(phenylethynyl)-1,3,2-dioxaborolane.

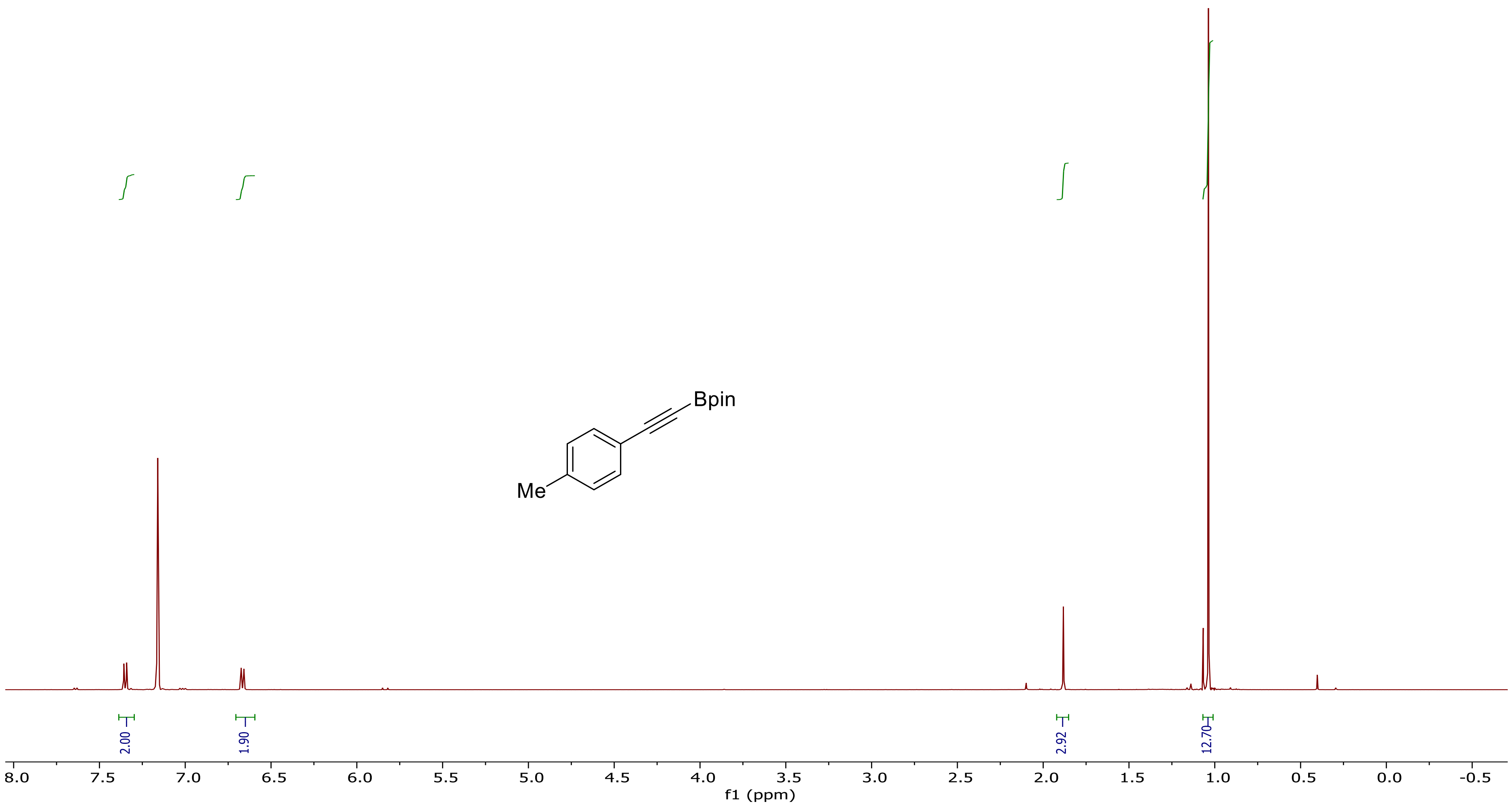

^{1}H NMR (C_6D_6, 500.12 MHz) of 4,4,5,5-Tetramethyl-2-(*p*-tolylethynyl)-1,3,2-dioxaborolane.

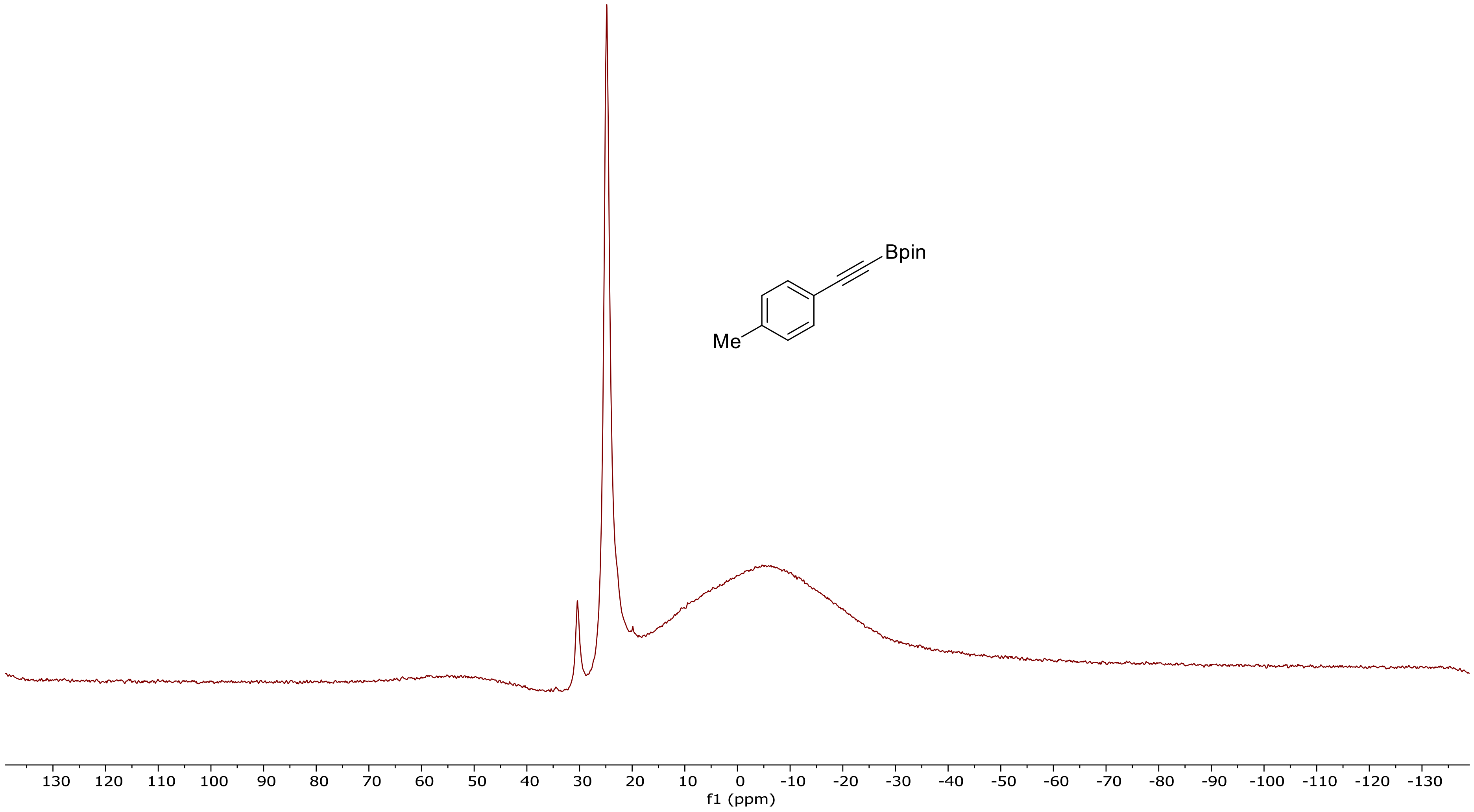

^{11}B NMR (C_6D_6, 128.34 MHz) of 4,4,5,5-Tetramethyl-2-(*p*-tolylethynyl)-1,3,2-dioxaborolane.

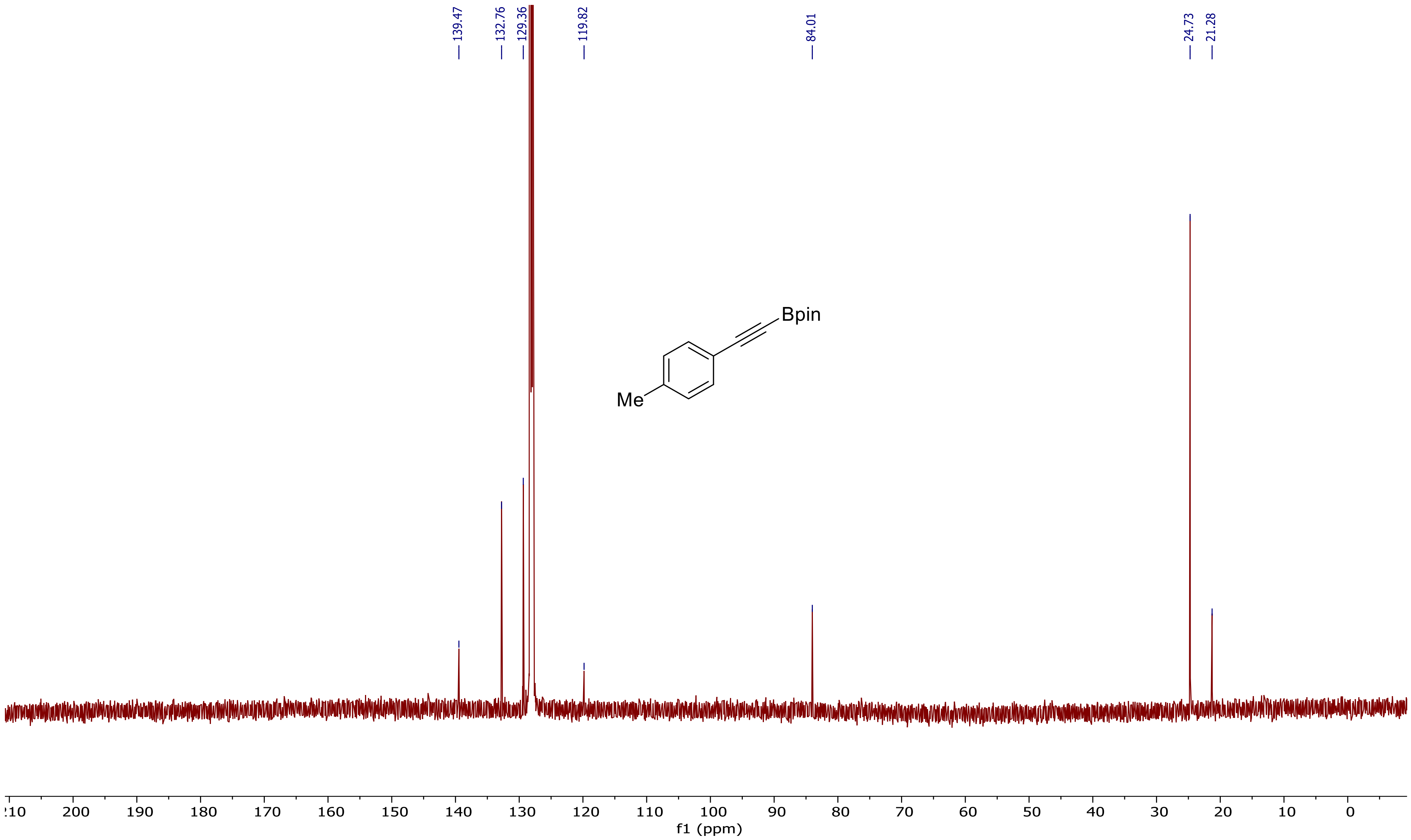

^{13}C NMR (C_6D_6, 125.77 MHz) of 4,4,5,5-Tetramethyl-2-(*p*-tolylethynyl)-1,3,2-dioxaborolane.

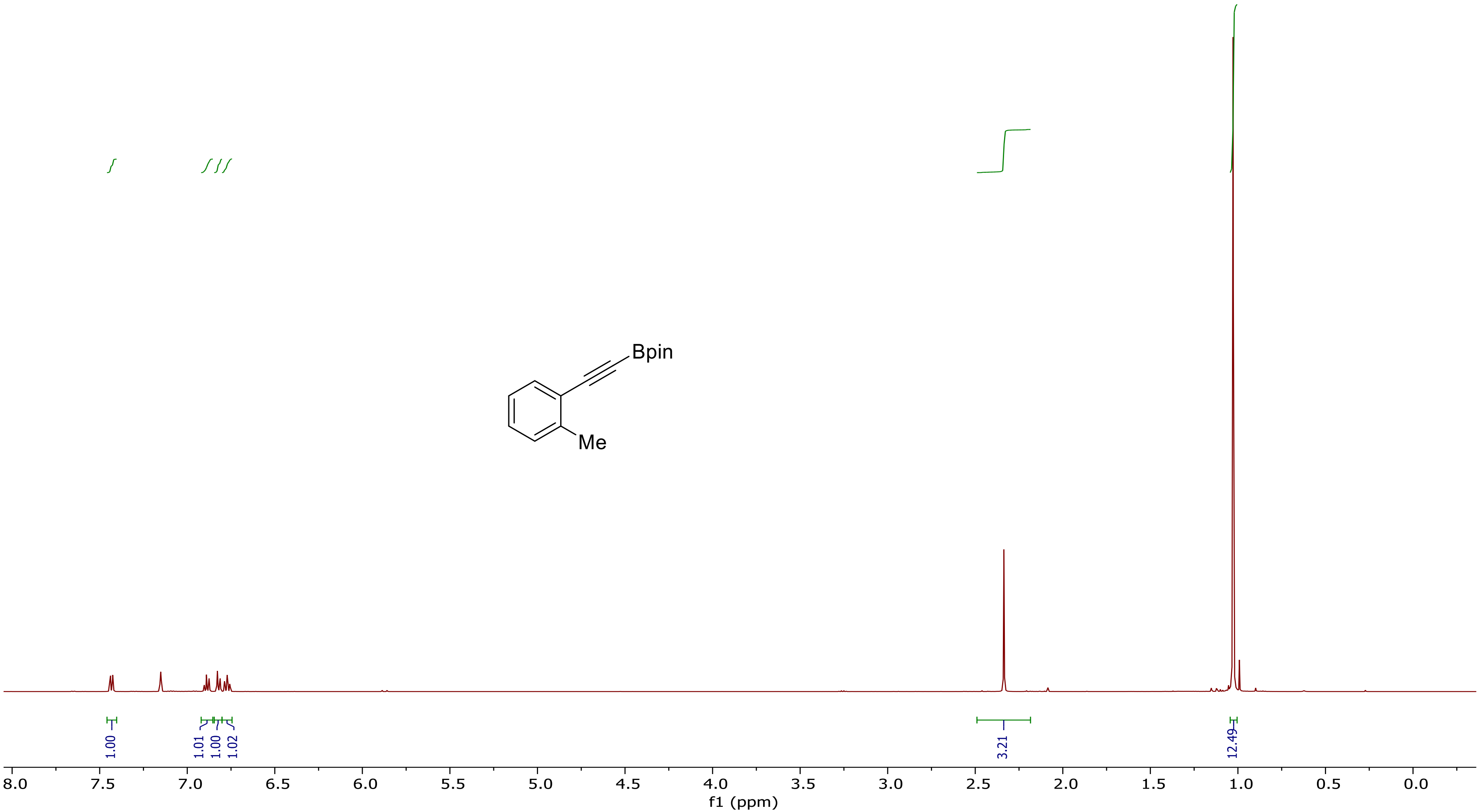

^{1}H NMR (C_6D_6, 500.12 MHz) of 4,4,5,5-Tetramethyl-2-(*o*-tolylethynyl)-1,3,2-dioxaborolane.

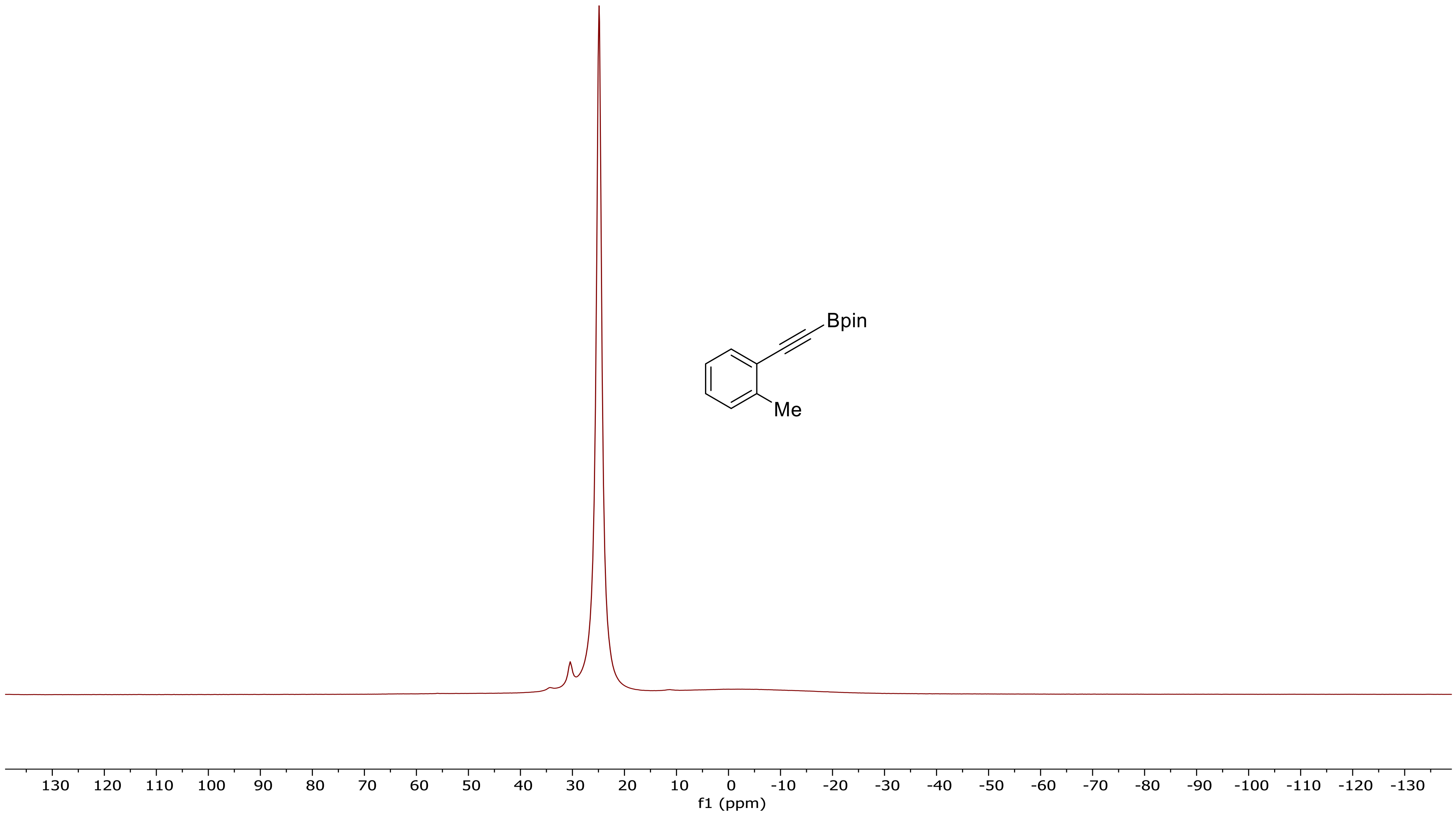

^{11}B NMR (C_6D_6, 128.34 MHz) of 4,4,5,5-Tetramethyl-2-(*o*-tolylethynyl)-1,3,2-dioxaborolane.

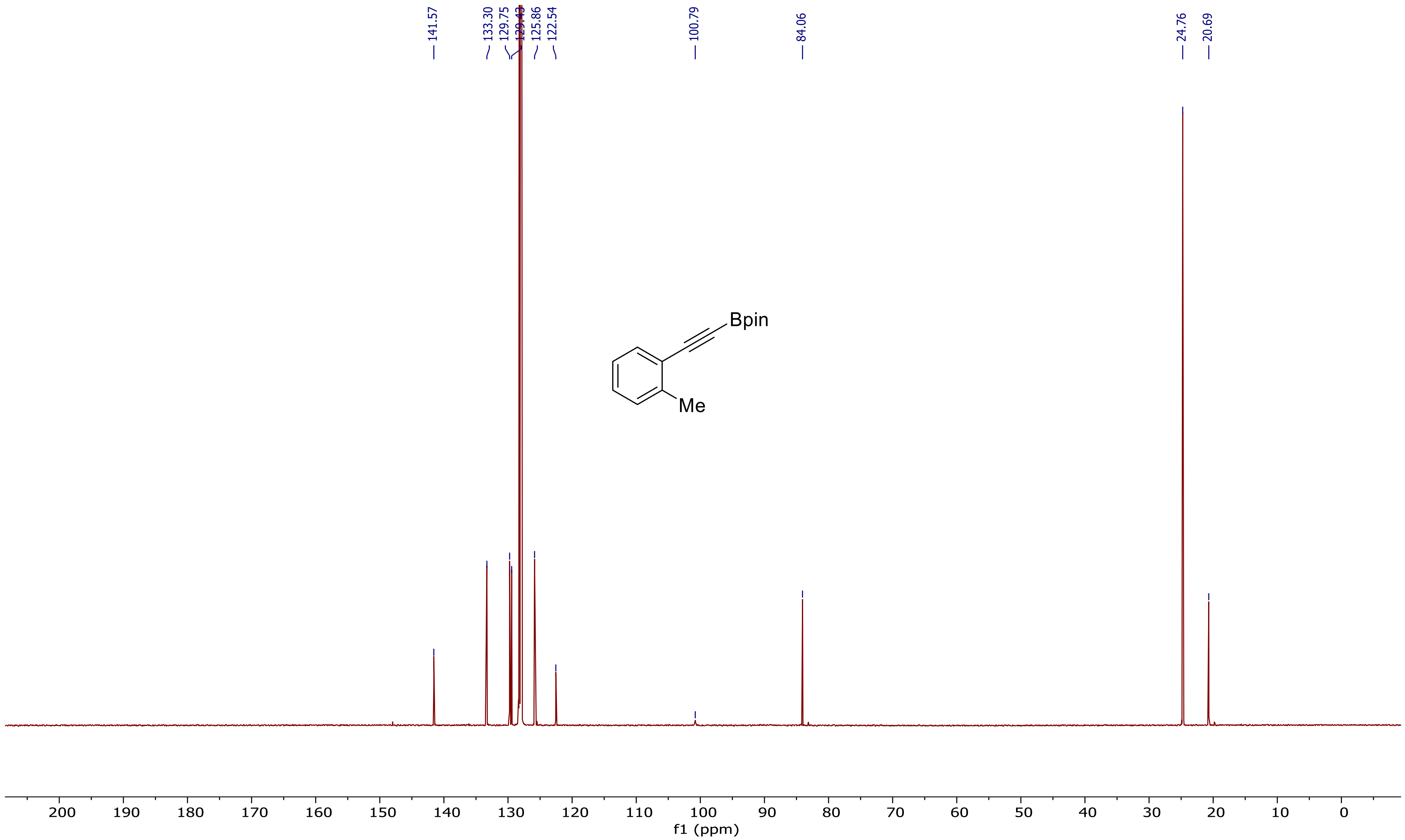

^{13}C NMR (C_6D_6, 125.77 MHz) of 4,4,5,5-Tetramethyl-2-(*o*-tolylethynyl)-1,3,2-dioxaborolane.

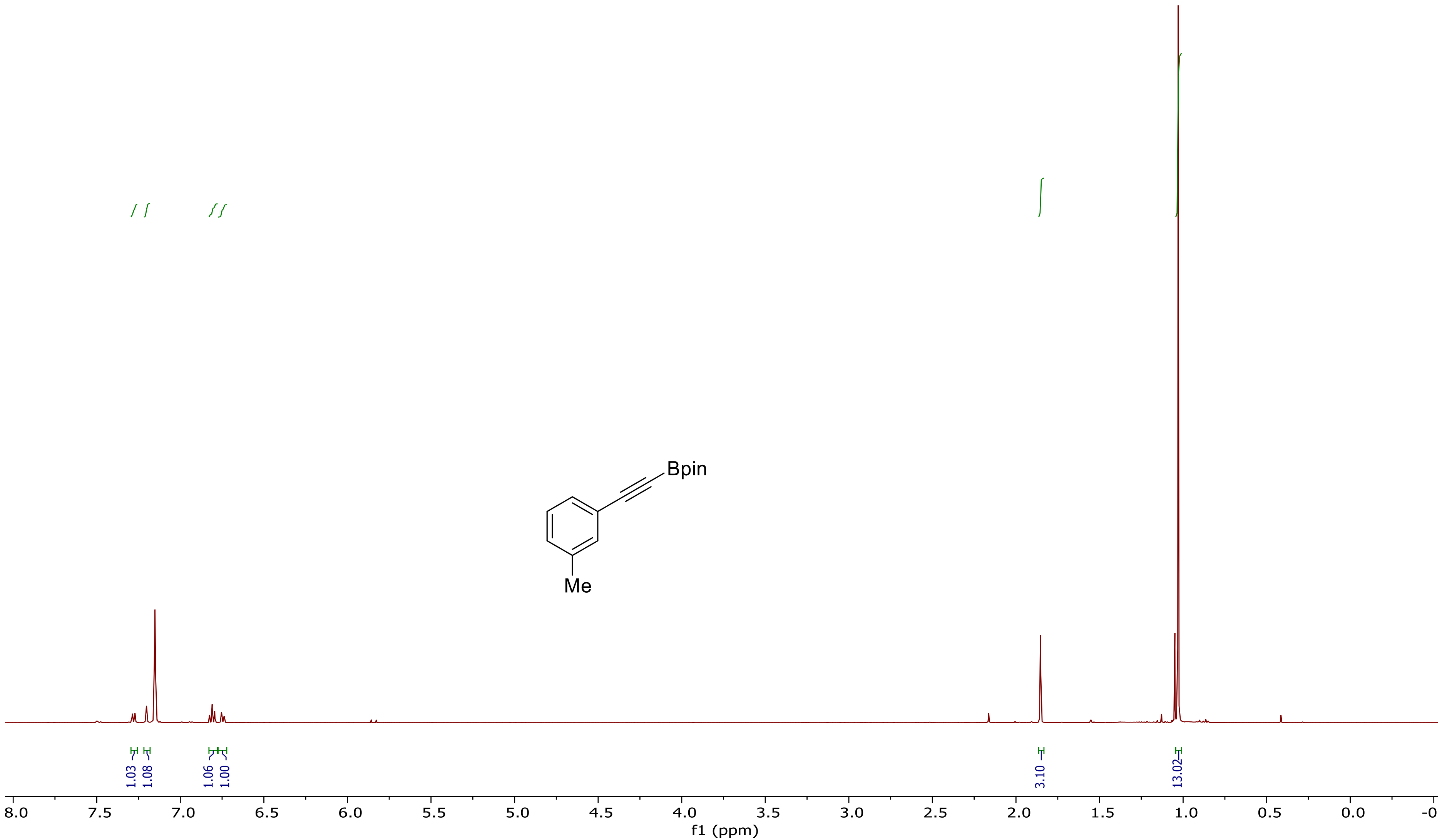

^{1}H NMR (C_6D_6, 500.12 MHz) of 4,4,5,5-Tetramethyl-2-(*m*-tolylethynyl)-1,3,2-dioxaborolane.

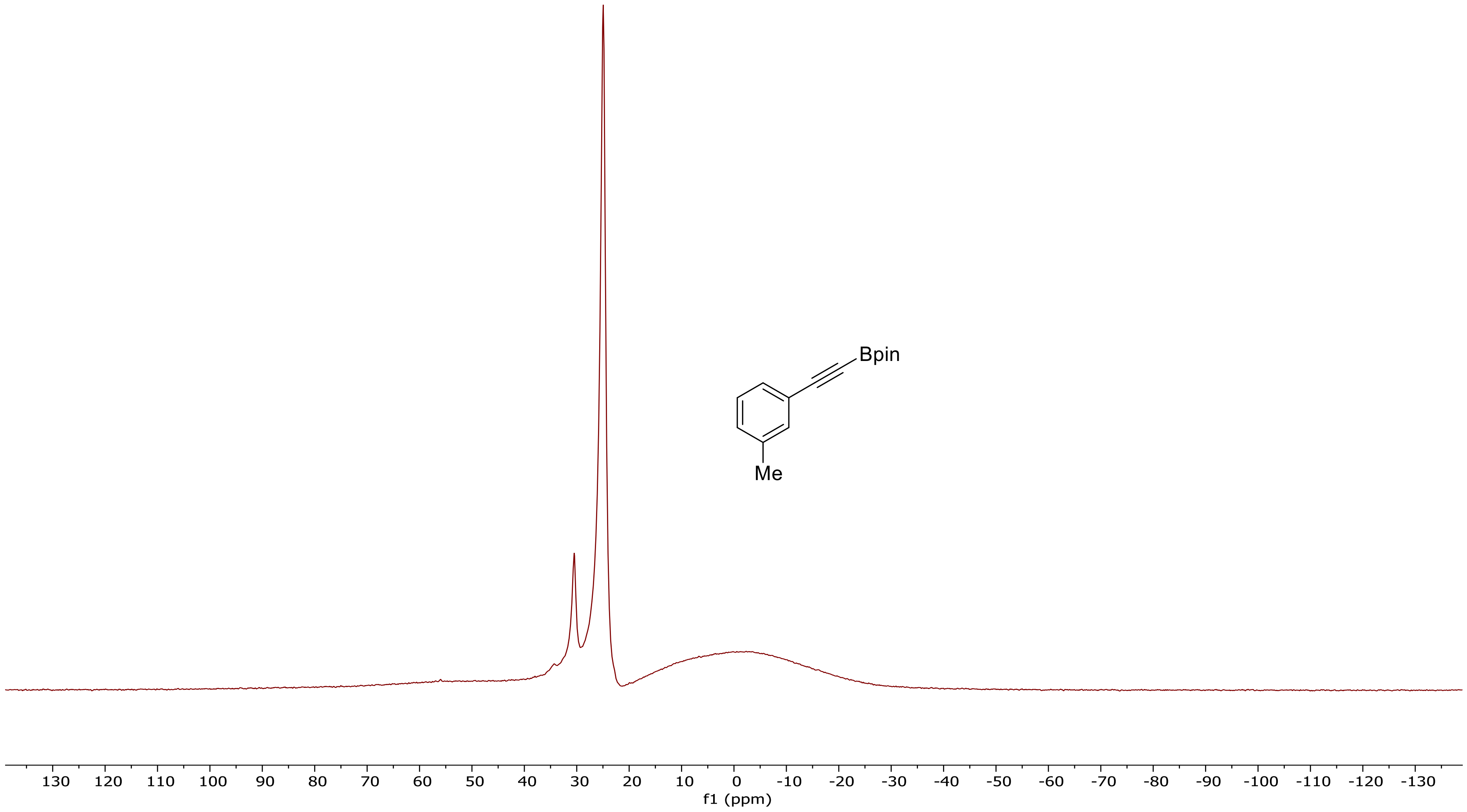

^{11}B NMR (C_6D_6, 128.34 MHz) of 4,4,5,5-Tetramethyl-2-(*m*-tolylethynyl)-1,3,2-dioxaborolane.

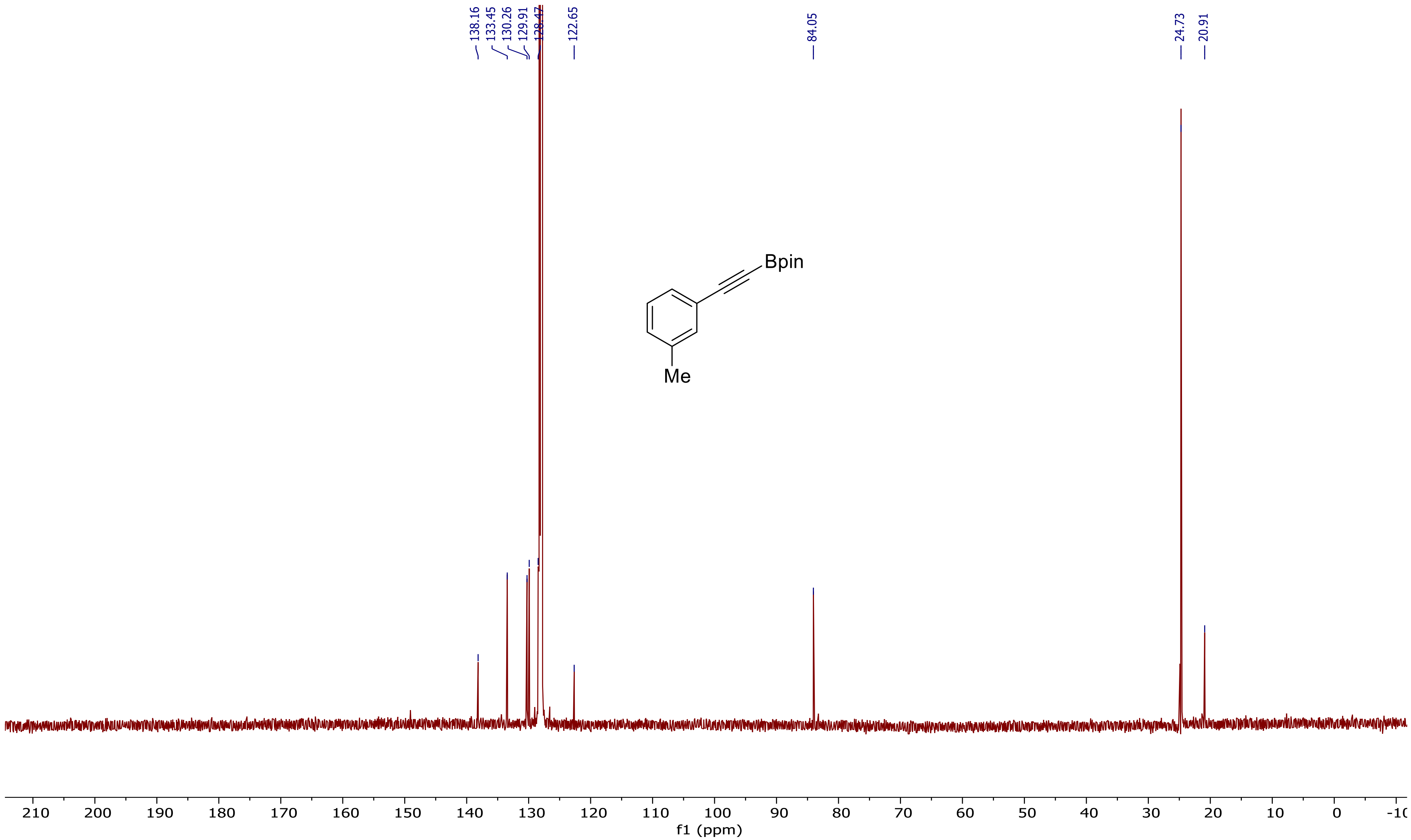

^{13}C NMR (C_6D_6, 125.77 MHz) of 4,4,5,5-Tetramethyl-2-(*m*-tolylethynyl)-1,3,2-dioxaborolane.

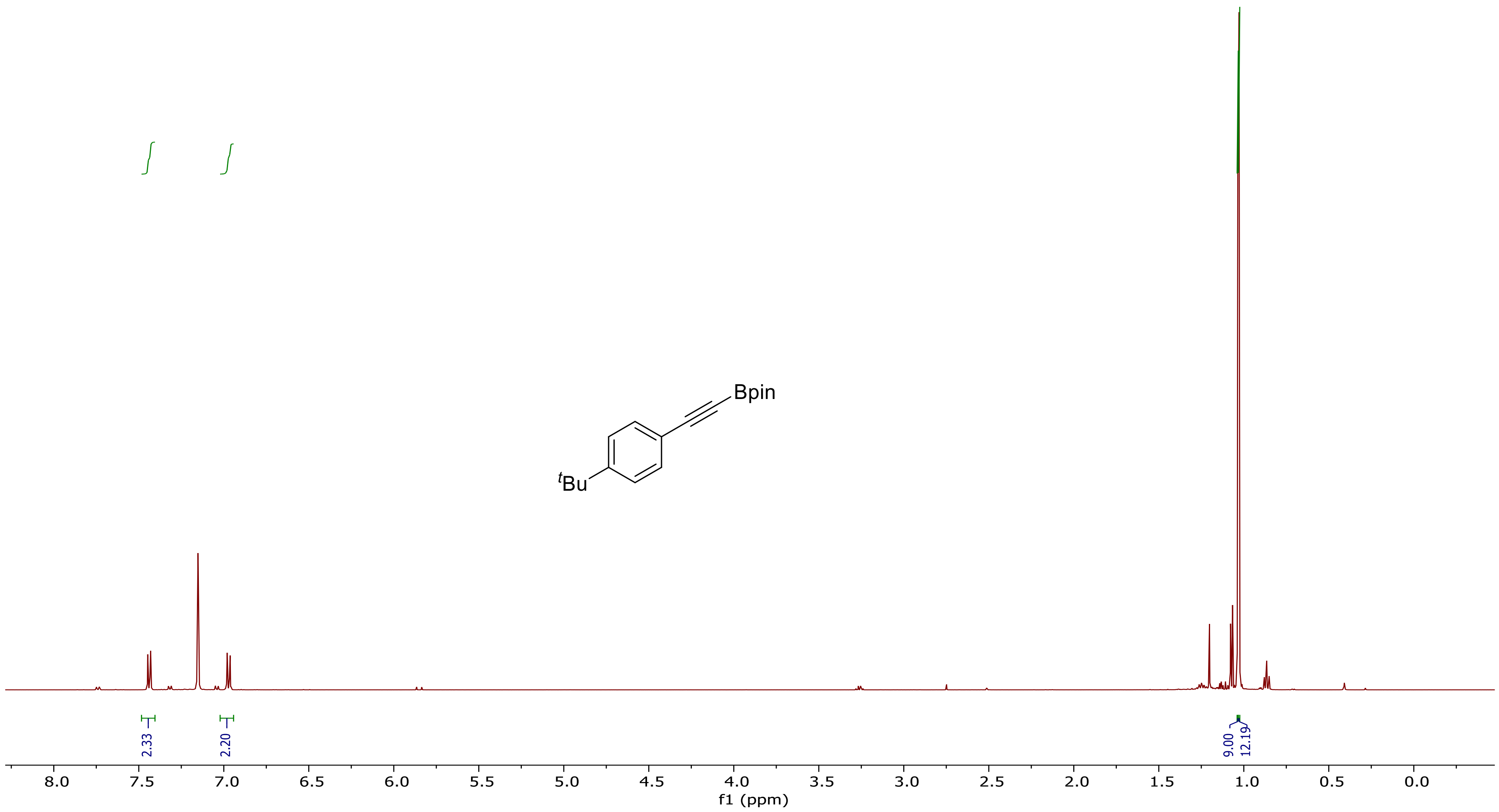

^{1}H NMR (C_6D_6, 500.12 MHz) of 2-((4-(*t*-Butyl)phenyl)ethynyl)-4,4,5,5-tetramethyl-1,3,2-dioxaborolane.

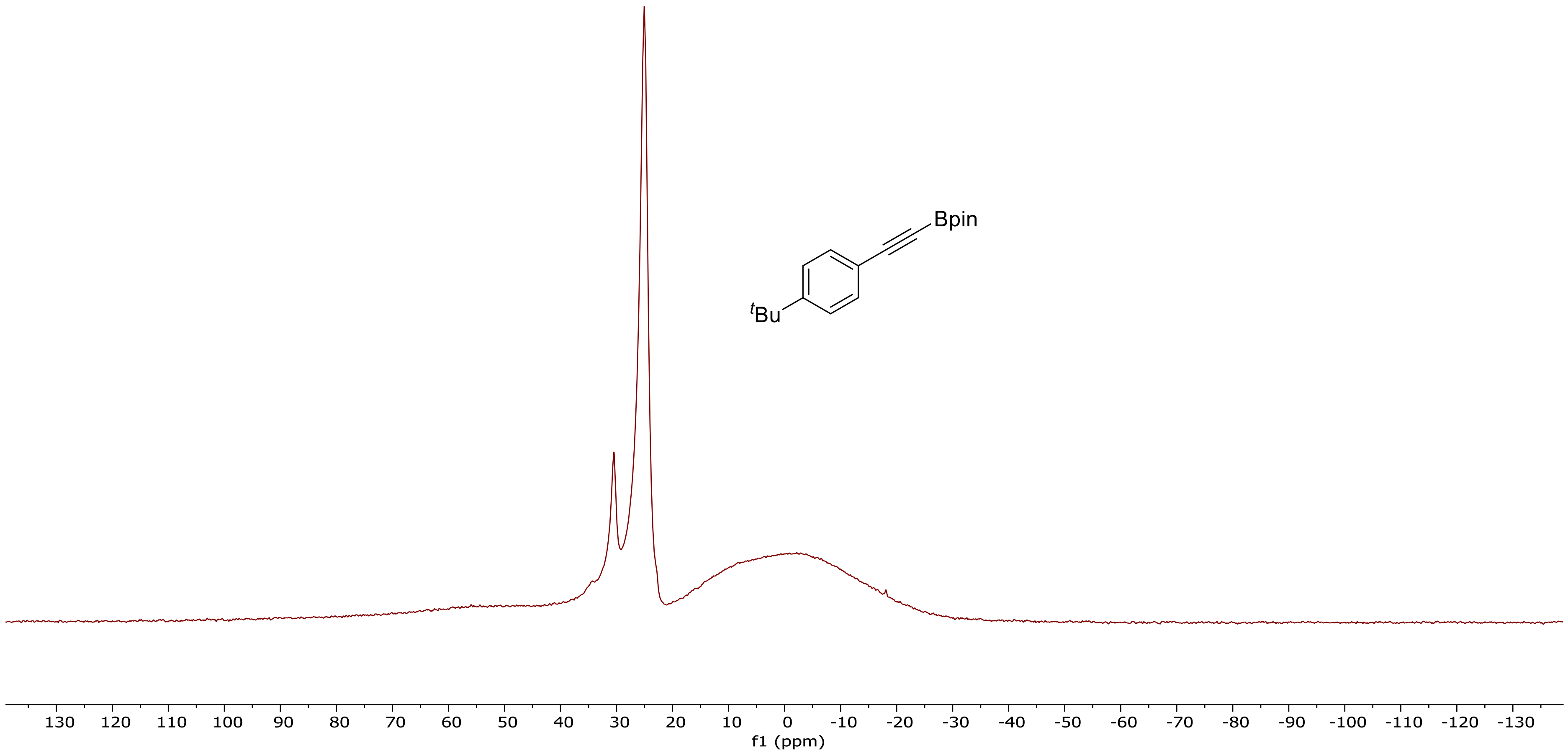

^{11}B NMR (C_6D_6, 128.34 MHz) of 2-((4-(*t*-Butyl)phenyl)ethynyl)-4,4,5,5-tetramethyl-1,3,2-dioxaborolane.

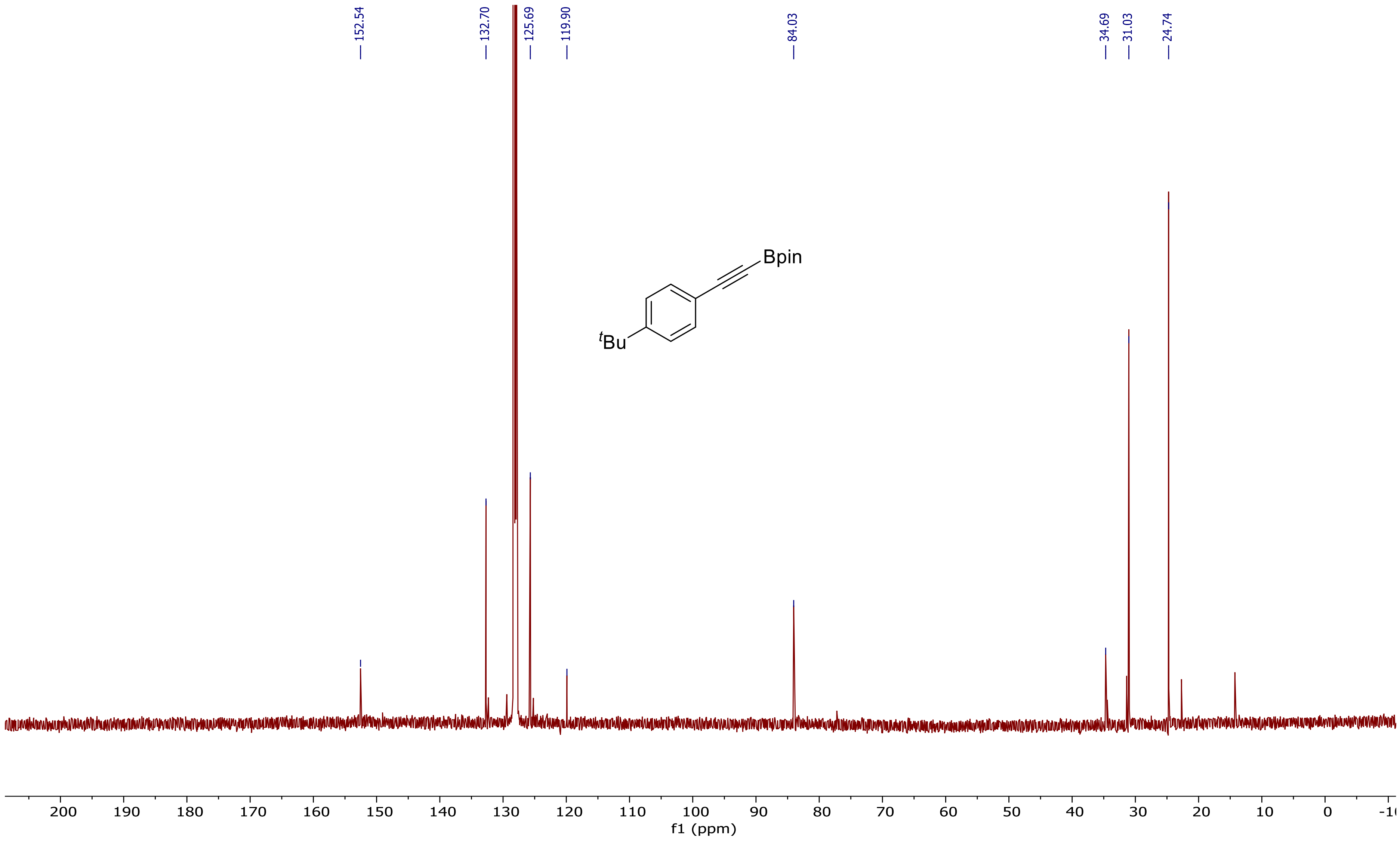

^{13}C NMR (C_6D_6, 125.77 MHz) of 2-((4-(*t*-Butyl)phenyl)ethynyl)-4,4,5,5-tetramethyl-1,3,2-dioxaborolane.

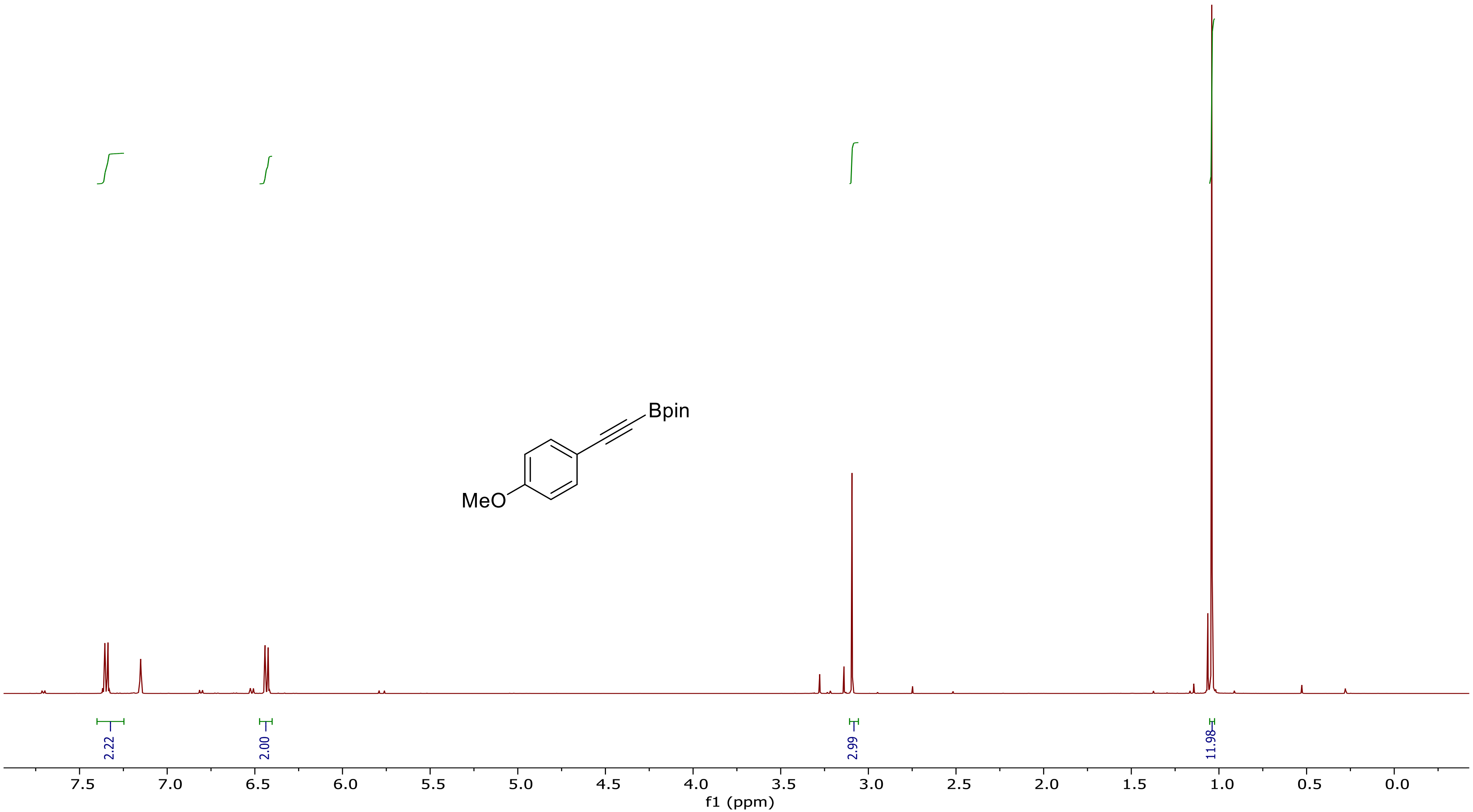

^{1}H NMR (C_6D_6, 500.12 MHz) of 2-((4-Methoxyphenyl)ethynyl)-4,4,5,5-tetramethyl-1,3,2-dioxaborolane.

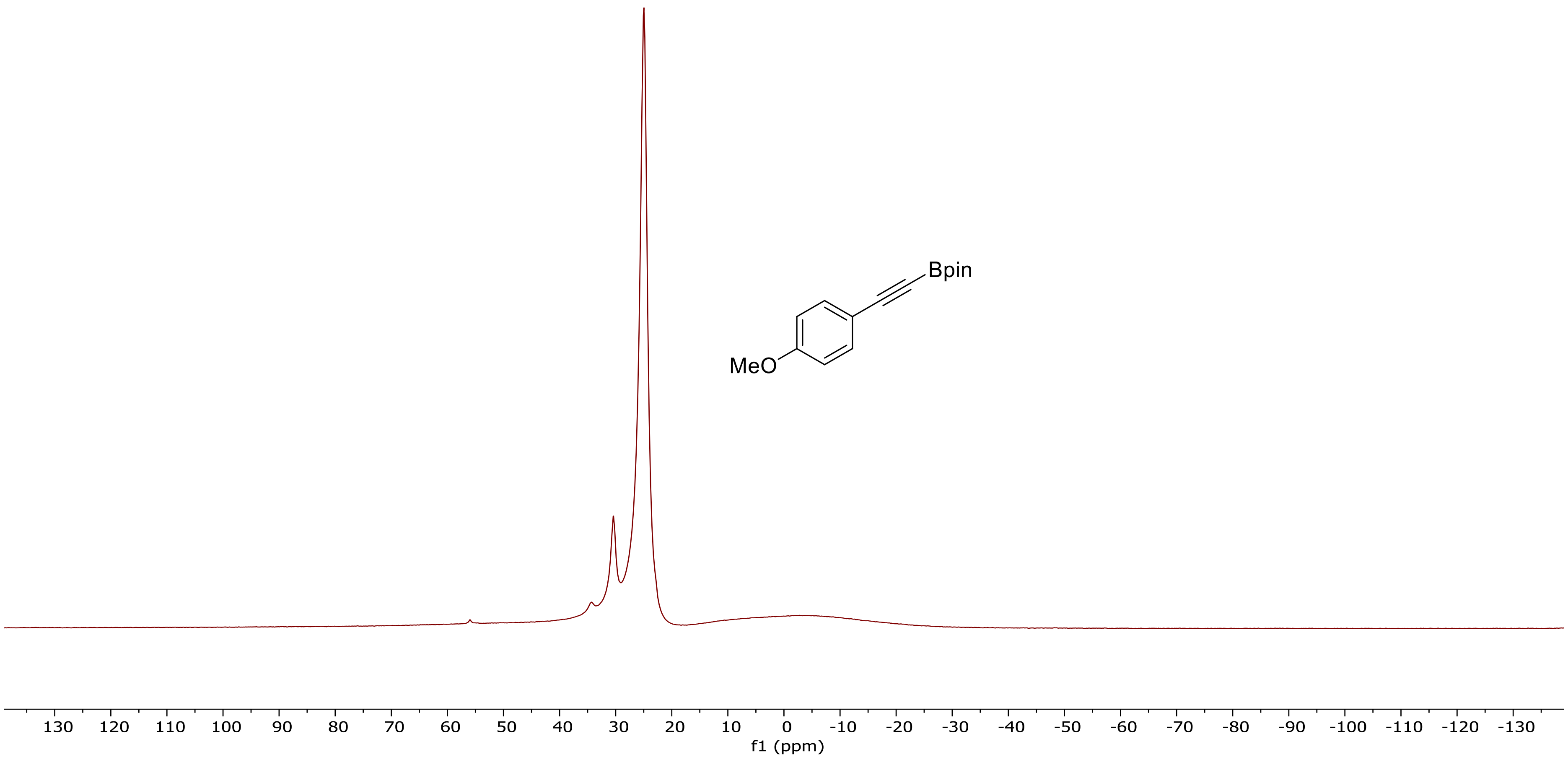

^{11}B NMR (C_6D_6, 128.34 MHz) of 2-((4-Methoxyphenyl)ethynyl)-4,4,5,5-tetramethyl-1,3,2-dioxaborolane.

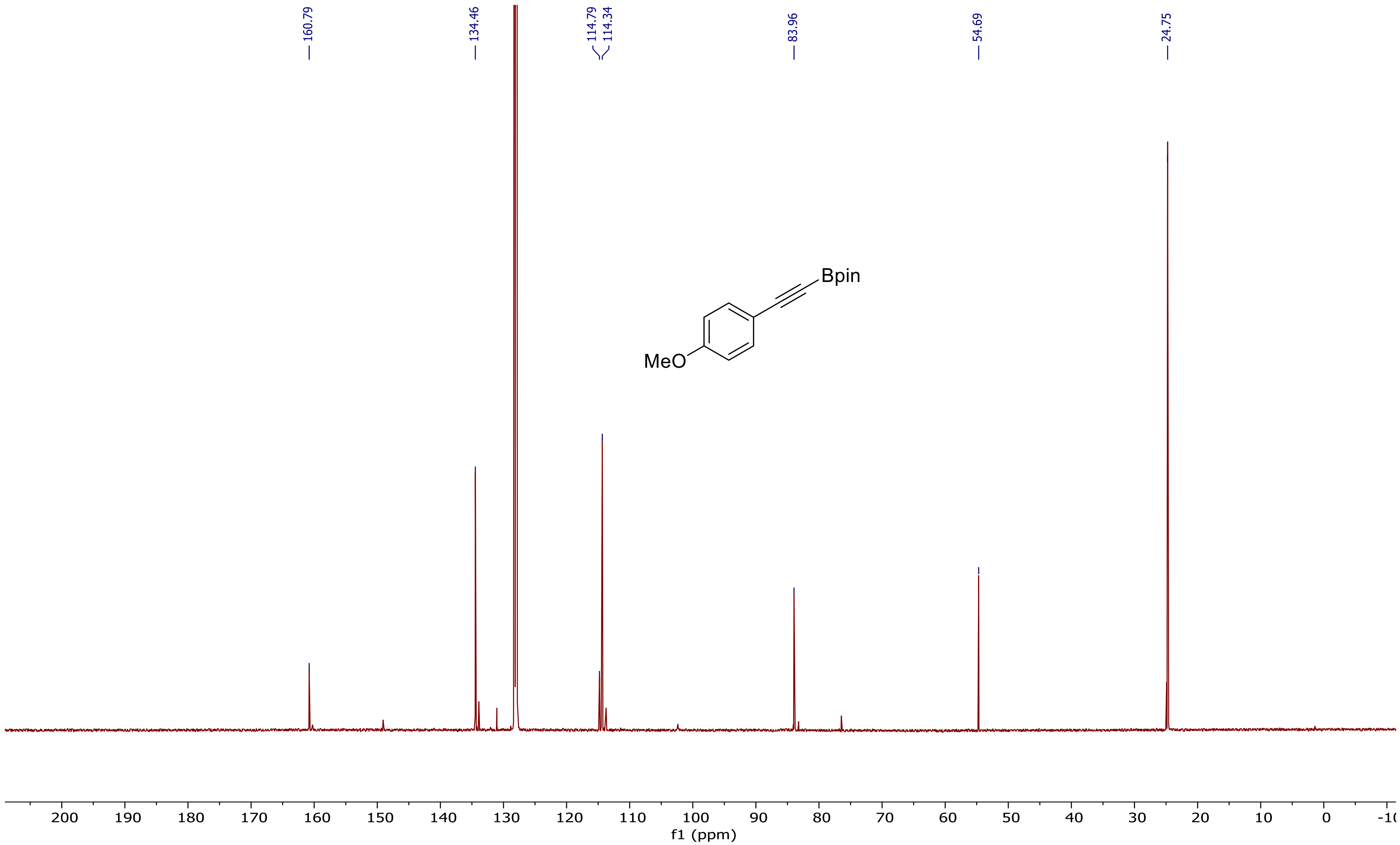

^{13}C NMR (C_6D_6, 125.77 MHz) of 2-((4-Methoxyphenyl)ethynyl)-4,4,5,5-tetramethyl-1,3,2-dioxaborolane.

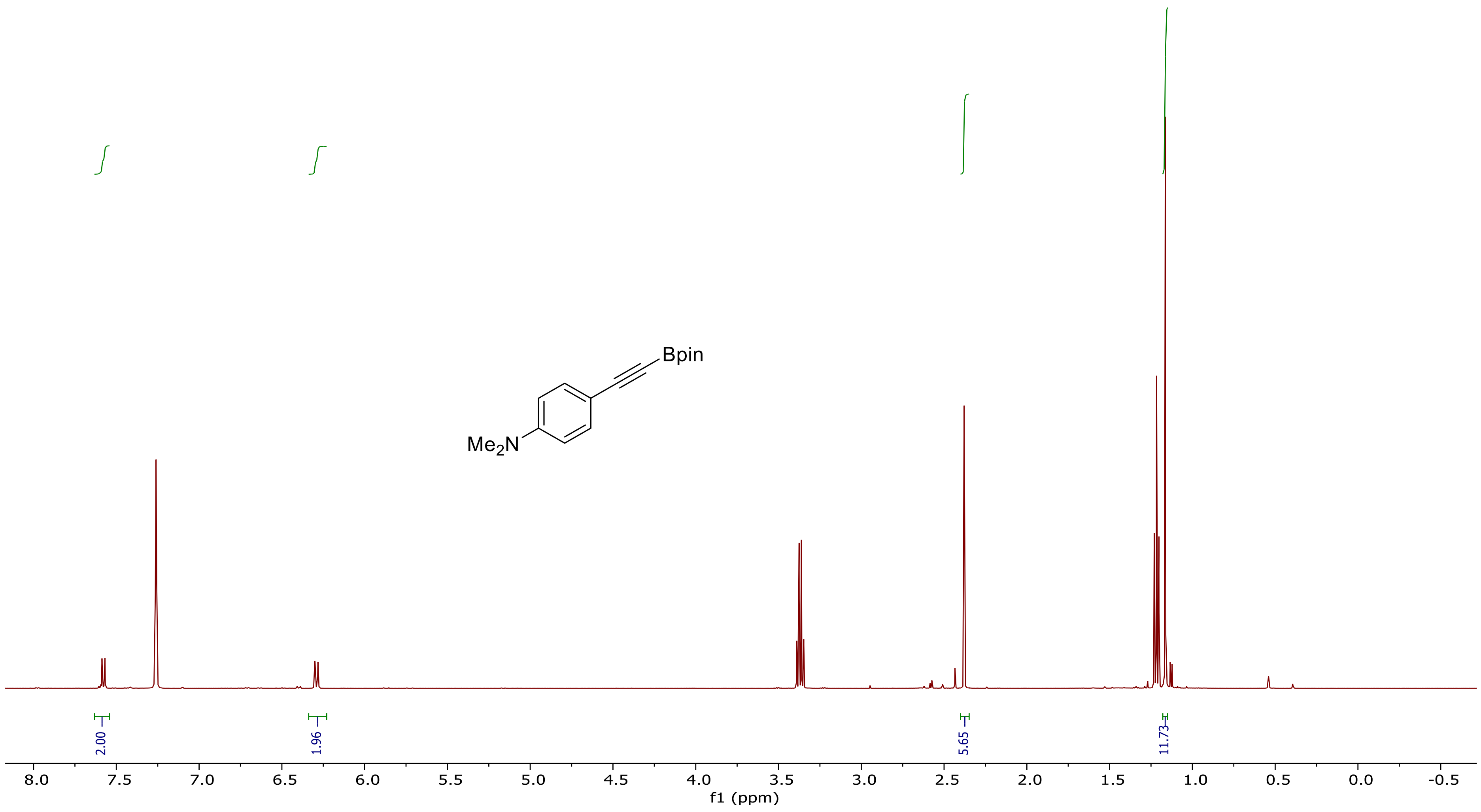

^{1}H NMR (C_6D_6, 500.12 MHz) of 4,4,5,5-Tetramethyl-2-(*p*-N,N-dimethylaniline)-1,3,2-dioxaborolane.

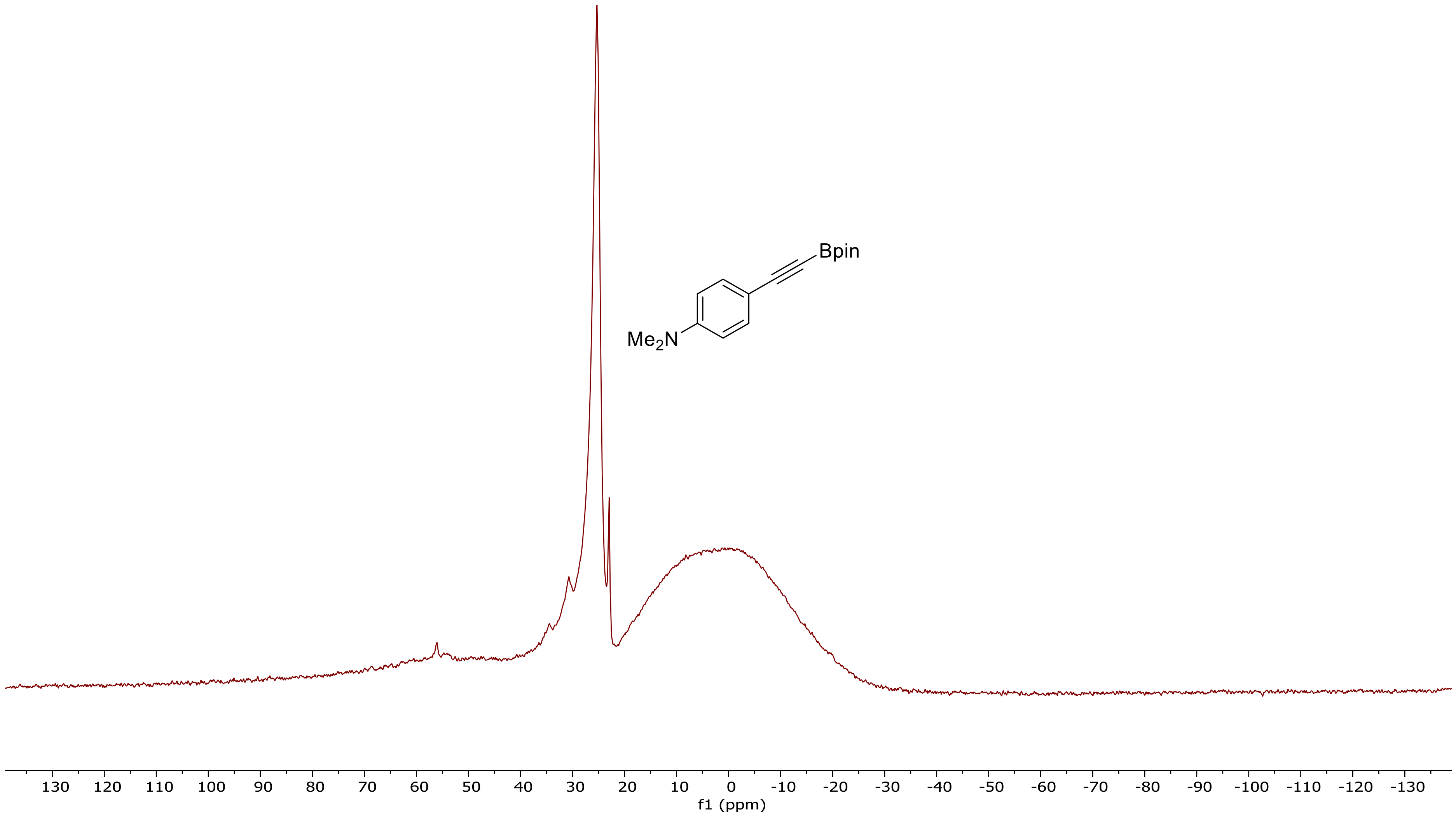

^{11}B NMR (C_6D_6, 128.34 MHz) of 4,4,5,5-Tetramethyl-2-(*p*-N,N-dimethylaniline)-1,3,2-dioxaborolane.

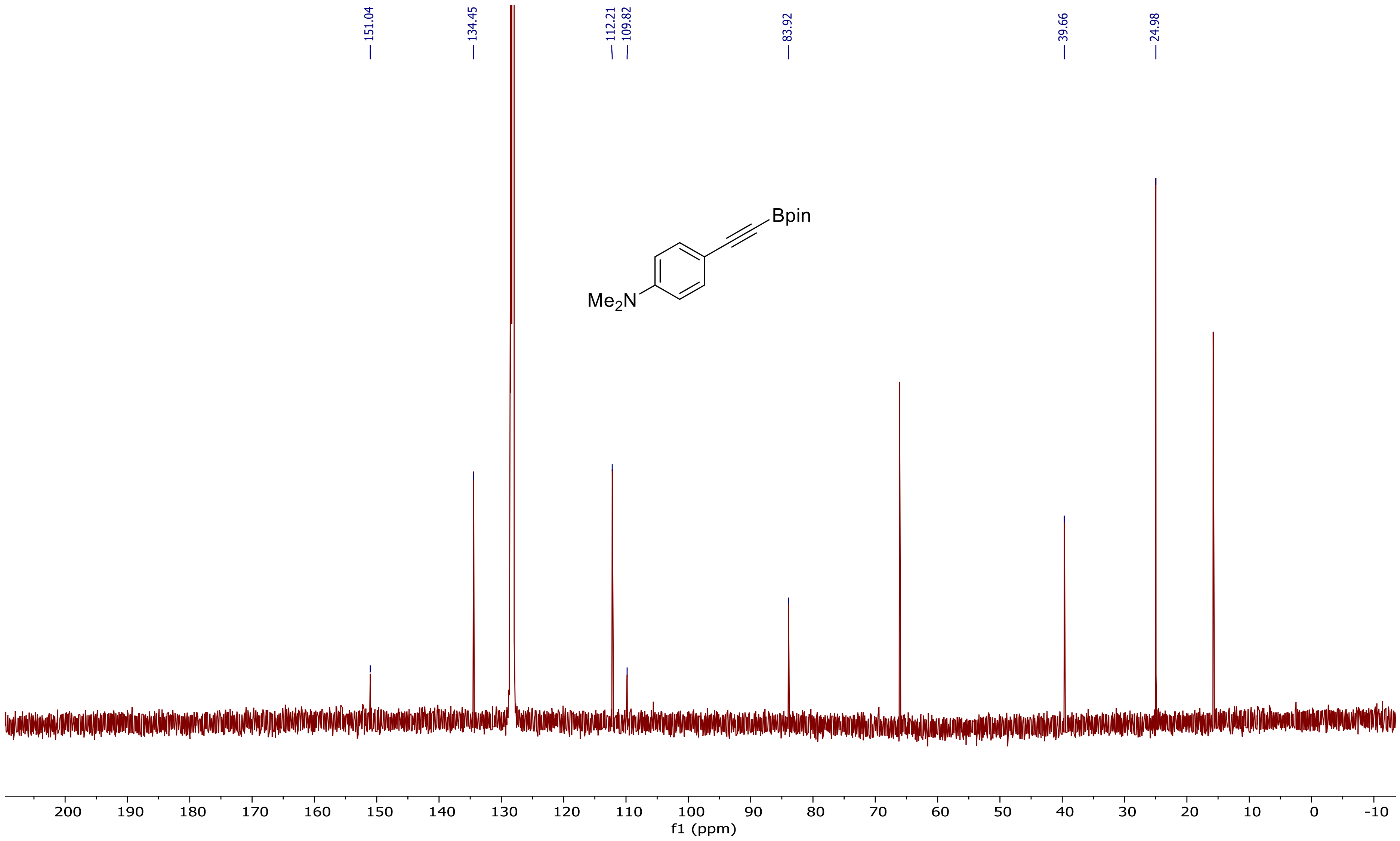

^{13}C NMR (C_6D_6, 125.77 MHz) of 4,4,5,5-Tetramethyl-2-(*p*-N,N-dimethylaniline)-1,3,2-dioxaborolane.

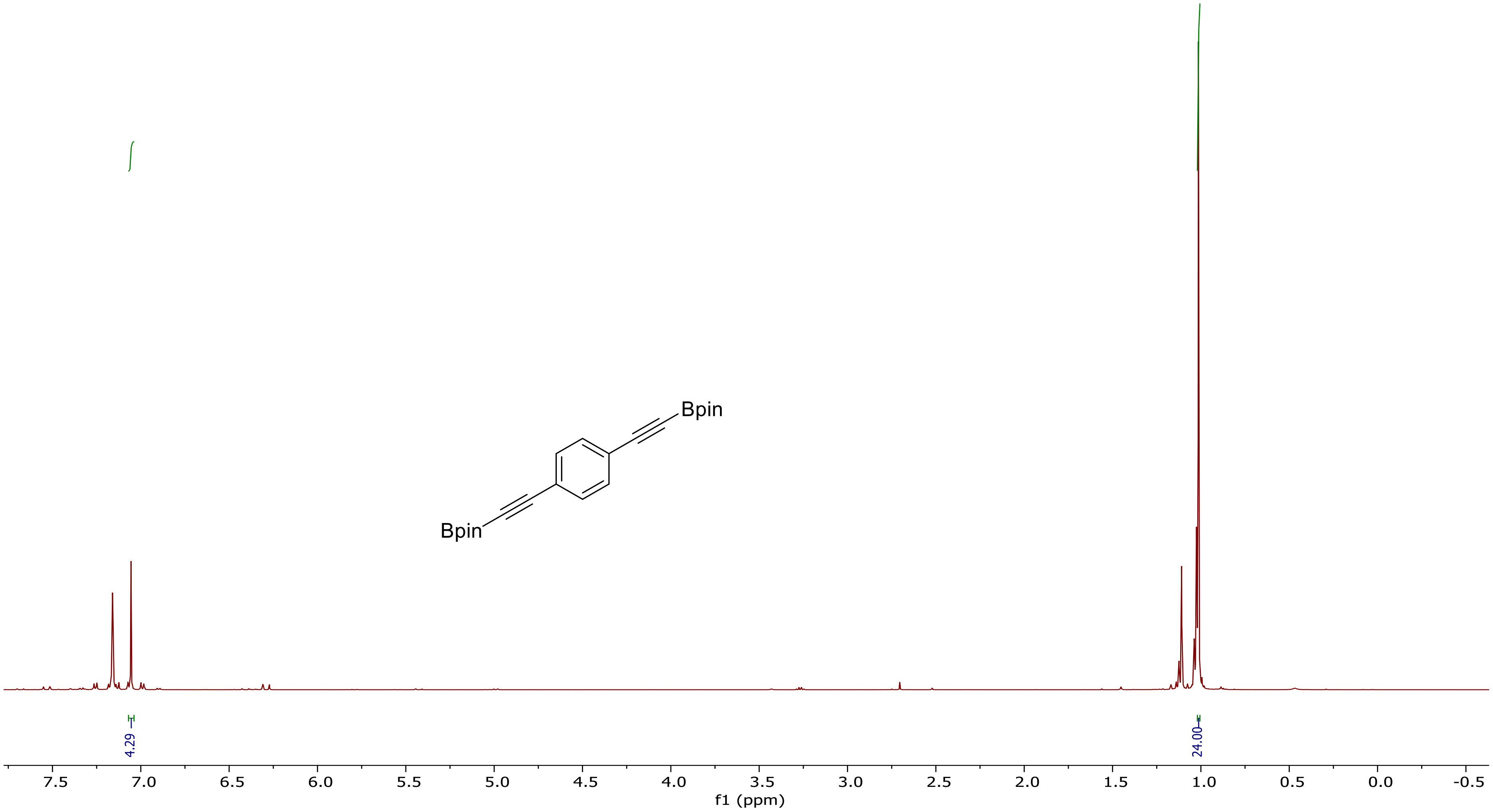

^{1}H NMR (C_6D_6, 500.12 MHz) of 1,4-Bis((4,4,5,5-tetramethyl-1,3,2-dioxaborolan-2-yl)ethynyl)benzene.

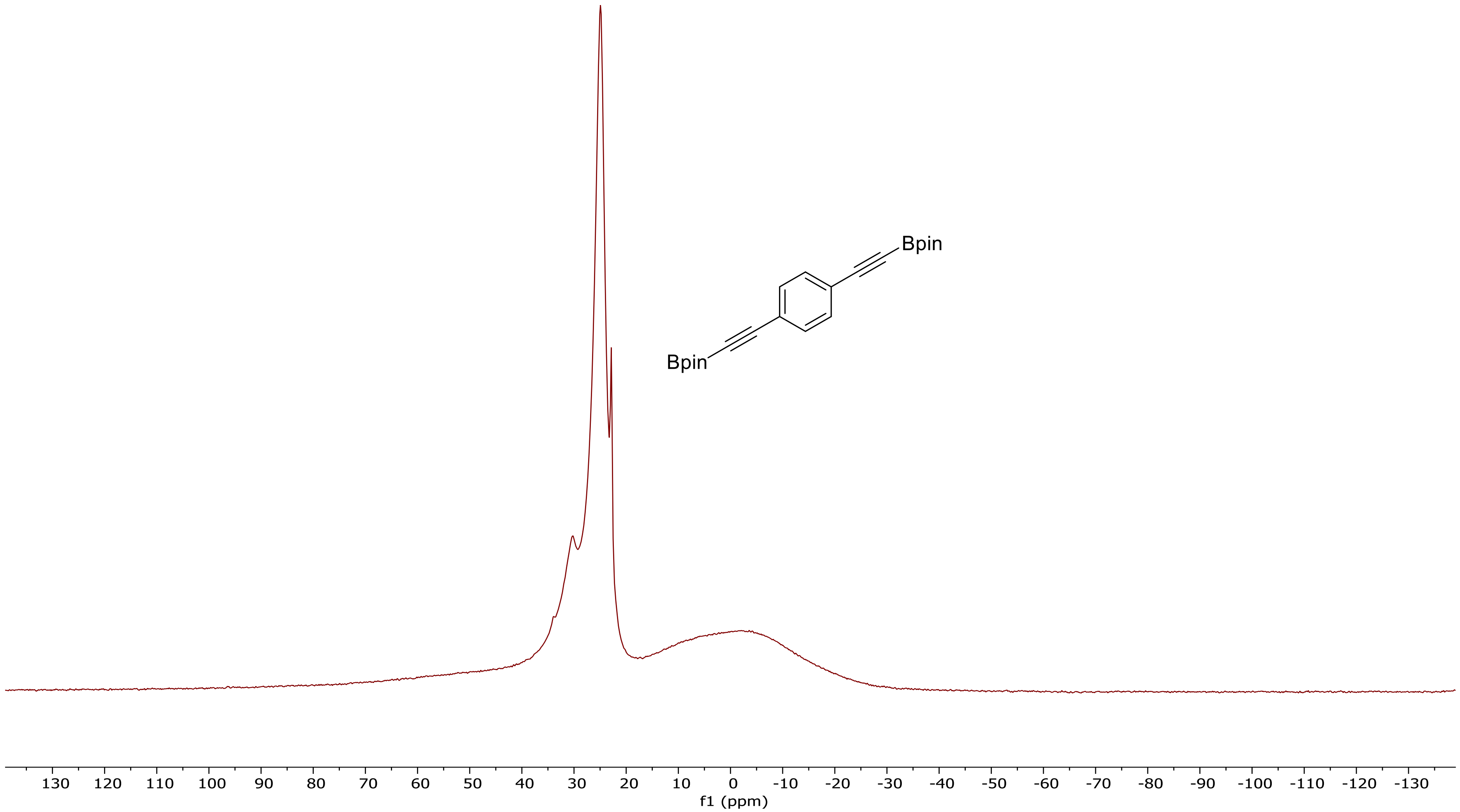

^{11}B NMR (C_6D_6, 128.34 MHz) of 1,4-Bis((4,4,5,5-tetramethyl-1,3,2-dioxaborolan-2-yl)ethynyl)benzene.

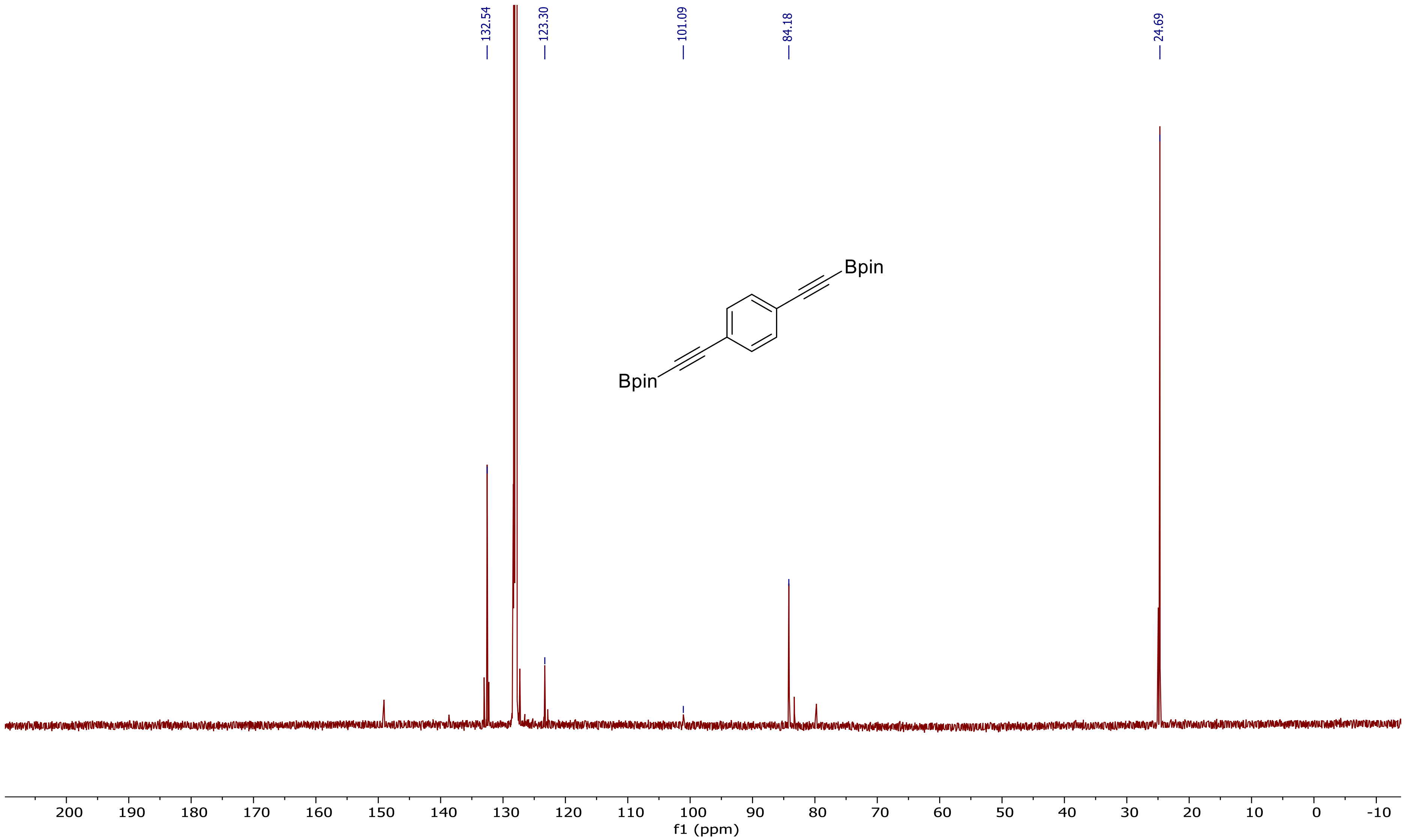

^{13}C NMR (C_6D_6, 125.77 MHz) of 1,4-Bis((4,4,5,5-tetramethyl-1,3,2-dioxaborolan-2-yl)ethynyl)benzene.

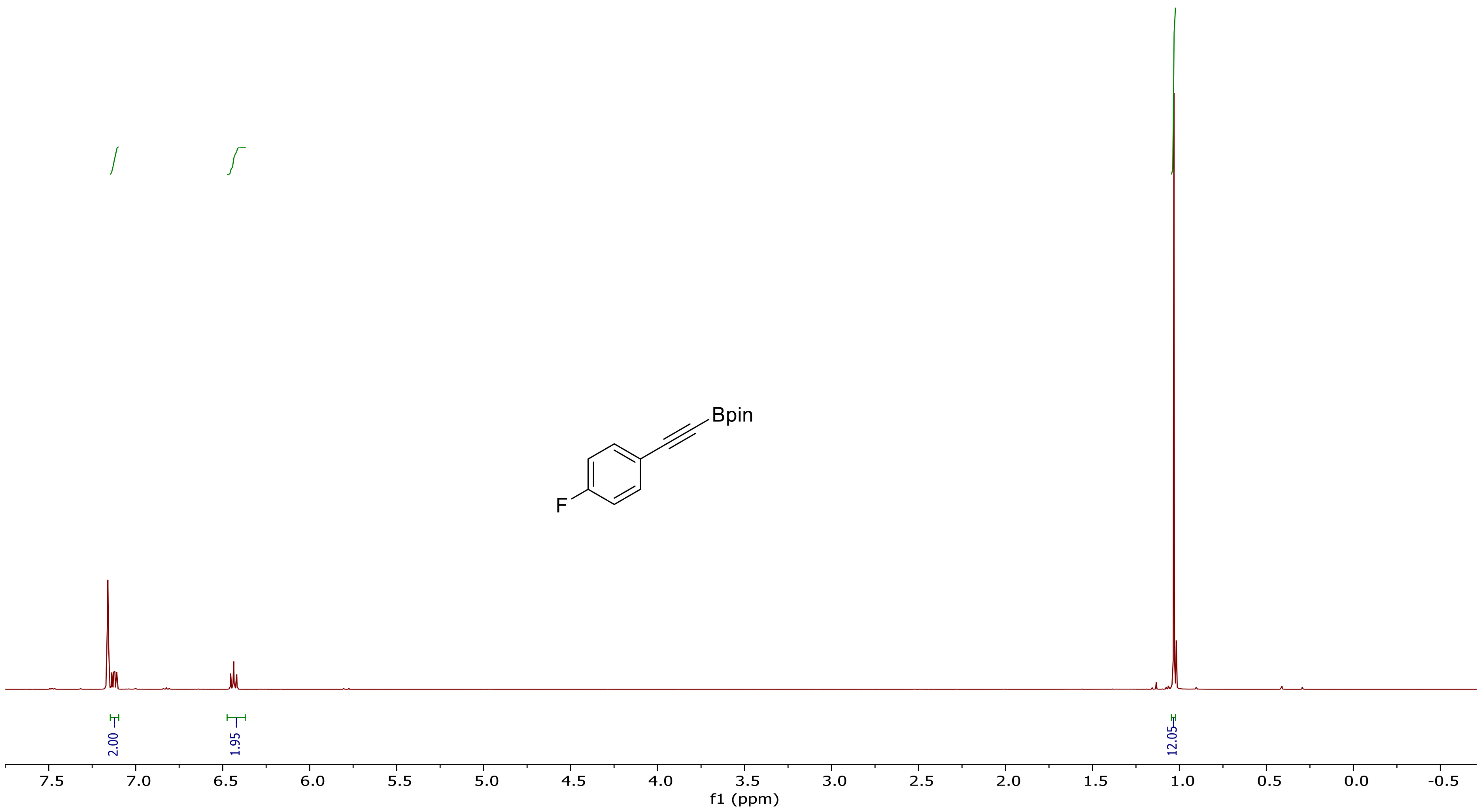

^{1}H NMR (C_6D_6, 500.12 MHz) of 2-((4-fluorophenyl)ethynyl)-4,4,5,5-tetramethyl-1,3,2-dioxaborolane.

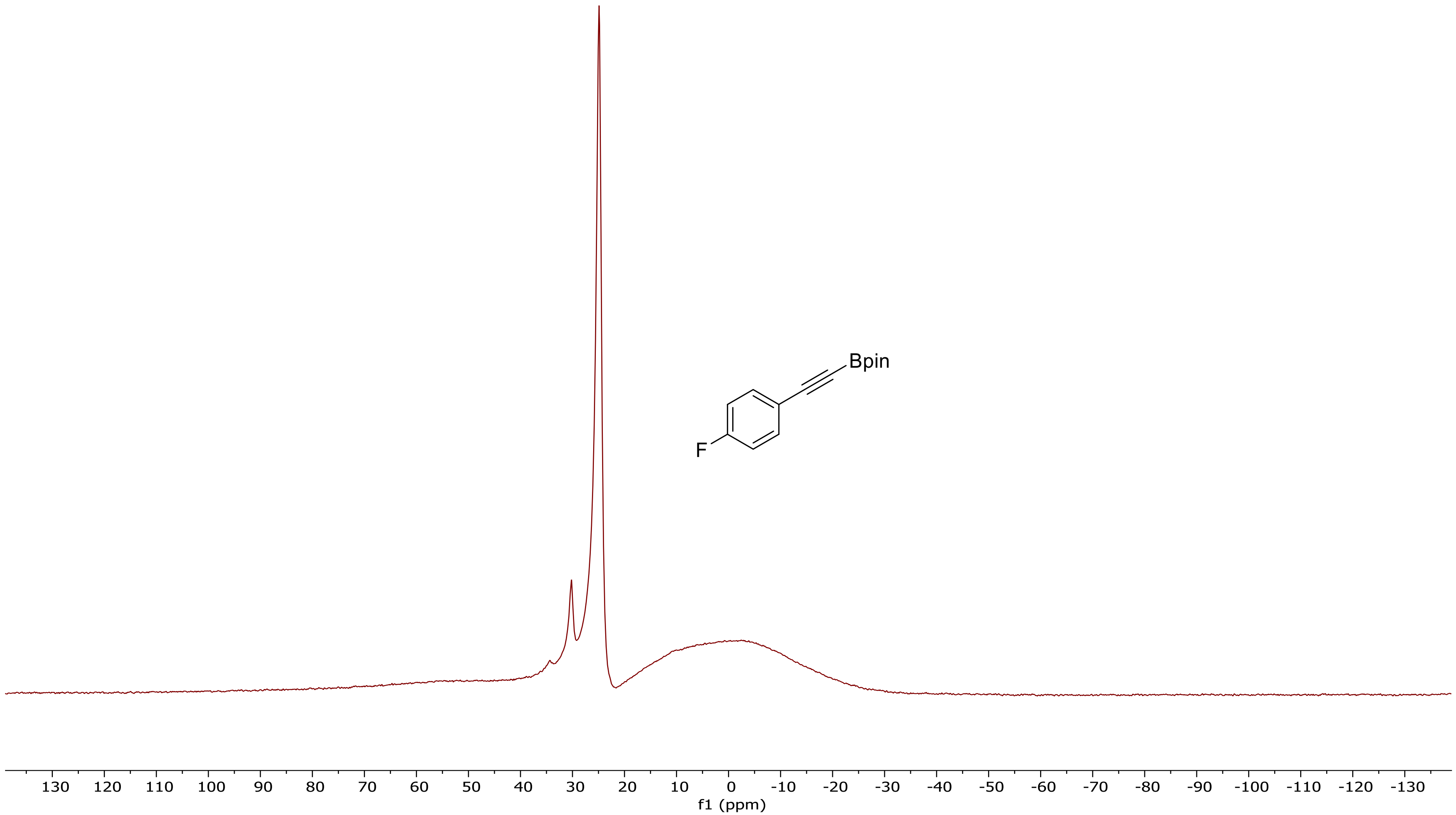

^{11}B NMR (C_6D_6, 128.34 MHz) of 2-((4-fluorophenyl)ethynyl)-4,4,5,5-tetramethyl-1,3,2-dioxaborolane.

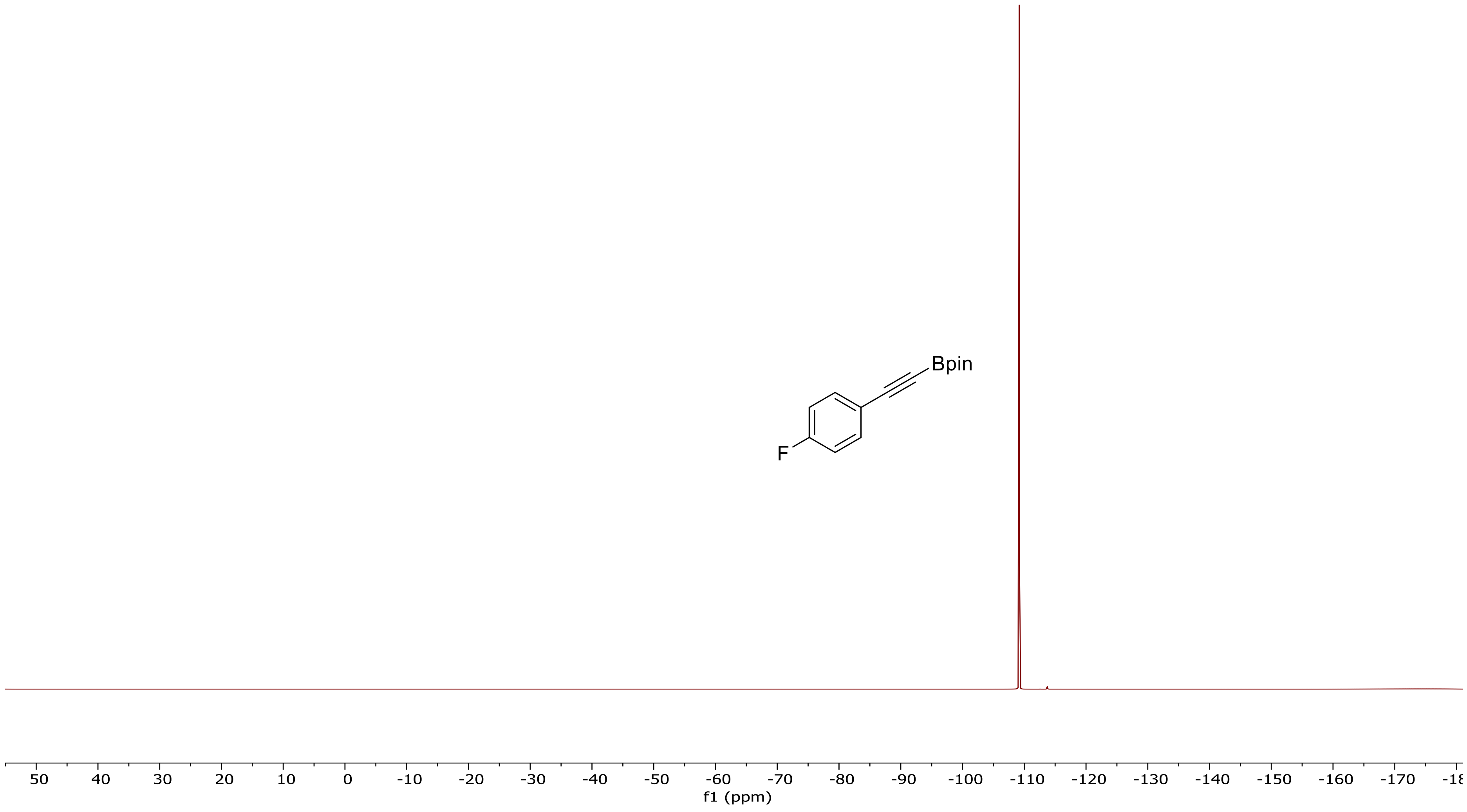

^{19}F NMR (C_6D_6, 470.39 MHz) of 2-((4-fluorophenyl)ethynyl)-4,4,5,5-tetramethyl-1,3,2-dioxaborolane.

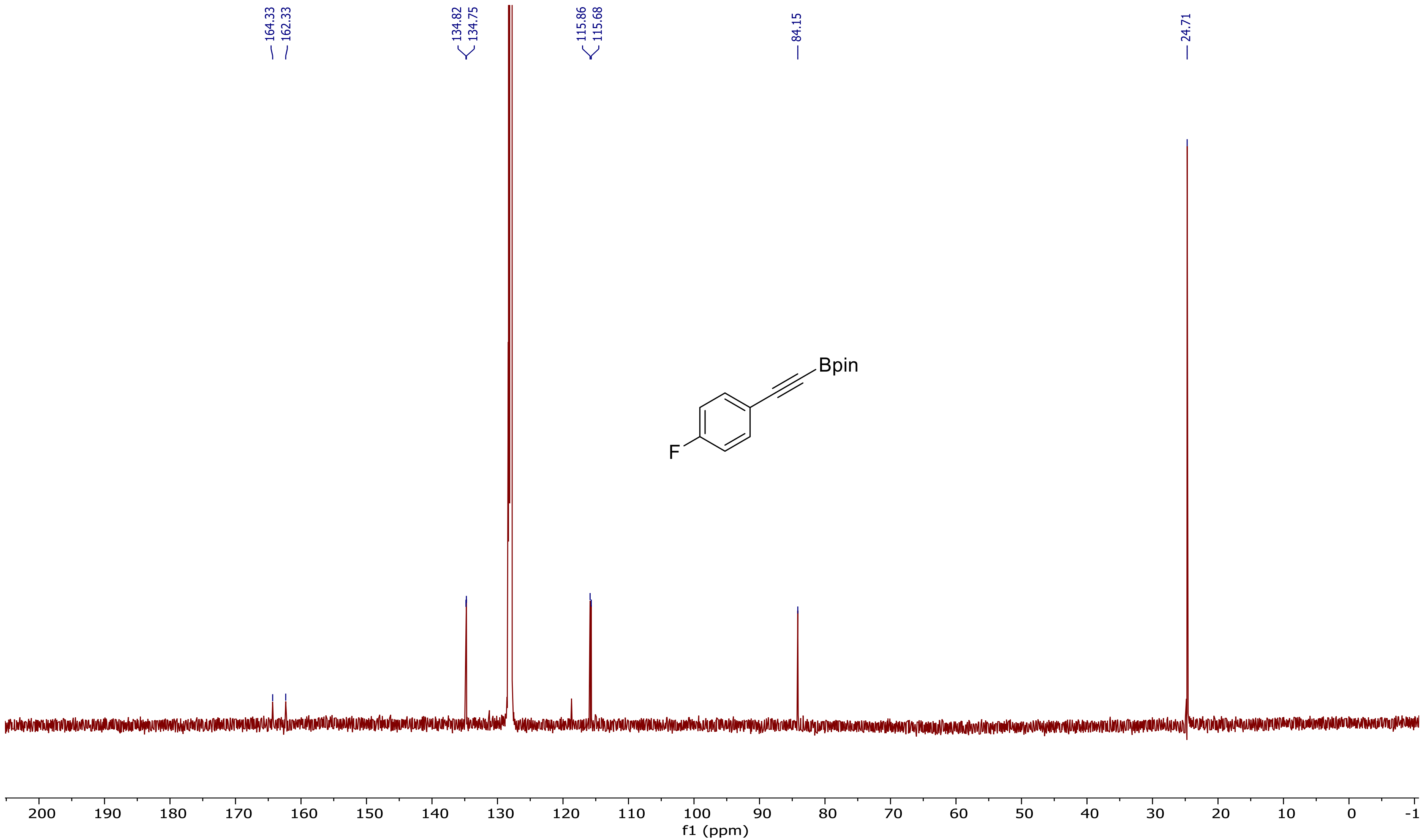

^{13}C NMR (C_6D_6, 125.77 MHz) of 2-((4-fluorophenyl)ethynyl)-4,4,5,5-tetramethyl-1,3,2-dioxaborolane.

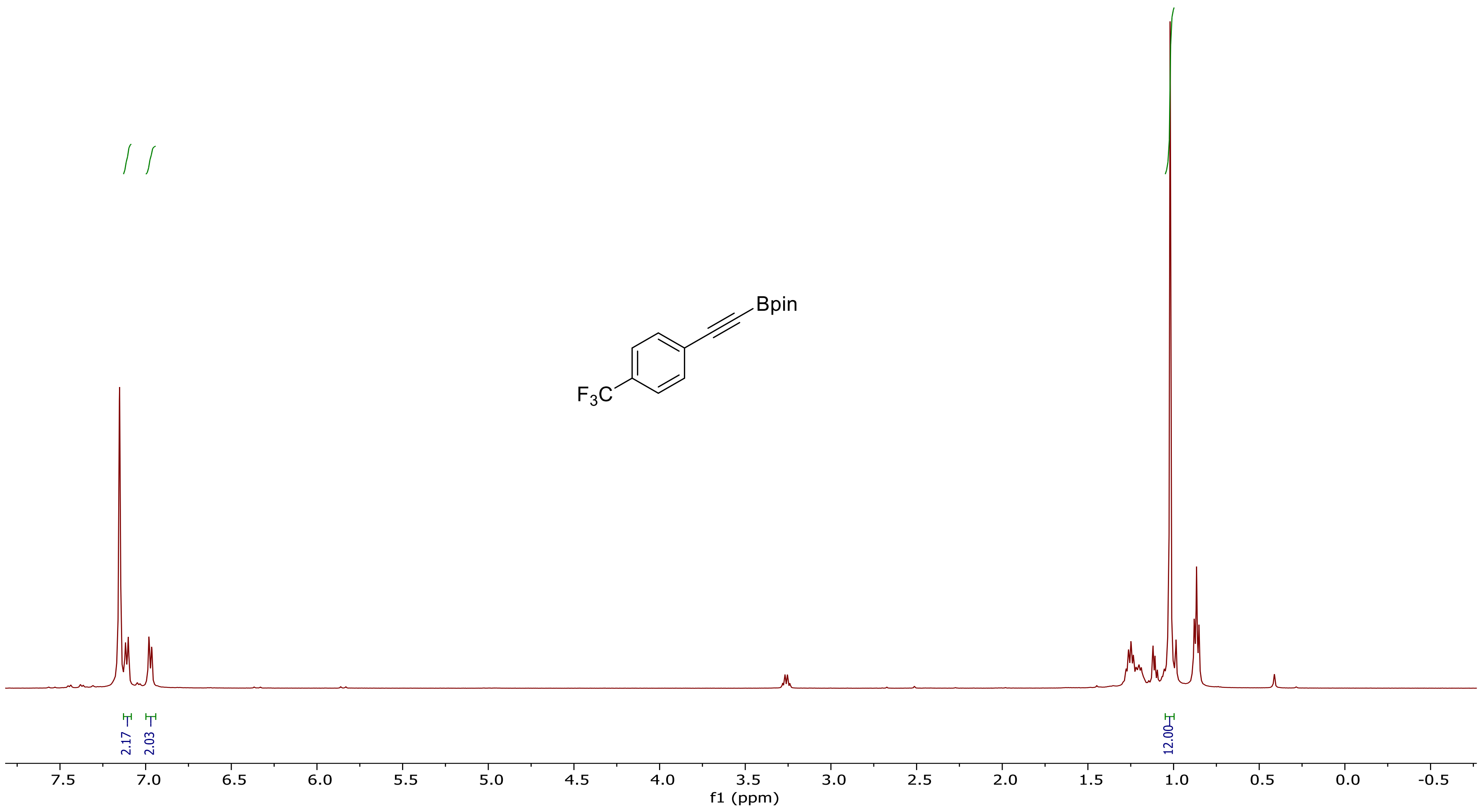

^{1}H NMR (C_6D_6, 500.12 MHz) of 4,4,5,5-Tetramethyl-2-((4-(trifluoromethyl)phenyl)ethynyl)-1,3,2-dioxaborolane.

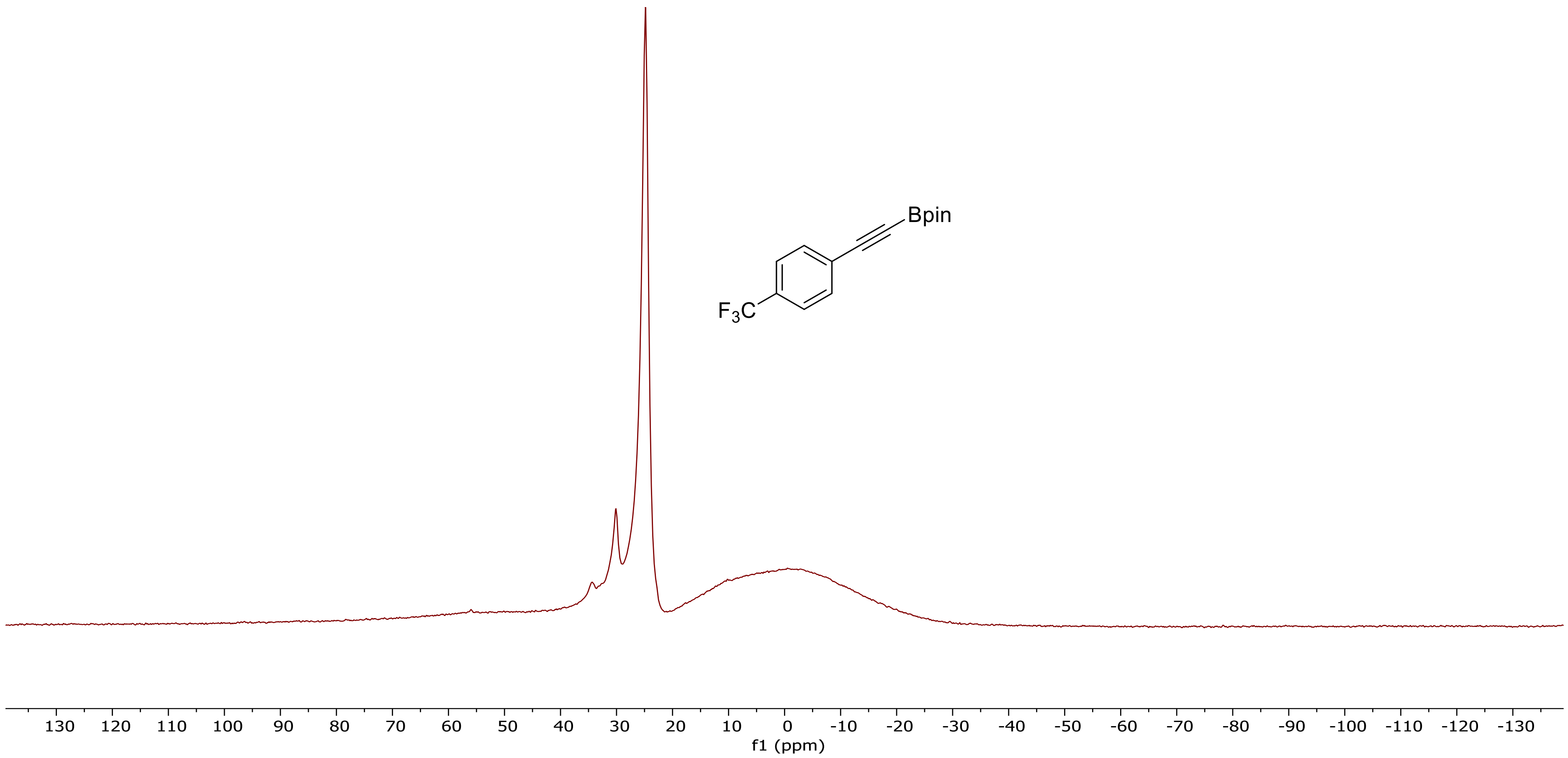

^{11}B NMR (C_6D_6, 128.34 MHz) of 4,4,5,5-Tetramethyl-2-((4-(trifluoromethyl)phenyl)ethynyl)-1,3,2-dioxaborolane.

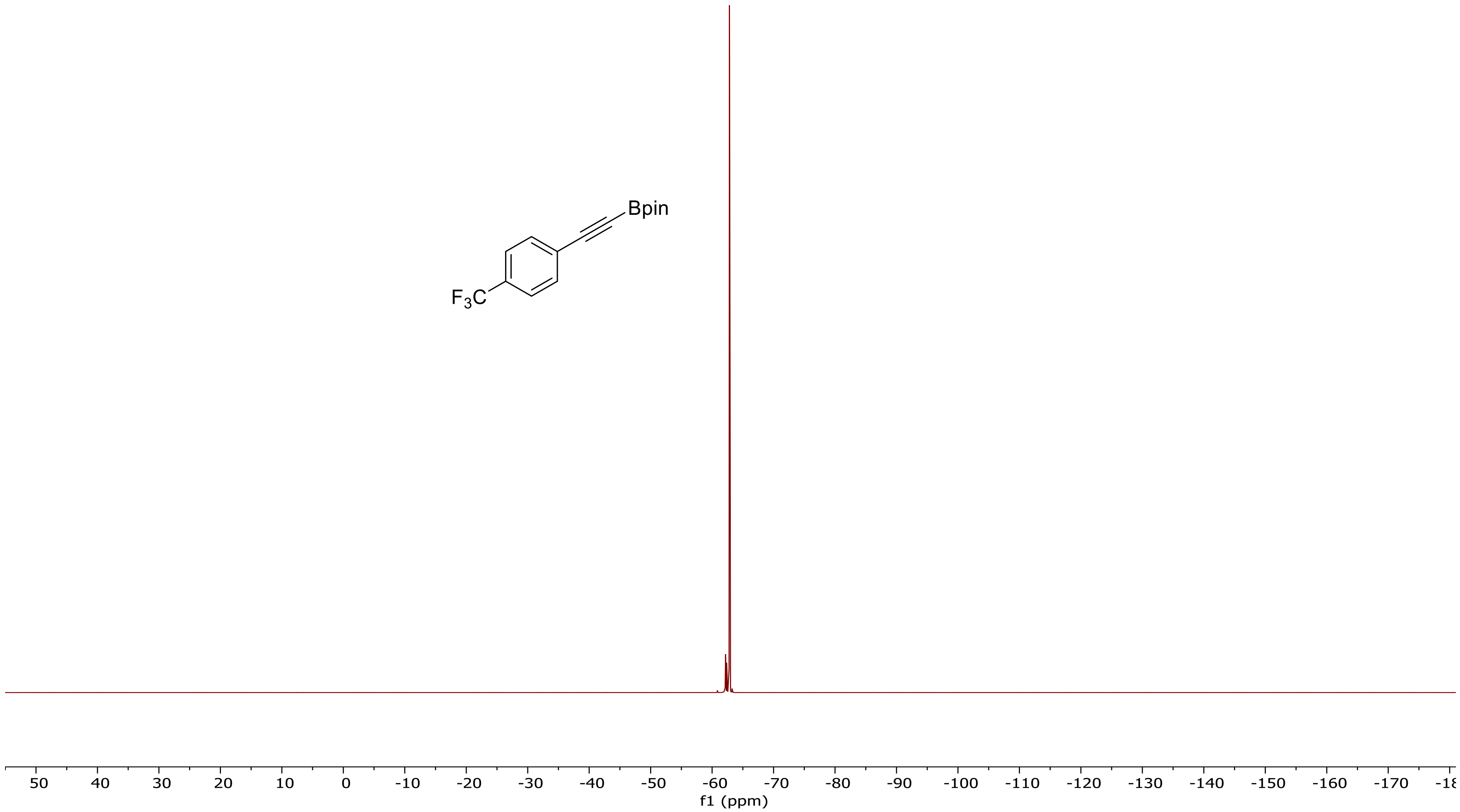

^{19}F NMR (C_6D_6, 470.39 MHz) of 4,4,5,5-Tetramethyl-2-((4-(trifluoromethyl)phenyl)ethynyl)-1,3,2-dioxaborolane.

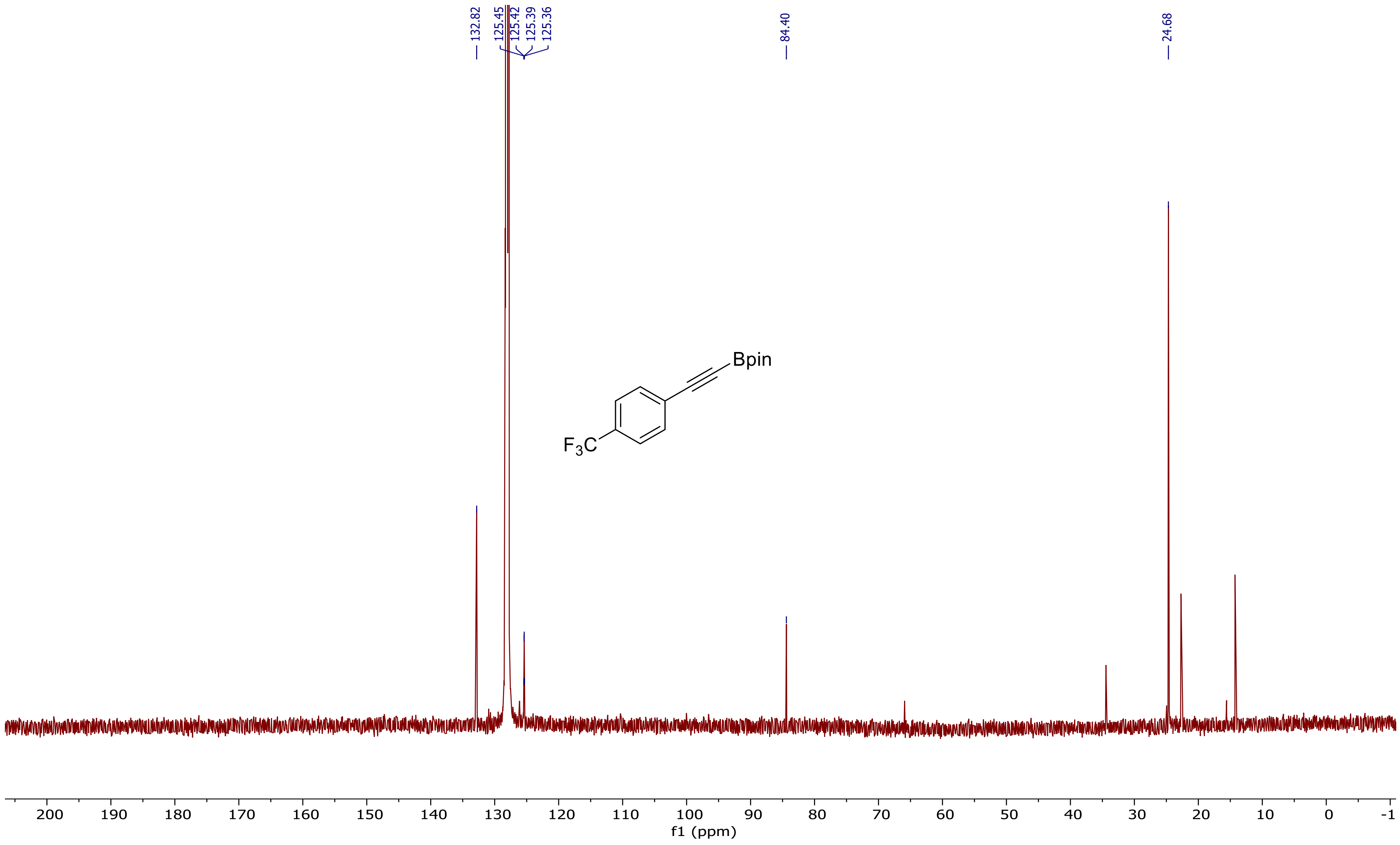

^{13}C NMR (C_6D_6, 125.77 MHz) of 4,4,5,5-Tetramethyl-2-((4-(trifluoromethyl)phenyl)ethynyl)-1,3,2-dioxaborolane.

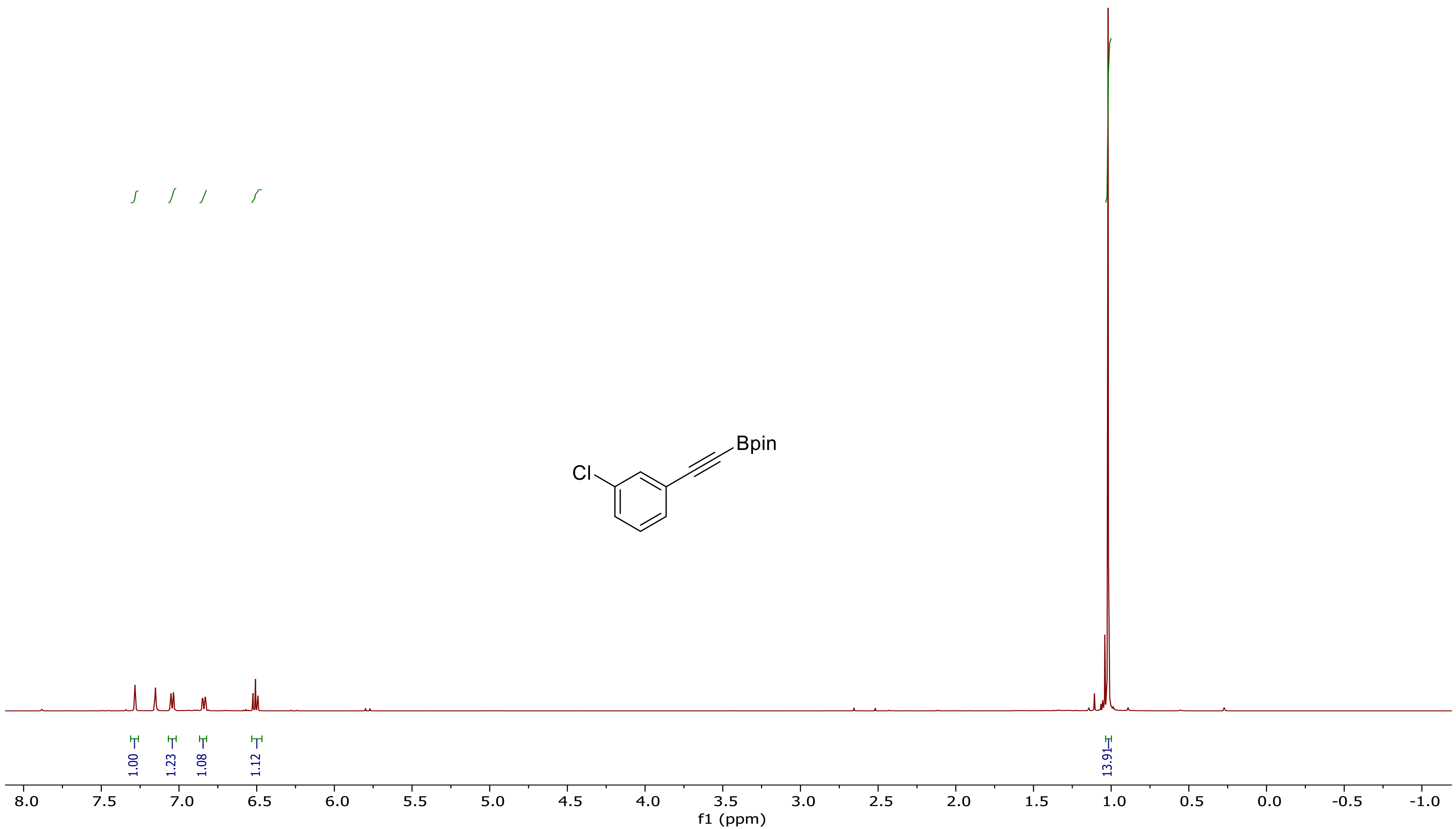

^{1}H NMR (C_6D_6, 500.12 MHz) of 4,4,5,5-Tetramethyl-2-((3-chlorophenyl)ethynyl)-1,3,2-dioxaborolane.

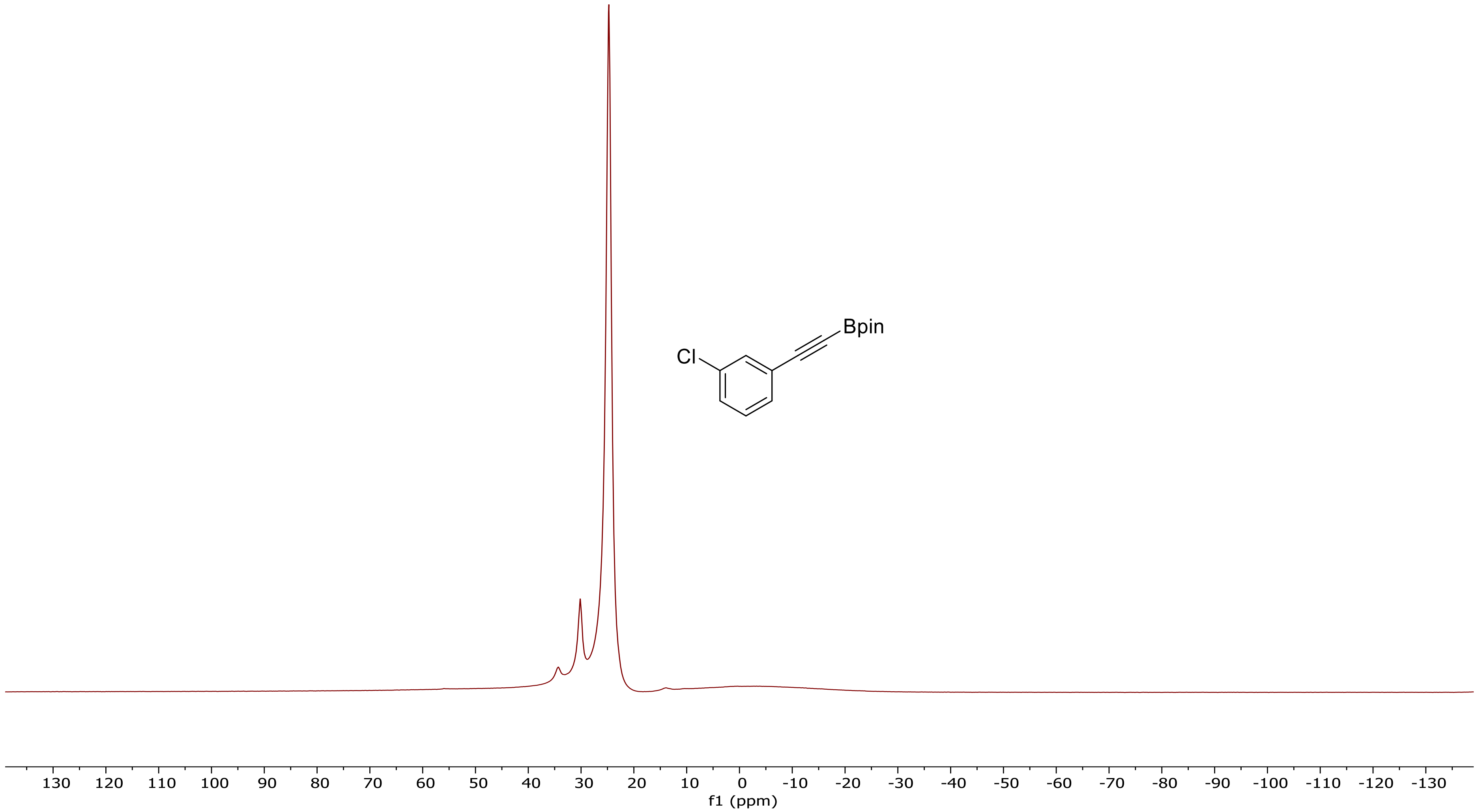

^{11}B NMR (C_6D_6, 128.34 MHz) of 4,4,5,5-Tetramethyl-2-((3-chlorophenyl)ethynyl)-1,3,2-dioxaborolane.

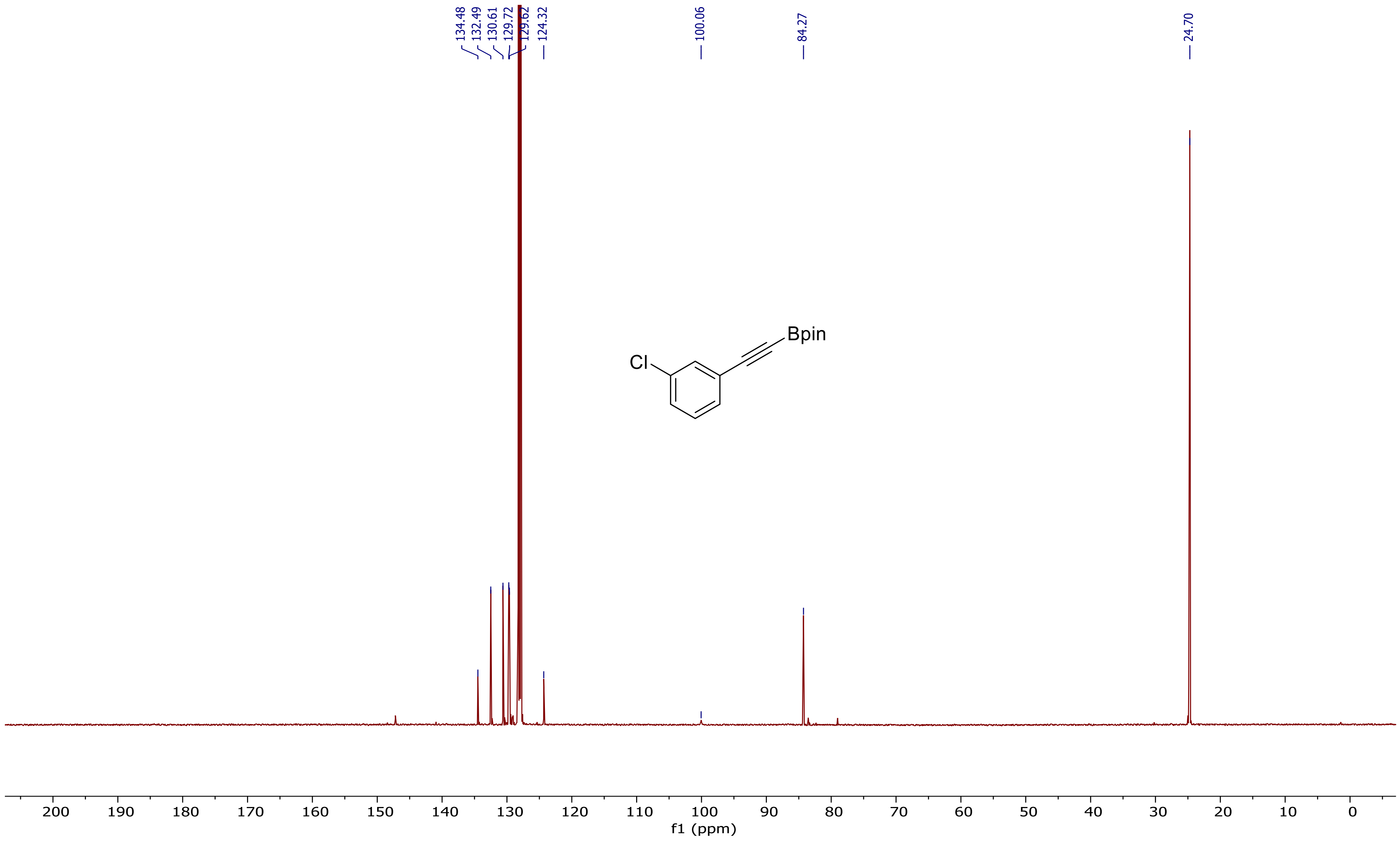

^{13}C NMR (C_6D_6, 125.77 MHz) of 4,4,5,5-Tetramethyl-2-((3-chlorophenyl)ethynyl)-1,3,2-dioxaborolane.

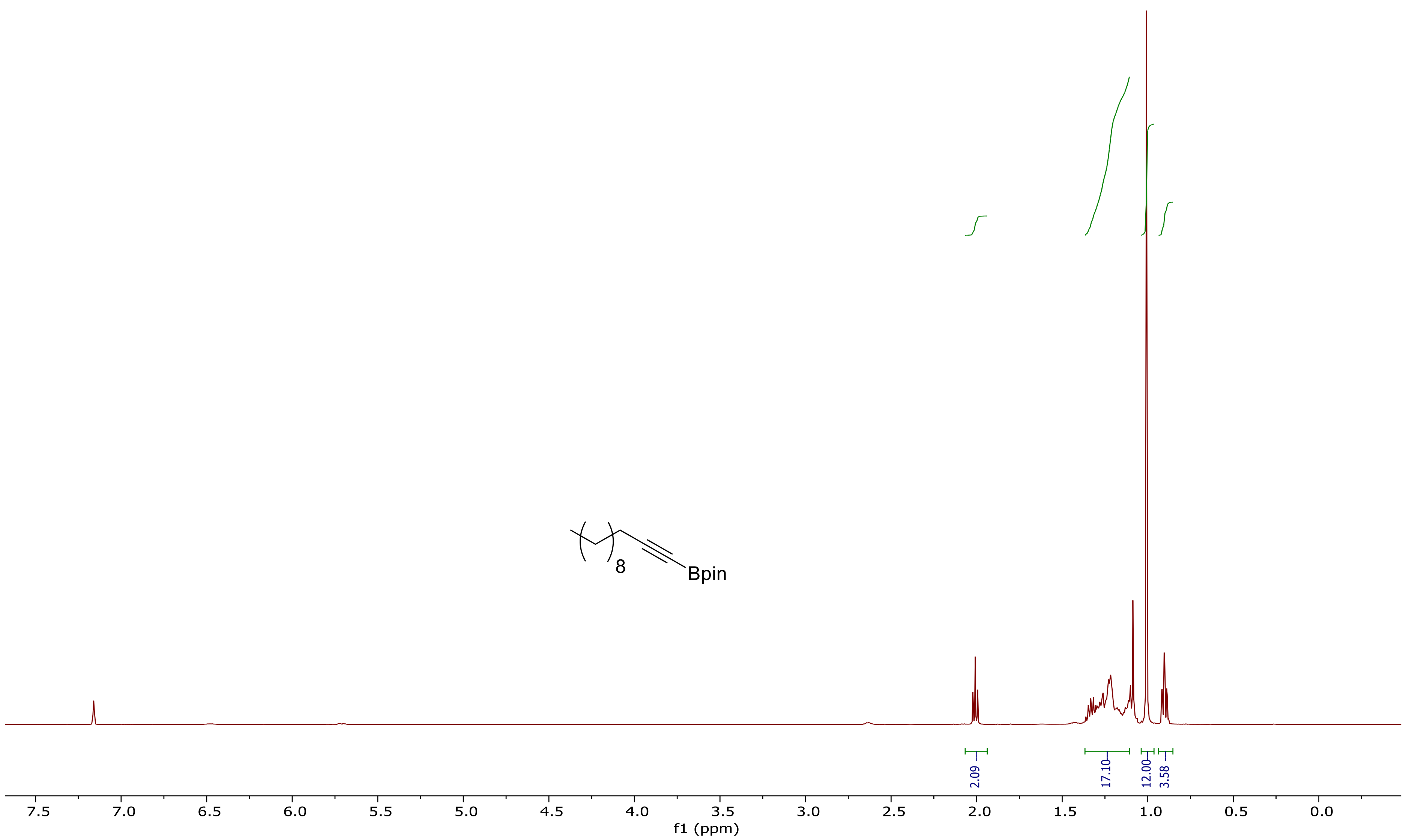

^{1}H NMR (C_6D_6, 500.12 MHz) of 4,4,5,5-Tetramethyl-2-(1-dodec-1-yn-1-yl)-1,3,2-dioxaborolane.

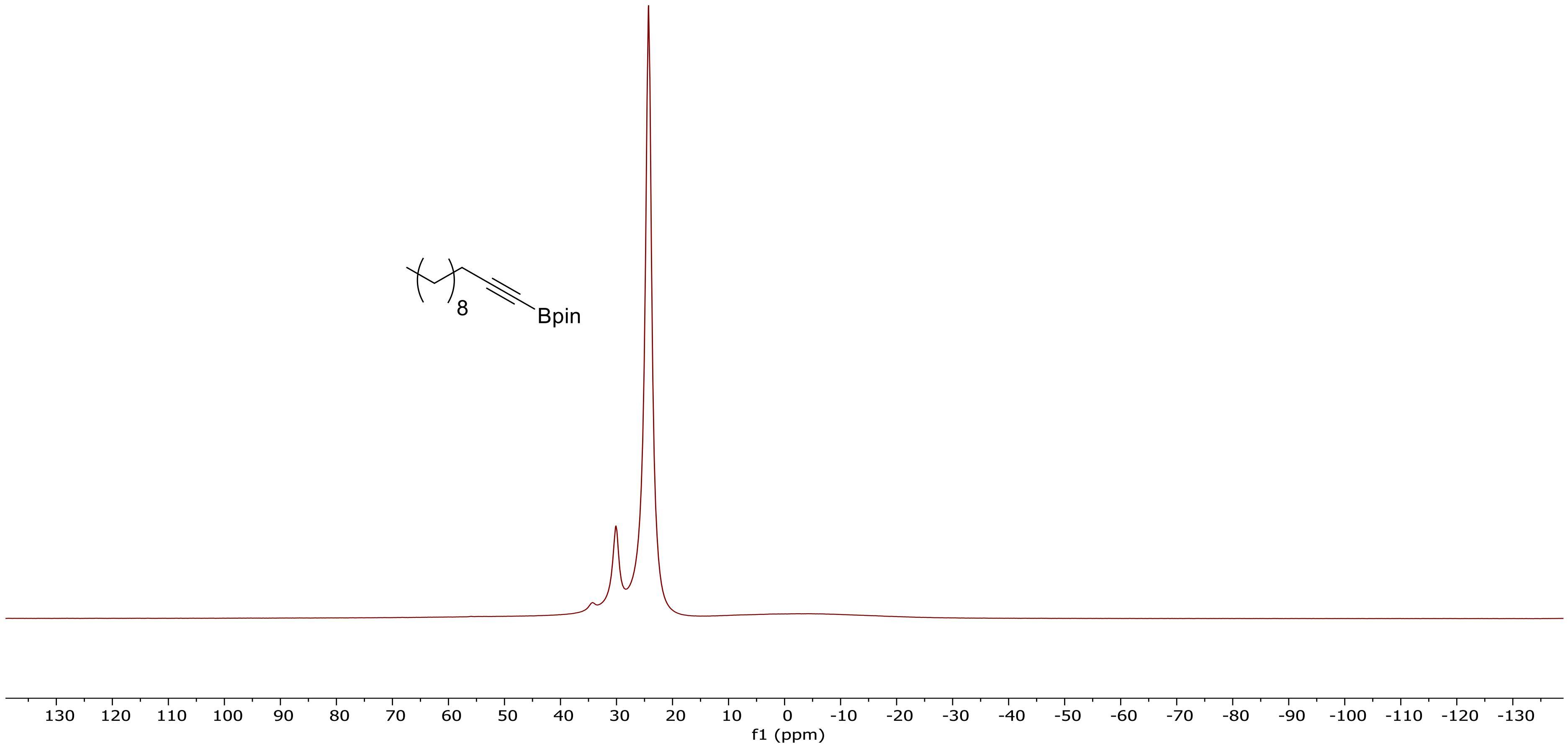

^{11}B NMR (C_6D_6, 128.34 MHz) of 4,4,5,5-Tetramethyl-2-(1-dodec-1-yn-1-yl)-1,3,2-dioxaborolane.

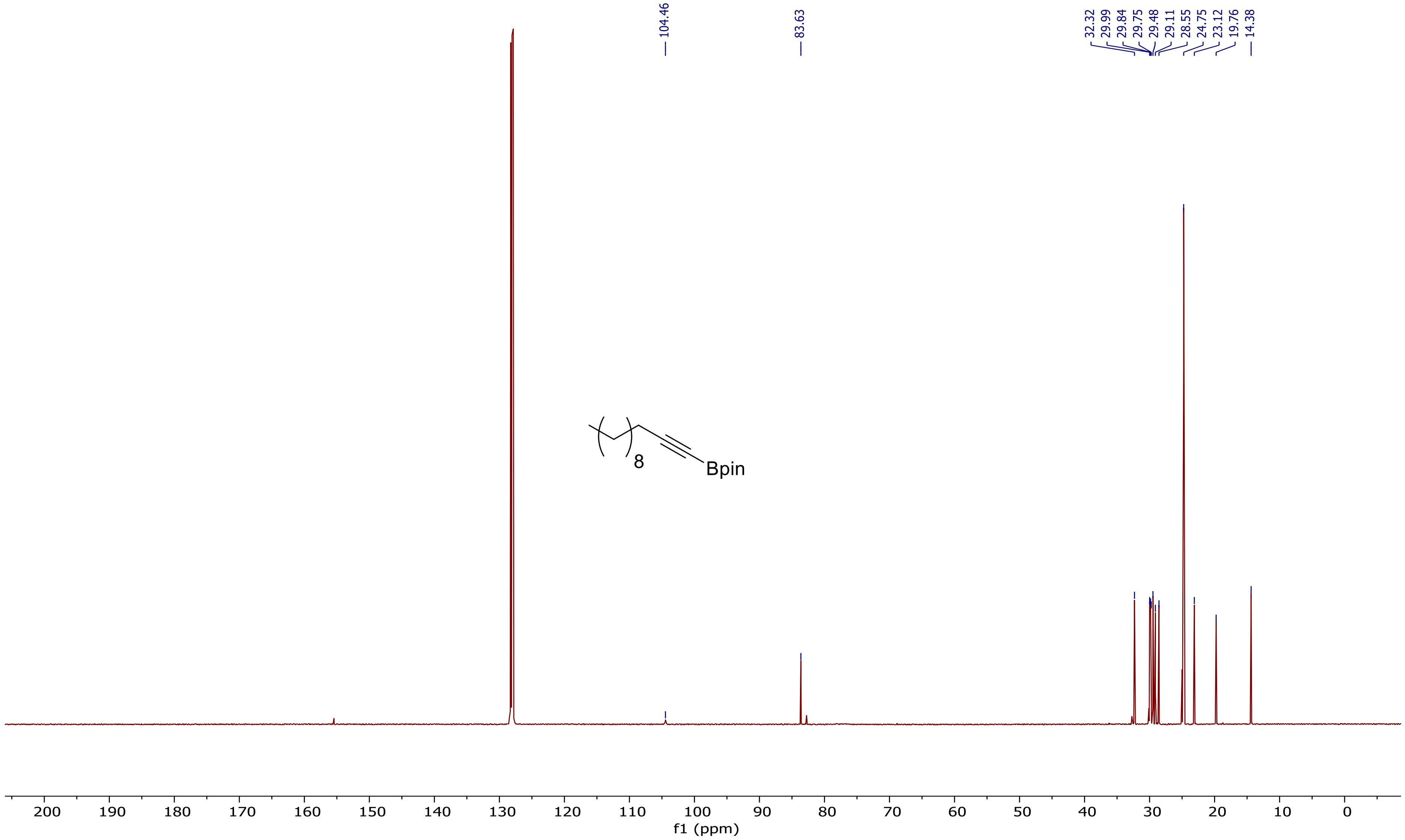

^{13}C NMR (C_6D_6, 125.77 MHz) of 4,4,5,5-Tetramethyl-2-(1-dodec-1-yn-1-yl)-1,3,2-dioxaborolane.

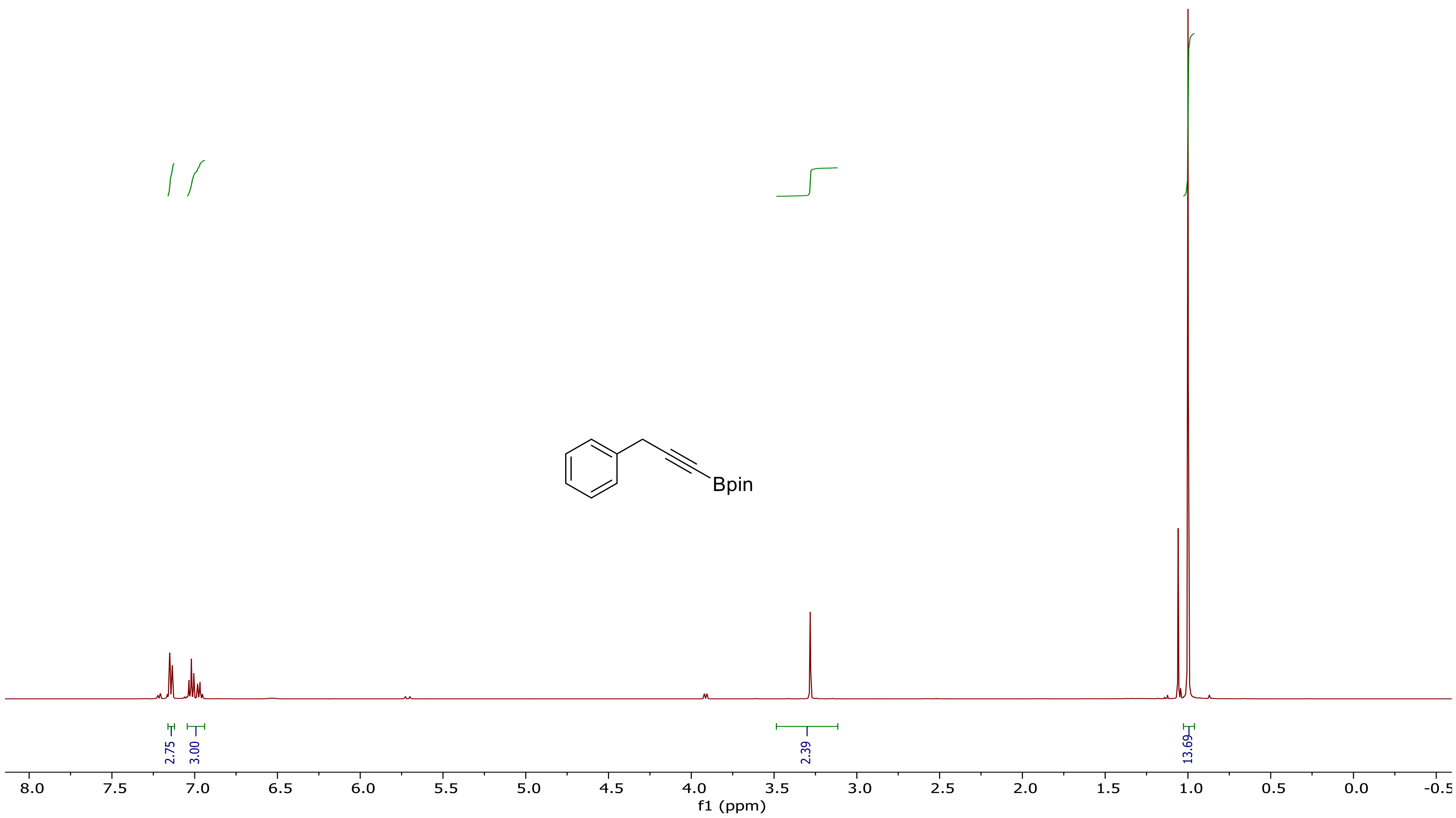

^{1}H NMR (C_6D_6, 500.12 MHz) of 4,4,5,5-Tetramethyl-2-(3-phenylprop-1-yn-1-yl)-1,3,2-dioxaborolane.

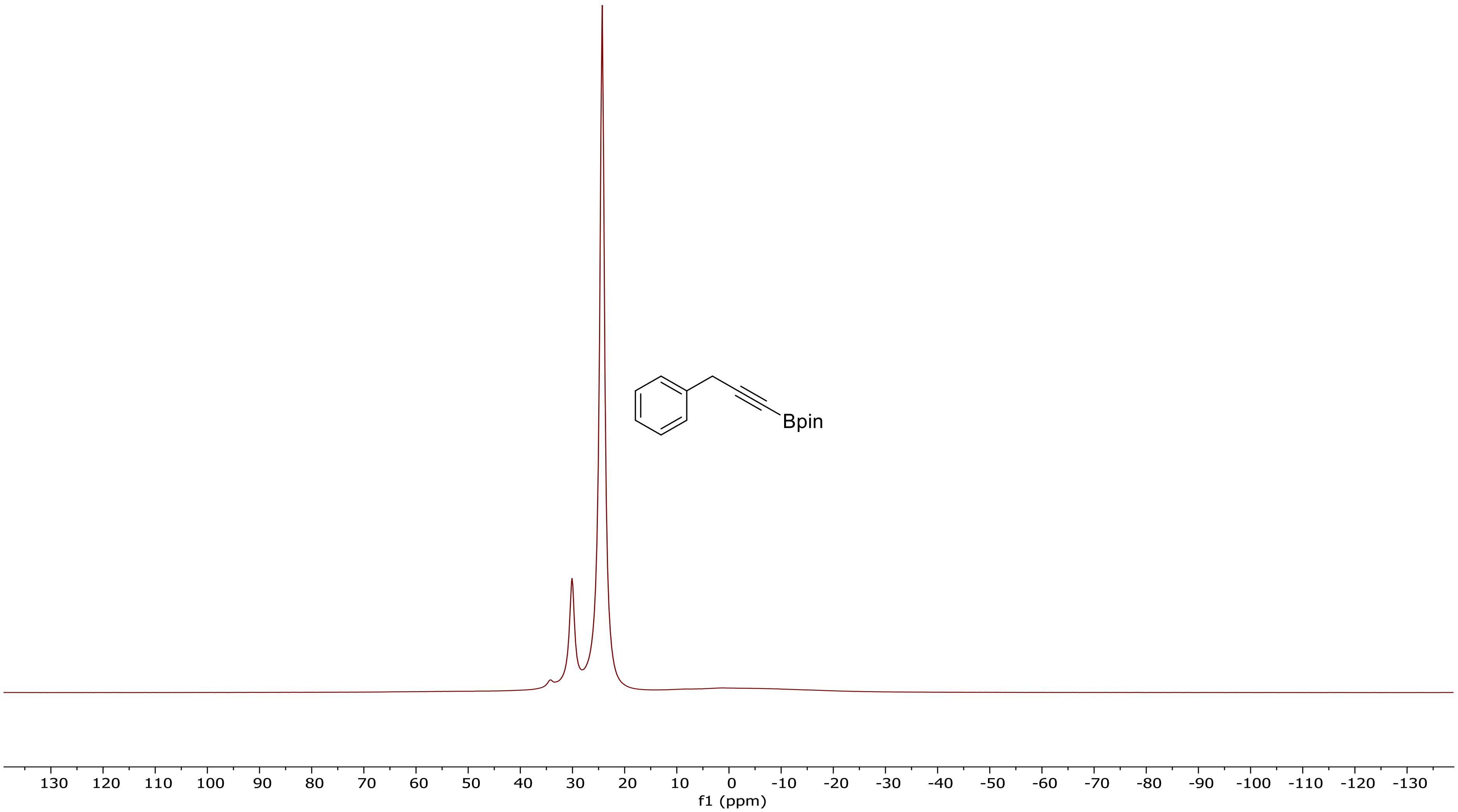

^{11}B NMR (C_6D_6, 128.34 MHz) of 4,4,5,5-Tetramethyl-2-(3-phenylprop-1-yn-1-yl)-1,3,2-dioxaborolane.

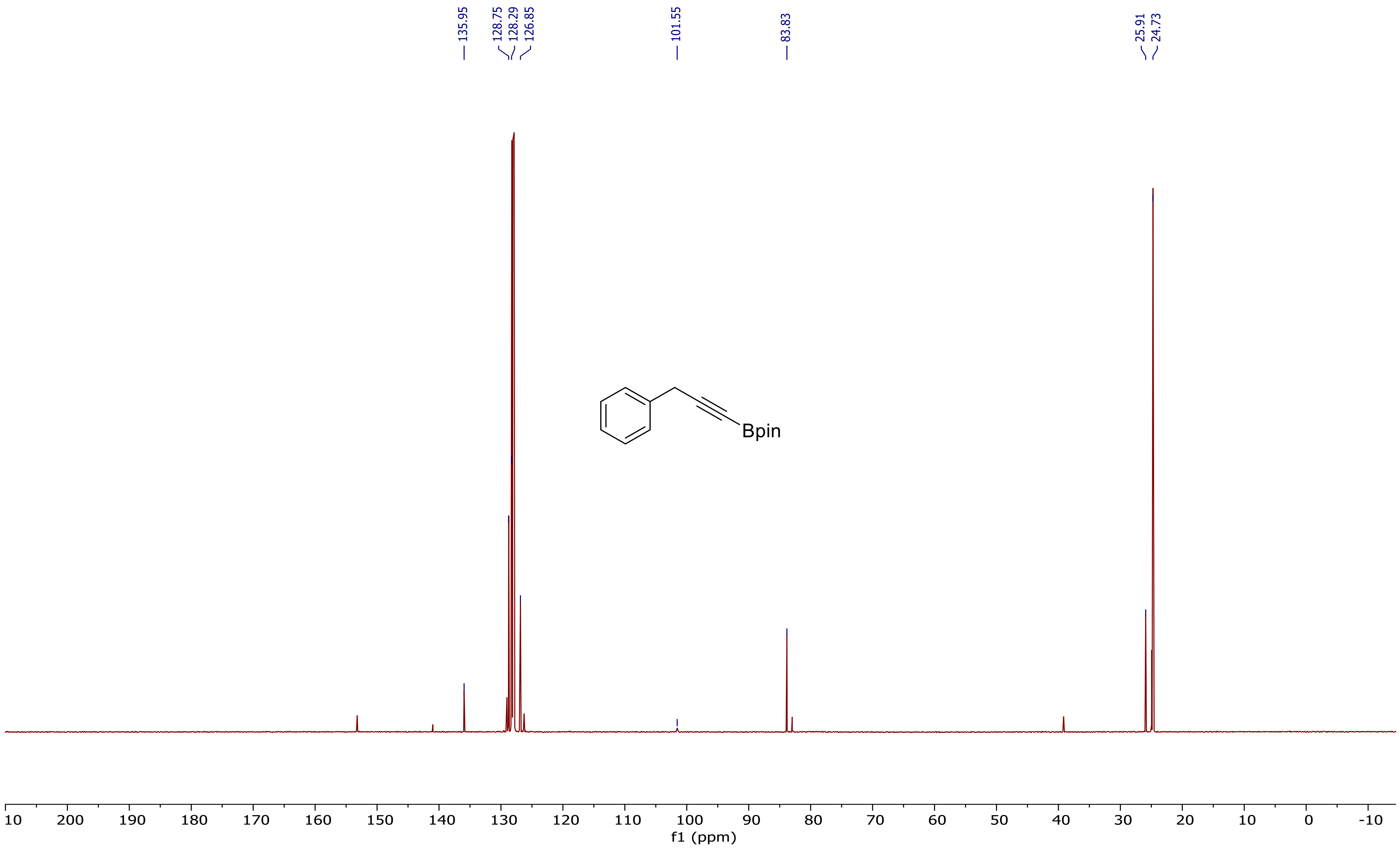

^{13}C NMR (C_6D_6, 125.77 MHz) of 4,4,5,5-Tetramethyl-2-(3-phenylprop-1-yn-1-yl)-1,3,2-dioxaborolane.

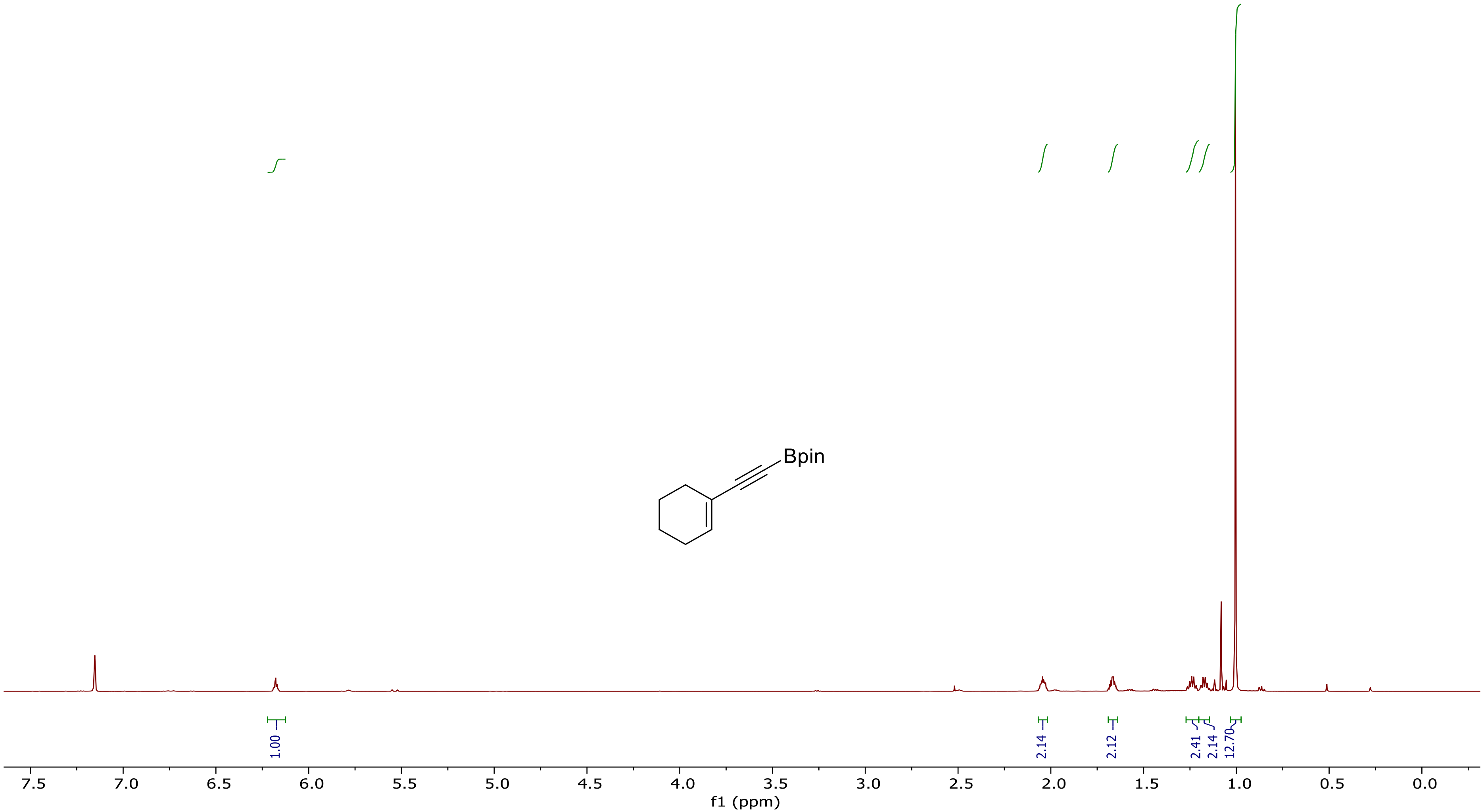

^{1}H NMR (C_6D_6, 500.12 MHz) of 4,4,5,5-Tetramethyl-2-(1-cyclohex-1-enyl)-1,3,2-dioxaborolane.

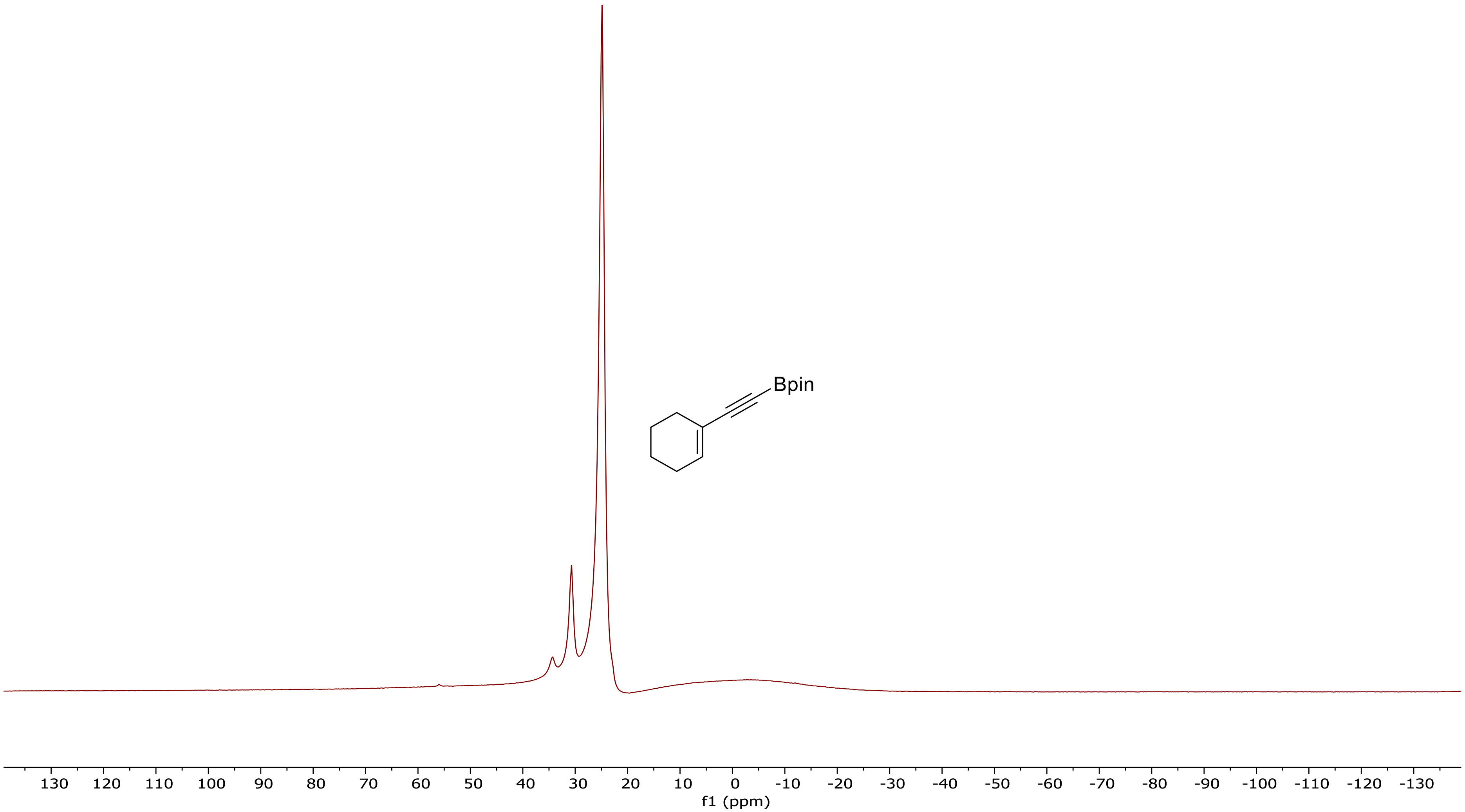

^{11}B NMR (C_6D_6, 128.34 MHz) of 4,4,5,5-Tetramethyl-2-(1-cyclohex-1-enyl)-1,3,2-dioxaborolane.

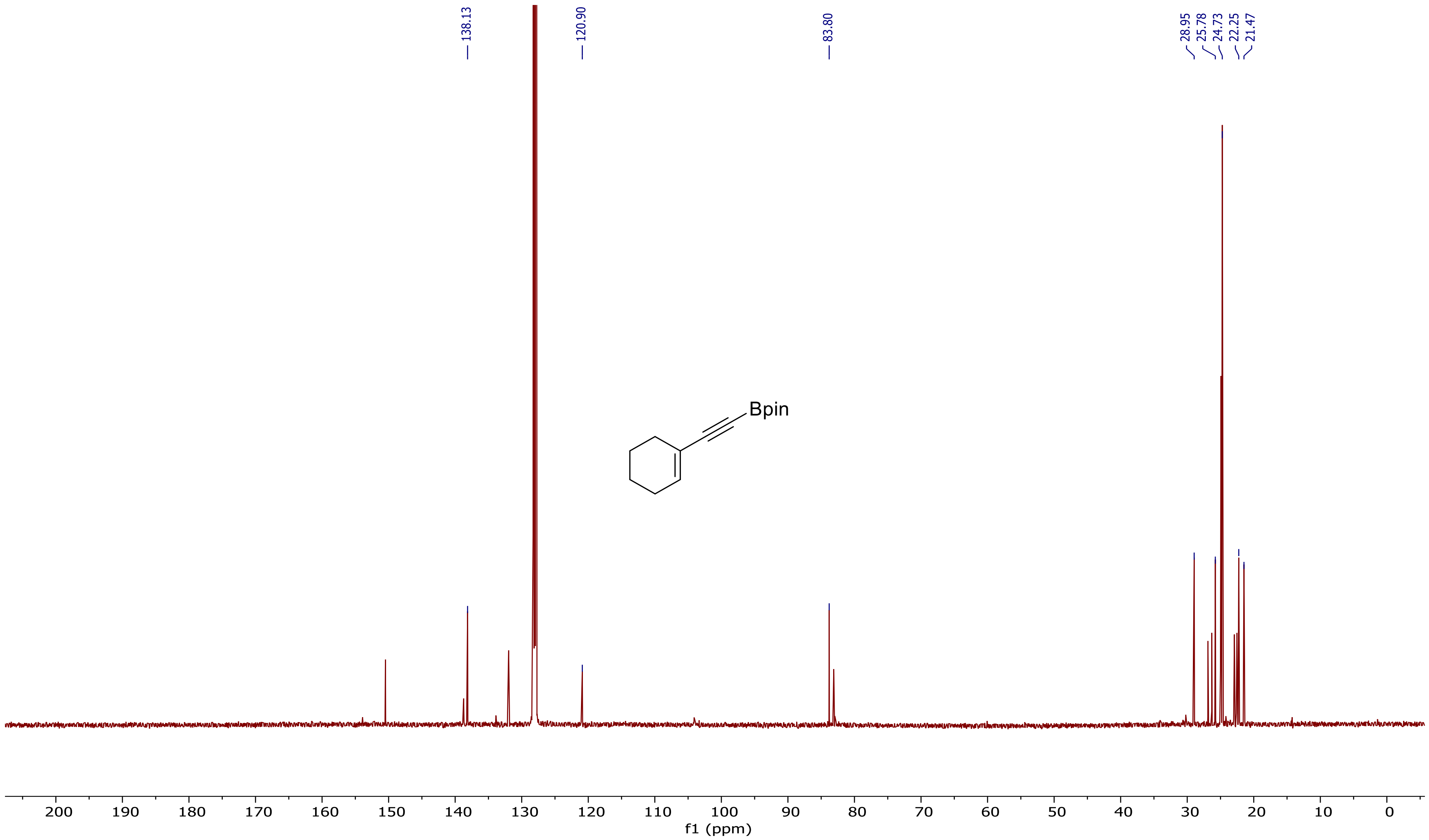

^{13}C NMR (C_6D_6, 125.77 MHz) of 4,4,5,5-Tetramethyl-2-(1-cyclohex-1-enyl)-1,3,2-dioxaborolane.

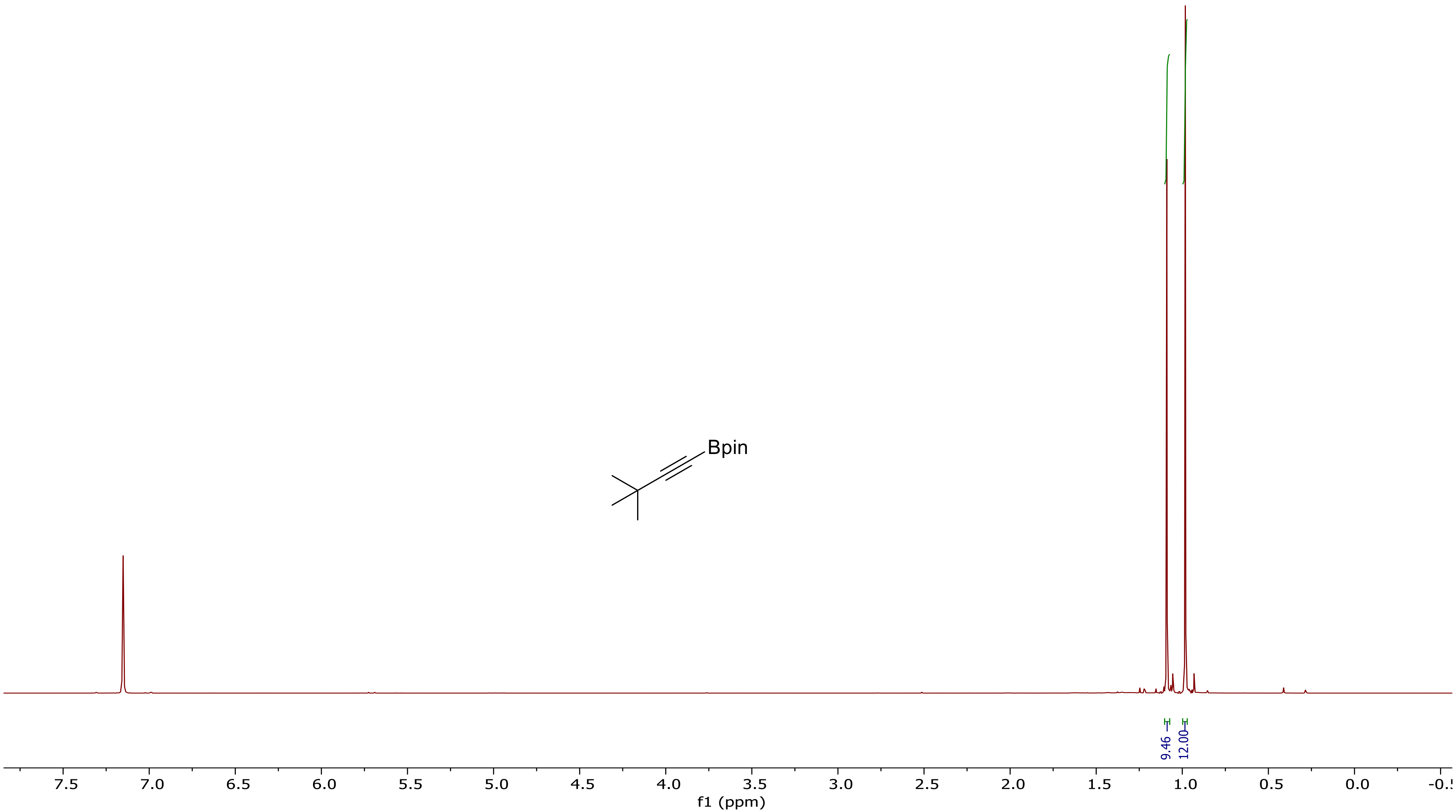

^{1}H NMR (C_6D_6, 500.12 MHz) of 4,4,5,5-Tetramethyl-2-(3,3-dimethylbut-1-yn-1-yl)-1,3,2-dioxaborolane.

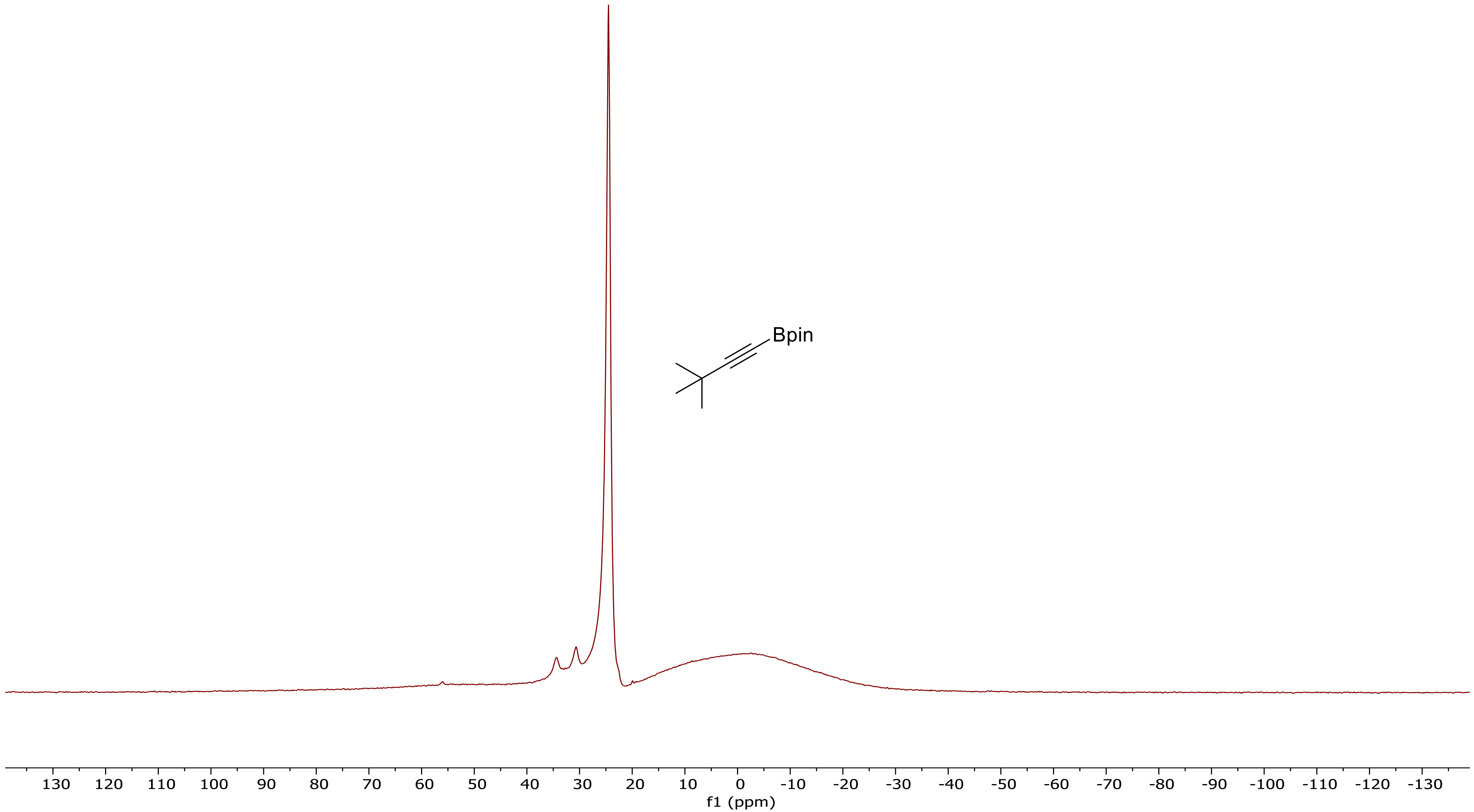

^{11}B NMR (C_6D_6, 128.34 MHz) of 4,4,5,5-Tetramethyl-2-(3,3-dimethylbut-1-yn-1-yl)-1,3,2-dioxaborolane.

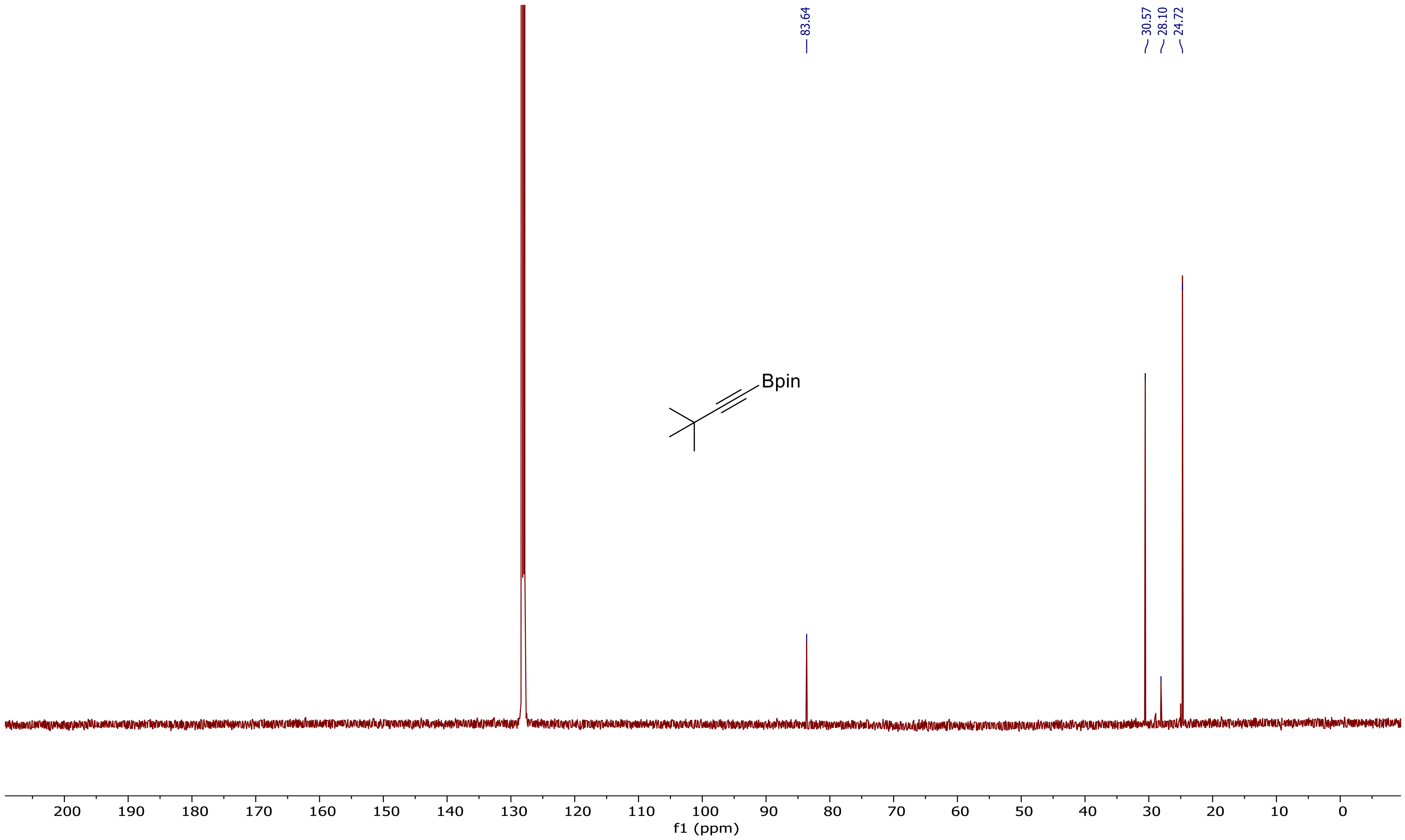

^{13}C NMR (C_6D_6, 125.77 MHz) of 4,4,5,5-Tetramethyl-2-(3,3-dimethylbut-1-yn-1-yl)-1,3,2-dioxaborolane.

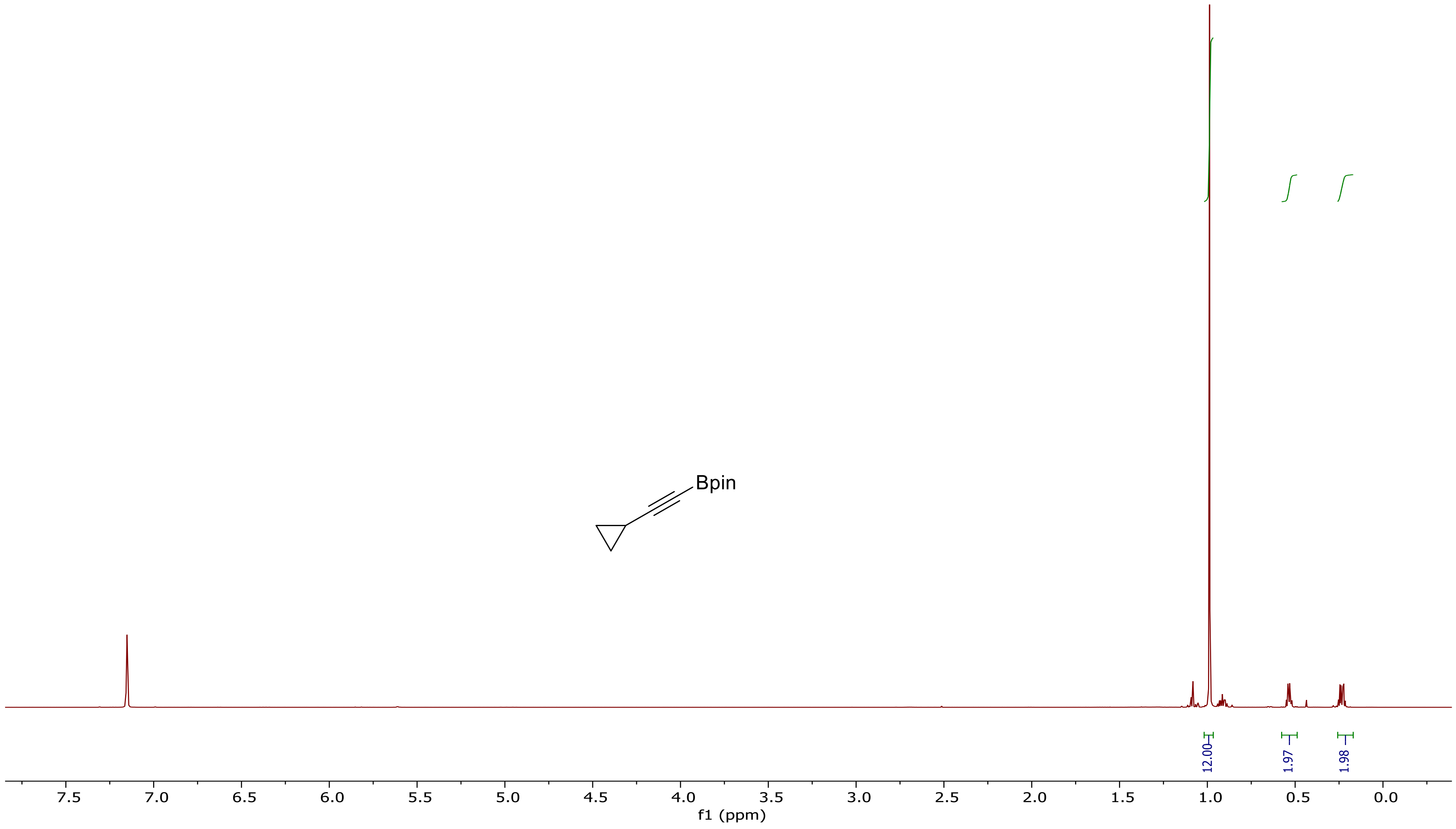

^{1}H NMR (C_6D_6, 500.12 MHz) of 4,4,5,5-Tetramethyl-2-(2-cyclopropyleth-1-yn-1-yl)-1,3,2-dioxaborolane.

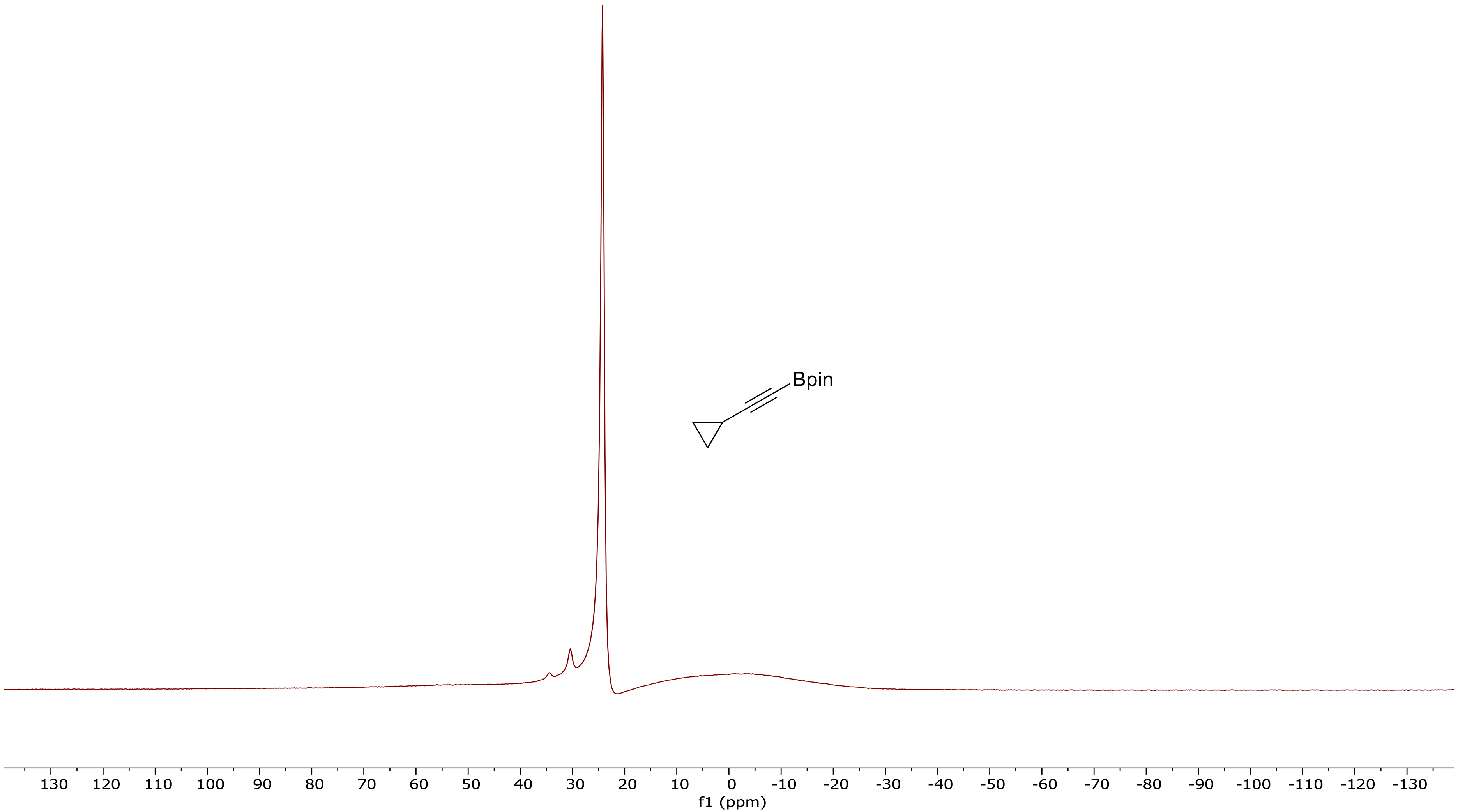

^{11}B NMR (C_6D_6, 128.34 MHz) of 4,4,5,5-Tetramethyl-2-(2-cyclopropyleth-1-yn-1-yl)-1,3,2-dioxaborolane.

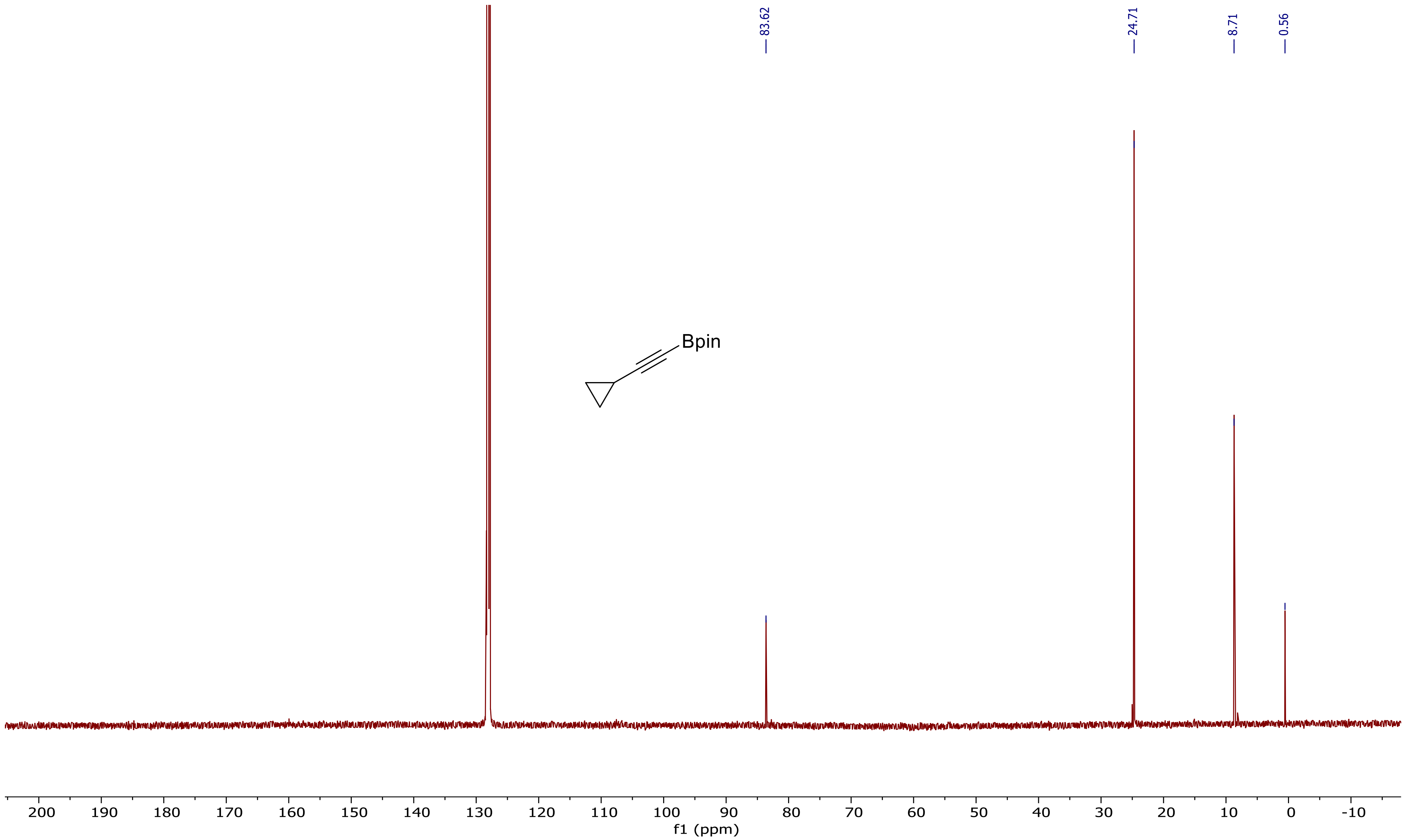

^{13}C NMR (C_6D_6, 125.77 MHz) of 4,4,5,5-Tetramethyl-2-(2-cyclopropyleth-1-yn-1-yl)-1,3,2-dioxaborolane.

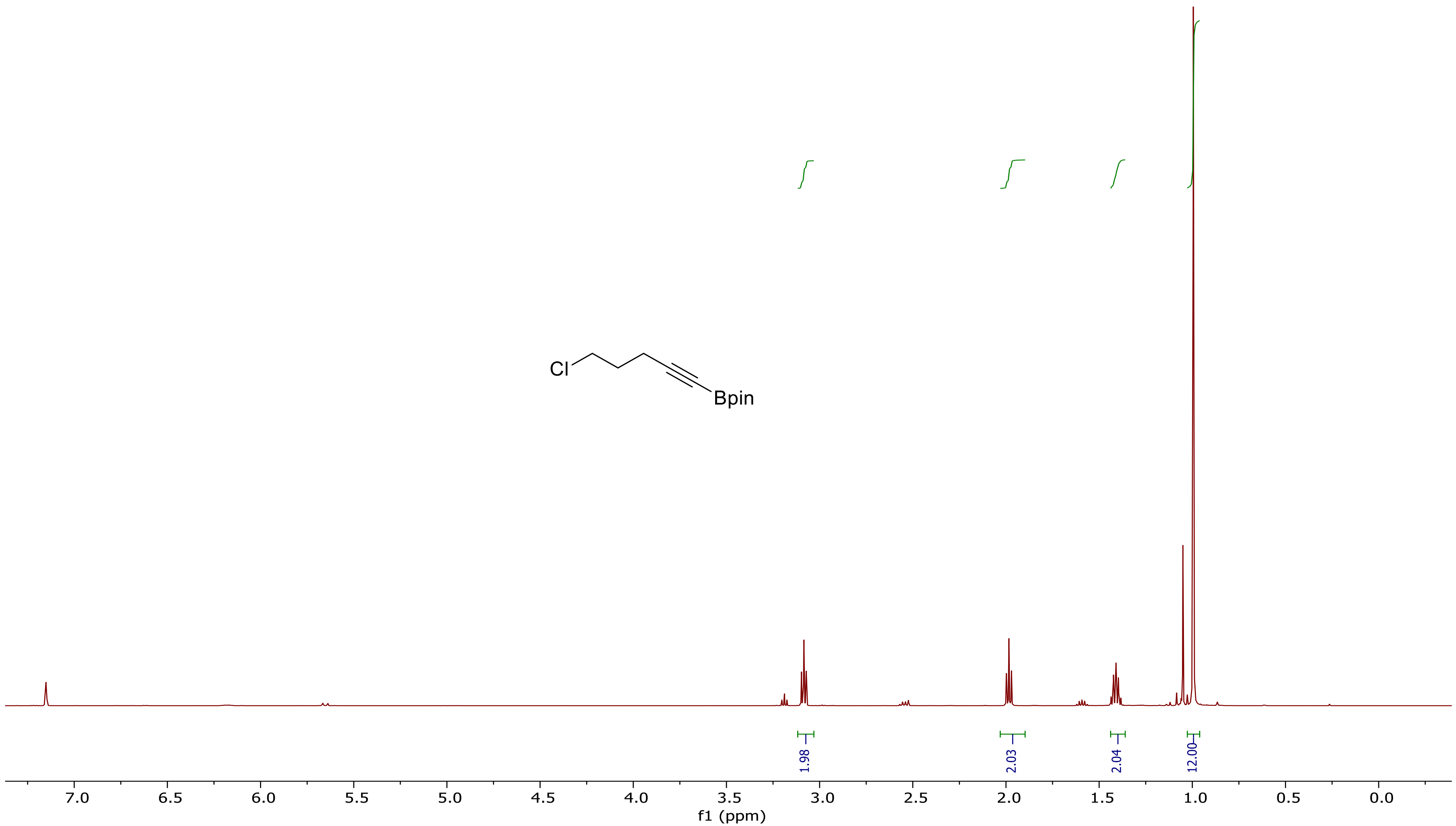

^{1}H NMR (C_6D_6, 500.12 MHz) of 4,4,5,5-Tetramethyl-2-(5-chloropent-1-yn-1-yl)-1,3,2-dioxaborolane.

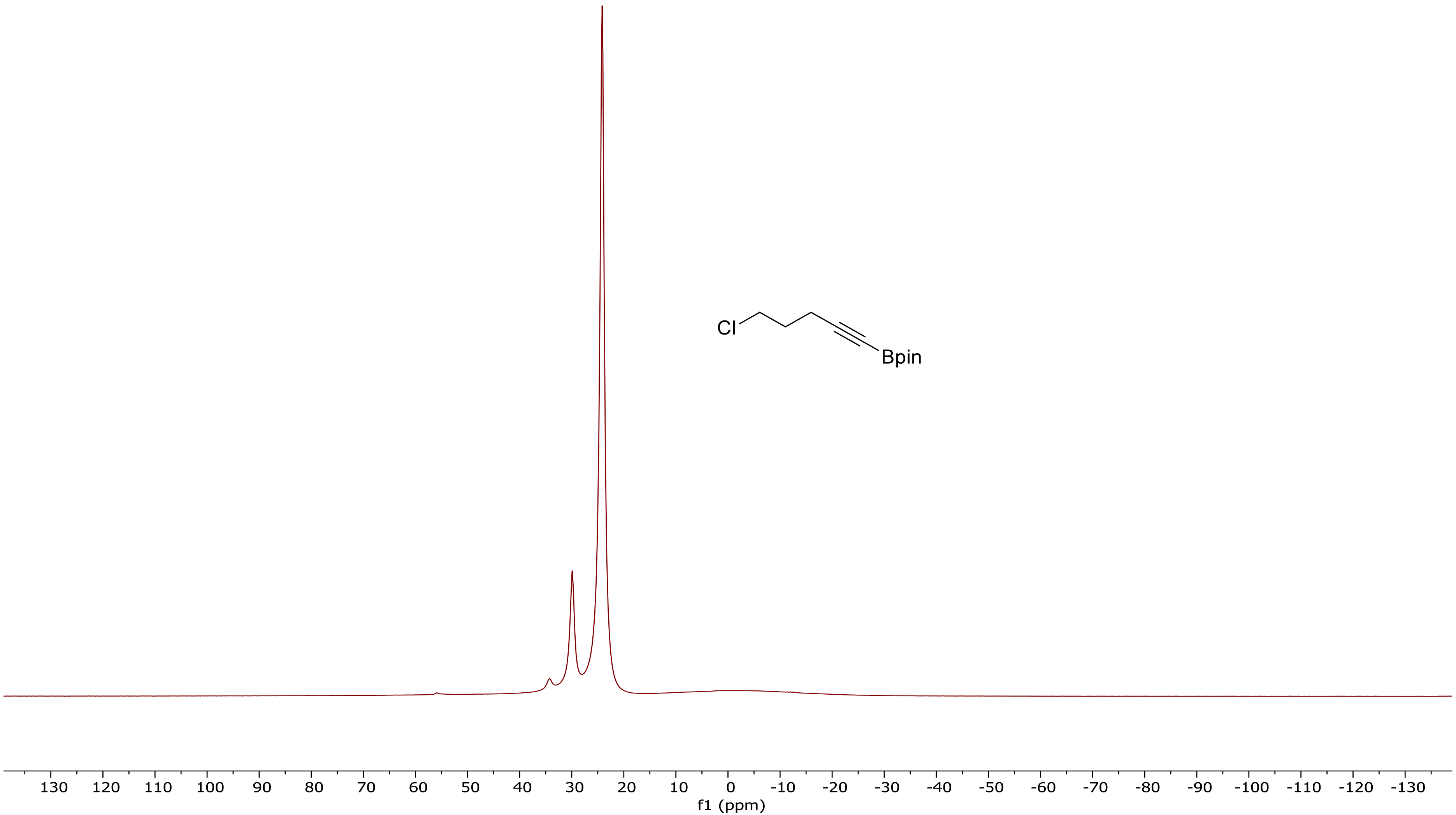

^{11}B NMR (C_6D_6, 128.34 MHz) of 4,4,5,5-Tetramethyl-2-(5-chloropent-1-yn-1-yl)-1,3,2-dioxaborolane.

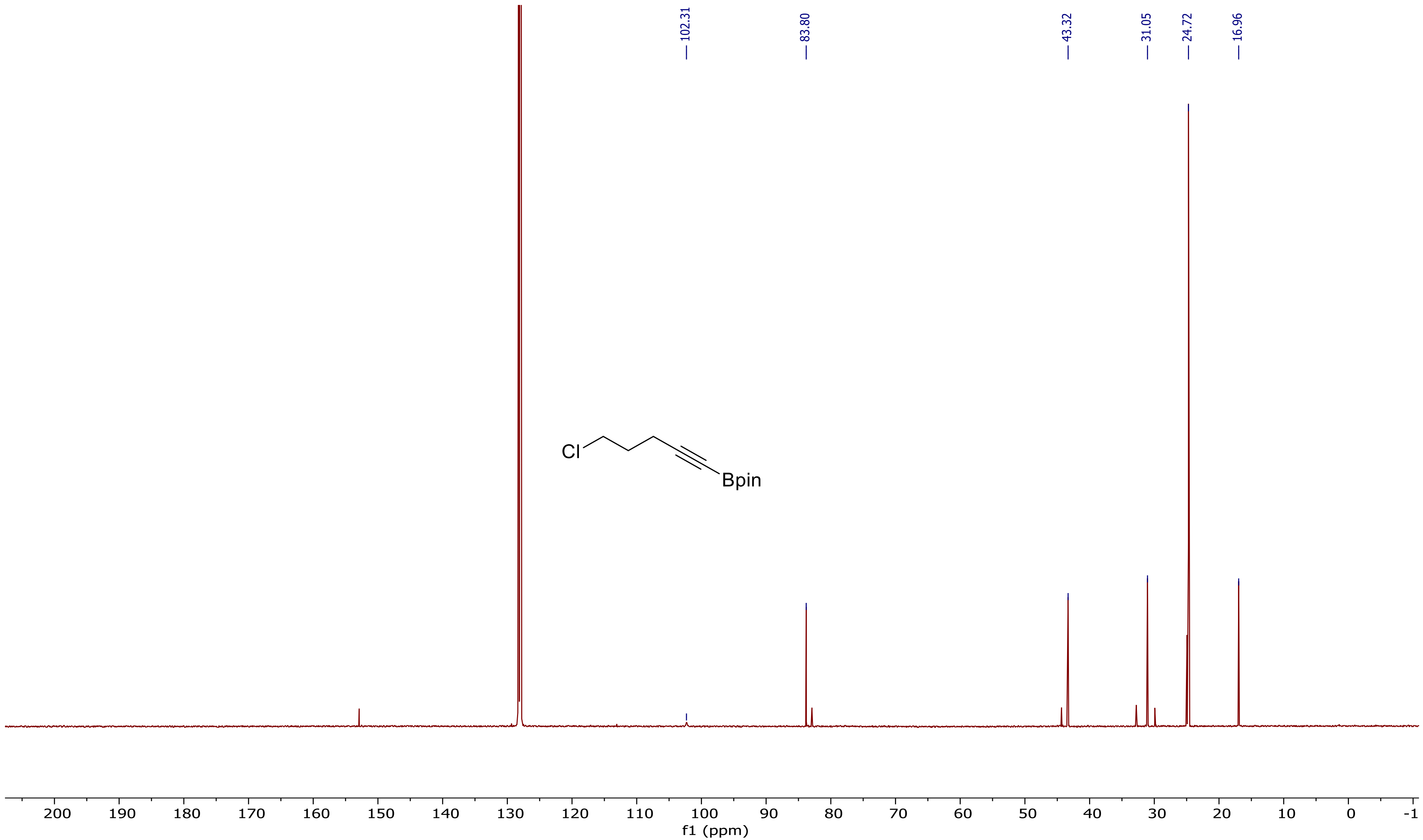

^{13}C NMR (C_6D_6, 125.77 MHz) of 4,4,5,5-Tetramethyl-2-(5-chloropent-1-yn-1-yl)-1,3,2-dioxaborolane.

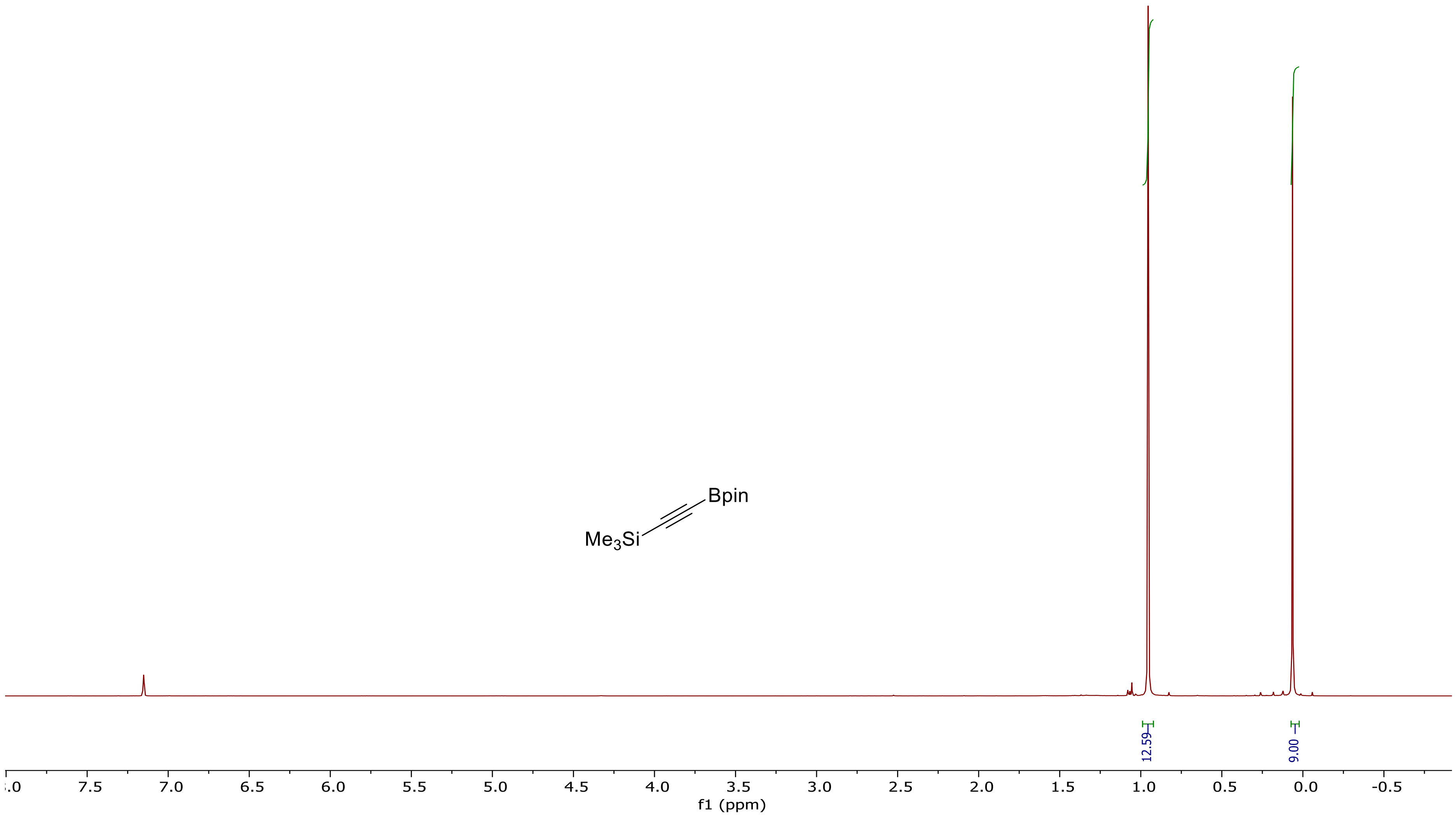

^{1}H NMR (C_6D_6, 500.12 MHz) of 4,4,5,5-Tetramethyl-2-(trimethylsilylethnynyl)-1,3,2-dioxaborolane.

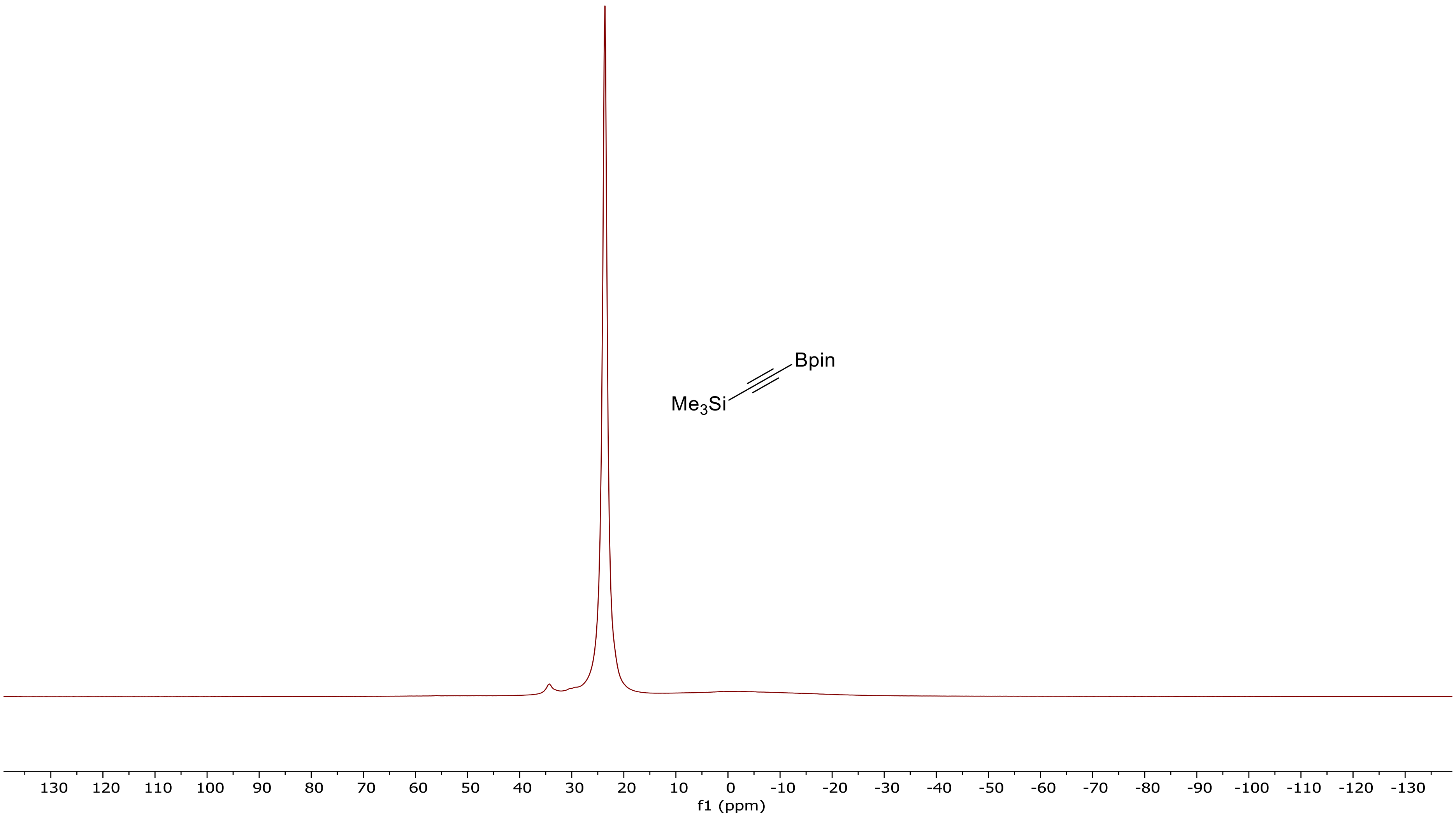

^{11}B NMR (C_6D_6, 128.34 MHz) of 4,4,5,5-Tetramethyl-2-(trimethylsilylethnynyl)-1,3,2-dioxaborolane.

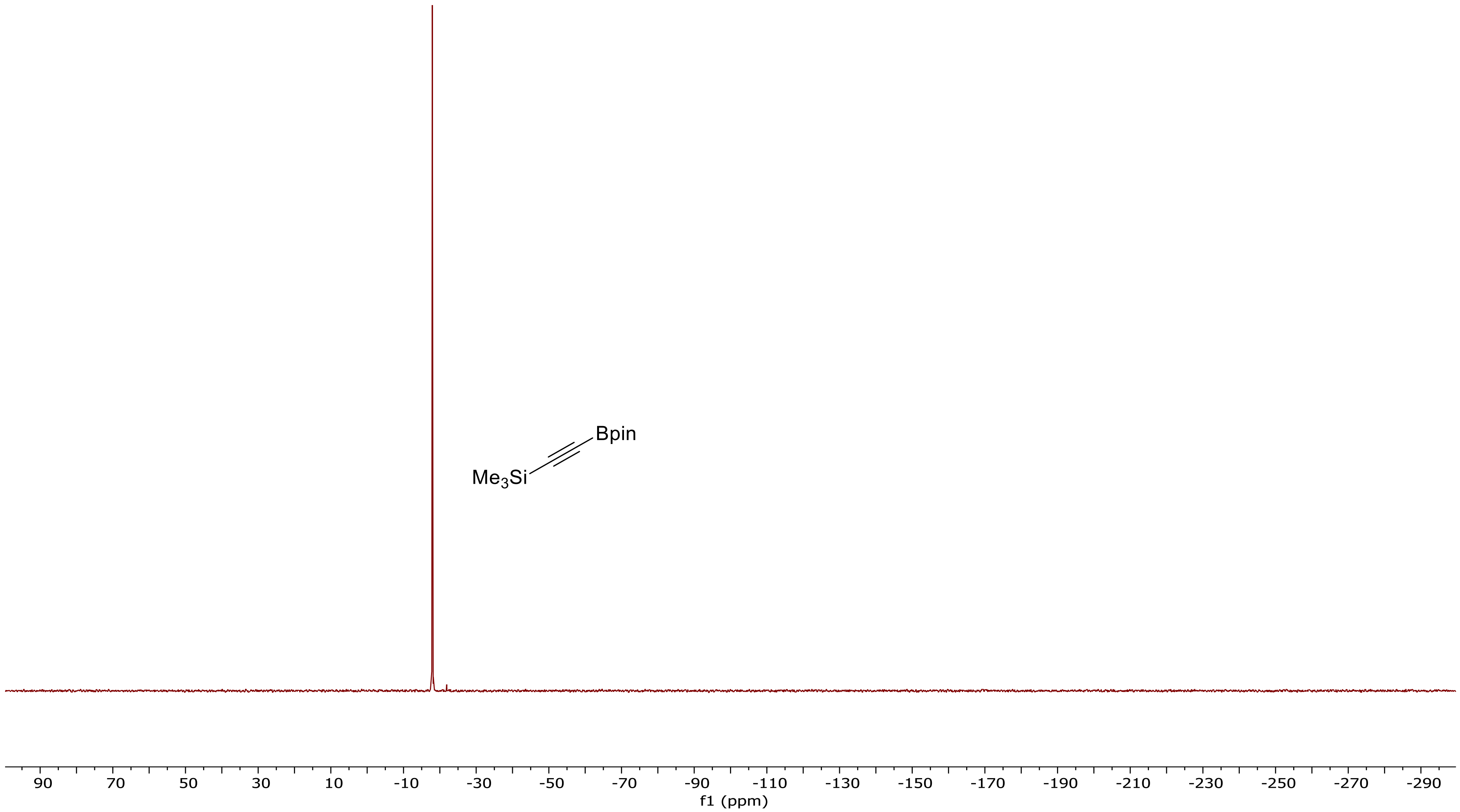

^{29}Si NMR (C_6D_6, 99.33 MHz) of 4,4,5,5-Tetramethyl-2-(trimethylsilylethnynyl)-1,3,2-dioxaborolane.

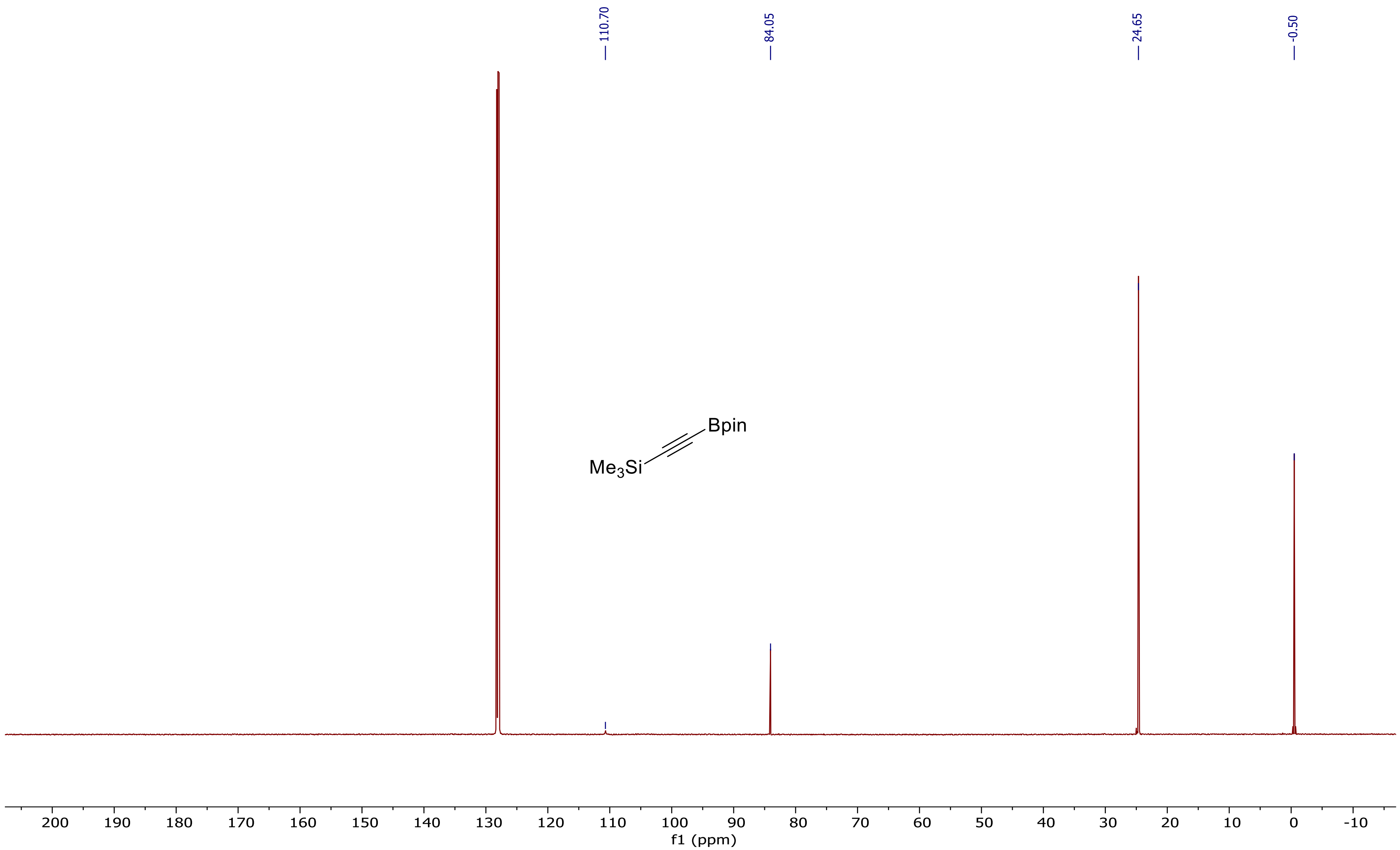

^{13}C NMR (C_6D_6, 125.77 MHz) of 4,4,5,5-Tetramethyl-2-(trimethylsilylethnynyl)-1,3,2-dioxaborolane.

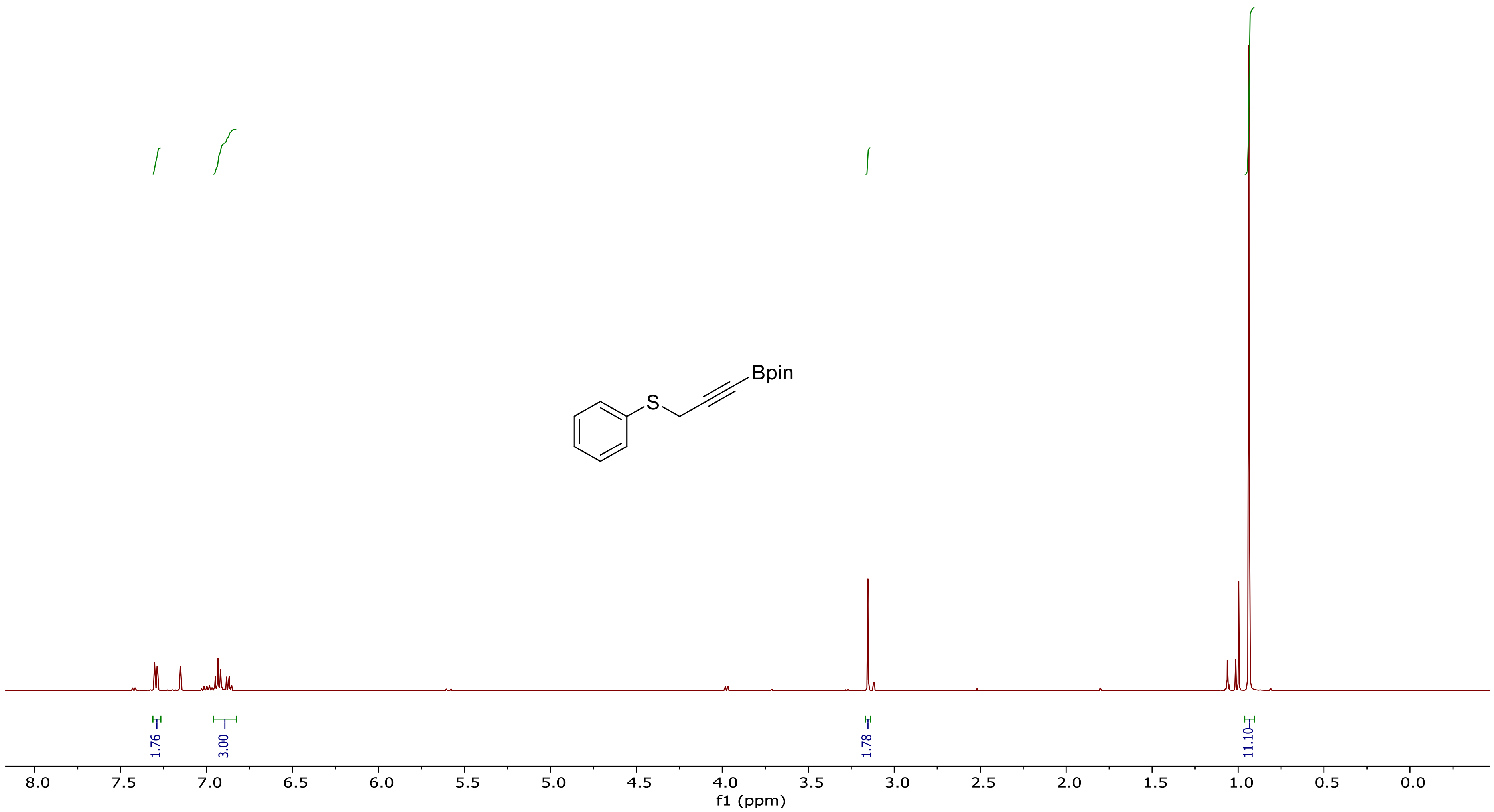

^{1}H NMR (C_6D_6, 500.12 MHz) of 4,4,5,5-Tetramethyl-2-(3-phenylsulfideprop-1-yn-1-yl)-1,3,2-dioxaborolane.

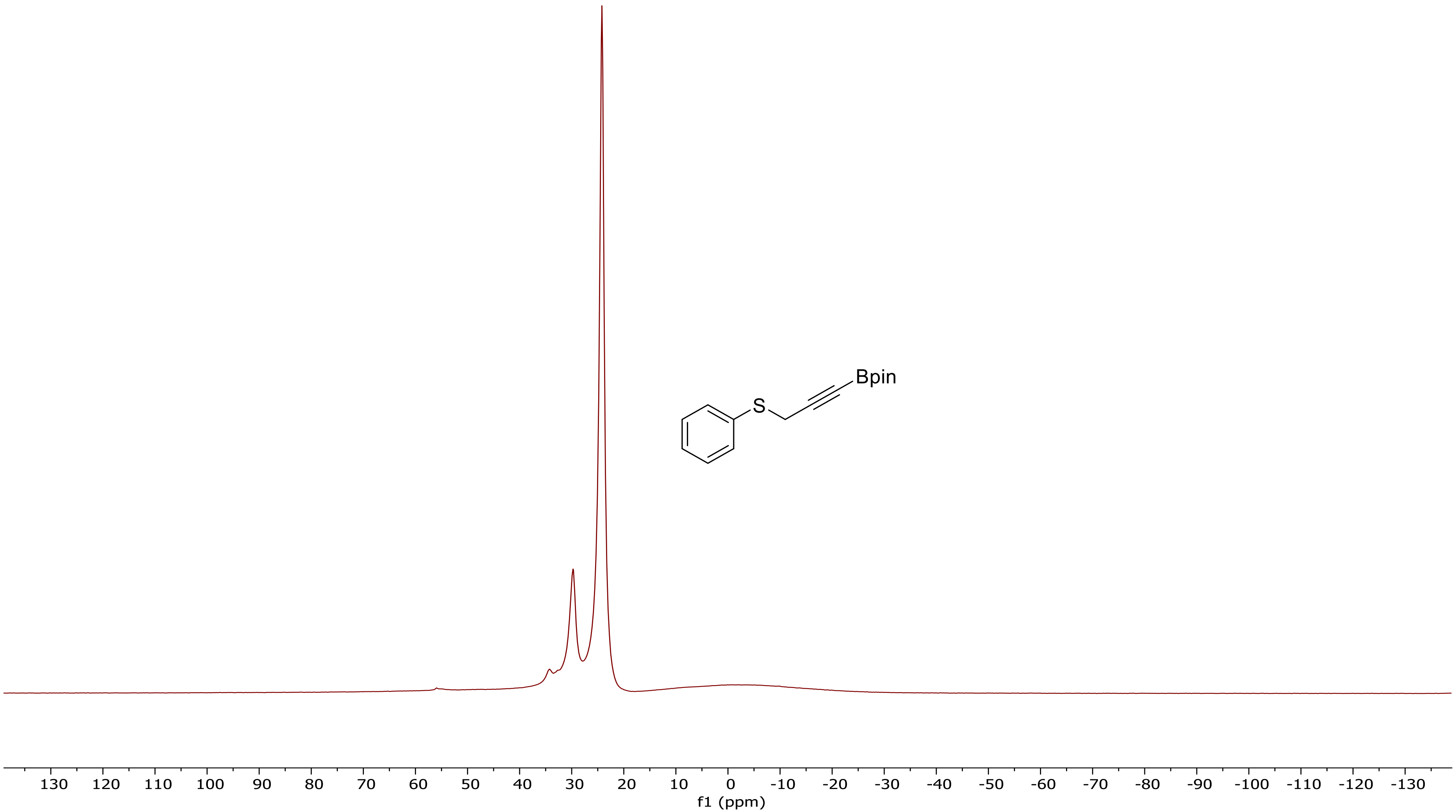

^{11}B NMR (C_6D_6, 128.34 MHz) of 4,4,5,5-Tetramethyl-2-(3-phenylsulfideprop-1-yn-1-yl)-1,3,2-dioxaborolane.

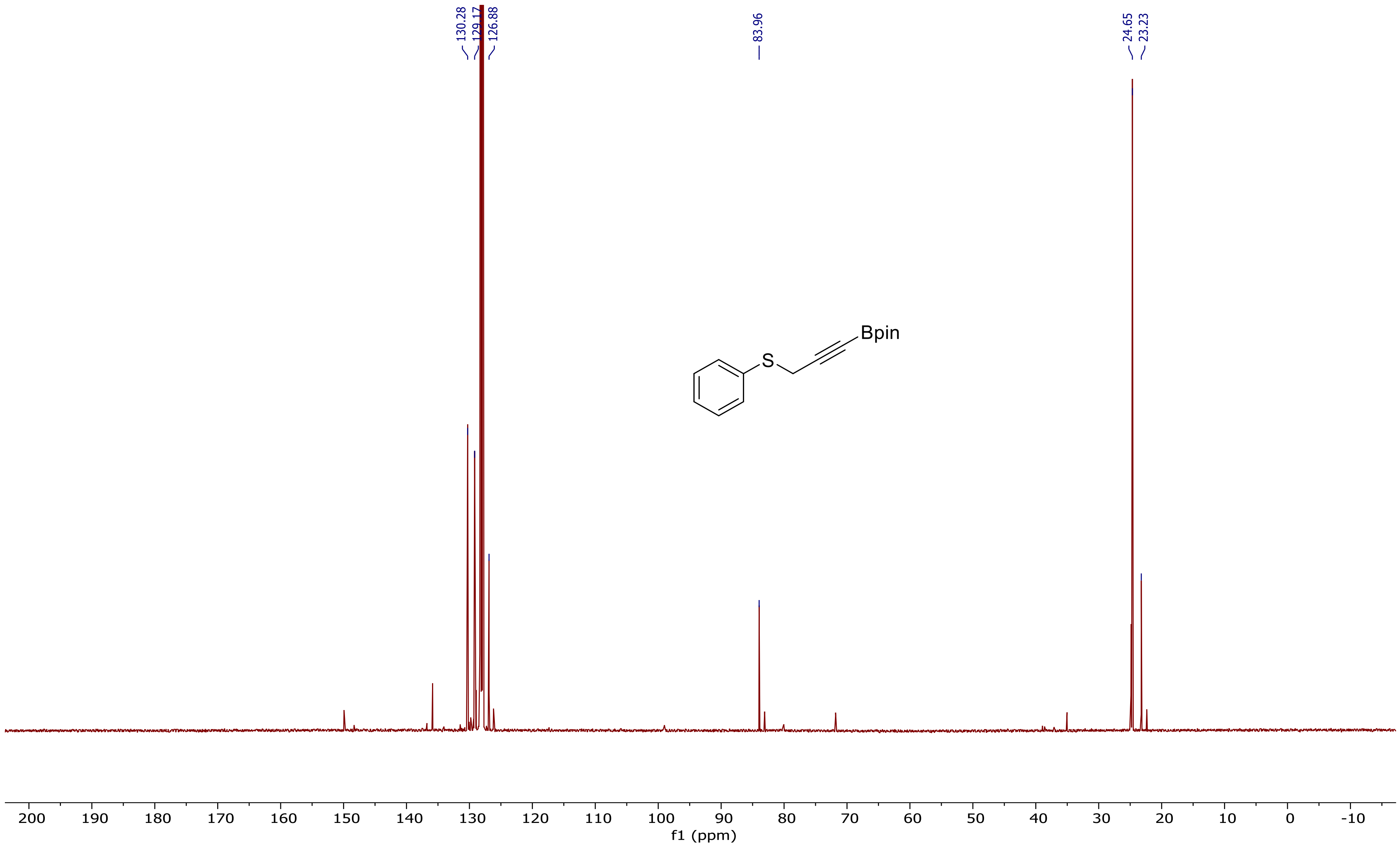

^{13}C NMR (C_6D_6, 125.77 MHz) of 4,4,5,5-Tetramethyl-2-(3-phenylsulfideprop-1-yn-1-yl)-1,3,2-dioxaborolane.

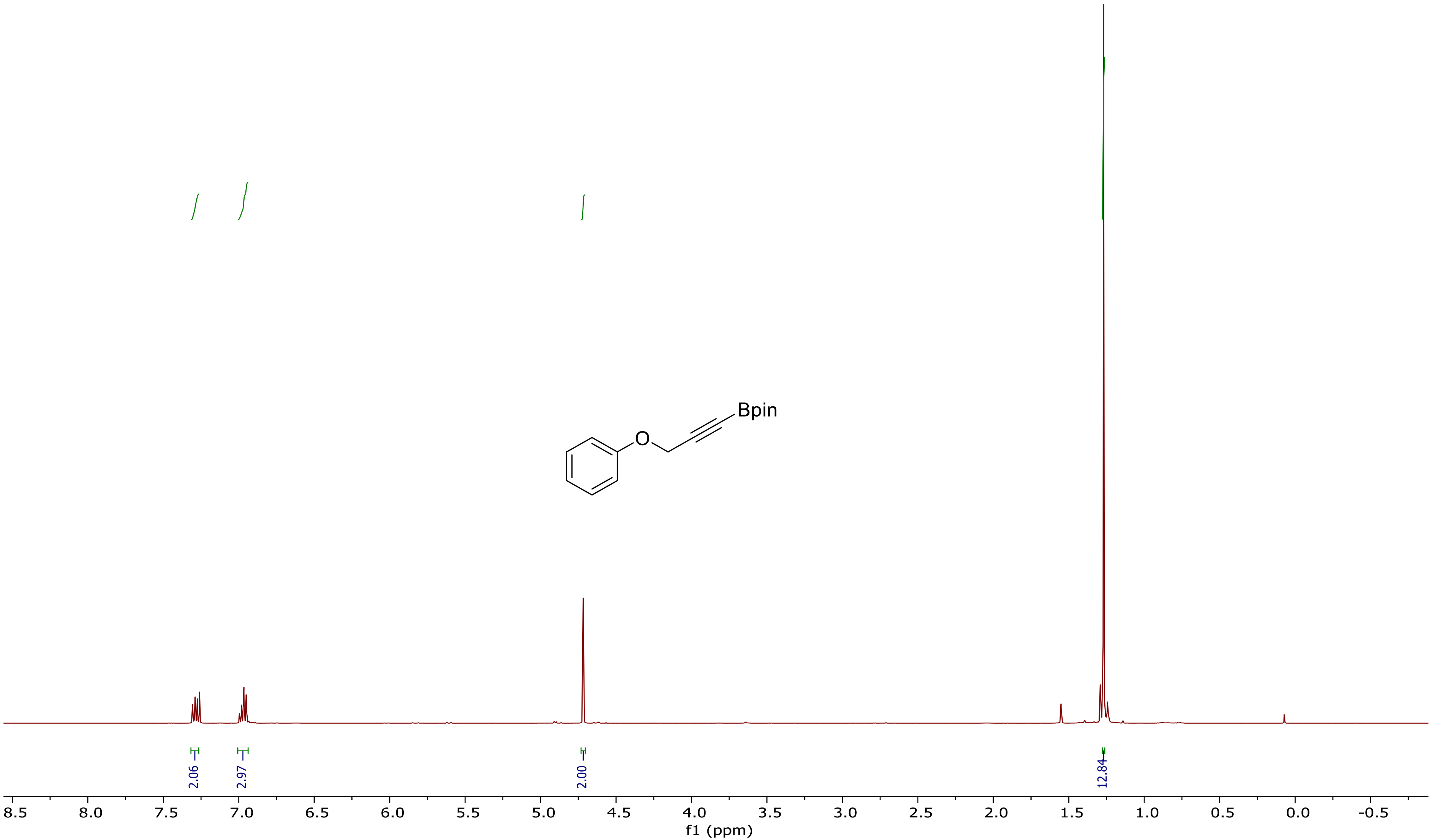

^{1}H NMR ($CDCl_3$, 500.12 MHz) of 4,4,5,5-Tetramethyl-2-(3-phenyletherprop-1-yn-1-yl)-1,3,2-dioxaborolane.

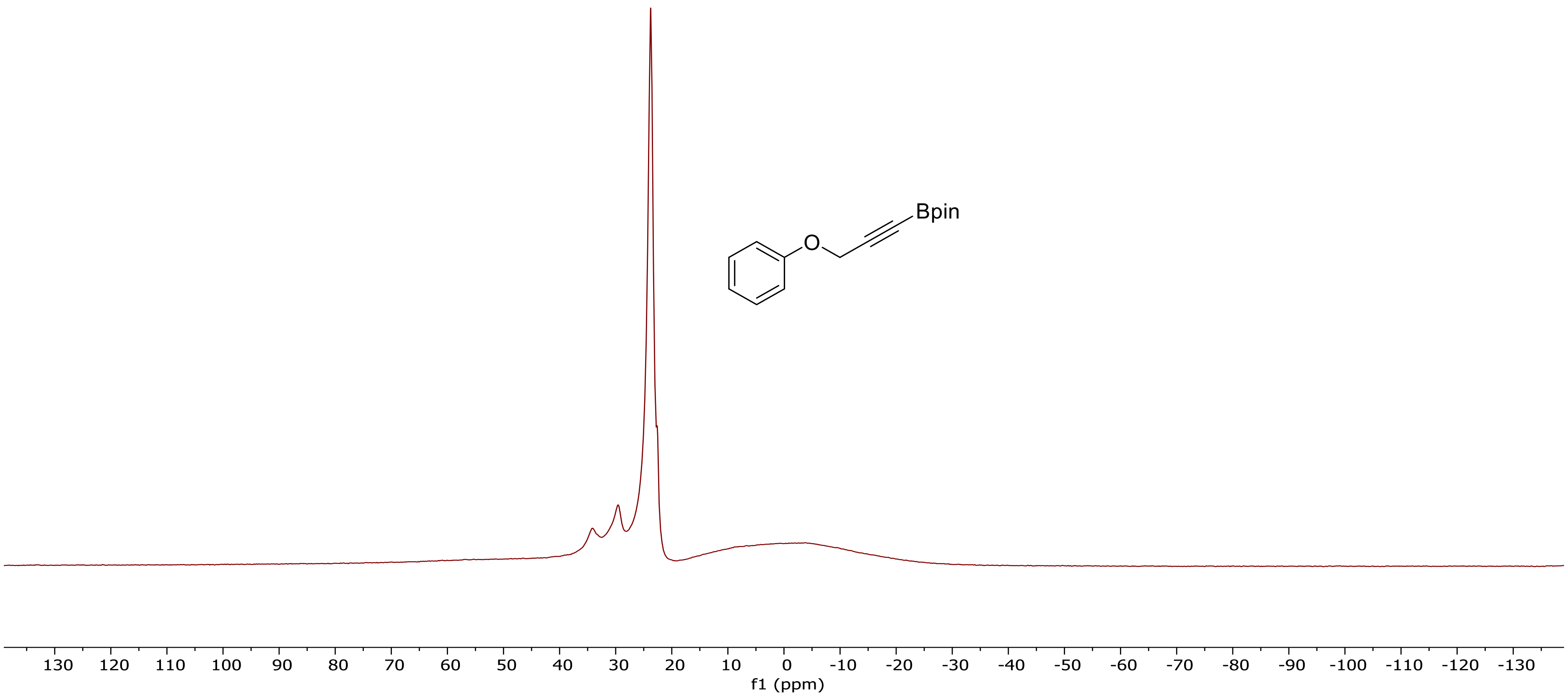

^{11}B NMR ($CDCl_3$, 128.34 MHz) of 4,4,5,5-Tetramethyl-2-(3-phenyletherprop-1-yn-1-yl)-1,3,2-dioxaborolane.

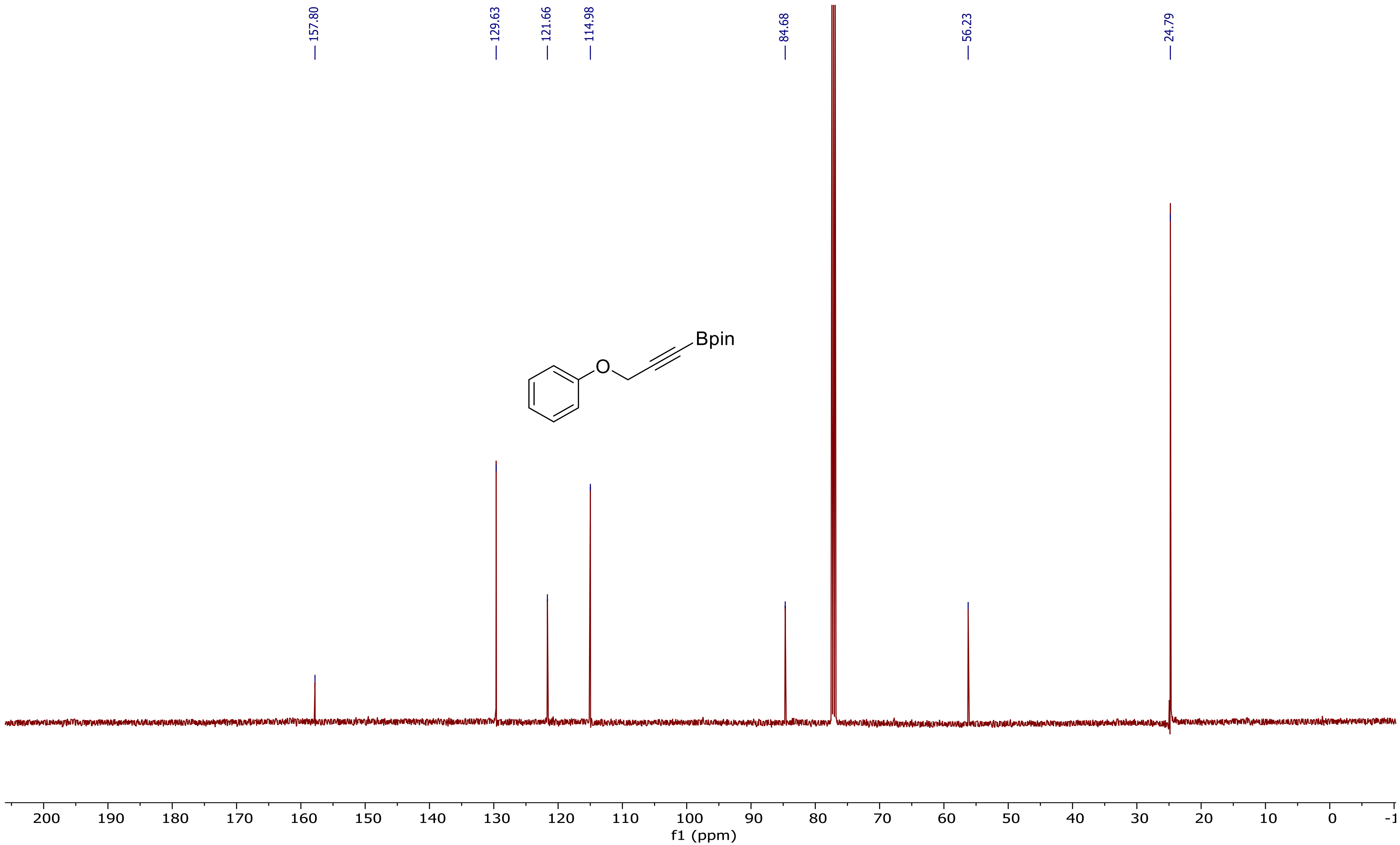

^{13}C NMR ($CDCl_3$, 125.77 MHz) of 4,4,5,5-Tetramethyl-2-(3-phenyletherprop-1-yn-1-yl)-1,3,2-dioxaborolane.

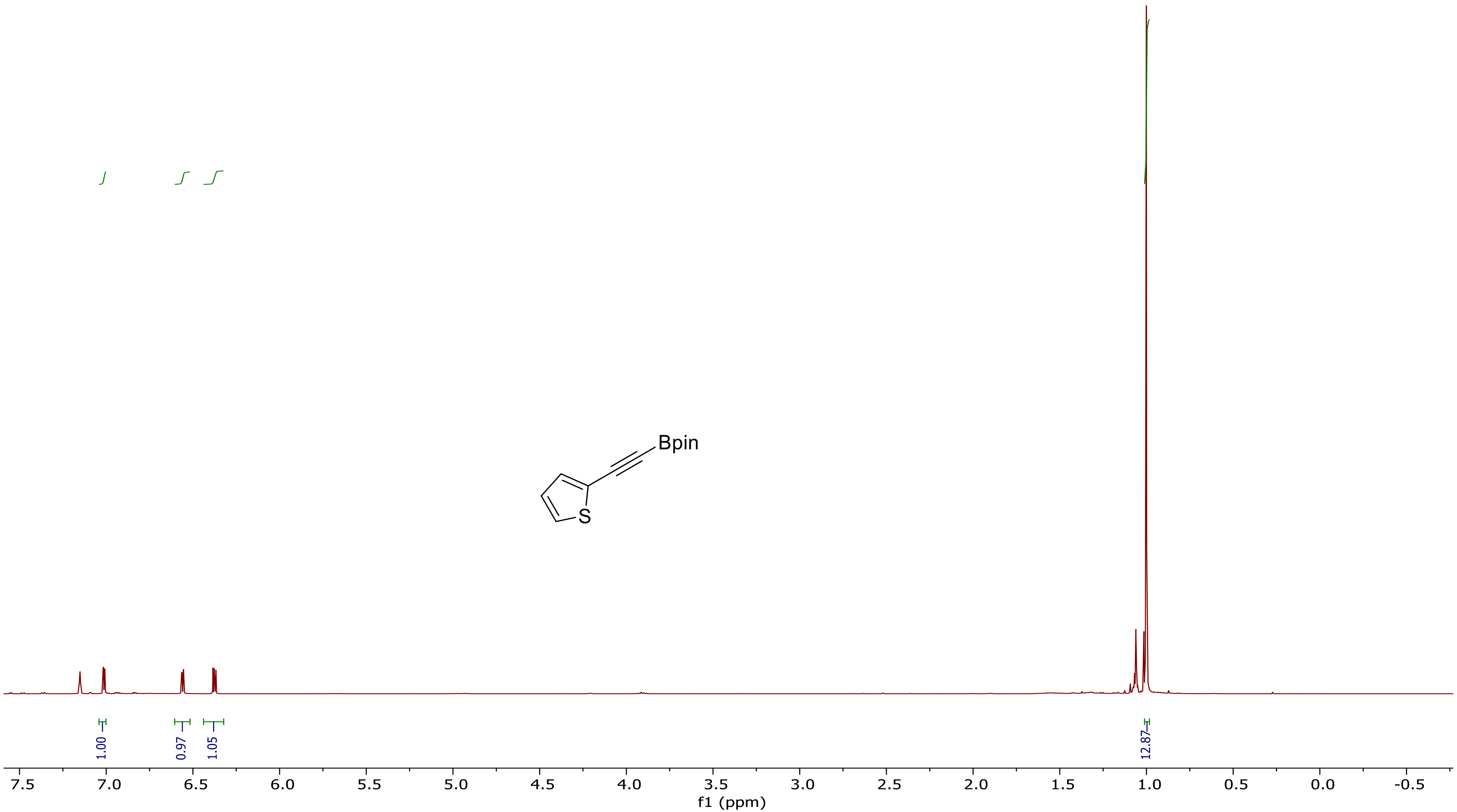

^{1}H NMR (C_6D_6, 500.12 MHz) of 4,4,5,5-Tetramethyl-2-(2-ethynylthiophene)-1,3,2-dioxaborolane.

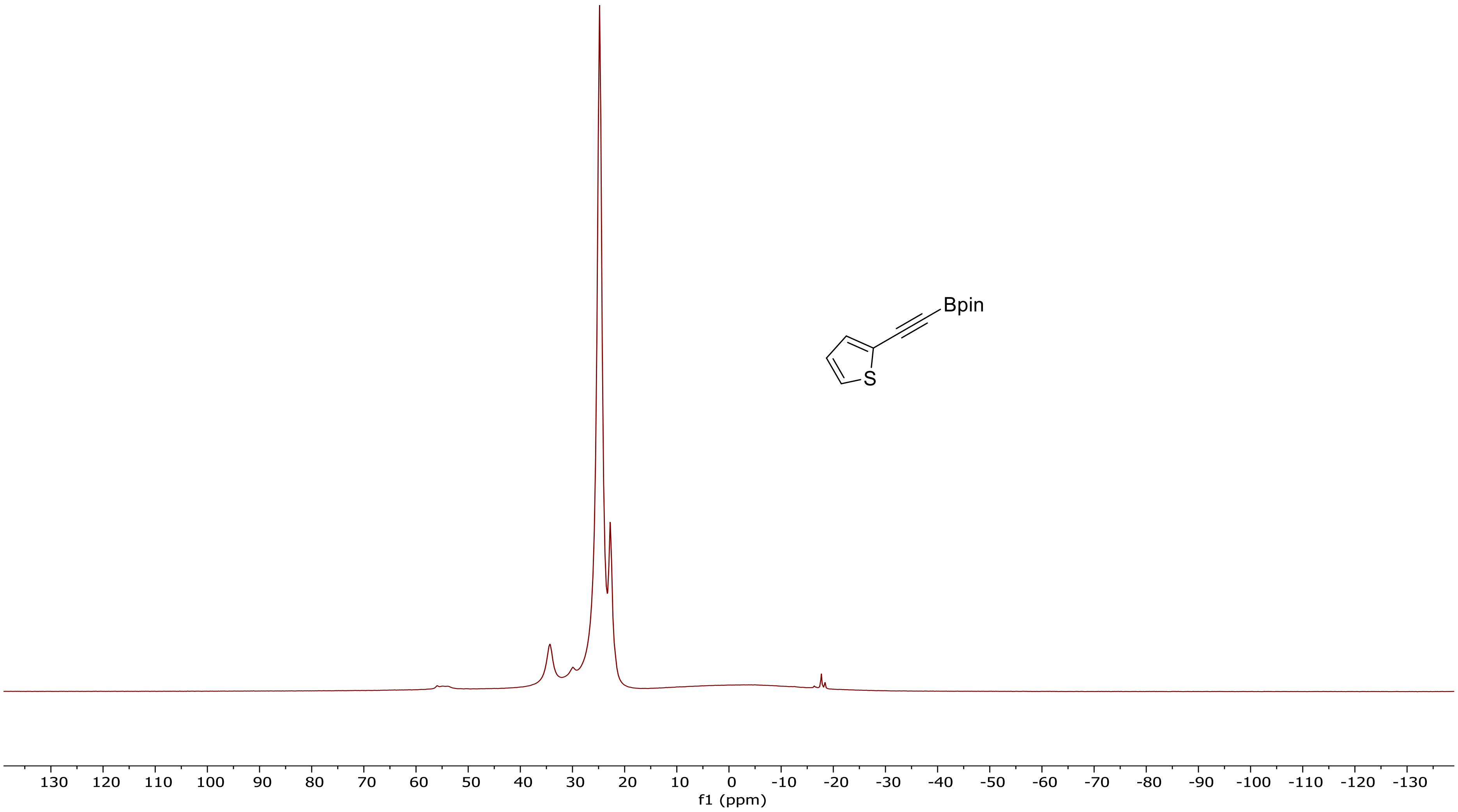

^{11}B NMR (C_6D_6, 128.34 MHz) of 4,4,5,5-Tetramethyl-2-(2-ethynylthiophene)-1,3,2-dioxaborolane.

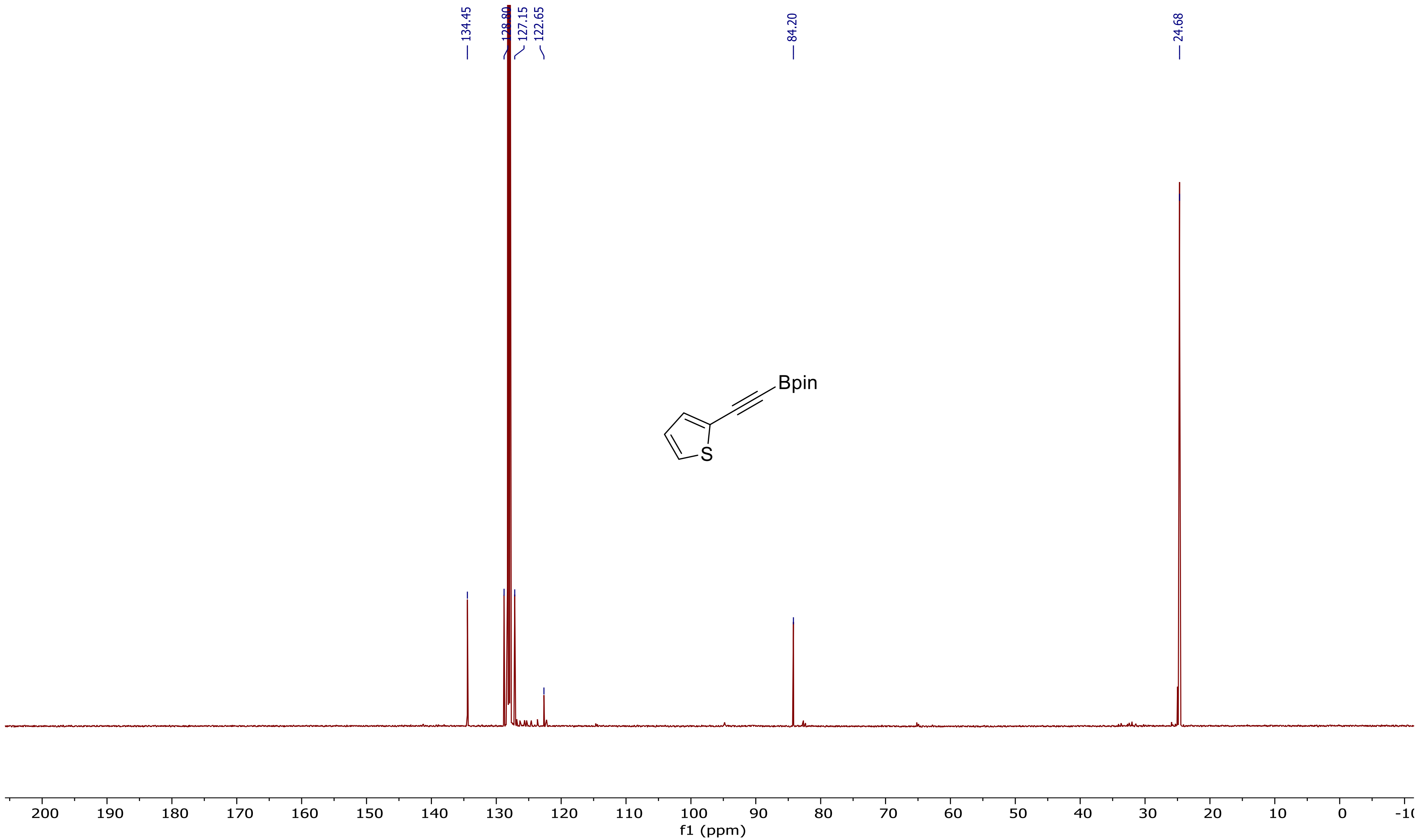

^{13}C NMR (C_6D_6, 125.77 MHz) of 4,4,5,5-Tetramethyl-2-(2-ethynylthiophene)-1,3,2-dioxaborolane.

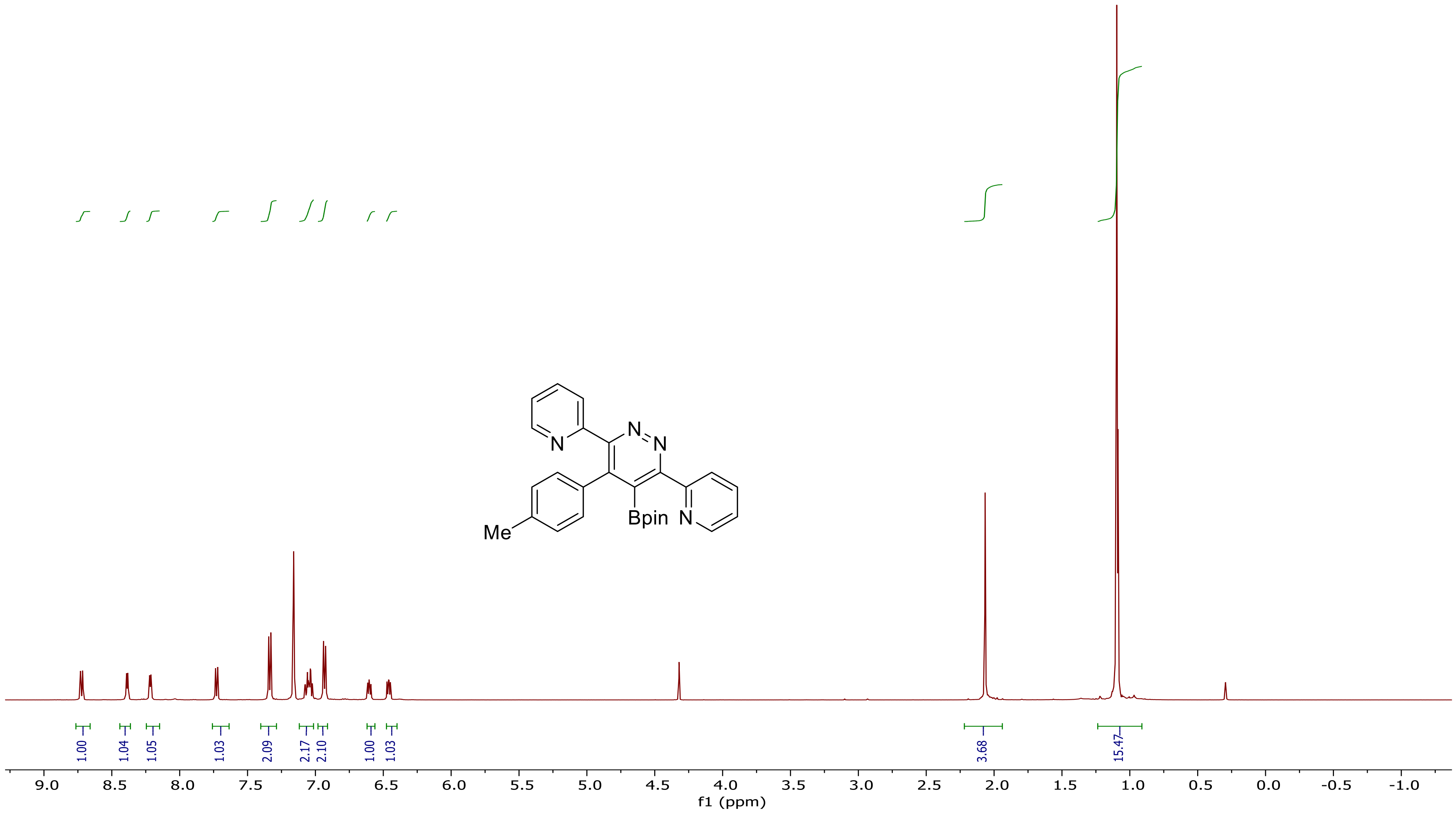

^{1}H NMR (C_6D_6, 500.12 MHz) spectrum of 3,6-bis(2-pyridyl)-4-tolyl-5-(4,4,5,5-tetramethyl[1,3,2]dioxaborolan-2-yl)pyridazine.

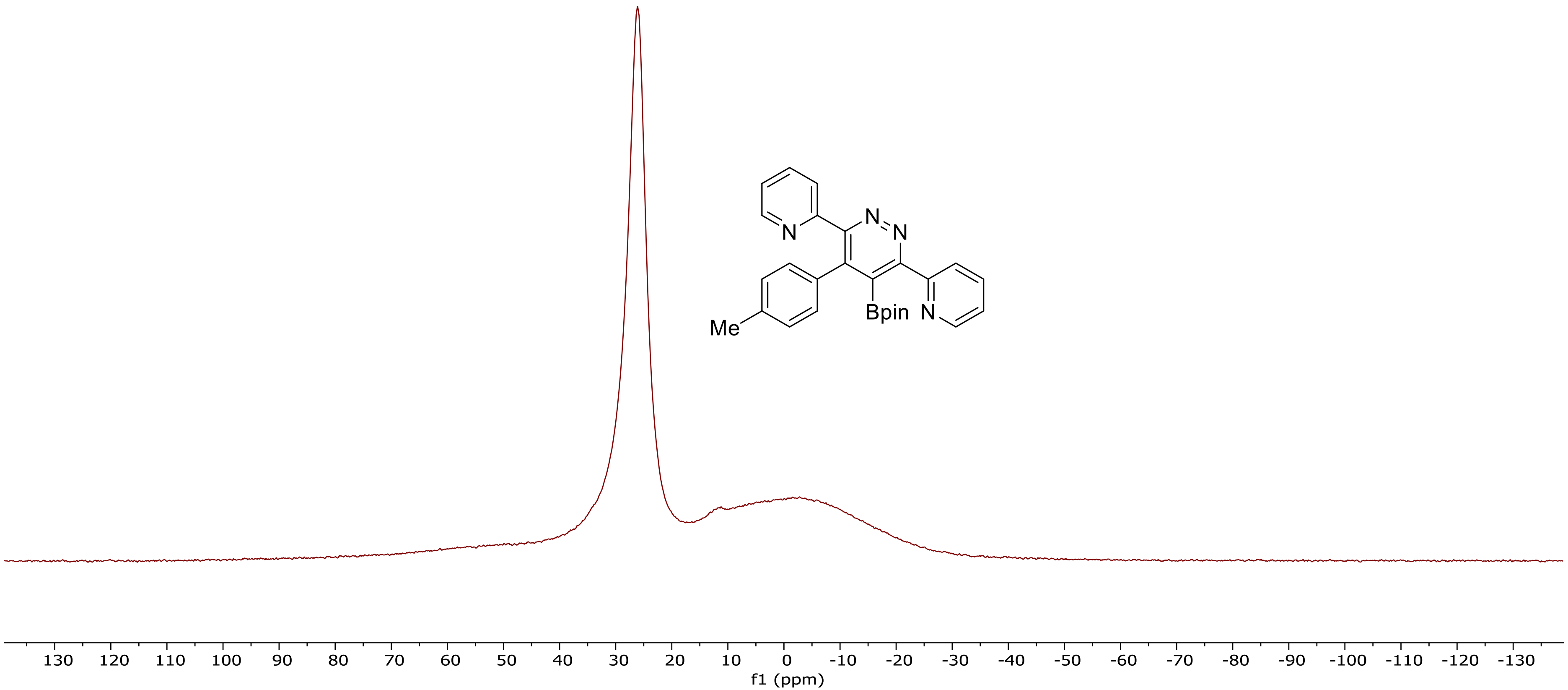

^{11}B NMR (C_6D_6, 128.34 MHz) of 3,6-bis(2-pyridyl)-4-tolyl-5-(4,4,5,5-tetramethyl[1,3,2]dioxaborolan-2-yl)pyridazine.

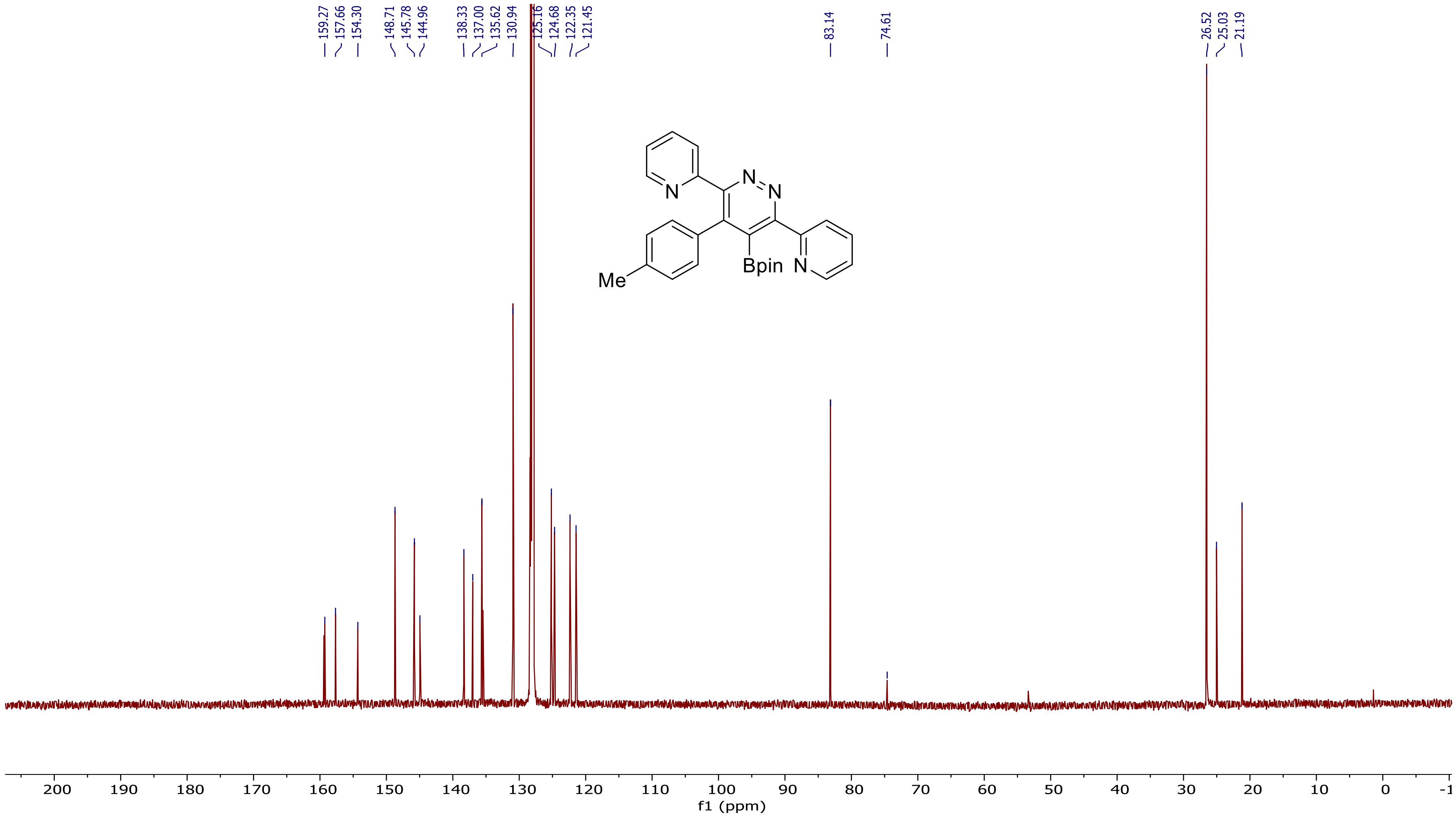

^{13}C NMR (C_6D_6, 125.77 MHz) of 3,6-bis(2-pyridyl)-4-tolyl-5-(4,4,5,5-tetramethyl[1,3,2]dioxaborolan-2-yl)pyridazine.

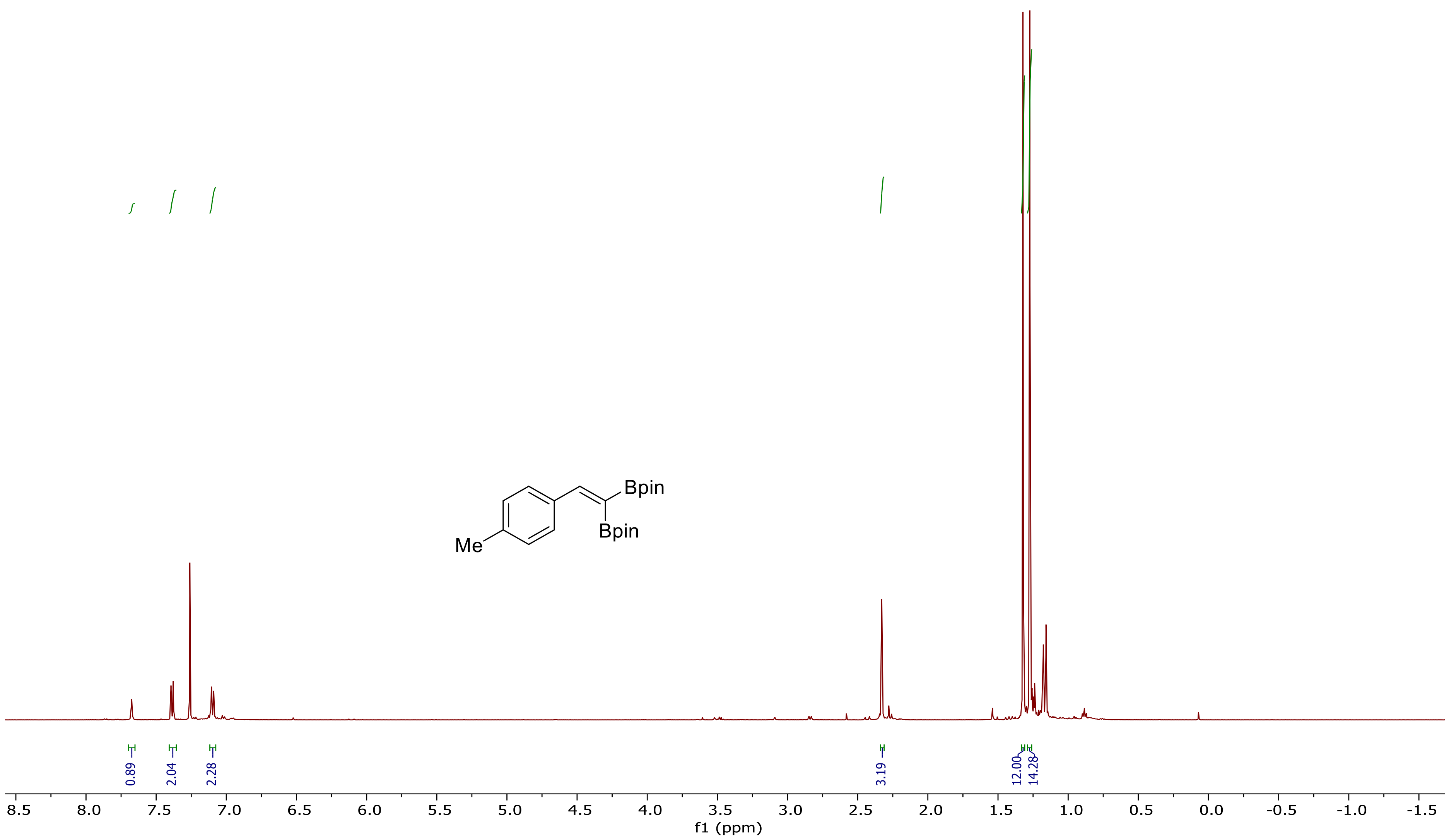

^{1}H NMR ($CDCl_3$, 500.12 MHz) spectrum of 2,2'-(2-(*p*-tolyl)ethene-1,1-diyl)bis(4,4,5,5-tetramethyl-1,3,2-dioxaborolane).

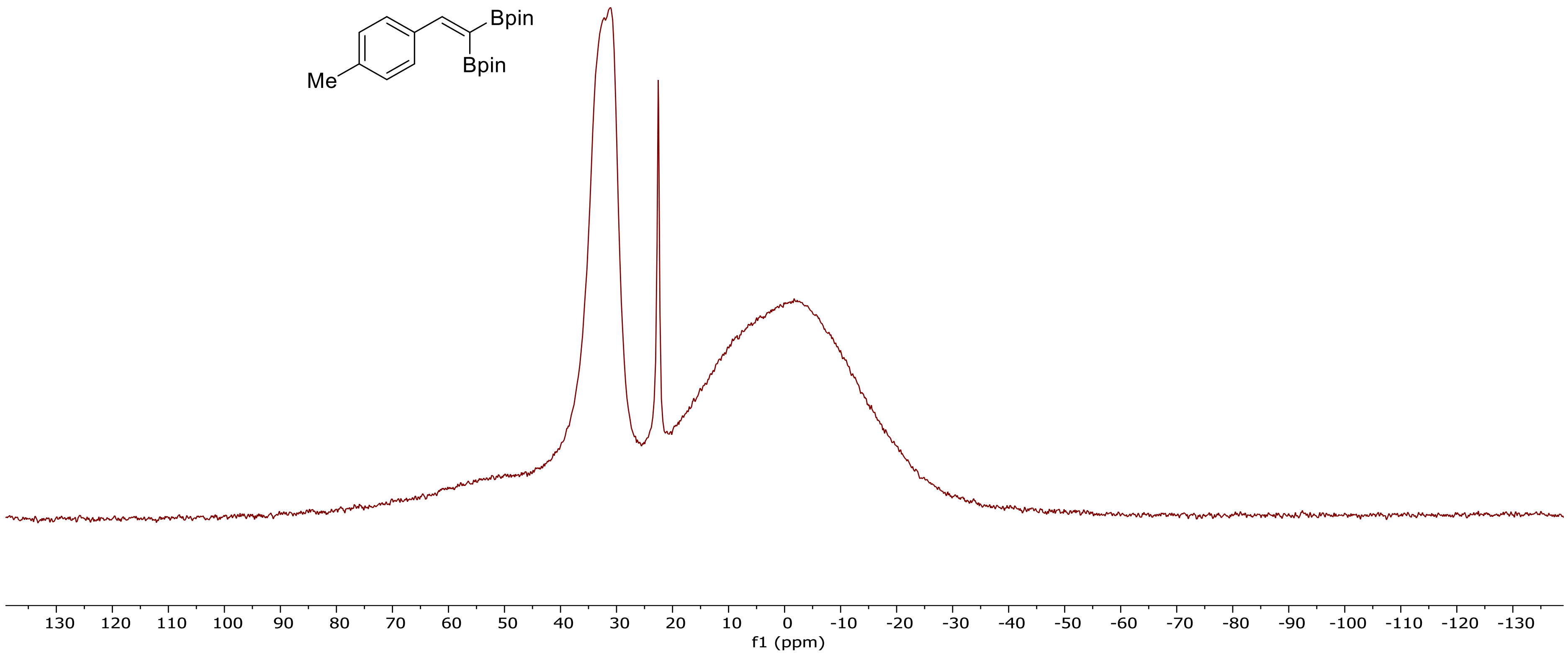

^{11}B NMR ($CDCl_3$, 128.34 MHz) of 2,2'-(2-(*p*-tolyl)ethene-1,1-diyl)bis(4,4,5,5-tetramethyl-1,3,2-dioxaborolane).

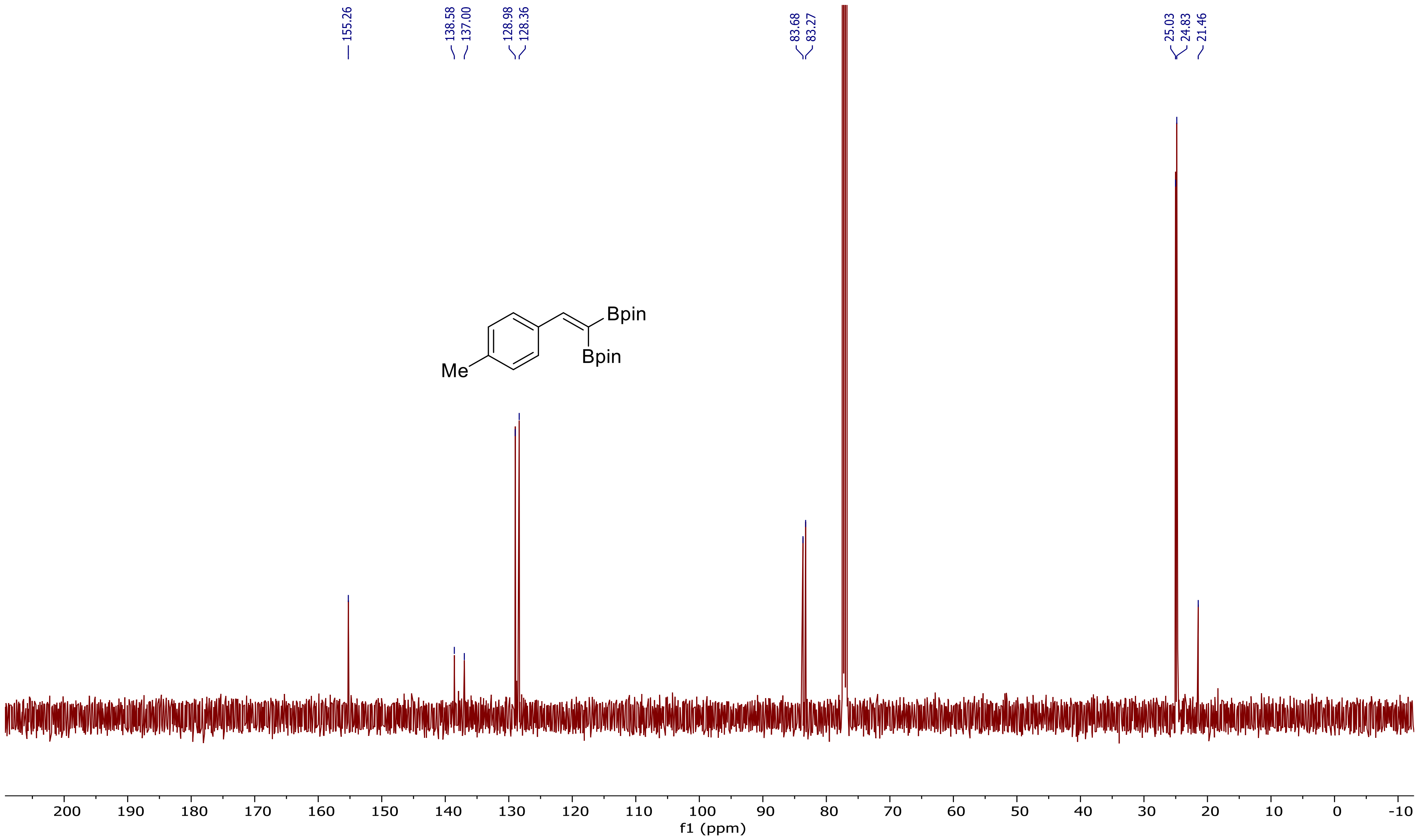

^{13}C NMR ($CDCl_3$, 125.77 MHz) of 2,2'-(2-(*p*-tolyl)ethene-1,1-diyl)bis(4,4,5,5-tetramethyl-1,3,2-dioxaborolane).

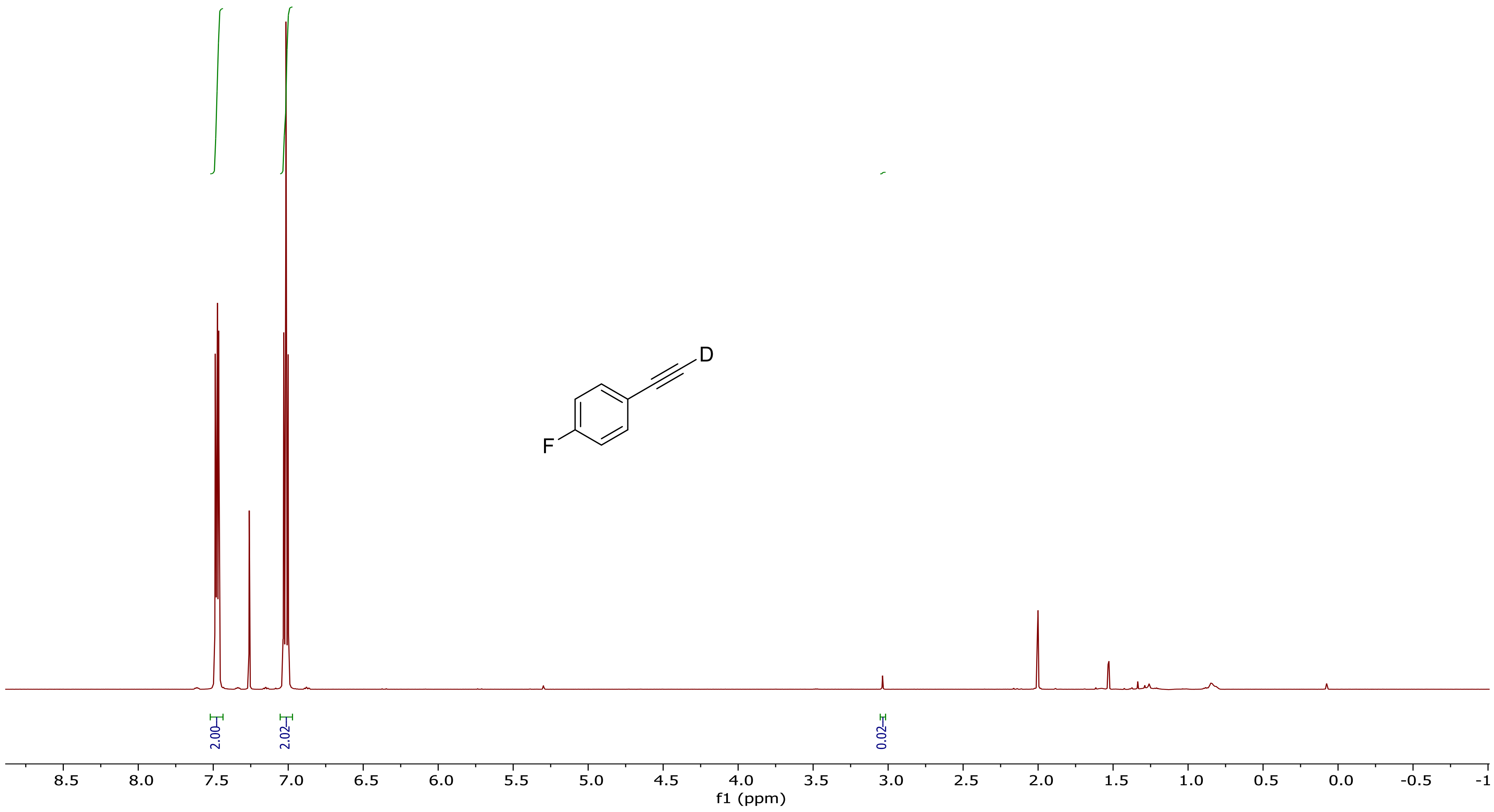

^{1}H NMR ($CDCl_3$, 500.12 MHz) spectrum of 1-deutero-4'-fluorophenylacetylene.

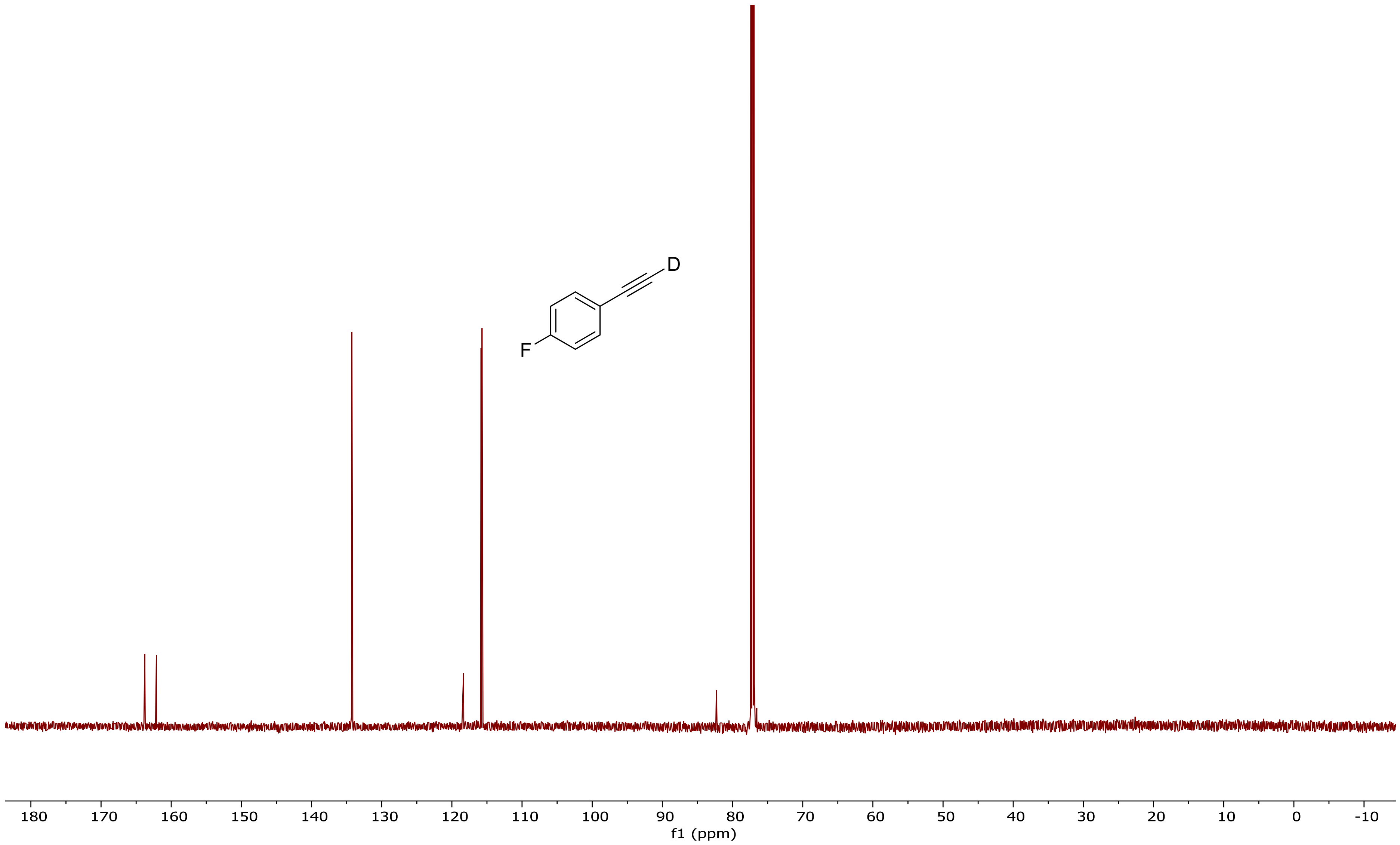

^{13}C NMR ($CDCl_3$, 125.77 MHz) of 1-deutero-4'-fluorophenylacetylene.

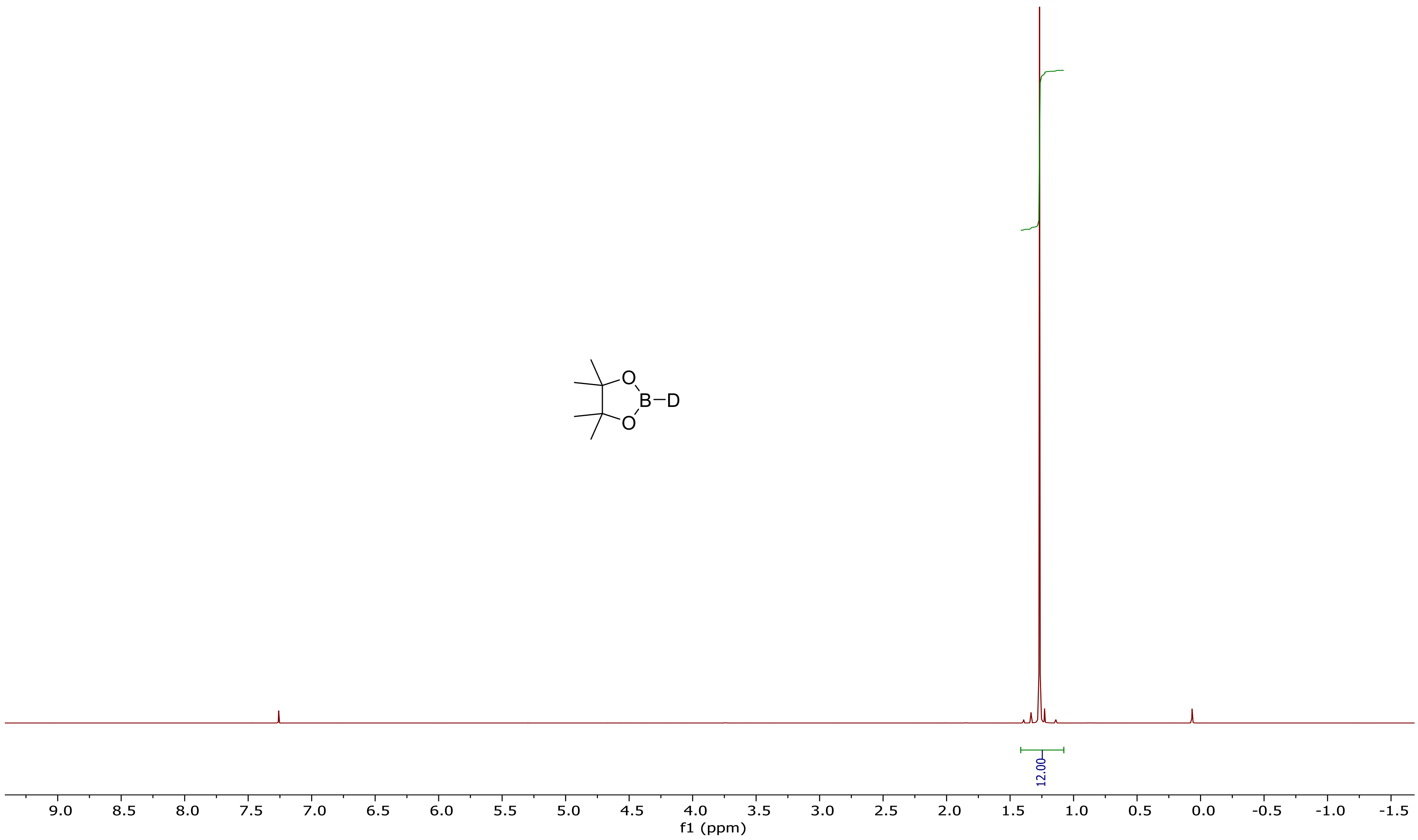

^{1}H NMR (500.12 MHz, $CDCl_3$) spectrum of DBpin.

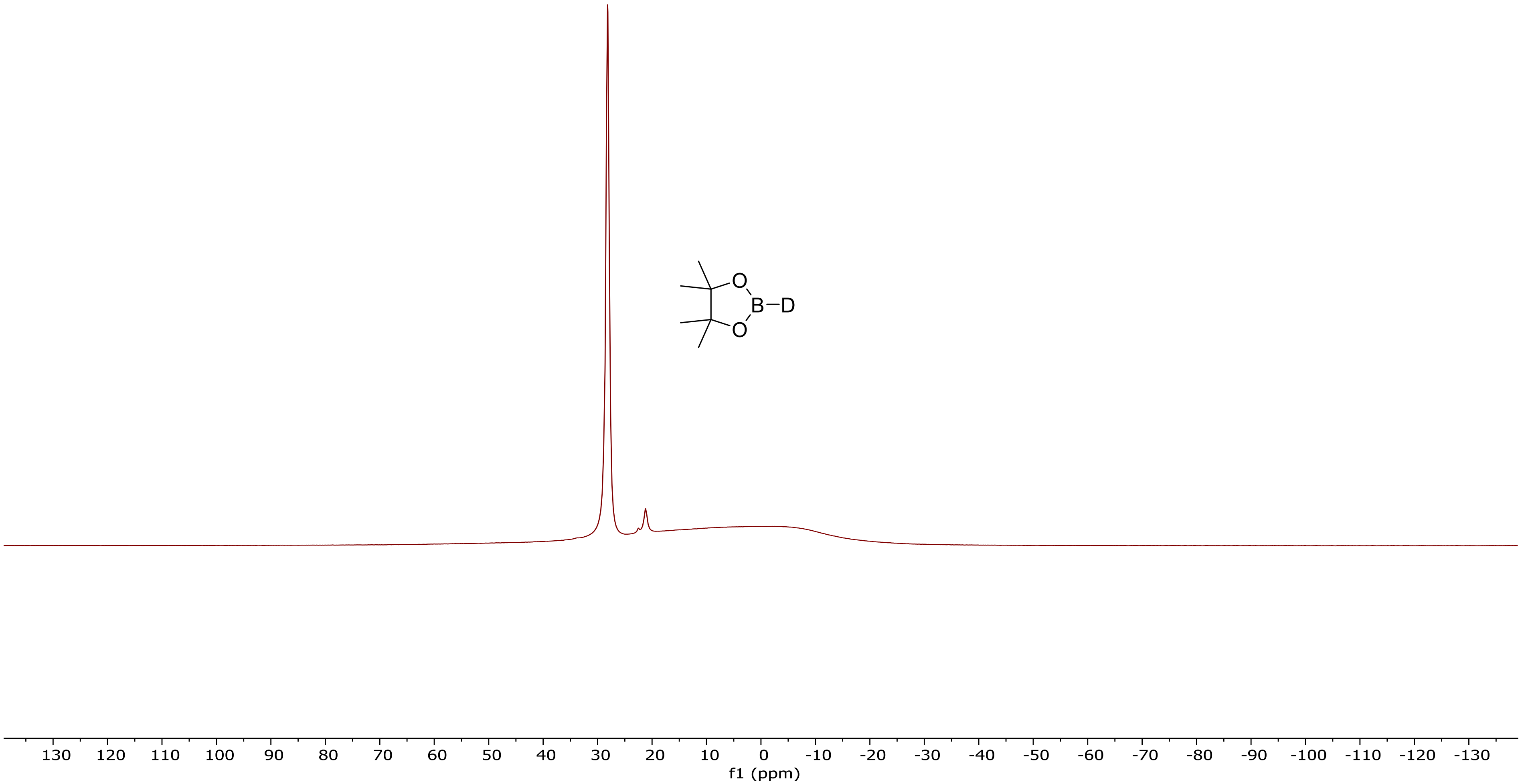

^{11}B NMR (128.34 MHz, $CDCl_3$) spectrum of DBpin.

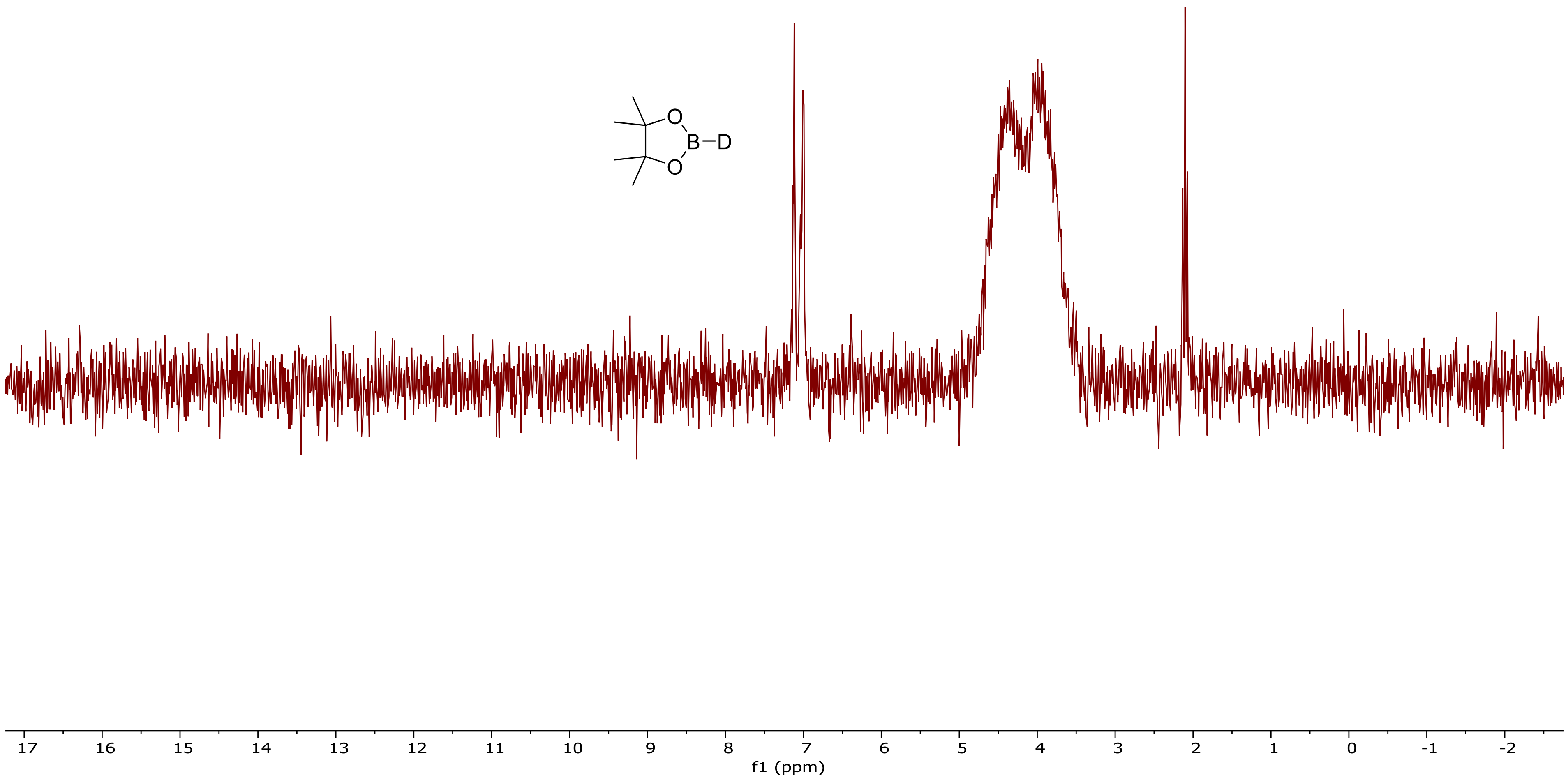

^{2}D NMR (76.75 MHz, toluene) spectrum of DBpin.

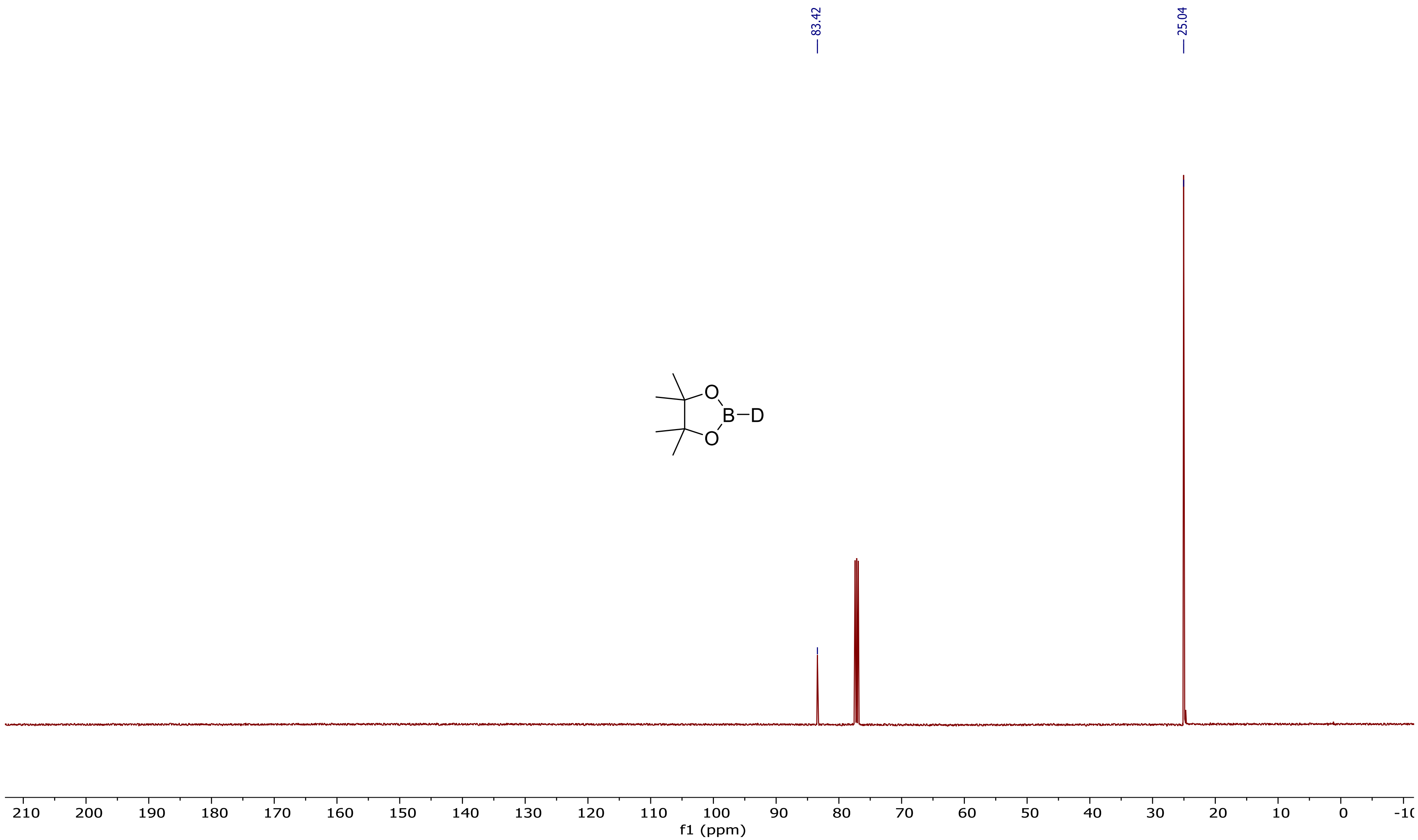

^{13}C NMR (125.77 MHz, $CDCl_3$) spectrum of DBpin.

11. References

[1] G. R. Fulmer, A. J. M. Miller, N. H. Sherden, H. E. Gottlieb, A. Nudelman, B. M. Stoltz, J. E. Bercaw, K. I. Goldberg, *Organometallics* **2010**, *29*, 2176–2179.

[2] K. Shirakawa, A. Arase, M. Hoshi, *Synthesis* **2004**, 1814–1820.

[3] D. Wei, B. Carboni, J. B. Sortais, C. Darcel, *Adv. Synth. Catal.* **2018**, *360*, 3649–3654.

[4] E. A. Romero, R. Jazzar, G. Bertrand, *Chem. Sci.* **2016**, *8*, 165–168.

[5] C. I. Lee, J. Zhou, O. V. Ozerov, *J. Am. Chem. Soc.* **2013**, *135*, 3560–3566.

[6] H. E. Ho, N. Asao, Y. Yamamoto, T. Jin, *Org. Lett.* **2014**, *16*, 4670–4673.

[7] T. León, E. Fernández, *Chem. Commun.* **2016**, *52*, 9363–9366.

[8] A. Geny, D. Lebæuf, G. Rouquié, K. P. C. Vollhardt, M. Malacria, V. Gandon, C. Aubert, *Chem. Eur. J.* **2007**, *13*, 5408–5425.

[9] K. Chernichenko, M. Nieger, M. Leskelä, T. Repo, *Dalton Trans.* **2012**, *41*, 9029–9032.

[10] S. Hirner, A. Kolb, J. Westmeier, S. Gebhardt, S. Middel, K. Harms, P. Von Zezschwitz, *Org. Lett.* **2014**, *16*, 3162–3165.

[11] S. P. Bew, G. D. Hiatt-Gipson, J. A. Lovell, C. Poullain, *Org. Lett.* **2012**, *14*, 456–459.

[12] A. W. M. Cummins, S. Li, D. R. Willcox, T. Muilu, J. H. Docherty, S. P. Thomas, *Tetrahedron* **2020**, *76*, 131084.

[13] F. A. L. Anet, D. J. O’Leary, *Tetrahedron Lett.* **1989**, *30*, 2755–2758.

[14] B. Bennett, W. T. Raynes, C. W. Anderson, *Spectrochim. Acta Part A Mol. Spectrosc.* **1989**, *45*, 821–827.

[15] J. Bures, *Angew. Chem. - Int. Ed.* **2016**, *55*, 16084–16087.

[16] D. K. Russell, T. A. Claxton, A. S. Grady, R. E. Linney, Z. Mahmood, R. D. Markwell, *J. Chem. Soc. Faraday Trans.* **1995**, *91*, 3015–3020.

[17] M. J. Frisch, G. W. Trucks, H. B. Schlegel, G. E. Scuseria, M. A. Robb, J. R. Cheeseman, G. Scalmani, V. Barone, G. A. Petersson, H. Nakatsuji, X. Li, M. Caricato, A. V. Marenich, J. Bloino, B. G. Janesko, R. Gomperts, B. Mennucci, H. P. Hratchian, J. V. Ortiz, A. F. Izmaylov, J. L. Sonnenberg, D. Williams-Young, F. Ding, F. Lipparini, F. Egidi, J. Goings, B. Peng, A. Petrone, T. Henderson, D. Ranasinghe,

V. G. Zakrzewski, J. Gao, N. Rega, G. Zheng, W. Liang, M. Hada, M. Ehara, K. Toyota, R. Fukuda, J. Hasegawa, M. Ishida, T. Nakajima, Y. Honda, O. Kitao, H. Nakai, T. Vreven, K. Throssell, J. A. Montgomery, Jr., J. E. Peralta, F. Ogliaro, M. J. Bearpark, J. J. Heyd, E. N. Brothers, K. N. Kudin, V. N. Staroverov, T. A. Keith, R. Kobayashi, J. Normand, K. Raghavachari, A. P. Rendell, J. C. Burant, S. S. Iyengar, J. Tomasi, M. Cossi, J. M. Millam, M. Klene, C. Adamo, R. Cammi, J. W. Ochterski, R. L. Martin, K. Morokuma, O. Farkas, J. B. Foresman, D. J. Fox, Gaussian, Inc., Wallingford CT, **2016**.

[18] J. Da Chai, M. Head-Gordon, *Phys. Chem. Chem. Phys.* **2008**, *10*, 6615–6620.

[19] A. V. Marenich, C. J. Cramer, D. G. Truhlar, *J. Phys. Chem. B* **2009**, *113*, 6378–6396.

[20] “Chemcraft - graphical software for visualization of quantum chemistry computations.,” can be found under https://www.chemcraftprog.com.
